# Supplementary material for: Effect of intra-dialytic pedaling exercise on dialysis adequacy: A randomized controlled trial
Source: PLoS One. 2026 May 15;21(5):e0348063. doi: 10.1371/journal.pone.0348063 (PMC13178916; doi:10.1371/journal.pone.0348063)
Supplement: S2 File — This file contains the full Persian-language thesis of the Master of Nursing student whose research underpinned the current manuscript. It contains the complete trial protocol and data documentation. (PDF) [file pone.0348063.s002.pdf]

بسم الله الرحمن الرحیم

دانشگاه علوم پزشکی و خدمات بهداشتی درمانی بوشهر
دانشکده پرستاری و مامایی
گروه پرستاری

پایان‌نامه کارشناسی ارشد پرستاری داخلی جراحی

مقایسه اثربخشی فعالیت فیزیکی حین دیالیز بر کفایت دیالیز، شاخص‌های همودینامیک و خستگی در بیماران تحت همودیالیز در بیمارستان‌های شهر بوشهر در سال ۱۳۹۹

دانشجو
محمود محمدی زاده

استاد راهنما
دکتر شهناز پولادی

اساتید مشاور
شریف شریفی
دکتر نیلوفر معتمد

بهمن ماه ۱۴۰۰

شماره: ۲۰/۱۸/۴۴۲۳۹/دپ
تاریخ : ۱۴۰۰/۱۱/۲۵
پیوست: .....دارد.......

صورتجلسه دفاع از پایان نامه ارشد

فرم شماره ۱۱

****

جلسه دفاعیه پایان نامه تحصیلی **آقای محمود محمدی زاده** دانشجوی کارشناسی ارشد رشته **پرستاری داخلی جراحی** با موضوع پایان نامه " مقایسه اثربخشی فعالیت فیزیکی حین دیالیز بر کفایت دیالیز، شاخص‌های همودینامیک و خستگی در بیماران تحت همودیالیز در بیمارستان‌های شهر بوشهر در سال ۱۳۹۹ " به راهنمایی **دکتر شهناز پولادی** مورخ ۱۴۰۰/۱۱/۰۴ ساعت ۱۲:۰۰ با حضور اعضای محترم هیات داوران متشکل از:

سمت	نام و نام خانوادگی	امضاء
استاد راهنما	دکتر شهناز پولادی	حاضر
استاد مشاور	جناب آقای شریف شریفی	غایب
استاد مشاور	دکتر نیلوفر معتمد	حاضر
استاد داور	دکتر محمدرضا یزدانخواه فرد	حاضر
استاد داور	دکتر سودابه زنده بودی	غایب
نماینده تحصیلات تکمیلی دانشکده	دکتر مریم روانی پور	حاضر
نماینده تحصیلات تکمیلی دانشگاه	آقای سید علی هاشمی فرد	حاضر

تشکیل گردید و ضمن ارزیابی به شرح پیوست با درجه **عالی** و نمره **۱۸/۳۵** مورد تأیید قرار گرفت.

مدیر تحصیلات تکمیلی دانشگاه

بسم الله الرحمن الرحیم

وزارت بهداشت، درمان و آموزش پزشکی

دانشگاه علوم پزشکی و خدمات بهداشتی درمانی بوشهر

دانشکده پرستاری و مامایی

جمهوری اسلامی ایران
وزارت بهداشت، درمان و آموزش پزشکی

پایان‌نامه کارشناسی ارشد **محمود محمدی زاده** در رشته **پرستاری داخلی- جراحی** به شماره دانشجویی **۹۶۱۴۴۰۱** فارغ التحصیل سال **۱۴۰۰** که با عنوان **" مقایسه اثربخشی فعالیت فیزیکی حین دیالیز بر کفایت دیالیز، شاخص‌های همودینامیک و خستگی در بیماران تحت همودیالیز در بیمارستان‌های شهر بوشهر در سال ۱۳۹۹"** زیر نظر **خانم دکتر شهناز پولادی** نگاشته شده و به شماره **۱۹۲۸۵** در دفتر پایان نامه‌های این دانشکده به ثبت رسیده و در تاریخ **۱۴۰۰/۱۱/۰۴** با درجه **عالی** مورد ارزیابی هیأت داوران قرار گرفته است.

هیأت داوران

ردیف	نام و نام خانوادگی	مرتبه علمی	سمت در هیأت	امضاء
۱	دکتر شهناز پولادی	دانشیار	استاد راهنما	
۲	شریف شریفی	مربی	استاد مشاور	
۳	دکتر نیلوفر معتمد	دانشیار	استاد مشاور	
۴	دکتر محمدرضا یزدانخواه‌فرد	استادیار	استاد داور	
۵	دکتر سودابه زنده‌بودی	استادیار	استاد داور	

تعهدنامه

عنوان پایان نامه: " **مقایسه اثربخشی فعالیت فیزیکی حین دیالیز بر کفایت دیالیز، شاخص‌های همودینامیک و خستگی در بیماران تحت همودیالیز در بیمارستان‌های شهر بوشهر در سال ۱۳۹۹"**

اینجانب **محمود محمدی زاده** دانشجوی **کارشناسی ارشد** رشته **پرستاری داخلی ـــ جراحی** دانشکده **پرستاری و مامایی** دانشگاه علوم پزشکی و خدمات بهداشتی درمانی بوشهر تحت راهنمایی **دکتر شهناز پولادی** متعهد می‌شوم:

- نتایج ارائه شده در این پایان نامه حاصل مطالعات علمی و عملی اینجانب بوده، مسئولیت صحت و اصالت مطالب مندرج را به‌طور کامل برعهده می‌گیرم.

- در خصوص استفاده از نتایج پژوهش‌های محققان دیگر به مرجع مورد نظر استناد شده است.

- مطالب مندرج در این پایان نامه را اینجانب یا فرد دیگری به منظور اخذ هیچ نوع مدرک یا امتیازی تاکنون به هیچ مرجعی تسلیم نکرده است.

- کلیه حقوق معنوی این اثر متعلق به دانشگاه علوم پزشکی و خدمات بهداشتی درمانی بوشهر است. مقالات مستخرج شده از پایان نامه، ذیل نام دانشگاه علوم پزشکی و خدمات بهداشتی درمانی بوشهر (Bushehr University of Medical Sciences) به چاپ خواهد رسید.

- حقوق معنوی تمام افرادی که در به دست آمدن نتایج اصلی پایان نامه تأثیرگذار بوده‌اند در مقالات مستخرج از رساله رعایت خواهد شد.

در خصوص استفاده از موجودات زنده یا بافت‌های آن‌ها برای انجام پایان‌نامه، کلیه ضوابط و اصول اخلاقی مربوطه رعایت شده است.

تاریخ

نام و امضاء دانشجو

مالکیت نتایج و حق نشر

- کلیه حقوق معنوی این اثر و محصولات آن (اعم از مقالات مستخرج، برنامه‌های رایانه‌ای، نرم‌افزارها و تجهیزات ساخته شده و مانند آن) به دانشگاه علوم پزشکی و خدمات بهداشتی درمانی بوشهر تعلق دارد و بدون اخذ اجازه کتبی از دانشگاه قابل واگذاری به شخص ثالث نیست.
- استفاده از اطلاعات و نتایج این پایان‌نامه بدون ذکر مرجع مجاز نیست.

چکیده

مقدمه: بیماران با نارسایی مزمن کلیوی تحت همودیالیز، با کاهش عملکرد و کاهش کیفیت زندگی همراه هستند؛ و فعالیت و بازتوانی جهت پیشگیری از کاهش قدرت عضلانی و کاهش عملکرد بیمار تحت دیالیز بسیار مهم می‌باشند. از طرفی دیگر پیش‌آگهی طولانی‌مدت بیماران دیالیزی کاملاً به کیفیت دیالیز آن‌ها وابسته می‌باشد. لذا مطالعه حاضر با هدف مقایسه اثربخشی فعالیت فیزیکی حین دیالیز بر کفایت دیالیز، شاخص‌های همودینامیک و خستگی در بیماران تحت همودیالیز در بیمارستان‌های شهر بوشهر در سال ۱۳۹۹ طراحی گردید.

مواد و روش‌ها: پژوهش حاضر یک مطالعه تجربی شاهددار است که بر روی ۸۴ نفر از بیماران تحت همودیالیز در بیمارستان‌های شهر بوشهر به صورت تخصیص تصادفی ساده در دو گروه آزمون (۴۲ نفر) و کنترل (۴۲ نفر) انجام شده است. در هردو گروه زمان دیالیز ۴ ساعت بود. فعالیت فیزیکی بیمار به صورت پدال زدن در بستر به مدت ۱۵ دقیقه در ۲ نوبت و با در نظر گرفتن ۱۵ دقیقه استراحت در بین تمرینات انجام گردید. متغیرهای مطالعه، قبل و بعد از مداخله با استفاده از ابزارهایی که روایی و پایایی آن بررسی گردید موردسنجش قرار گرفت. به منظور تجزیه‌وتحلیل داده‌ها، از روش‌های آمار توصیفی و استنباطی شامل آزمون مجذور کای دو، تست دقیق فیشر، من ویتنی یو و آزمون t تست با استفاده از نرم افزار SPSS.ver24 انجام شد.

یافته‌ها: یافته‌ها نشان داد که بین بیماران گروه آزمون و کنترل از لحاظ اطلاعات جمعیت شناختی بجز در سن بیماران (p=۰/۰۲) و تحصیلات (p=۰/۰۲) تفاوت آماری معناداری وجود نداشت. نتایج نشان داد که تفاوت معناداری بین دو گروه از نظر میزان اوره خون (p=۰/۱۳)، تغییرات نیتروژن اوره خون (BUN) (p=۰/۰۷) و وزن (p=۰/۸۲) بیماران وجود نداشت؛ با تعدیل اثر سن و تحصیلات، میانگین تغییرات شاخص‌های همودینامیک (درجه حرارت، نبض، فشارخون، تنفس و درصد اشباع اکسیژن خون شریانی) بین گروه آزمون و کنترل نشان داد که تفاوت معناداری بین دو گروه وجود ندارد (p>۰/۰۵)؛ همچنین مقایسه میانگین Kt/V بیماران بین گروه آزمون و کنترل تفاوت معناداری را نشان نداد (p=۰/۱۱)؛ میانگین خستگی بیمار قبل از مداخله (p=۰/۰۰۱) و در بعد از اتمام مداخله (p=۰/۰۰۱) بین گروه آزمون و کنترل تفاوت معناداری را نشان داد. به‌طوری که میانگین خستگی بیمار در گروه آزمون کمتر از گروه کنترل بود.

نتیجه گیری: نتایج مطالعه حاکی از آن است که فعالیت فیزیکی حین همودیالیز بر کفایت دیالیز و تغییرات شاخص‌های همودینامیک در بیماران تحت همودیالیز مؤثر نبوده است؛ اما فعالیت فیزیکی حین همودیالیز موجب شده بود که خستگی بیماران تحت همودیالیز افزایش یابد. لذا به نظر می‌رسد فعالیت فیزیکی می‌تواند به عنوان یک مداخله در بهبود شرایط بیماران تحت همودیالیز در نظر گرفته شود اما با توجه به عدم تأثیرگذاری بر کفایت دیالیز و افزایش خستگی بیماران همودیالیز، بایستی به نوع مداخله، مدت و زمان آن توجه ویژه‌ای شود.

کلمات کلیدی: خستگی، شاخص‌های همودینامیک، فعالیت فیزیکی، کفایت دیالیز، نارسایی مزمن کلیوی، همودیالیز

سپاسگزاری

خدای بزرگ را شاکرم که توفیق رسیدن بدین نقطه را ارزانیم داشت و در دیگری از دنیای معرفت به رویم گشود و شوق دانستن دانستنی‌ها و درک فهمیدنی‌ها را در جانم به ودیعه گذاشت.

اکنون صمیمانه مراتب سپاس خود را تقدیم استاد گرانقدرم **سرکار خانم دکتر شهناز پولادی** می‌نمایم که با صبر و شکیبایی و راهنمایی‌های عالمانه‌ی خویش، مرا در تهیه و تدوین این رساله یاری کردند و به خود می‌بالم که سعادت شاگردی در محضر پرفیضشان را داشتم.

همچنین بر من است که از دیگر استادان بزرگوارم **جناب آقای شریف شریفی** و **سرکار خانم دکتر نیلوفر معتمد** که مسئولیت مشاوره این پایان‌نامه را بر عهده داشتند و در راستای انجام این طرح تجربیات ارزشمندشان را صادقانه و سخاوتمندانه در اختیار من گذاشتند صمیمانه تشکر و سپاسگزاری نمایم.

سپاس بی دریغ از **آقای دکتر یزدانخواه فرد** و **خانم دکتر زنده بودی**، که صبورانه زحمت داوری این پایان‌نامه را تقبل نمودند.

سپاس بی پایان از کارشناسان تحصیلات تکمیلی دانشگاه و مرکز توسعه پژوهش‌های بالینی بیمارستان شهدای خلیج فارس بوشهر.

و کلام آخر این که در مقابل این همه عظمت و شکوه، مرا نه توان سپاس است و نه کلام وصف.

تقدیم به

پدرم

کوهی استوار و حامی من در تمام طول زندگی

مادرم

سنگ صبورم که الفبای زندگی به من آموخت

فهرست مطالب

فصل اول: معرفی پژوهش

۱. کلیات پژوهش ۲

۱-۱. بیان مسئله ۲

۱-۲. اهداف پژوهش ۱۴

۱-۲-۱. هدف کلی ۱۴

۱-۲-۲. اهداف ویژه (اختصاصی) ۱۵

۱-۲-۳. هدف کاربردی ۱۶

۱-۳. سؤالات پژوهش ۱۶

۱-۴. فرضیات پژوهش ۱۶

فصل دوم: چارچوب پنداشتی و مروری بر متون

۲. مقدمه ۲۱

۲-۱. چارچوب پنداشتی ۲۱

۲-۱-۱. تئوری رفتار برنامه‌ریزی شده ۲۱

۲-۱-۲. کلیه ۲۲

۲-۱-۲-۱. عملکرد کلیه ۲۲

۲-۱-۲-۲. فیزیولوژی کلیه ۲۴

۲-۱-۲-۳. نارسایی کلیه ۲۵

۲-۱-۲-۴. انواع نارسایی کلیه ۲۵

۲-۱-۲-۴-۱. نارسایی حاد کلیه ۲۶

۲-۱-۲-۴-۲. بیماری مزمن کلیوی ۲۶

۲-۱-۲-۶. دیالیزر ۳۲

۲-۱-۲-۷. عوارض دیالیز ۳۵
۲-۱-۲-۸. کفایت دیالیز ۳۸
۲-۱-۲-۸-۱. روش‌های تعیین کفایت دیالیز ۳۹
۲-۱-۲-۸-۲. عوامل تأثیرگذار بر کفایت دیالیز ۴۱
۲-۱-۳. فعالیت فیزیکی ۴۳
۲-۱-۳-۱. تعریف فعالیت فیزیکی ۴۳
۲-۱-۳-۲. فواید فعالیت بدنی و وضعیت فعالیت بدنی در دنیا ۴۴
۲-۱-۳-۳. ورزش و فعالیت فیزیکی در بیماران تحت دیالیز ۴۵
۲-۱-۴. خستگی ۴۶
۲-۱-۴-۱. تعریف خستگی ۴۶
۲-۱-۴-۲. انواع خستگی ۴۷
۲-۱-۴-۳. خستگی در بیماران همودیالیز ۴۸
۲-۲. مروری بر متون ۴۹
۲-۲-۱. مطالعات داخلی ۴۹
۲-۲-۲. مطالعات خارجی ۵۳
۲-۳. جمع‌بندی ۵۷

فصل سوم: روش شناسی پژوهش

۳. روش‌شناسی پژوهش ۶۰
۳-۱. مواد و روش پژوهش ۶۰
۳-۱-۱. نوع پژوهش ۶۰
۳-۱-۲. جامعه و واحد پژوهش ۶۰
۳-۱-۳. محیط پژوهش ۶۰

۳-۱-۴. معیارهای ورود به مطالعه ۶۰

۳-۱-۵. معیارهای خروج از مطالعه ۶۱

۳-۱-۷. روش نمونه‌گیری ۶۲

۳-۱-۸. ابزار گردآوری داده‌ها ۶۲

۳-۱-۹. روش پژوهش ۶۴

۳-۱-۱۱. روش تجزیه‌وتحلیل داده‌ها ۶۷

۳-۲. ملاحظات اخلاقی ۶۷

فصل چهارم: یافته های پژوهش

۴. یافته‌های پژوهش ۷۰

فصل پنجم: بحث و نتیجه گیری

۵-۱ بحث و بررسی یافته‌ها و پیشنهادات ۸۸

۵-۲. نتیجه گیری نهایی ۹۶

۵-۳. کاربرد نتایج ۹۷

۵-۳-۱. کاربرد نتایج در بالین ۹۷

۵-۳-۲. کاربرد نتایج در مدیریت ۹۷

۵-۳-۳. کاربرد نتایج در آموزش ۹۷

۵-۴. محدودیت‌های مطالعه ۹۸

۵-۵. پیشنهادات پژوهشی برای مطالعات آینده ۹۸

منابع و مآخذ

فهرست منابع ۱۰۱

پیوست‌ها

پیوست ۱. فرم اطلاعات نمونه ۱۱۸

پیوست ۲. پرسشنامه شدت خستگی ۱۲۲
پیوست ۳. مصوبه اخلاق ۱۲۳
پیوست۲. پرسشنامه شدت خستگی ۱۲۲
پیوست۳. مصوبه اخلاق ۱۲۳

فهرست جداول

جدول ۴-۱. مقایسه متغیرهای جمعیت شناختی بیماران بین گروه آزمون و کنترل ۷۲
جدول ۴-۲. مقایسه میزان اوره خون و وزن در بیماران قبل و بعد از مداخله بین دو گروه آزمون و کنترل در بیمارستان‌های شهر بوشهر در سال ۱۳۹۹ ...... ۷۳
جدول ۴-۳. مقایسه میانگین تغییرات اوره خون و وزن در بیماران در طول مداخله بین دو گروه آزمون و کنترل در بیمارستان‌های شهر بوشهر در سال ۱۳۹۹ ...... ۷۴
جدول ۴-۴. مقایسه میانگین Kt/V در بیماران بعد از مداخله بین دو گروه آزمون و کنترل در بیمارستان‌های شهر بوشهر در سال ۱۳۹۹ ...... ۷۵
جدول ۴-۵. مقایسه شاخص‌های همودینامیک درصد اشباع اکسیژن خون شریانی در بیماران قبل و بعد از مداخله در هر دو گروه آزمون و کنترل به تفکیک ۷۶
جدول ۴-۶. مقایسه شاخص همودینامیک درجه حرارت در بیماران قبل و بعد از مداخله در هر دو گروه آزمون و کنترل به تفکیک ۷۷
جدول ۴-۷. مقایسه شاخص همودینامیک RR در بیماران قبل و بعد از مداخله در هر دو گروه آزمون و کنترل به تفکیک ۷۸
جدول ۴-۸. مقایسه شاخص همودینامیک PR در بیماران قبل و بعد از مداخله در هر دو گروه آزمون و کنترل به تفکیک ۷۹
جدول ۴-۹. مقایسه شاخص همودینامیک فشارخون سیستول در بیماران قبل و بعد از مداخله در هر دو گروه آزمون و کنترل به تفکیک ۸۰
جدول ۴-۱۰. مقایسه شاخص همودینامیک فشارخون دیاستول در بیماران قبل و بعد از مداخله در هر دو گروه آزمون و کنترل به تفکیک ۸۱

جدول ۴-۱۱. مقایسه شاخص همودینامیک فشارخون متوسط شریانی در بیماران قبل و بعد از مداخله در هر دو گروه آزمون و کنترل به تفکیک ۸۲

جدول ۴-۱۲. مقایسه میانگین تغییرات شاخص‌های همودینامیک (درجه حرارت، نبض، فشارخون، تنفس و درصد اشباع اکسیژن خون شریانی) در بیماران در طول مداخله بین گروه آزمون و کنترل.................. ۸۳

جدول ۴-۱۳. مقایسه خستگی در بیماران قبل و بعد از مداخله در هر دو گروه آزمون و کنترل به تفکیک ۸۴

جدول ۴-۱۴. مقایسه میانگین تغییرات خستگی در بیماران، در طول مداخله بین گروه در بیماران تحت همودیالیز در بیمارستان‌های شهر بوشهر در سال ۱۳۹۹ .... ۸۵

جدول ۴-۱۵. تعیین و مقایسه سطح خستگی در بیماران قبل و بعد از مداخله بین دو گروه آزمون و کنترل در بیمارستان‌های شهر بوشهر در سال ۱۳۹۹ .... ۸۶

فهرست اشکال

شکل ۲-۱: مراحل بیماری مزمن کلیه ۲۸

شکل ۲-۲. ماشین‌های دیالیز ۳۴

شکل ۲-۳. فیستول شریانی وریدی ۳۴

شکل ۳-۱. نمودار کنسورت، مراحل اجرای تحقیق ۶۶

فصل اول

مقدمه و کلیات پژوهش

۱. کلیات پژوهش

۱-۱. بیان مسئله

بیماری‌های مزمن همراه با عوارض و مشکلاتی مانند بیماری‌های قلبی عروقی، پرفشاری خون، دیابت، زخم‌های گوارشی، چربی خون بالا، آسم، آرتروز و بیماری‌های مزمن کلیوی همراه می‌باشد. در آخرین بررسی و مطالعه جهانی ۲۰۱۶[1] که توسط انستیتوی سنجش و ارزشیابی سلامت[2]، در سیاتل[3] ایالات‌متحده و با مشارکت بیش از ۱۳۰ کشور انجام گردید؛ بیان شد که ۷۲ درصد از مرگ‌ومیرهای جهان در سال ۲۰۱۶، ناشی از بیماری‌های مزمن غیر واگیر بوده است. این میزان از مرگ‌ومیر در ایران در سال ۲۰۱۶، هشتاد درصد می‌باشد(۱). بیماری‌های مزمن کلیوی یکی از مهم‌ترین و خطرناک‌ترین بیمارهای مزمن در سطح جهان است، که باعث اثرات کوتاه‌مدت و طولانی‌مدت و کاهش کیفیت زندگی بیماران می‌گردد(۲, ۳). مطالعات اخیر نشان می‌دهد که در ایالات‌متحده بین۱۱/۵ تا ۱۴/۵ درصد از بالغین در مراحل ابتدایی تا پیشرفته نارسایی مزمن کلیوی قرار دارند(۴). برخلاف تمام تلاش‌ها جهت پیشگیری و درمان نارسایی مزمن کلیوی، تعداد موارد ابتلا به نارسایی مزمن کلیوی در حال افزایش است. شیوع نارسایی مزمن کلیوی در مناطق مختلف جهان متفاوت است، به‌طوری‌که در ایران ۶/۵ تا ۲۳/۷ درصد و در مکزیک تا ۳۳ درصد برآورد شده است(۵, ۶). نارسایی مزمن کلیوی باعث بروز عوارضی مانند آنمی، بیماری‌های قلبی عروقی، بیماری‌های استخوانی، بیماری‌های عفونی، افزایش مرگ‌ومیر، خستگی، اختلالات جنسی،

---

[1] Global Burden Of Diseases Study 2016
[2] Institute Of Health Metrics And Evaluation
[3] Seattle

افسردگی، کاهش سلامت روانی و درنهایت کاهش کیفیت زندگی می‌گردد(۷, ۸). از آن رو که این بیماری افراد را به سمت بروز بیماری‌های ایسکیمیک قلبی و پرفشاری خون سوق می‌دهد(که خود جزء سه علت اصلی مرگ‌ومیر هستند)، اهمیت و ارزش بالاتری در مدیریت این بیماری مزمن به چشم می‌آید(۹). از طرفی طبق گزارش فوق‌الذکر، دو عامل از علل اصلی بروز نارسایی مزمن کلیوی، یعنی پرفشاری خون و دیابت شیرین به ترتیب از رتبه دهم به ششم و از رتبه دوازدهم به هفتم در علل مرگ‌ومیر طی سال ۲۰۱۶، رسیده‌اند(۹) که باعث افزایش بروز نارسایی مزمن کلیوی به عنوان یک عامل اصلی مرگ‌ومیر می‌شوند(۱).

درمان‌های مختلفی جهت جایگزینی نارسایی مزمن کلیوی[1] وجود دارند. این درمان‌ها شامل پیوند کلیوی، دیالیز صفاقی و همچنین دیالیز خونی (همودیالیز) می‌باشد(۱۰). در پایان سال ۲۰۱۶ حدود ۳۷۳۰۰۰۰ نفر در جهان با این بیماری برآورد شدند که تعداد ۲۶۴۸۰۰۰ نفر تحت درمان با همودیالیز و تنها ۳۴۱۰۰۰ نفر تحت درمان با دیالیز صفاقی قرار گرفتند. بیماران پیوندی نیز حدود ۷۴۱۰۰۰ نفر بودند. پیش‌بینی رشد جهانی بیماران دیالیزی تا سال ۲۰۲۲ نشان می‌دهد که جمعیت این بیماران به حدود ۴ میلیون نفر خواهد رسید. تا پایان سال ۱۳۹۵ جمعیت بیماران با نارسایی مزمن کلیوی در ایران به حدود ۵۸ هزار نفر رسید، که از ۳۱۰۰۰ نفر تعداد بیمار دیالیزی حدود ۲۹۲۰۰ نفر یعنی ۹۵ درصد از آن‌ها تحت همودیالیز قرار دارند. رشد سالانه بیماران همودیالیزی در جهان به‌طور متوسط ۶-۵ درصد از سال ۲۰۱۵ تا ۲۰۱۶ بوده است که این نرخ در ایران حدود ۷ درصد یعنی بالاتر از رشد جهانی می‌باشد(۱۱).

هرچند مزایای پیوند کلیه ازلحاظ افزایش کیفیت زندگی و کاهش افسردگی و اضطراب و افزایش عزت‌نفس، در برابر همودیالیز، مورد تائید است(۱۲, ۱۳)، ولی دریافت‌کنندگان پیوند کلیه یک نگاه منفی به لحاظ رژیم سخت داروهای سرکوب‌کننده ایمنی، عوارض آن‌ها، نیاز به ویزیت‌های مکرر، عفونت‌ها و اضطراب و نگرانی در مورد رد پیوند و از دست دادن عضو پیوندی دارند(۱۴). از دیگر درمان‌های جایگزین نارسایی مزمن کلیه، دیالیز صفاقی می‌باشد. دیالیز صفاقی باعث کاهش هزینه‌های درمان، حفظ باقیمانده عملکرد کلیه‌ها، کاهش خطر مرگ‌ومیر در افراد تحت درمان، نداشتن محدودیت‌های دیالیز در مرکز،

[1] Renal Replacement Therapy

می‌باشد(۱۵). هرچند در برخی مطالعات عوارض یکسانی برای آن در برابر همودیالیز به‌دست آمده است(۱۶)، ولی دیالیز صفاقی می‌تواند با عوارضی مانند پریتونیت، آسیت، عفونت‌های قارچی، عوارض مربوط به کاتتر شکمی، پارگی روده‌ها و خونریزی همراه باشد(۱۷). برخلاف این‌که بهترین درمان جایگزین در نارسایی مزمن کلیوی، پیوند کلیه می‌باشد(۱۸)تا پایان سال ۲۰۱۶ همچنان همودیالیز رایج‌ترین روش درمانی در جهان بود که حدود ۷۰ درصد از بیماران نارسایی مزمن کلیوی از آن استفاده می‌کنند(۱۹). انجام همودیالیز در بیمارانی که آمادگی جسمی و روحی مناسب دارند باعث کاهش میزان بستری شدن و کاهش در هزینه‌ها می‌گردد. از طرفی انجام دیالیز باعث کنترل و کاهش عوارضی مانند افزایش فسفات خون، فشارخون بالا، آنمی و بهبود تغذیه می‌شود. جهت انجام همودیالیز برنامه‌های مختلف و متنوعی مانند همودیالیز در خانه[۱](۶-۳ بار در هفته)، همودیالیز در مرکز[۲]، همودیالیز کوتاه‌مدت روزانه[۳] و همودیالیز بلندمدت شبانه[۴](۷-۹ساعت) پیشنهاد می‌گردد که معمول‌ترین روش انجام آن در حال حاضر، همودیالیز در مرکز به مدت سه جلسه در هفته و هر جلسه ۳-۵ ساعت می‌باشد[۵](۲۰, ۲۱). بیماران مزمن کلیوی تحت درمان با همودیالیز معمولاً سه جلسه در هفته و هر بار ۳ تا ۴ ساعت یعنی حدود ۹ تا ۱۲ ساعت از وقت خود را جهت انجام همودیالیز می‌گذرانند(۲۱). از دهه ۱۸۵۰ میلادی که توماس گراهام[۶](استاد شیمی دانشگاه گلاسکوی اسکاتلند)، قانون انتشار انتخابی یا جدا شدن مواد از عرض غشاء نیمه‌تراوا را کشف کرد تاکنون، همودیالیز پیشرفت‌های زیاد و چشم‌گیری نموده است. اولین تلاش برای انجام دیالیز برای یک بیمار اورمیک در سال ۱۹۲۴ توسط دکتر جورج هاس[۷] انجام شد، تا اینکه در سال ۱۹۴۳ اولین صافی مناسب جهت همودیالیز انسان توسط دکتر ویلم کیلف[۸] ساخته شد. تاکنون تلاش‌های بی‌شماری جهت جایگزینی مناسب دیالیز انجام شده است ولی در حال حاضر بیشترین راه جایگزین نارسایی مزمن کلیوی دیالیز و از جمله همودیالیز می‌باشد(۲۲).

---

[1] Home Long Hemodialysis
[2] In-Center Hemodialysis
[3] Short Frequent Hemodialysis
[4] Home Long Hemodialysis Nighttime
[5] Conventional Hemodialysis
[6] Thomas Graham
[7] Georg Haas
[8] Dr.Willem J. Kplff

هدف از انجام همودیالیز، برداشت مواد دفعی و مایعات اضافی از بدن و بهبود محیط داخلی بدن جهت ادامه حیات و افزایش کیفیت زندگی می‌باشد(۲۳). همودیالیز از تجمع مواد و سموم دفعی، آب و دیگر مواد ناخالص در بدن جلوگیری می‌کند. همودیالیز به کنترل فشارخون کمک کرده و در تنظیم سطح الکترولیت‌های بدن نقش اساسی دارد. ازجمله این الکترولیت‌ها سدیم، پتاسیم و فسفات می‌باشند. مسلماً بدون انجام دیالیز این مواد تجمع یافته و باعث عوارض اساسی بر سیستم‌های قلبی عروقی، عضلانی اسکلتی، نورولوژیک، گوارش و سیستم بافری و آب و الکترولیت و ... می‌گردد(۲۲). هرچند همودیالیز باعث پیشگیری از مرگ ناشی از اورمی می‌گردد؛ با این وجود میزان بقاء بیماران نارسایی مزمن کلیوی بسیار کمتر از جمعیت عمومی می‌باشد(۲۴). فاکتورهای متعددی در میزان بقاء این بیماران تأثیر دارد؛ که از جمله می‌توان به علت نارسایی کلیوی، روش درمان جایگزین، وجود بیماری‌های دیگر مانند بیماری‌های قلبی عروقی، دیابت و پرفشاری خون و همچنین میزان کفایت دیالیز اشاره کرد(۲۴). پیش‌آگهی طولانی‌مدت بیماران دیالیزی کاملاً به کیفیت دیالیز آن‌ها وابسته می‌باشد. کفایت دیالیز همچنین شاخصی برای میزان مرگ‌ومیر و از پیامدهای اولیه دیالیز می‌باشد. بیمارانی که از کفایت دیالیز بهتری برخوردار باشند، زندگی طولانی‌تری خواهند داشت و بنابراین از شانس بیشتری جهت استفاده از درمان جایگزین پیوند کلیه برخوردار خواهند بود(۲۵).

کفایت نامطلوب دیالیز موجب افزایش عوارض نارسایی کلیوی، مدت زمان بستری شدن و هزینه‌های درمانی می‌گردد. افزایش کفایت دیالیز باعث بالا رفتن کیفیت زندگی و سلامت جسمی و معنوی بیماران می‌گردد(۲۶). از جمله اختلالات جسمی که ارتباط مستقیمی با کفایت دیالیز دارد، اختلالات عملکرد جنسی(۲۷)؛ که با بالا رفتن کفایت دیالیز بروز آن کاهش می‌یابد؛ بیماری‌های قلبی عروقی(۲۱) و همچنین عارضه‌ای شایع مانند خارش پوست که در بیماران با کفایت بالاتر کمتر دیده می‌شود(۲۸). از این‌رو یکی از راه‌های کاهش میزان مرگ‌ومیر، کاهش مدت زمان بستری شدن، کاهش عوارض بیماری کلیوی، افزایش امید به زندگی، افزایش کیفیت زندگی و کاهش هزینه‌های درمانی، بالا بردن کفایت دیالیز در بیماران همودیالیزی می‌باشد(۲۹). هدف نهایی در بیماران با نارسایی مزمن کلیوی در مرحله ۵ بالا بردن کیفیت

زندگی همراه با زندگی طولانی به عنوان هدفی دیگر می‌باشد. از این رو گاهی کفایت دیالیز با مدیریت کیفیت سایر جنبه‌های بیمار اشتباه گرفته می‌شود. این نکته بسیار اهمیت دارد که کفایت دیالیز از کفایت مراقبت از بیمار تمیز داده شود. این مراقبت‌ها؛ که می‌تواند مستقل از دیالیز و یا مربوط به آن باشد؛ شامل مدیریت کم‌خونی، تغذیه، بیمارهای متابولیک استخوان، دیابت و بیماری قلبی عروقی باشد(۲۱). پاکسازی اوره[1] در یک فرد سالم حدوداً ۱۳۰سی‌سی در دقیقه است. کلیرانس اوره در ۲۴ ساعت در فرد سالم حدود ۲۰۰ لیتر می‌باشد. در نتیجه برای بررسی کفایت کلیه یک فرد سالم در شبانه‌روز ۵ لیتر یعنی در هر روز ۵ بار کل مایعات بدن این فرد به‌طور کامل از اوره توسط کلیه‌ها پاک می‌شود(این ۵ لیتر ۴۰ بار در بدن از اوره پاک می‌شود) و برای بررسی کفایت هفتگی یک فرد سالم به عدد ۳۵ می‌رسیم، اما در فردی با نارسایی مزمن کلیوی که هفته‌ای سه بار دیالیز می‌شود حداقل کفایت تعیین شده برای یک جلسه برابر با ۱/۲و در هفته برابر با ۶/ ۳ می‌باشد. این بدین معناست که کفایت دیالیز هفتگی با سه جلسه دیالیز برابر با یک‌دهم کفایت کلیه یک فرد سالم است درنتیجه دیالیز فقط ۱۰تا۱۵درصد کار کلیه سالم را(فقط از نظر کلیرانس اوره) انجام می‌دهد و البته که بقیه کارهای کلیه را اصلاً انجام نمی‌دهد. بنابراین افزایش و حفظ این کفایت جهت بهبود هر چه بهتر کیفیت زندگی بیمار ضروری است(۲۰, ۳۰). موسسه ملی کیفیت پیامدهای دیالیز[2]پاکسازی اوره را به عنوان کفایت دیالیز تعریف می‌کند(۳۰, ۳۱). دیالیز بهینه(کافی) می‌تواند به عنوان درمان دیالیزی تعریف شود که باعث می‌شود بیماران به احساسی همانند زمانی‌که نارسایی کلیوی نداشته‌اند، دست یابند. مقدار دیالیز که در طول یک جلسه دیالیز انجام می‌شود، با Kt/V[3] و نسبت کاهش اوره[4] محاسبه می‌شود. انجام دیالیز با بهترین کیفیت، باعث پیامد بهتر خواهد بود. Kt/V اوره اندازه‌گیری اثربخشی درمان دیالیز در از بین بردن مواد زائد، به‌ویژه نسبت پاکسازی اوره و زمان در دیالیز بر روی حجم توزیع اوره (کل بدن آب) می‌باشد(۲۲). جهت پاکسازی اوره طبق الگوی کینیتیک اوره[4] از شاخص Kt/V و همچنین نسبت کاهش اوره استفاده می‌گردد، و حداقل شاخص Kt/V ۱/۲ و نسبت کاهش اوره ۶۵٪

---

[1] Urea Clearance

[2] Foundation Dialysis Outcomes Quality Initiative (NKF KDOQI)

3 K : Dialyzer Clearance Of Urea T : Dialysis Time V : Volume Of Distribution Of Urea, Approximately Equal To Patient's Total Body Water

[4] Urea Reduction Ratio

برآورد شده است. Kt/V نشان‌دهنده کسری از حجم آب بدن است که طی یک جلسه دیالیز از اوره پاک می‌شود. K ضریب پاکسازی صافی به میلیمتر در دقیقه، t زمان دیالیز به دقیقه از شروع تا پایان دیالیز و V حجم توزیع اوره به میلیمتر در بدن می‌باشد (۳۰, ۳۲).

بیماران با نارسایی مزمن کلیوی تحت همودیالیز، با کاهش عملکردی و کاهش کیفیت زندگی همراه هستند(۳۳, ۳۴). این عملکرد ضعیف فیزیکی با خطر بالای مرگ‌ومیر و بستری شدن، اختلالات متابولیسم استخوان، تغییرات کاتابولیسم پروتئین، سوء تغذیه و التهاب مزمن، نوروپاتی و میوپاتی اورمیک همراه می‌باشد. علاوه بر این برخی اختلالات روانی مانند افسردگی و اضطراب مربوط به درمان آن‌ها، در بیماران همودیالیزی بیشتر است (۳۵, ۳۶). همچنین، همودیالیز در یک وضعیت افقی انجام می‌شود و فرد تقریباً ۸۰۰ ساعت در هر سال را بدون فعالیت جسمانی می‌گذراند، بنابراین عوارضی همچون کاهش استقامت جسمی بدن، آتروفی عضلانی، ضعف عضلانی و کاهش قدرت را به دنبال خواهد داشت(۳۷). بنابراین، انجام تمرین ورزشی منظم در حین دیالیز در بیماران همودیالیزی باعث افزایش عملکرد جسمانی و کاهش آتروفی عضلانی می‌شود(۳۸). فعالیت باعث تأثیر مثبت بر عملکرد فیزیکی، افسردگی و کیفیت زندگی می‌گردد. البته همواره یک سری موانع جهت انجام فعالیت در همودیالیز وجود دارد. از دید بیماران، کمبود دانش مربوط به فعالیت، ترس از آسیب، کمبود ظرفیت و توانایی انجام فعالیت و کمبود انگیزه و تجربه می‌باشند. از دیدگاه دیالیزکاران، کمبود افراد حرفه‌ای جهت نظارت بر برنامه‌های فعالیت، کمبود منابع مالی، و درگیری محدود مراقبین بهداشتی می‌باشد. پرستاران با توجه به درگیری مداوم و نزدیکی بیشتر نسبت به سایر مراقبین بهداشتی با بیماران و خانواده آن‌ها در موقعیت مناسبی جهت کمک به بیماران برای انجام فعالیت و برنامه‌های ورزشی قرار دارند(۳۹). فعالیت و بازتوانی جهت پیشگیری از کاهش قدرت عضلانی و کاهش عملکرد بیمار مبتلا به نارسایی مزمن کلیوی تحت دیالیز بسیار مهم می‌باشند. فعالیت باعث افزایش ظرفیت هوازی، عملکرد عضلانی و وضعیت کیفیت زندگی می‌شود. از طرفی فعالیت فیزیکی خطر عود بیماری و مرگ‌ومیر ناشی از آن‌ها مانند سرطان، بیماری‌های قلبی عروقی و بیماری‌های انسدادی ریه را کاهش می‌دهد(۴۰).

فعالیت حین دیالیز می‌تواند، باعث کاهش فاکتورهای التهابی شامل پروتئین واکنشی سی[1]، فاکتور نکروزی توموری آلفا[2]، مونوسیت‌ها و اینترلوکین گردد. این مساله می‌تواند بیمار نارسایی مزمن کلیوی تحت همودیالیز را تا حدودی از خطر بیماری‌های قلبی عروقی و مرگ‌ومیر ناشی از آن همراه با التهاب مزمن و افزایش مونوسیت‌ها در امان نگه دارد(۴۱). از دیگر مزایای مهم فعالیت حین دیالیز[3]، افزایش میزان عبور مواد محلول در خون و اثربخشی بهتر دیالیز است. در مقایسه با همودیالیز در حال استراحت، جریان خون و پرفیوژن عضلات در حال ورزش افزایش یافته، بنابراین میزان مبادله بین خون و عضلات افزایش می‌یابد. به دنبال این افزایش تبادل، مواد زائد بیشتری مانند اوره می‌توانند از عضلات وارد گردش خون شوند و از طریق دیالیز دفع شوند و در طول انجام فعالیت حین دیالیز از گردش خون حذف گردند(۴۲, ۴۳).

طبق آمار سالانه سیستم اطلاع‌رسانی مرکز کلیوی ایالات‌متحده، میزان مرگ‌ومیر بیماران دیالیزی که سه بار در هفته دیالیز می‌شوند، به‌طور غیرقابل قبولی بالا می‌باشد؛ و در مقایسه با سایر انواع دیالیزهای مکرر[4] مانند دیالیز روزانه و دیالیز شبانه این آمار بالاتر است. انجام دیالیز ۶ بار در هفته می‌تواند پیامدهای بسیار مثبتی برای بیمار نارسایی مزمن کلیوی تحت درمان همودیالیز داشته باشد(۴۴). این نوع همودیالیز به امکانات، شرایط فیزیکی، مالی و بیمه‌ای خاصی نیاز دارد، که محدودیت‌هایی در انجام آن ایجاد می‌کند. علیرغم تمامی مزایای FHN، پیامدهایی نیز دارد. هر جلسه دیالیز به‌طور متوسط حدود ۴۵ دقیقه جهت تردد بیمار و خانواده در مسیر خانه تا مرکز دیالیز، زمان نیاز دارد که باعث افزایش زمان همودیالیز تا حدود ۱۱ تا ۱۴ ساعت بیشتر در هفته می‌شود(۱۰). همچنین دیالیز مکرر نیاز به دسترسی عروقی مناسب دارد که این نوع همودیالیز باعث افزایش عوارض مربوط به دسترسی عروقی شد(۴۵). دیالیز مکرر روزانه باعث بالا رفتن سرعت از دست رفتن عملکرد باقیمانده کلیوی[5] در بیماران جدید، بالا رفتن خطر عفونت در بیماران، افزایش کار و خستگی بیمار و همراهان می‌شود(۴۶).

[1] C-Reactive Protein
[2] Tomoral Necrosis Factor_α
[3] Intra Dialytic Exercise
[4] Frequent Hemodialysis Network(FHN)
[5] Residual Kidney Function

مدت زمان همودیالیز و Kt/V هر دو می‌توانند به عنوان اندازه‌گیری اولیه کفایت دیالیز، مهم باشند چرا که در صورت صفر شدن هرکدام از آن‌ها در بیماری که دارای عملکرد باقیمانده کلیه نمی‌باشد، باعث مرگ گردد. شواهد با کیفیت کمی جهت اثبات نیاز به حداقل ۴ ساعت درمان در همه بیماران، بدون توجه به‌اندازه بدن، میزان برداشت مواد و عملکرد باقیمانده کلیوی، وجود دارد. از جهتی شواهدی وجود ندارد که افزایش زمان تا ۲۴ ساعت در هفته دیالیز می‌تواند مضر باشد. هرچند تصمیم نهایی در مورد زمان همودیالیز بهتر است بر اساس پذیرش و تجربه بیمار، عملکرد باقیمانده کلیه، سطح بدن، میزان Kt/V و میزان اولترافیلتراسیون باشد(۴۷)، درمان همودیالیز سنتی به‌طورمعمول برای بیماران با نارسایی مزمن کلیوی در سطح جهان ۳ تا ۵ ساعت، ۳ بار در هفته انجام می‌شود(۲۱). در ایالات‌متحده به بیماران با جثه کوچک و زنان که دیالیز با کیفیت دارند(مثلاً Kt/V>1/2)، اجازه همودیالیز در جلسات کوتاه‌تر مثلاً ۳/۵-۲/۵ ساعت داده می‌شود، که در تضاد با قانون حداقل ۴ ساعت می‌باشد. به طور کلی زمان و Kt/V دو عامل از هم جداناشدنی و زمان یک عامل جبری در برآورد Kt/V می‌باشد. به طور کلی در نظر گرفتن Kt/V به‌تنهایی کافی نمی‌باشد، خصوصاً در زنان و افراد با جثه کوچک و باید علاوه بر آن میزان عملکرد باقیمانده کلیه و میزان اولترافیلتراسیون[1] را در نظر گرفت(۴۷).

علت اصلی ابتلا به نارسایی کلیوی پرفشاری خون می‌باشد. از این رو بیماران نارسایی مزمن کلیوی همواره با تغییرات و بی‌ثباتی در وضعیت همودینامیک مواجه هستند(۴۸). درواقع پرفشاری خون می‌تواند منجر به نارسایی کلیوی شده و از طرفی نارسایی کلیوی باعث پرفشاری خون گردد و بررسی و مونیتورینگ این ایندکس، مهم جلوه می‌کند(۴۹). فشارخون اغلب به میزان حجم مربوط است. پرفشاری خون می‌تواند نشانه‌ای از اضافه بار مایع باشد و فشارخون پایین می‌تواند بیانگر کم آبی[2] بیمار باشد. شایع‌ترین عارضه حین دیالیز افت فشارخون[3]، در ارتباط با کاهش سریع حجم خون در گردش به واسطه اولترافیلتراسیون است. همچنین نبودن انقباض عروقی مناسب ناشی از داروهای ضد فشارخون یا سایر عوامل قلبی از دیگر

[1] Ultrafiltration
[2] Dehydration
[3] Hypotention

علل افت فشارخون است. افت فشارخون با شروع همودیالیز در برخی از بیماران که حجم خون کمی دارند رخ می‌دهد. این مساله ناشی از جابجایی حجم، همزمان با پر شدن صافی از خون بیمار است. افت فشارخون تأخیری معمولاً به برداشت مایع از فضای عروقی (اولترافیلتراسیون) بیش از توانایی بیمار برای جبران آن، نسبت داده می‌شود. هیپوتانسیون ممکن است تا زمانی که فشارخون سیستولی ۴۰ تا ۵۵ میلی‌متر جیوه کاهش یابد، بدون علامت باشد. تعداد کمی از بیماران دچار افزایش فشارخون حین دیالیز می‌شوند. در برخی بیماران افزایش فشارخون نتیجه برون ده قلبی افزایش یافته ناشی از اضافه بار مایع است. در سایر موارد ممکن است افزایش مقاومت عروق محیطی در زمینه سیستم عروقی یا واکنشی اتفاق می‌افتد(۲۲). انجام فعالیت به صورت یک دوره کوتاه‌مدت می‌تواند باعث یک کاهش گذرا در فشارخون سیستولیک به میزان ۱۵-۵ میلی‌متر جیوه در یک فرد با فشارخون بالا شود که این کاهش فشارخون می‌تواند تا ۲۲ ساعت به طول بیانجامد. این نظریه بیمار را مستعد افت فشارخون به دنبال فعالیت می‌کند. از طرفی در فعالیت طولانی‌مدت این افت فشارخون محسوس نبوده و علائمی ایجاد نمی‌کند و فعالیت طولانی‌مدت می‌تواند باعث کاهش فشارخون در بیمار پرفشاری خون هم در سیستولیک و هم در دیاستول در زمان استراحت گردد(۵۰).

هرچند سودمندی اثر فعالیت در طی همودیالیز روی مدیریت و کنترل فشارخون دیده شده است(۵۱, ۵۲)، اما کنترل آن حین همودیالیز ضروری به نظر می‌رسد. به علت کاهش حجم خون به دنبال فعالیت احتمال هیپوتانسیون در فعالیت متوسط تا شدید حین همودیالیز محتمل است. هرچند افزایش ضربان قلب به دنبال فعالیت می‌تواند این افت فشار را تا حدودی جبران کند(۵۳, ۵۴). در شروع دیالیز اندازه‌گیری درجه حرارت، نبض و تنفس میزان پایه ثبت می‌شود. افزایش درجه حرارت بیانگر عفونت یا عوارض بیماری است. همچنین درجه حرارت بالا می‌تواند نشانه‌ای از عفونت دسترسی عروقی، درجه حرارت بالای محلول دیالیز، واکنش به عامل عفونی و واکنش آنافیلاکتیک باشد. تاکیکاردی ممکن است ناشی از کم‌خونی یا افزایش حجم مایع باشد. آریتمی ممکن است نشان‌دهنده عوارض قلبی از جمله اختلالات مربوط به الکترولیت‌ها باشد. تاکیکاردی می‌تواند با کاهش حجم خون (ناشی از

اولترافیلتراسیون) همراه باشد و درست پیش از هیپوتانسیون رخ دهد. از طرفی تاکی‌پنه می‌تواند نشانه‌ای از افزایش مایع در بدن باشد. بنابراین پایش مداوم بیمار و ماشین دیالیز دست‌کم هر نیم ساعت به‌وسیله پرستار، ضروری است(۲۲). اگرچه عملکرد کلیه‌ها تا حدودی از طریق دیالیز جایگزین می‌شود، بیماران با نارسایی مزمن کلیوی علائم بسیاری مانند تهوع، استفراغ، اختلال خواب، خارش و خستگی را تجربه می‌کنند. خستگی یکی از علائم معمول در بیماران با درمان جایگزین همودیالیز است که آن را تجربه می‌کنند. خستگی از میزان بروز بالایی در حدود ۹۷-۶۰٪ برخوردار است(۵۵-۵۸). خستگی یک پدیده پیچیده است که شامل جنبه‌های بسیاری از وجود مسائل جسمی و روحی و عاطفی تعیین می‌شود. خستگی همچنین می‌تواند به عنوان یک وضعیت که فرد دچار ناراحتی و کاهش توانایی عملکرد به دلیل کمبود انرژی می‌شود، توصیف شود(۵۶). بیشترین نوع خستگی که پس از همودیالیز دیده می‌شود، خستگی جسمی و خستگی عمومی بدن و کاهش فعالیت‌ها می‌باشد. افرادی که دارای بیماری مزمن کلیوی هستند، سطح بالایی از خستگی را بدون در نظر گرفتن سن، جنس، وضعیت سلامتی و مدت همودیالیز تجربه می‌کنند و شناخت سطح خستگی در این بیماران، اثرات آن بر زندگی روزمره آن‌ها و کاهش آن جهت بهبود شرایط زندگی لازم است(۵۹). از طرفی خستگی با عواملی مانند افسردگی، اضطراب، مرگ‌ومیر، بیماری‌های مزمن همراه و سطح اینترلوکین و سایر فاکتورهای ضدالتهابی مانند پروتئین واکنشی سی در بیماران نارسایی مزمن کلیوی در ارتباط است و به عنوان یک عامل مستقل نه‌تنها به صورت روتین بررسی‌شده بلکه با هدف کاهش مرگ‌ومیر در بیماران تحت همودیالیز نیاز به مداخله دارد(۶۰-۶۲). خستگی در بیماران همودیالیز از ابعاد جسمی و ذهنی مورد توجه می‌باشد. خستگی جسمی، به عنوان کمبود انرژی شرح داده می‌شود که در روزهای دیالیز بدتر می‌شود. خستگی با عملکرد فیزیکی، محدودیت‌های نقش، سطح فعالیت‌ها و کیفیت زندگی جسمی و ذهنی وابسته است. وجود خستگی مداوم باعث راندن بیماران به سمت جدایی از دیگران و جامعه می‌گردد. همچنین باعث اختلال در یادآوری بیماران و کاهش ارتباط با اطرافیان و بی‌توجهی به محیط اطراف می‌شود. بدنبال این کاهش شرکت در فعالیت‌های اجتماعی، بیمار به حدود ۵ ساعت زمان نیاز دارد تا به حالت عادی بازگردد.

خستگی با عوامل متعددی مانند جنسیت، سن، نژاد، سطح آموزش، میزان اضافه وزن بین جلسات همودیالیز و وزن‌گیری حین دیالیز، افسردگی، آنمی، سطح آلبومین سرم و کفایت دیالیز در ارتباط است. با توجه به تأثیر عمیق و مخرب آن برکیفیت زندگی و افزایش خطر مرگ‌ومیر مرتبط با آن در بیماران همودیالیز، بررسی عمیق‌تر آن ضروری به نظر می‌رسد.(۵۸). برخی بیماران پس از دیالیز احساس کمبود قدرت و انرژی با حس ضعف و خشک شدن بدن می‌کنند، آن‌ها از کمبود تمایل و قدرت برای شرکت در فعالیت‌ها شکایت دارند زیرا احساس خستگی بیش از حد می‌کنند و آن‌ها احساس تنبلی کرده و از عدم توانایی جهت بلند شدن و انجام کارهای خود شکایت دارند. طبق مطالعات یک رابطه معنادار بین کاهش فعالیت فیزیکی و افزایش افسردگی و خستگی وجود دارد. بدنبال کاهش فعالیت، قدرت افراد کاهش یافته، که باعث بالا رفتن افسردگی و خستگی می‌گردد(۶۳-۶۵). جهت درمان و کاهش خستگی درمان‌های دارویی و غیر دارویی وجود دارد. درمان دارویی شامل ال کارنیتین، ویتامین سی و مصرف اریتروپویتین جهت برطرف کردن آنمی می‌باشد(۵۶) و درمان‌های غیردارویی شامل انواع فعالیت ها، یوگا، ریلکسیشن، طب سوزنی و دیالیز می‌باشد(۵۶, ۶۶). انجام فعالیت حین دیالیز می‌تواند باعث کاهش میزان مرگ‌ومیر، افسردگی، اختلالات خواب و خستگی گردد(۶۳).

جهت اندازه‌گیری میزان خستگی ابزار متعددی در دسترس می‌باشند مانند پرسشنامه خستگی چالدر[۱]، فهرست خستگی چندبعدی[۲]، فرم کوتاه مقیاس زنده‌دلی[۳]، مقیاس شدت خستگی[۴] و مقیاس خستگی پیپر[۵] که V-S SF-36 در بیماران دیالیزی کاربرد بیشتری دارد(۶۷) و MFI-20 از اعتبار داخلی کمتر و مشکلات درک در بیماران دیالیزی برخوردار است(۶۸) و به علت دارا بودن تعداد زیاد سؤال موجب طولانی شدن پاسخ‌دهی و خستگی بیمار می‌شود. CFQ از ۱۱ گویه برخوردار است و در سندرم خستگی مزمن کاربرد دارد و دو بعد یعنی خستگی ذهنی و خستگی فیزیکی را می‌سنجد (۶۹). ذاکری مقدم و

---
[1] Chalder Fatigue Questionniare(CFQ)
[2] Multimensional Fatigue Invertory(MFI-20)
[3] Vitality Scale(V-S SF-36)
[4] Fatigue Severity Scale(FSS)
[5] Piper Fatigue Scale(PFS)

متدین در مطالعات خود جهت بررسی خستگی در بیماران مزمن از FSS استفاده کرده و آن را مفید می‌داند(۶۳, ۷۰).

مطالعات مختلفی در زمینه فعالیت حین دیالیز و کفایت دیالیز در جهان و کمتر از آن در ایران انجام شده است که از جمله می‌توان به مطالعه کرکمن[1] (۲۰۱۳)، در مورد انجام فعالیت با پدال زدن در حین دیالیز و افزایش زمان دیالیز اشاره کرد که در نتایج آن در گروه فعالیت تأثیر معنی‌داری در افزایش کفایت دیالیز دیده نشد و در گروه بیماران با زمان دیالیز بیشتر، نسبت به گروه کنترل، افزایش در کفایت دیالیز دیده شد(۷۱). ولی در مطالعه ویل استرن[2](۲۰۰۵)، که در مورد تأثیر انجام فعالیت کششی قبل از دیالیز و پدال زدن حین دیالیز انجام شد، افزایش کفایت دیالیز در گروه مداخله مشاهده گردید(۷۲). همچنین طی مطالعه ریاحی(۱۳۹۱)، که روی بیماران مزمن کلیوی تحت درمان با همودیالیز صورت گرفت و در آن گروه مداخله حدود ۴۰ تا ۶۰ دقیقه فعالیت در ابتدای دیالیز با استفاده از پدال داشتند، اختلاف معنی‌داری در کفایت دیالیز بیماران گروه کنترل و مداخله نشان داده نشد(۷۳). و در مطالعه شفیع پور(۱۳۹۶)، انجام فعالیت حین دیالیز و همچنین انجام فعالیت در خانه، باعث بهبود در کفایت دیالیز بیماران گردید(۷۴).

هر چند در مطالعه‌ای که در سال ۲۰۰۴ توسط وایتیلینگهام[3] در مورد افزایش زمان دیالیز به ۵ ساعت و انجام فعالیت حین دیالیز صورت گرفت و در آن به بررسی تأثیر آن‌ها در برداشت فسفات و افزایش Kt/V پرداخته شد، تغییری در افزایش کفایت دیالیز در گروه فعالیت حین دیالیز با دوچرخه ثابت دیده نشد(۷۵) ولی در مطالعه گروسارد[4](۲۰۱۵)، که در بیماران همودیالیز و انجام فعالیت ۳۰ دقیقه‌ای با دوچرخه انجام شد، باعث افزایش آمادگی جسمانی، کاهش پروفایل چربی و بهبود وضعیت آنتی‌اکسیدان گردید(۳). در سایر مطالعات که در زمینه فعالیت انجام شد، متدین(۱۳۹۳)، به بررسی اثر فعالیت حین دیالیز در بیماران کلیوی تحت درمان با همودیالیز پرداخت. این فعالیت شامل فعالیت ذهنی و جسمی بود که تأثیر آن‌ها بر میزان خستگی بیماران سنجیده شد و کاهش میزان خستگی در بیماران تحت مداخله

---

[1] Danielle L Kirkman
[2] Van Vilsteren
[3] Vaithilingam
[4] Carole Groussard

نسبت به شروع پژوهش دیده شد(۶۳). و در مطالعه پیکاریلو[1](۲۰۱۶)، که به بررسی و تعیین عوامل سایکولوژیک نظیر اضطراب، استرس، افسردگی و کیفیت خواب در میزان خستگی بیماران مزمن کلیوی پرداخت، کاهش در میزان این عوامل سایکولوژیک باعث کاهش خستگی بیماران همودیالیز می‌گردد(۶۲) و با توجه به تأثیر مثبت فعالیت بر کاهش میزان این عوامل سایکولوژیک(۷۶, ۷۷)، می‌توان نتیجه گرفت که افزایش فعالیت می‌تواند باعث کاهش میزان خستگی بیماران تحت درمان با همودیالیز شود. در مطالعات متعدد انجام شده در زمینه فعالیت حین دیالیز که در نقاط مختلف جهان انجام شده است، نتایج متفاوتی حاصل گردیده است. هر چند این گونه مطالعه در ایران کمتر انجام شده است، ولی تنها از یک جنبه و یک نوع مداخله مدنظر گرفته شده است و نسبت به سایر مداخلات جهت افزایش کفایت دیالیز و سایر پیامدها مورد بررسی قرار نگرفته است. در این پژوهش، ضمن پرداختن به اثرات فعالیت حین دیالیز بر کفایت دیالیز، اثرات بررسی آن بر سایر پیامدهای مهم مانند وضعیت همودینامیک بیماران، میزان خستگی پرداخته شد.

با توجه به نقش مهم پرستاران دیالیز در مراقبت مداوم و یکپارچه و تحت نظر داشتن بیمار و نقش مهم ایشان در آموزش بیمار همودیالیزی، می‌توان با استفاده از حضور پرستاران دیالیز در انجام مداخلات مؤثر در ارتقا و بهبود کیفیت دیالیز و سایر پیامدها و بهبود وضعیت بیمار، اعم از جسمی، روحی و روانی به نتایج بهتری دست یافت. این پژوهش با هدف بررسی میزان اثربخشی فعالیت فیزیکی حین همودیالیز بر کفایت دیالیز، شاخص‌های همودینامیک و خستگی بیماران همودیالیز در شهر بوشهر انجام شد.

۱-۲. اهداف پژوهش

۱-۲-۱. هدف کلی

مقایسه اثربخشی فعالیت فیزیکی حین دیالیز بر کفایت دیالیز، شاخص‌های همودینامیک و خستگی در بیماران تحت همودیالیز در بیمارستان‌های شهر بوشهر در سال ۱۳۹۹

[1] Federica Picariello

۱-۲-۲. اهداف ویژه (اختصاصی)

۱) تعیین ویژگی‌های جمعیت شناختی در دو گروه آزمون و کنترل در بیمارستان‌های شهر بوشهر در سال ۱۳۹۹

۲) تعیین و مقایسه میزان اوره خون و وزن در بیماران قبل و بعد از مداخله در دو گروه آزمون و کنترل به تفکیک در بیماران تحت همودیالیز در بیمارستان‌های شهر بوشهر در سال ۱۳۹۹

۳) تعیین و مقایسه میزان اوره خون و وزن بیماران قبل و بعد از مداخله بین دو گروه آزمون و کنترل در بیمارستان‌های شهر بوشهر در سال ۱۳۹۹

۴) مقایسه میانگین تغییرات اوره خون و وزن در بیماران در طول مداخله بین دو گروه آزمون و کنترل در بیمارستان‌های شهر بوشهر در سال ۱۳۹۹

۵) تعیین و مقایسه Kt/V در بیماران بعد از مداخله بین دو گروه آزمون و کنترل در بیمارستان‌های شهر بوشهر در سال ۱۳۹۹

۶) تعیین و مقایسه شاخص‌های همودینامیک (درجه حرارت، نبض، فشارخون، تنفس و درصد اشباع اکسیژن خون شریانی) در بیماران قبل و بعد از مداخله در دو گروه آزمون و کنترل به تفکیک در بیمارستان‌های شهر بوشهر در سال ۱۳۹۹

۷) تعیین و مقایسه شاخص‌های همودینامیک (درجه حرارت، نبض، فشارخون، تنفس و درصد اشباع اکسیژن خون شریانی) در بیماران قبل و بعد از مداخله بین دو گروه آزمون و کنترل در بیمارستان‌های شهر بوشهر در سال ۱۳۹۹

۸) مقایسه میانگین تغییرات شاخص‌های همودینامیک (درجه حرارت، نبض، فشارخون، تنفس و درصد اشباع اکسیژن خون شریانی) در بیماران در طول مداخله بین گروه آزمون و کنترل در بیمارستان‌های شهر بوشهر در سال ۱۳۹۹

۹) تعیین و مقایسه میزان خستگی در بیماران، قبل و بعد از مداخله در دو گروه آزمون و کنترل به تفکیک در بیماران تحت همودیالیز در بیمارستان‌های شهر بوشهر در سال ۱۳۹۹

۱۰) تعیین و مقایسه میزان خستگی در بیماران قبل و بعد از مداخله بین دو گروه آزمون و کنترل در بیمارستان‌های شهر بوشهر در سال ۱۳۹۹

۱۱) مقایسه میانگین تغییرات خستگی در بیماران، در طول مداخله بین گروه در بیماران تحت همودیالیز در بیمارستان‌های شهر بوشهر در سال ۱۳۹۹

۱-۲-۳. هدف کاربردی

- توانمندسازی بیماران همودیالیزی
- بهبود وضعیت جسمی بیماران همودیالیزی
- بهبود کیفیت زندگی بیماران همودیالیزی

۱-۳. سؤالات پژوهش

۱) ویژگی‌های جمعیت شناختی در دو گروه بیماران مشارکت‌کننده تحت همودیالیز در بیمارستان‌های شهر بوشهر در سال ۱۳۹۸ چگونه است؟

۱-٤. فرضیات پژوهش

۱) میزان اوره خون و وزن در بیماران قبل و بعد از مداخله در دو گروه به تفکیک متفاوت است.

۲) میزان اوره خون و وزن در بیماران قبل و بعد از مداخله بین دو گروه متفاوت است.

۳) تغییرات اوره خون و وزن در بیماران در طول مداخله در دو گروه متفاوت است.

۴) Kt/V در بیماران بعد از مداخله بین دو گروه متفاوت است.

۵) شاخص‌های همودینامیک (درجه حرارت، نبض، فشارخون، تنفس و درصد اشباع اکسیژن خون شریانی) در بیماران قبل و بعد از مداخله در دو گروه به تفکیک متفاوت است.

۶) شاخص‌های همودینامیک (درجه حرارت، نبض، فشارخون، تنفس و درصد اشباع اکسیژن خون شریانی) در بیماران قبل و بعد از مداخله بین دو گروه متفاوت است.

۷) تغییرات شاخص‌های همودینامیک (درجه حرارت، نبض، فشارخون، تنفس و درصد اشباع اکسیژن خون شریانی) در بیماران در طول مداخله در هر دو گروه متفاوت است.

۸) میزان خستگی در بیماران، قبل و بعد از مداخله در دو گروه به تفکیک متفاوت است.

۹) میزان خستگی در بیماران قبل و بعد از مداخله در بین دو گروه متفاوت است.

۱۰) تغییرات خستگی در بیماران، در طول مداخله در دو گروه متفاوت است.

۱-۵. تعریف مفهومی و عملیاتی واژه‌ها

۱-۵-۱. همودیالیز

تعریف علمی: همودیالیز روندی است برای خارج ساختن مایع و فراورده‌های زائد اورمیک از بدن با استفاده از عبور خون در مجاورت یک پرده نیمه‌تراوا و عبور مواد زائد و آب از این غشاء، هنگامی استفاده می‌شود که کلیه‌ها نتوانند چنین وظیفه‌ای را انجام دهند(۷۸).

تعریف عملی: در این مطالعه منظور از دیالیز، همودیالیز می‌باشد که در مرکز و تحت نظارت پرستار و محقق و به مدت ۴ ساعت و سه بار در هفته انجام می‌گردد.

۱-۵-۲. کفایت دیالیز

تعریف علمی: موسسه ملی کیفیت پیامدهای دیالیز[1] پاکسازی اوره را به عنوان کفایت دیالیز تعریف می‌کند. دیالیز بهینه (کافی) می‌تواند به عنوان درمان دیالیزی تعریف شود که باعث می‌شود بیماران به احساسی همانند زمانی‌که نارسایی کلیوی نداشته‌اند، دست یابند. انجام دیالیز با بهترین کیفیت، باعث پیامد بهتر خواهد بود.(۳۰, ۳۱).

تعریف عملی: در این مطالعه منظور از کفایت دیالیز محاسبه معیار Kt/V (Kکلیرانس اوره، T مدت زمان دیالیز، V: توزیع حجم اوره) و نسبت کاهش اوره[2] می‌باشد. (۷۹)

[1] Foundation Dialysis Outcomes Quality Initiative (NKF KDOQI)
[2] Urea Reduction Ratio

۱-۵-۳. شاخص‌های همودینامیک

تعریف علمی: شاخص‌های همودینامیک و علائم حیاتی معیارهای آماری و فیزیولوژیک مختلفی هستند که توسط پزشک و پرستار کنترل و ثبت شده و به‌منظور ارزیابی اساسی‌ترین عملکردهای بدن انسان بکار می‌روند. ثبت علائم حیاتی بخش جداناپذیر از یک پروسه درمانی است. برداشتن علائم حیاتی به‌طور طبیعی مستلزم کنترل و ثبت دما و درجه حرارت بدن، تعداد نبض (ضربان قلب)، فشارخون و تعداد تنفس است، اما گاه ممکن است شامل اندازه‌گیری‌های دیگری چون اکسیژن اشباع در خون، درد و غیره نیز باشد.(۸۰).

تعریف عملی: در این مطالعه جهت بررسی شاخص‌های همودینامیک، نبض، فشارخون، تنفس، درصد اشباع اکسیژن خون شریانی و درجه حرارت مورداندازه‌گیری قرار می‌گیرد.

۱-۵-۴. خستگی

تعریف علمی: انجمن تشخیص پرستاری آمریکا، تشخیص پرستاری خستگی را به صورت احساس ضعف، بی‌حالی، فرسودگی، نداشتن انرژی و کاهش ظرفیت برای انجام فعالیت‌های فکری و فیزیکی تعریف کرده است(۸۱). افراد خسته انرژی قبلی خود را نداشته، فعالیت کمتری انجام می‌دهند و برای حداقل فعالیت‌های روزمره تلاش بیشتری می‌کنند(۸۲).

تعریف عملی: در این مطالعه خستگی با استفاده از پرسشنامه ۹ سؤالی کروپ و همکاران (۱۹۸۹) سنجیده می‌شود و نمره‌ای که آزمودنی‌ها بر اساس طیف لیکرت از این پرسشنامه دریافت می‌کنند میزان شدت خستگی افراد را مشخص می‌کند.

۱-۵-۵. زمان دیالیز

تعریف علمی: طبق اعلام موسسه ملی کیفیت پیامدهای دیالیز، درمان همودیالیز سنتی به طور معمول برای بیماران با نارسایی مزمن کلیوی در سطح جهان ۳ تا ۵ ساعت (به‌طور متوسط ۴ ساعت)، ۳ بار در هفته انجام می‌گردد(۲۱).

تعریف عملی: در این مطالعه زمان دیالیز برای بیماران گروه کنترل و گروه مداخله فعالیت به مدت ۴ ساعت خواهد بود.

۱-۵-۶. فعالیت

تعریف علمی: به هرگونه فعالیت یا حرکت بدن که در اثر انقباض و انبساط عضلات اسکلتی ایجاد شده و نیازمند صرف انرژی است، فعالیت بدنی گفته می‌شود(۸۳).

تعریف عملی: در این مطالعه، فعالیت فیزیکی به‌صورت انجام حرکت پدال زدن با پدال مارک Amitted در دو ساعت ابتدایی دیالیز به شکل دو دوره ۱۵ دقیقه‌ای و با ۱۵ دقیقه استراحت بین آن‌ها صورت می‌گیرد.

فصل دوم

چارچوب پنداشتی

و مروری بر مطالعات

۲. مقدمه

در این بخش، در ابتدا به تئوری رفتار برنامه‌ریزی شده به عنوان زیر بنای فکری این مطالعه اشاره می‌شود و سپس به انواع اختلالات کلیوی دیالیز و عوامل مرتبط با آن و همچنین سایر مفاهیم مطالعه از جمله کیفیت زندگی، شاخص‌های همودینامیک و خستگی اشاره خواهد شد. در پایان ضمن مروری بر مطالعات داخلی و خارجی در پایان جمع‌بندی نهایی از مرور متون ارائه می‌گردد.

۲-۱. چارچوب پنداشتی

۲-۱-۱. تئوری رفتار برنامه‌ریزی شده

یکی از تئوری‌های آموزشی که بر قصد رفتاری بنا نهاده شده تئوری رفتار برنامه‌ریزی شده[1] است که توسط آژن[2] در سال ۱۹۹۱ ارائه شد(۸۴). بر اساس تئوری رفتار برنامه‌ریزی شده رفتار یک فرد تابعی از قصد اوست و قصد به وسیله ساختارهای نگرش نسبت به رفتار، هنجارهای ذهنی و کنترل رفتار درک‌شده تعیین می‌شود. رفتار برنامه‌ریزی شده چهارچوب مفهومی مفیدی برای برخورد با پیچیدگی‌های رفتار اجتماعی انسان فراهم می‌کند. این تئوری برخی از مفاهیم اصلی علوم رفتاری و اجتماعی را در برمی‌گیرد و این مفاهیم را به صورتی تعریف می‌کند که امکان پیش‌بینی و فهم رفتارهای اختصاصی در بافت‌های ویژه فراهم شود. ساختارهای ذکرشده معمولاً قصد رفتاری را با صحت بالایی پیش‌بینی می‌کنند(۸۵). هنگام استفاده از رفتار برنامه‌ریزی شده به عنوان یک چارچوب نظری، هدف مداخلات تغییر باورهای رفتاری،

[1] Theory of Planned Behavior(TPB)
[2] Ajzen

هنجاری و/یا کنترلی و در نتیجه ایجاد انگیزه در عملکرد رفتار است. به این معنا که یک مداخله موفق می‌تواند باورها را در مورد پیامدهای مثبت افزایش و باورها را در مورد پیامدهای منفی کاهش دهد و مهارت‌ها یا دانش را برای انجام رفتار افزایش دهد و موانع واقعی را کاهش داده یا تسهیل‌کننده واقعی ایجاد کند و این تصور را تقویت کند که دیگران مهمترین رفتار را تأیید می‌کنند(۸۶). قابلیت رفتار برنامه‌ریزی شده در پیش‌بینی رفتار تغذیه‌ای و فعالیت فیزیکی قبلاً به اثبات رسیده است(۸۷, ۸۸). استفاده از رفتار برنامه‌ریزی شده از این نظر مهم است که پیش‌بینی و فهم چرایی انجام رفتارهای اختصاصی را در اختیار ما قرار می‌دهد. قابلیت تئوری رفتار برنامه‌ریزی شده در ارتباط با رفتارهای تغذیه‌ای (محدودیت سدیم و پتاسیم) و فعالیت فیزیکی در بیماران همودیالیزی محک زده شده است و راهکارهای مفیدی در طراحی مداخلات آموزشی در این بیماران را در اختیار مراقبین سلامت قرار می‌دهد؛ و راه را برای انجام مداخلات کم‌هزینه و در عین حال اثربخش هموار می‌کند(۸۹). چارچوب ذهنی در این مطالعه‌این است که همودیالیز در عین حالی‌که روش مناسبی برای بهبود وضعیت سلامت بیماران کلیوی است ولی می‌تواند خستگی را به همراه داشته باشد اما با روش‌هایی از جمله فعالیت فیزیکی که یک مداخله کم‌هزینه و راحت برای بیماران همودیالیز می‌باشد، می‌توان اثربخشی کیفیت و وضعیت همودینامیک را بهبود بخشید.

۲-۱-۲. کلیه

۲-۱-۲-۱. عملکرد کلیه

کلیه‌ها یک جفت ارگان لوبیایی شکل و قرمز مایل به قهوه‌ای هستند و در فضای خلف صفاقی (پشت و خارج حفره صفاقی) روی دیواره خلفی شکم از مهره دوازدهم سینه‌ای تا مهره سوم کمری بالغین قرار دارد. وزن کلیه افراد بالغ ۱۱۳ تا ۱۷۰ گرم و ابعاد آن ۱۰ تا ۱۲ سانتی مترطول، ۶ سانتی متر عرض و ۲/۵ سانتی متر ضخامت است (۹۰). کلیه‌ها دارای یک قسمت قشری به قطر یک سانتی متر و یک قسمت مرکزی که از ۱۶-۱۵ توده هرمی شکل به نام هرم‌های کلیوی تشکیل یافته است. رأس این هرم‌ها به طرف مرکز و ناف کلیه متوجه بوده و مجاری آن‌ها به داخل کالیس ها[1] باز می‌شوند. از به هم پیوستن کالیس ها،

[1] Callis

لگنچه به وجود می‌آید، بالاخره در ناحیه ناف لگنچه[1] به شکل قیفی باریک شده و به حالب[2] منتهی می‌شود. حالب در پشت صفاق به طرف پایین کشیده شده، از لبه لگن خاصره می‌گذرد و به مثانه[3] ختم می‌گردد و مثانه ادرار را از طریق پیشابراه[4] خارج می‌کند (۹۱). واحد عملی کلیه، نفرون می‌باشد و ادرار در این قسمت تشکیل می‌شود. دو کلیه در مجموع دارای ۲۴۰۰۰۰۰ نفرون می‌باشند (۹۲). نفرون، لوله پیچیده‌ای است که از یک لایه بافت پوششی که در یک پایانه بسته است و در پایانه دیگر به درون لگنچه باز می‌شود، ساخته شده است. تعداد نفرون‌ها در انسان پس از تولد افزایش نمی‌یابد؛ اما درازای نفرون در دوره رشد بیشتر می‌شود. هر نفرون از کپسول بومن، لوله پیچ‌خورده نزدیک، لوله هنله[5]، لوله پیچ‌خورده دور و مجرای جمع کننده که بین چندین نفرون مشترک است و مختص یک نفرون نیست، تشکیل شده است. در هر دقیقه ۵۰۰ سی‌سی پلاسما وارد نفرون شده و تنها ۱۰۰ سی‌سی از آن پالایش می‌گردد. با نگرش به این که در همین مدت تنها کمتر از ۱ سی‌سی ادرار پایه‌ریزی می‌گردد، بنابراین بیشتر از ۹۹ درصد از آبی که وارد کپسول بومن[6] شده‌است، در عمل باز جذب به خون بازگشت داده می‌شود. مهم‌ترین عمل کلیه‌ها در انسان و پستانداران دفع مواد نیتروژن دار مانند اوره است. نفرون‌ها می‌توانند اوره را تا صد برابر در ادرار افزایش دهند؛ ولی اگر میزان آن در ادرار به ۵ درصد برسد فرآوری مسمومیت می‌کند (۹۳).

عملکرد کلیه از این نظر مهم است که نقش‌های بسیار مهمی در بدن به عهده دارند:

۱. دفع مواد زائد متابولیکی و سایر مواد سمی تنظیم حجم مایعات بدن
۲. نگهداری تعادل الکترولیتی
۳. تنظیم PH خون (۹۴).

[1] Pelvis
[2] Ureter
[3] Bladder
[4] Urethra
[5] Loop Of Henle
[6] Bowman's Capsule

علاوه بر این، کلیه‌ها نقش مهم دیگری نیز به عهده دارند که عبارت است از: تولید رنین که بر میزان سدیم، حجم مایعات و فشارخون تأثیر می‌گذارد و منجر به تولید اریتروپوئتین که تولید گلبول‌های قرمز خون را تنظیم می‌کند، می‌گردد(۹۳). همچنین کلیه سالم گیرنده چند هورمون است:

- هورمون آنتی دیورتیک که توسط غده هیپوفیز ترشح و باعث کاهش دفع آب می‌شود.
- هورمون آلدوسترون[1] که توسط غده فوق کلیوی تولید شده و باعث احتباس سدیم، ترشح یون هیدوژنوپتاسیم[2] می‌شود.
- هورمون پاراتیروئید که ترشح فسفر و بی‌کربنات را افزایش می‌دهد و محرکی است برای تبدیل ویتامین D به فرم فعال ویتامین D3[3] است(۹۵).

۲-۱-۲-۲. فیزیولوژی کلیه

کلیه عضو بسیار پرعروقی است. حجمی معادل ۲۰ تا ۲۵ درصد برون ده قلبی در حالت استراحت را دریافت می‌کند که بیشتر از ml/min ۱۰۰۰ است. هر کلیه از شریان کلیوی که از آئورت بطنی جدا می‌شود، خون دریافت می‌کند و از راه ورید کلیوی خون از کلیه‌ها خارج می‌شود. شریان کلیوی به شریانچه‌های آوران منشعب می‌شود که مویرگ‌های گلومرولار نیز به هم می‌پیوندند و شریانچه‌های وابران را تشکیل می‌دهند(۹۴, ۹۶). میزان جریان خون کلیه به میزان مایعات بدن و برون ده قلبی بستگی دارد. از دست رفتن آب بدن، از دست دادن خون، نارسایی احتقانی قلب[4]، انفارکتوس میوکارد[5] مواردی است که می‌تواند خون‌رسانی کلیه‌ها را مختل کند و باعث نارسایی کلیه‌ها شوند(۹۵, ۹۶). این بیماری در بیمارانی که میزان دفع پروتئین قابل توجهی دارند یا اینکه فشارخون بالاتری نسبت به آن‌هایی که چنین شرایطی را ندارند، سریع‌تر پیشرفت می‌کند (۹۷).

---
[1] Aldosterone
[2] Hydogen Potassium
[3] 1, 25 (Oh)2 D3
[4] Congestive Heart Failure
[5] Myocardial Infarction

۲-۱-۲-۳. نارسایی کلیه

نارسایی کلیوی، در سراسر دنیا، یکی از مشکلات عمده سلامت عمومی محسوب می‌شود و به آسیب موقتی یا دائمی کلیه‌ها اطلاق می‌گردد که منجر به از دست دادن عملکرد طبیعی کلیه‌ها می‌شود. بنابر گزارش مرکز مدیریت پیوند و بیماری‌های خاص وزارت بهداشت، جمعیت بیماران دچار نارسایی کلیه در کشور ۳۲۰ هزار نفر است که ۴۹ درصد این بیماران از روش درمان پیوند و ۴۸ درصد از دیالیز و ۳ درصد از روش دیالیز صفاقی استفاده می‌کنند. روند رو به رشد این بیماران در جهان بیانگر آن است که تعداد بیماران تحت درمان‌های جایگزین، مثل پیوند کلیه، دیالیز صفاقی و همودیالیز نیز رو به فزونی است. دیالیز روندی است برای خارج ساختن مایع و فرآورده‌های زائد و اورمیک از بدن و هنگامی استفاده می‌شود که کلیه‌ها نتوانند چنینی وظیفه‌ای را انجام دهند، دیالیز خونی شایع‌ترین روش دیالیز است. در بیماران مبتلا به نارسایی مزمن کلیه دیالیز خونی از مرگ جلوگیری می‌کند هرچند درمان‌کننده بیماری‌های کلیوی نبوده و فعالیت درون‌ریز و متابولیک کلیه‌ها را جبران نمی‌کند(۹۸-۱۰۰).

نارسایی کلیه زمانی اتفاق می‌افتد که کلیه‌ها قادر به دفع مواد زائد متابولیک یا انجام عملکردهای تنظیمی نباشد. موادی که در حالت طبیعی در ادرار دفع می‌شوند، به دلیل اختلال در عملکرد کلیوی، در مایعات بدن تجمع می‌یابند و بر کارکردهای درون‌ریز و متابولیک اثر می‌گذارند و موجب بر هم خوردن تعادل مایع و الکترولیت و اسید - باز می‌شوند. بیماری کلیوی یک بیماری سیستمیک است که سرانجام بیشتر بیماری‌های کلیه و مجاری ادراری به آن ختم می‌شود. هر سال تعداد مرگ‌های ناشی از بیماری‌های کلیوی برگشت‌ناپذیر افزایش می‌یابد(۷۸).

۲-۱-۲-۴. انواع نارسایی کلیه

نارسایی کلیه با توجه به ماهیت و سیر بیماری به دو فرم نارسایی حاد کلیه[1] و نارسایی مزمن کلیه[2] دسته‌بندی می‌شود(۹۵).

[1] Acute Renal Failure (Arf)
[2] Chronic Kidney Disease (Ckd)

۲-۱-۲-۴-۱. نارسایی حاد کلیه

نارسایی حاد کلیه یک کاهش ناگهانی در عملکرد کلیه ناشی از آسیب کلیه‌ها می‌باشد. با توجه به مدت و شدت نارسایی حاد کلیه، دامنه وسیعی از عوارض متابولیک بالقوه تهدیدکننده زندگی ممکن است ایجاد شود که شامل اسیدوز متابولیک و اختلال مایع و الکترولیت هست. هدف از درمان، جایگزین کردن موقت عملکرد کلیه، جهت به حداقل رساندن عوارض کشنده و کاهش عوامل افزایش‌دهنده آسیب کلیوی باهدف کوتاه کردن مدت زمان‌کاهش عملکرد کلیه هست. درمان به‌موقع نارسایی حاد کلیه باعث بهبودی شده و معمولاً عارضه‌ای به‌جای نمی‌گذارد(۹۴, ۹۵).

آسیب حاد کلیه از دست دادن سریع عملکرد کلیه به دنبال آسیب کلیه است. بسته به مدت و شدت آسیب حاد کلیه، طیف وسیعی از عوارض متابولیکی تهدیدکننده حیات ممکن است رخ دهد که شامل اسیدوز متابولیک و عدم تعادل مایعات و الکترولیت‌ها است. هدف از درمان، جایگزین کردن عملکرد کلیه به طور موقتی برای به حداقل رساندن عوارض مرگبار و کاهش علل بالقوه‌ی افزایش آسیب کلیوی با هدف به حداقل رساندن طولانی‌مدت کاهش عملکرد کلیه می‌باشد. آسیب حادکلیه مشکلی است که در بیماران بستری در بیمارستان و همچنین بیماران سرپایی دیده می‌شود. در آسیب حاد کلیه میزان کراتینین سرم ممکن است یک افزایش ۵۰ درصد یا بیشتر نسبت به حد پایه داشته باشد (میزان طبیعی کراتینین کمتر از یک میلی‌گرم در دسی لیتر است). در این حالت حجم ادرار نرمال و یا می‌تواند متغیر باشد. تغییرات احتمالی که در ادرار دیده می‌شود، عبارتند از: عدم اولیگوری (حجم ادرار بیشتر از ۸۰۰cc در روز)، اولیگوری[1] (حجم ادرار کمتر از ۵۰۰cc در روز)، یا آنوری (حجم ادرار کمتر از ۵۰cc در روز) (۷۸).

۲-۱-۲-۴-۲. بیماری مزمن کلیوی

بیماری مزمن کلیوی[2] به آسیب کلیوی یا کاهش میزان فیلتراسیون گلومرولی[3] برای ۳ ماه یا بیشتر گفته می‌شود. بیماری مزمن کلیوی با کاهش کیفیت زندگی، افزایش هزینه‌های مراقبت‌های بهداشتی و

---

[1] Oliguria
[2] Chronic Kidney Disease(CKD)
[3] Glomerular Filtration Rate(GFR)

مرگ زودرس در ارتباط است. در صورت عدم درمان، نارسایی مزمن کلیوی منجر به مراحل آخر بیماری کلیوی می‌شود که اختلال پیش‌رونده و برگشت‌ناپذیر عملکرد کلیوی در نتیجه احتباس مواد زاید اورمیک است و نیاز به استفاده از درمان‌های جایگزین کلیه نظیر دیالیز و پیوند کلیه می‌گردد. عوامل خطر شامل بیماری‌های قلبی عروقی، دیابت، فشارخون و چاقی می‌باشند. تحقیقات اخیر حاکی از آن است که در ایالات‌متحده ۱۰ درصد از جمعیت ۲۰ سال و بالای آن مبتلا به بیماری مزمن کلیوی هستند.

دیابت اولین و شایع‌ترین دلیل نارسایی مزمن کلیه است کفایت دیالیز همچنین شاخصی برای میزان مرگ‌ومیر و از پیامدهای اولیه دیالیز می‌باشد. بیش از ۳۵ درصد جمعیت ۲۰ سال و بالاتر افراد دیابتی بیماری مزمن کلیوی دارند. دومین دلیل بیماری مزمن کلیوی هیپرتانسیون به دنبال گلومرونفریت و پیلونفریت، کلیه پلی‌کیستیک و سرطان کلیه، وراثت و یا اختلالات ژنتیکی است. در ایالات متحده بیش از ۲۰ درصد از جمعیت ۲۰ سال و بالای آن که فشارخون بالا دارند مبتلا به بیماری مزمن کلیوی هستند(۷۸).

۲-۱-۲-۴-۳. پاتوفیزیولوژی

در مراحل اولیه بیماری مزمن کلیوی، ممکن است آسیب عمده‌ای به کلیه‌ها وارد شده باشد، ولی علائم و نشانه‌ای وجود ندارد، پاتوفیزیولوژی بیماری مزمن کلیوی تاکنون به طور واضح شناخته نشده اما به نظر می‌رسد آسیب کلیه‌ها به‌واسطه‌ی التهاب حاد طولانی‌مدت باشد که مربوطه به ارگان خاصی نیست و همچنین همراه با علائم عمومی خفیف است(۷۸).

۲-۱-۲-۵. مراحل بیماری مزمن کلیه

بیماری مزمن کلیوی توسط موسسه ملی کلیه به پنج مرحله طبقه‌بندی شده است (شکل ۲-۱). مرحله پنجم زمانی ایجاد می‌شود که کلیه‌ها قادر به دفع مواد زائد ناشی از فعالیت‌های متابولیکی بدن و یا انجام عملکردهای تنظیمی نباشند، بنابراین درمان‌های جایگزین کلیه برای ادامه حیات ضروری است. غربالگری و نیز مداخلات اولیه بسیار مهم هستند زیرا مانع از پیشرفت همه‌ی بیماران به سمت مرحله‌ی پنجم CKD می‌شوند. مبتلایان به CKD در معرض خطر بیشتری برای ابتلا به بیماری‌های قلبی - عروقی هستند که این مسئله علت اصلی ابتلا به بیماری و مرگ‌ومیر می‌باشد. درمان پرفشاری خون، آنمی و

هایپرگلیسمی و ردیابی پروتئینوری در کند کردن پیشرفت بیماری و ارتقای نتایج کمک‌کننده می‌باشد(۷۸).

٪ عملکرد کلیه	GFR*	مراحل بیماری مزمن کلیوی	
90-100%	۹۰ یا بیشتر	آسیب به کلیه با عملکرد نرمال	مرحله ۱
89-60%	۶۰-۸۹	آسیب به کلیه و از دست دادن قسمتی از عملکرد آن	مرحله ۲
59-45%	۴۵-۵۹	از دست دادن جزئی تا متوسط عملکرد کلیه	مرحله ۳a
44-30%	۳۰-۴۴	از دست دادن متوسط تا شدید عملکرد کلیه	مراحل ۳b
29-15%	۱۵-۲۹	از دست دادن شدید عملکرد کلیه	مرحله ۴
Less than 15%	کمتر از ۱۵	نارسایی مزمن کلیوی	مرحله ۵

عدد GFR نشاندهنده میزان عملکرد کلیه بوده و با کاهش آن، بیماری کلیوی تشدید میشود.

شکل ۲-۱. مراحل بیماری مزمن کلیه

۲-۱-۲-۵-۱. تظاهرات بالینی

افزایش سطح کراتینین سرم نشان‌دهنده بیماری کلیوی اساسی است. زمانی که سطح کراتینین افزایش می‌یابد، نشانه‌های بیماری مزمن کلیوی شروع می‌شود. کم‌خونی به علت کاهش تولید اریتروپوئیتین توسط کلیه‌ها، اسیدوز متابولیک و تغییرات غیرطبیعی در کلسیم و فسفر، بیان‌کننده‌ی پیشرفت CKD

هستند. احتباس مایع سبب ادم و نارسائی احتقانی قلب[1] می‌گردد. با پیشرفت این بیماری، تغییرات غیرطبیعی در الکترولیت‌ها رخ می‌دهد، نارسایی قلبی بدتر شده و کنترل فشارخون مشکل‌تر می‌شود(۷۸).

۲-۱-۲-۵-۲. بررسی و یافته‌های تشخیصی

GFR میزان پلاسمای فیلتر شده از طریق گلومرول در واحد زمان می‌باشد. کلیرانس (پاکسازی) کراتینین، میزان کراتینینی است که کلیه‌ها قادر به دفع آن در طی ۲۴ ساعت هستند. مقادیر طبیعی این یافته‌ها در زنان و مردان متفاوت است. محاسبه GFR به عنوان یک شاخص بررسی مهم در CKD می‌باشد(۷۸).

۲-۱-۲-۵-۳. تدابیر درمانی

درمان بیماران مبتلا به CKD شامل درمان بیماری زمینه‌ای است. بررسی‌های منظم بالینی جهت حفظ و نگهداشتن فشارخون کمتر از ۱۳۰ میلی‌متر جیوه، بسیار مهم است. تدابیر درمانی همچنین شامل ارجاع زودهنگام بیمار برای آغاز درمان‌های جایگزین کلیه بر اساس وضعیت کلیوی بیمار، پیشگیری از عوارض با کنترل عوامل خطر بیماری‌های قلبی، درمان هیپرگلیسمی، آنمی، قطع سیگار، کاهش وزن، داشتن برنامه ورزشی، کاهش مصرف نمک و الکل می‌باشد(۷۸).

۲-۱-۲-۵-۴. روش‌های جایگزین درمان کلیه

نارسایی نهایی کلیه با افزایش اوره و کراتینین خون و عدم تعادل الکترولیتی مشخص می‌شود و وقتی که بیمار به این نقطه می‌رسد معمولاً برای ادامه زندگی نیاز به ادامه درمان‌های جایگزینی شامل دیالیز صفاقی[2]، همودیالیز و پیوند کلیه می‌باشد (۱۰۱).

الف) دیالیز صفاقی

یکی از روش‌های جایگزین کارکرد کلیه، دیالیز صفاقی می‌باشد. در این روش انتقال مواد و آب از طریق یک پرده زنده که صفاق بیمار است انجام می‌شود. این پرده بین خون بیمار (عروق خونی پرده صفاق)

---

[1] Congestive Heart Failure(CHF)
[2] Pretoneal Dialysis (Pd)

و مایع دیالیز (مایع صفاق) است و موجب انتقال اوره،کراتینین و دیگر توکسین های اورمیک از خون بیمار به مایع صفاق می‌شود و سپس با سیکل‌های دیالیز از بدن خارج می‌شوند. در واقع در انتقال مواد از پرده صفاق، دیواره مویرگ‌های صفاقی بیشترین نقش را دارا هستند و انتقال آب و مواد را در سه اندازه مختلف عهده‌دار هستند(۱۰۲).

ب) همودیالیز

همو به معنای خون است و دیالیز به جدا کردن یا فرایند پالایش اشاره دارد. سموم یا مواد زائد متابولیک توسط یک پرده نیمه تراوا از خون پالایش و از راه مایع دیالیز بیرون ریخته می‌شـــود (۱۰۳). اولین دســتگاه همودیالیز در سال ۱۹۱۳ توسط Able ارائه شد. اولین همودیالیز موفق در سال ۱۹۴۴ در هلند توسط Berk و Kolf انجام گردید. در ایران همودیالیز از اوایل دهه ۱۳۵۰ شروع شده است (۱۰۴).

هدف همودیالیز خارج کردن مواد سمی از خون و خارج کردن آب اضافی از بدن می‌باشد. یک کلیه مصنوعی که غشاء مصنوعی نیمه تراوا است، جایگزین گلومرول‌ها و توبول‌های کلیوی جهت فیلتراسیون می‌شود. در همودیالیز، خون مملو از مواد سمی و دفعی نیتروژن دار از بدن بیمار به ماشین و دیالیزر[1] وارد می‌شود، سموم تصفیه و برداشته می‌شود و خون مجدداً به بیمار برمی‌گردد (۹۵). انجام همودیالیز در مواردی نظیر صعود فزاینده سطح پتاسیم سرم، افزایش حجم و فشار مایعات یا ادم ریوی، اسیدوز پیش‌رونده، پریکاردیت و اورمی پیشرونده، ضرورت پیدا می‌کند. همچنین برای خارج ساختن داروها یا سایر سموم از خون، نظیر مسمومیت با داروها و سموم نیز استفاده می‌شود. دیالیز نگهدارنده در بیماران مبتلا به نارسایی مزمن کلیه در صورت وجود علایم حاد اورمیک (تهوع، استفراغ، بی‌اشتهایی شدید، افزایش گیجی و اختلالات روانی)، کاهش پتاسیم خون[2]، افزایش حجم مایعات که به درمان با دیورتیک‌ها و محدودیت با مایعات هم جواب نمی‌دهد و احساس ناخوشی عمومی کاربرد دارد (۷۸). برای بیمارانی که نارسایی مزمن کلیه دارند، همودیالیز موجب جلوگیری از مرگ می‌شود. بیش از ۹۰ درصد از بیمارانی که

[1] Dialyzer
[2] Hypokalemia

به درمان طولانی مدت جایگزین کلیه نیاز دارند، تحت درمان با همویالیز هستند. اغلب بیماران، همودیالیز متناوب را سه بار در هفته با طول مدت ۳ تا ۵ ساعت در مراکز سرپایی دریافت می‌کنند (۹۲).

این درمان برای بیمارانی که دچار ادم مقاوم به درمان، کمای کبدی، هیپرکلسمی، هیپرتانسیون و اورمی هستند نیز کاربرد دارد(۷۸). یکی از موارد اورژانسی اندیکاسیون دیالیز، که عموماً در بیماران با نارسایی مزمن کلیه ایجاد می‌شود، ایجاد صدای مالش پریکاردی است، که نشانگر پریکاردیت اورمیک است. تصمیم برای شروع دیالیز، فقط پس از مشورت با بیمار، خانواده وی، پزشک معالج و سایر افراد تیم درمان بیمار انجام پذیرد. پرستار می‌تواند با پاسخ به سؤالات، اطلاع‌رسانی شفاف و حمایت از تصمیم‌گیری به بیمار و خانواده کمک نماید. یک پیوند کلیه موفقیت‌آمیز می‌تواند نیاز برای همودیالیز را مرتفع سازد. پیوند کلیه تنها سبب بهبود کیفیت زندگی در افراد دچار ESKD نمی‌شود، بلکه عملکرد فیزیولوژیک بدن نیز به مراتب بهتر می‌گردد. بیمارانی که قبل از شروع دیالیز از یک دهنده زنده، پیوند کلیه می‌شوند، عمری به مراتب طولانی‌تر از افرادی دارند که قبل از پیوند کلیه تحت درمان با دیالیز قرارگرفته بودند. همودیالیز برای بیمارانی که به طور حاد بیمار بوده و نیاز به دیالیزهای کوتاه مدت مانند دیالیز روزانه یا هفتگی تا زمان برگشت عملکرد کلیه و همچنین در بیمارانی که دچار ESKD بوده و نیازمند درمان همیشگی و بلندمدت هستند، استفاده می‌شود. برای بیمارانی که نارسایی مزمن کلیه دارند، همودیالیز موجب جلوگیری از مرگ می‌شود. اگرچه این روش، بیماری کلیوی را بهبود نمی‌بخشد و از بین رفتن فعالیت‌های آندوکرین و متابولیکی کلیه را جبران نمی‌کنند اما جان بیمار را نجات می‌دهد. بیش از ۹۰ درصد از بیمارانی که به درمان طولانی مدت جایگزین کلیه نیاز دارند، تحت درمان با دیالیز خونی مزمن هستند. همودیالیز ممکن است در منزل توسط بیمار یا مراقبین وی انجام پذیرد. با دیالیز خانگی، زمان درمان و فواصل تکرار آن متناسب با نیازهای دلخواه بیمار تنظیم می‌گردد هدف از همودیالیز استخراج مواد نیتروژنی سمی از خون و خارج کردن آب اضافی از بدن می‌باشد. در همودیالیز، خون آلوده به سموم و مواد نیتروژنی، از بیمار به یک ماشین منتقل می‌شود، این ماشین یک دیالیزر[1] (کلیه مصنوعی است که خون در آن تصفیه از طریق

[1] Dialyzer

غشا نیمه تراوا) شده و سپس به بیمار برگردانده می‌شود. اساس کار همودیالیز عبارت‌اند از: انتشار[۱]، اسموز[۲] و اولترافیلتراسیون[۳]. سموم و فرآورده‌های زاید خونی توسط انتشار دفع می‌شوند که در طی انتشار مواد از جایی که غلظت بیشتری دارد، یعنی از خون، به جایی که غلظت کمتری دارد، یعنی دیالیزر، منتقل می‌شود. محلول دیالیز محلولی است متشکل از تمام الکترولیت‌هایی که در مایع خارج سلولی با غلظت‌های معین، وجود دارند. با تنظیم دقیق محلول دیالیز می‌توان سطوح الکترولیت خون را تحت کنترل گرفت، غشاء نیمه تراوا، از عبور مولکول‌های بزرگی مثل سلول‌های قرمز خون و پروتئین‌ها ممانعت می‌نماید. آب اضافی از طریق اسموز از بدن خارج می‌شود در طی این فرآیند، آب از منطقه‌ای که غلظت مواد محلول کمتر است، یعنی خون به منطقه‌ای که غلظت مواد محلول بیشتر است، یعنی محلول دیالیز، منتقل می‌شود. در اولترافیلتراسیون، آب، تحت‌فشار زیاد از منطقه‌ای با فشار کم عبور می‌کند که این فرآیند در خارج کردن آب اضافی، مؤثرتر از اسموز می‌باشد، ضمناً در این کار از یک فشار منفی یا نیروی ساکشن روی غشای دیالیز نیز استفاده می‌شود. به دلیل اینکه بیماران کلیوی معمولاً قادر به دفع آب از بدن خود نیستند، این نیرو برای خارج کردن آب و حفظ تعادل مایع ضروری می‌باشد. سیستم بافری بدن با استفاده از محلول دیالیزی که از بی‌کربنات (شایع‌ترین ترکیب) یا استات ساخته شده، حفظ می‌شود، که استات نیز به بی‌کربنات متابولیزه می‌گردد. برای جلوگیری از انعقاد خون در مدار دیالیز، هپارین تجویز می‌شود و سرانجام خون تصفیه شده با هدف دفع مایعات، تعادل الکترولیتی و اصلاح اسیدوز به بدن برمی‌گردد(۷۸).

۲-۱-۲-۶. دیالیزر

صافی‌های دیالیز، دستگاه‌هایی با فیبرهای توخالی می‌باشند که از هزاران لوله نازک تشکیل می‌گردد که خون در میان توبول ها حرکت می‌کند. این لوله‌ها متخلخل هستند و به عنوان غشاء نیمه تراوا عمل می‌کنند، تبادل سموم، مایع و الکترولیت‌ها از طریق غشاء نیمه‌تراوای توبول ها، اتفاق می‌افتد. گردش مداوم محلول دیالیز در اطراف توبول ها، شیب غلظتی را جهت تسهیل تبادل مواد زاید خون از طریق غشای نیمه

---
[1] Diffusion
[2] Osmosis
[3] Ultrafiltration

تراوا به مایع دیالیز و دفع آن فراهم می‌نماید (شکل ۱). یکی دیگر از پیشرفت‌های تکنولوژیکی و تغییرات فن‌آوری ساخت این دستگاه‌ها، دیالیزر با سرعت زیاد است که در آن از نفوذپذیری زیاد غشاءها استفاده می‌شود که این موضوع کلیرانس مولکول‌های با وزن کم و متوسط را افزایش می‌دهد. این غشاءهای مخصوص، سرعت معمول ورود و خروج جریان خون، در دیالیز را افزایش می‌دهند (۳۰۰ml/min-۵۰۰). دیالیزهای با سرعت بالا، کیفیت درمان را افزایش داده و مدت زمان دیالیز و همچنین نیاز به هپارین را در طول دیالیز کاهش می‌دهد(۷۸).

۲-۱-۲-۶-۱. دسترسی عروقی[1]

دستیابی به سیستم عروقی بیمار باید به‌گونه‌ای باشد که اجازه جریان خون جهت تصفیه و تمیز کردن و همچنین برگشت آن به سیستم عروقی بیمار با سرعت ۸۰۰-۳۰۰ ml / min را بدهد. راه‌های متعددی برای دستیابی به عروق وجود دارد. دستیابی به عروق معمولاً با مشخص نمودن محل توسط عکس‌برداری از طریق عمل جراحی، در اتاق عمل، بخش رادیولوژی و یا بر بالین بیمار انجام می‌شود.

۲-۱-۲-۶-۲. راه‌های دسترسی داخل عروقی

دسترسی فوری به گردش خون بیمار برای همودیالیز حاد، با گذاشتن یک کاتتر دو مجرایی کاف دار با قطر بزرگ در یکی از وریدهای ساب کلاوین، ژیگولار داخلی یا فمورال، توسط پزشک امکان‌پذیر است (شکل ۲-۲).

این روش دسترسی عروقی دارای خطراتی است (نظیر هماتوم، پنوموتوراکس، عفونت، ترومبوز ورید زیر ترقوه‌ای، جریان ناکافی) در هنگامی که نیاز طولانی مدت به کاتتر نباشد، مثل موارد بهبود وضعیت بیمار یا دستیابی به دیگر راه‌های دسترسی عروقی، کاتتر خارج می‌شود. همچنین می‌توان کاتترهای دارای کاف دو مجرائی را توسط جراح یا رادیولوژیست در ورید زیر ترقوه‌ای بیمار وارد کرد. از آنجا که این کاتترها دارای کافی در زیرپوست هستند لذا در بهبود محل ورود کاتتر، بستن محل زخم و خطر عفونت بالارونده کاهش می‌یابد. این مشخصه سبب می‌شود که این کاتتر ها برای استفاده طولانی مدت مناسب باشند.

[1] Vascular Access

اگرچه میزان عفونت، برجای ماندن طولانی و تداوم سپتی سمی از دلایل عمده‌ی بستری در بیمارستان هستند(۷۸).

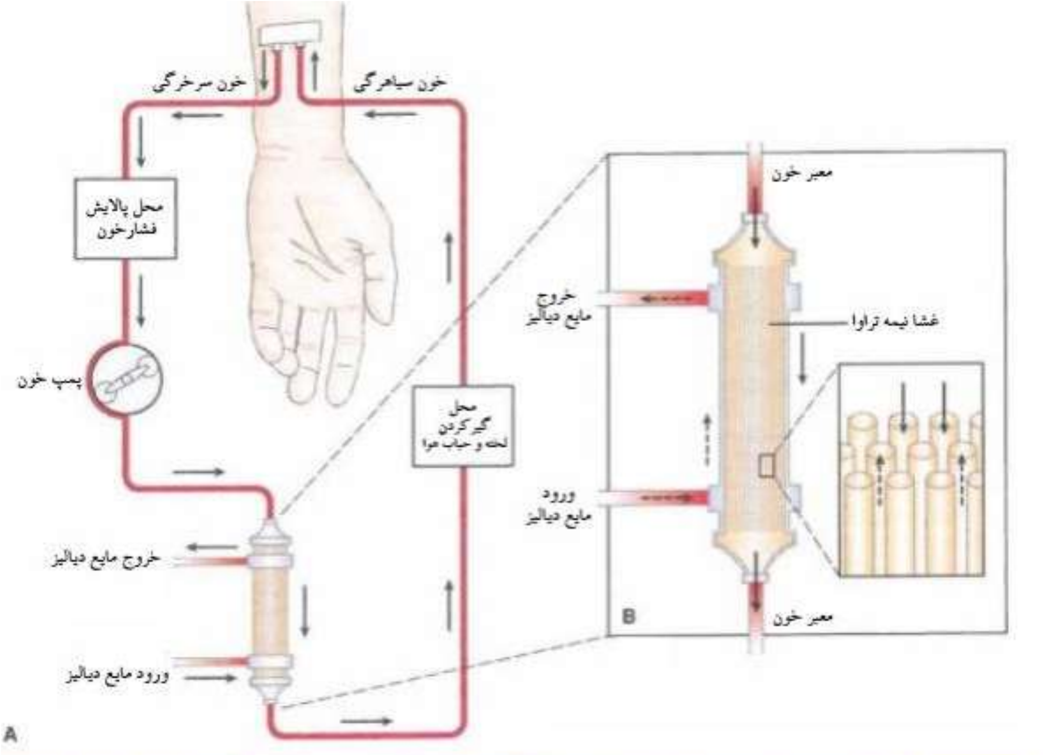

دستگاه همودیالیز. خون از یک شریان به یک دستگاه دیالیز پمپ می‌شود(A) و در آنجا از داخل لوله ها
سلوفانی جریان می یابد(B) که به عنوان غشای نیمه تراوا عمل می کنند. محلول دیالیز با ترکیب شیمیای مشابه خون (به ج
اوره و فرآورده های زاید)، در اطراف لوله جریان دارد. فرآورده های زاید در خون از طریق غشای نیمه تراوا به محلو
دیالیز انتشار می یابند.

شکل ۲-۲. ماشین‌های دیالیز

۲-۱-۲-۶-۳. فیستول شریانی وریدی

یک روش دائمی‌تر برای دستیابی به عروق، فیستول شریانی وریدی[1] است که از طریق جراحی در ناحیه ساعد با اتصال پهلوبه‌پهلو یا انتها به پهلوی یک ورید و یک شریان، ایجاد می‌شود. (شکل ۲-۳) برای تأمین جریان خون کافی جهت عبور از دیالیزر، سوزن‌هایی داخل این فیستول وارد می‌شود که برای جریان خون شریانی به دیالیزر از قسمت شریانی فیستول و از قسمت ورید آن، برای برگشت مجدد خون دیالیز

[1] Arterial Venus Fistula(AVF)

شده، استفاده می‌شود. فیستول برای آماده شدن نیاز به ۲ تا ۳ ماه وقت دارد. این زمان به‌منظور ترمیم فیستول و اتساع قسمت وریدی فیستول جهت استفاده از سوزن‌های درشت شماره ۱۶-۱۵-۱۴، مورد نیاز می‌باشد. در طی این مدت به بیماران آموزش داده می‌شوند که برای افزایش اندازه این عروق جهت وارد کردن سوزن‌های درشت در فیستول، ورزش نمایند (مثلاً فشردن یک توپ پلاستیکی برای فیستول ساعد). این دسترسی عروقی از زمان جاگذاری بیشترین عمر مفید را دارا می‌باشد، بنابراین بهترین گزینه برای بیمارانی که نیاز به همودیالیز مداوم دارند، می‌باشد(۷۸).

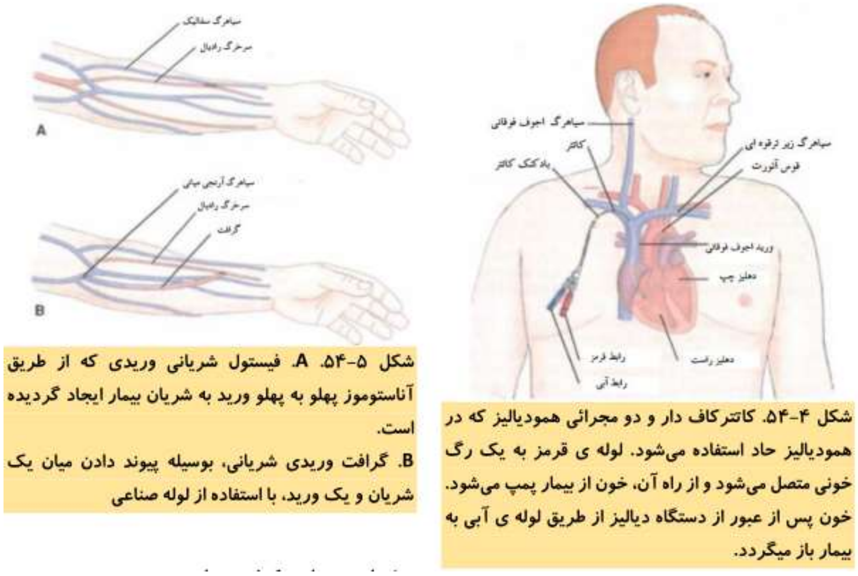

شکل ۲-۳. فیستول شریانی وریدی

۲-۱-۲-۷. عوارض دیالیز

اگرچه همودیالیز می‌تواند طول عمر بیمار را افزایش دهد، اما نه‌تنها نمی‌تواند طول عمر را در این بیماران به حد طبیعی برساند بلکه به برگشت کامل عملکرد کلیه نیز کمک نمی‌کند. بیمار در معرض مشکلات و عوارض متعددی قرار دارد. با آغاز دیالیز اختلال در متابولیسم چربی (هیپرتری‌گلیسریدمی) شدت یافته و منجر به عوارض قلبی-عروقی می‌گردد. نارسایی قلبی، بیماری کرونری قلب، آنژین، سکته و بیماری‌های عروق محیطی ممکن است روی داده و باعث ناتوانی بیمار شوند. یکی از عللی که منجر به مرگ در میان بیماران تحت همودیالیز مزمن می‌شود، بیماری قلبی عروقی می‌باشد. آنمی به علت کاهش تولید

گلبول‌های قرمز ناشی از کمبود هورمون اریتروپویتین و از دست دادن خون در طی همودیالیز در این بیماران دیده می‌شود.

همودیالیز، در کنار کاهش علائم بیماران و افزایش طول عمر آنها، یکسری عوارض و مشکلات برای این بیماران به همراه دارد. از جمله این عوارض می‌توان به کاهش فشارخون، کرامپ عضلانی، سندرم عدم تعادل، تهوع و استفراغ، تنگی نفس، سردرد، کم‌خونی، افزایش فشارخون، آنژین صدری، آریتمی، واکنش‌های آلرژیک، خستگی، کاهش اشتها، کاهش عملکرد جنسی، هیپوتانسیون، کرامپ عضلانی، درد قفسه سینه و پشت و خارش اشاره کرد (۱۰۵).

زخم‌های معده و سایر مشکلات گوارشی به علت وجود استرس فیزیولوژیک ناشی از بیماری مزمن مصرف برخی داروها و مشکلات همراه، ایجاد می‌شود. معمولاً بیماران با اورمی در شروع دیالیز احساس تهوع و مزه فلز در دهانشان را گزارش می‌کنند(۷۸).

در صورت شیفت سریع مایعات به دستگاه همودیالیز، فرد دچار استفراغ می‌شود و همین امر منجر به سوء تغذیه در این بیماران می‌شود. اختلال در متابولیسم کلسیم سبب، استودیستروفی کلیوی شده و این اختلال موجب ایجاد دردهای استخوانی و شکستگی می‌گردد. رسوب فسفر در پوست منجر به خارش می‌شود بیشتر بیمارانی که تحت همودیالیز قرار دارند، دچار مشکلات اساسی در خواب می‌شوند که سلامتی آنان به دلیل این مشکل بیشتر به خطر می‌افتد.

انجام دیالیز در اوایل صبح یا نزدیک شب می‌تواند به اختلال در خواب منجر گردد. سایر اختلالاتی که همراه با درمان دیالیز وجود دارد، عبارت‌اند از:

۱. در فواصل بین دوره‌های دیالیز به علت تجمع مایعات حملاتی از کوتاهی تنفس دیده می‌شود.
۲. هیپوتانسیون که ممکن است در حین درمان به علت خروج مایعات اتفاق بیافتد که علائم شایع آن شامل تهوع، استفراغ، تعریق، تاکیکاردی و خواب‌آلودگی است.
۳. کرامپ های دردناک عضلاتی در پایان دیالیز به علت خروج سریع مایع و الکترولیت‌ها از فضای خارج سلولی رخ می‌دهد.

۴. در صورت جدا شدن لاین های خونی و با جدا شدن سوزن دیالیز ممکن است خون زیادی از دست برود.

۵. دیس ریتمی‌های قلبی که حاصل تغییرات الکترولیت و PH یا خارج شدن داروهای ضد آریتمی در حین دیالیز هستند.

۶. آمبولی هوا که بندرت اتفاق می‌افتد و علت آن ورود هوا به سیستم عروقی بیمار است.

۷. درد قفسه سینه در بیماران مبتلا به بیماری‌های آترواسکلروتیک قلب، ایجاد می‌شود.

۸. سندروم عدم تعادل دیالیزی که در اثر انتقال مایع مغزی اتفاق می‌افتد و با علائمی نظیر سردرد، تهوع و استفراغ، بی‌قراری کاهش سطح هوشیاری یا تشنج مشخص می‌شود. معمولاً در نارسایی حاد کلیه و یا مواقعی که نیتروژن اوره خون[1] خیلی بالا باشد، (BUN> ۱۵۰) این وضعیت ایجاد می‌شود(۷۸).

بیماران نارسایی مزمن کلیوی همواره با تغییرات و بی‌ثباتی در وضعیت همودینامیک مواجه هستند(۴۸). بی‌ثباتی همودینامیک بیماران همودیالیز می‌تواند منجر به افت فشار داخل ریه و کاهش اثر همودیالیز به دلیل میزان فیلتراسیون ناکافی یا توقف زودرس درمان شود(۱۰۶). مهم‌ترین و شایع‌ترین اختلال و ناثباتی همودینامیک در طی همودیالیز بخصوص اولترافیلتراسیون، افت فشارخون یا هایپوتانسیون می‌باشد. تجمع سدیم و مایعات که ممکن است در طول زمان به دلیل عدم تعادل مایع مثبت مایع در بیماران دیالیزی رخ دهد، مسئول بار اضافی مایع خارج سلولی است که با اثرات نامطلوب و پیامدهای قلبی عروقی همراه است(۱۰۷). بار اضافی مایع خارج سلولی و مدیریت مایع ضعیف علت اساسی عوارض قلبی عروقی در بیماران همودیالیزی است(۱۰۸). وجود بیش از حد مایعات به‌خودی‌خود تأثیر مخرب مستقل و افزاینده‌ای بر فشارخون (اعم از فشارخون پایین یا بالا) در پیامدهای بیماران دیالیزی دارد و آن را افزایش می‌دهد(۱۰۹). از طرفی دیگر در جمعیت عمومی، اشباع اکسیژن شریانی یک شاخص مفید برای اکسیژناسیون شریانی و اندازه‌گیری معمول برای بسیاری از کاربردهای تشخیصی است. به طور مداوم، برای

[1] Blood urea nitrogen(BUN)

مطالعه آسیب‌های تنفسی در بیماران همودیالیز، اندازه‌گیری میزان اشباع اکسیژن نیز به طور قابل توجهی ارزشمند است(۱۱۰).

پرستار بخش دیالیز و مراقبتهای پرستاری، نقش بسیار مهمی در کنترل، حمایت، بررسی و آموزش به بیمار دارد. در طی دیالیز، وضعیت بیمار، دیالیزر و محلول دیالیز[۱] نیاز به کنترل مداوم دارند تا عوارض بالقوه متعددی از جمله آمبولی هوا، فراپالایش ناکافی یا بیش از حد (افت فشارخون، کرامپ، استفراغ)، نشت خون، آلودگی و لخته شدن مایع در حال چرخش و عوارض دسترسی عروقی ردیابی شوند. مراقبت از بیمار و حفظ و نگهداری وسایل و تجهیزات دسترسی عروقی تحت عنوان مراقبت از بیماران دیالیزی بستری آورده شده است(۷۸).

علاوه بر موارد ذکر شده، بیماران تحت همودیالیز به برخی اختلالات روانشناختی مانند افسردگی، خودکشی و دمانس یا فراموشی نیز دچار می‌شوند (۱۱۱). ساگلمبین[۲] و همکاران بیان داشتند که بعضی عوارض مانند خستگی به همراه سایر عوارض، پیامدهای متعدی از جمله اختلال عملکرد جنسی و کاهش کیفیت زندگی جنسی را در بیماران تحت همودیالیز موجب می‌شوند (۱۲). همچنین، انجام همودیالیز در یک وضعیت افقی و ماندن بدون فعالیت جسمانی بیمار موجب بوجود آمدن عوارضی همچون کاهش استقامت جسمی بدن، آتروفی عضلانی، ضعف عضلانی و کاهش قدرت می‌شود(۳۷). بنابراین، یکی از راه‌های کاهش عوارض همودیالیز، انجام تمرین ورزشی منظم در حین دیالیز است که باعث افزایش عملکرد جسمانی و کاهش آتروفی عضلانی می‌شود(۳۸).

۲-۱-۲-۸. کفایت دیالیز

همودیالیز یک درمان جایگزین کار کلیه در بیماران با نارسایی کلیوی است، لذا به هر اندازه بتواند این نقش را بهتر ایفاء کند، بیمار حال عمومی بهتری داشته، از عوارض نارسایی کلیه کمتر رنج برده، طول عمر بیشتری خواهد داشت. مشخص گردیده است بیمارانی که کفایت دیالیز آنها پائین است، طول عمر

[1] Dialysate
[2] Saglimbene

کوتاهتر و بیماران با کفایت دیالیز مطلوب و بالا، طول عمر برابر با بیماران پیوند کلیه دارند(۱۰۳). کفایت دیالیز تحت تأثیر سه عامل عمده می‌باشد:

۱) قابلیت[1] غشاء صافی در برداشت و انتقال مواد زائد خون: عبارت است از توانایی غشاء[2] در برداشت مواد محلول در خون که به‌صورت یک ضریب ثابت یا KOA[3] نشان داده می‌شود. هرچه KOA صافی بیشتر باشد، امکان کفایت دیالیز بهتر، بیشتر می‌شود.

۲) میزان جریان خون: با جریان خون بیشتر، امکان کفایت دیالیز بالاتر، بیشتر می‌شود.

۳) مدت زمان دیالیز: حداقل زمان هر جلسه دیالیز، ۴ ساعت می‌باشد؛ اما در حین دیالیز گاه به عللی، عملیات دیالیز متوقف می‌گردد که اگر بعداً به زمان دیالیز اضافه نشود، بیمار عملاً کمتر از ۴ ساعت دیالیز شده است(۱۰۳).

۲-۱-۲-۸-۱. روش‌های تعیین کفایت دیالیز

جهت بررسی کفایت دیالیز می‌توان از روشهای مختلفی استفاده کرد، از جمله این روشها: بررسی علائم بالینی، احساس رضایت از زندگی، هیپرتروفی بطن چپ، قدرت هدایت عصبی، متابولیسم مواد معدنی، فشارخون، کنترل حجم مایعات بدن و میزان برداشت اوره از بدن می‌باشند(۱۱۲, ۱۱۳).

جهت ارزیابی کفایت دیالیز، ارزیابی نشانه‌های بالینی بیماران ناکافی است. به خاطر اینکه تزریق اریتروپوئیتین اغلب نشانه‌هایی اورمی را حذف می‌کند(۱۱۴).

غلظت اوره در خون به راحتی قابل‌اندازه‌گیری است و تقریباً در تمام بدن به طور یکنواخت توزیع شده است. اما اندازه‌گیری و پایش مداوم میزان BUN کافی به نظر نمی‌رسد زیرا مقادیر پایین آن بیش از آنکه نشان دهنده برداشت کافی اوره توسط دیالیز باشد، بیشتر بیانگر وضعیت نامطلوب تغذیه و مصرف ناکافی پروتئین می‌باشد(۱۱۲, ۱۱۳). لذا به نظر می‌رسد بهترین شاخص، سنجش میزان برداشت اوره باشد(۱۰۳).

[1] eficiency
[2] membrane
[3] coefficient transfer mass

بررسی کفایت دیالیز با محاسبه R یا URR یا (KT/V)میسر می‌گردد. محاسبه فرمول (KT/V) هدف اصلی در دیالیز، برداشت بیشتر مواد زائد و توکسین هایی است که در اورمی وجود دارد لذا اگر در هر جلسه دیالیز، میزان برداشت مواد زائد بیشتر باشد، کفایت دیالیز و شاخص (KT/V) بهتر شده است.

محاسبه (KT/V[1]):

(KT/V) **شامل سه چیز است:**

الف: K (کلرانس[2] آب خون): کلرانس عبارت است از میزانی از حجم خون که در واحد زمان از یک ماده پاک شده است. پس از عبور خون از صافی، بخشی از مواد زائد آن برداشته می‌شود. کلرانس خود تحت تأثیر سه عامل میزان جریان خون، قابلیت صافی و میزان جریان مایع دیالیز می‌باشد.

$$\boldsymbol{k} = \frac{\text{اوره قبل از صافی} - \text{اوره پس از صافی}}{\text{اوره قبل از صافی}} * \text{(در قیقه) میزان جریان خون}$$

اهمیت کلیرانس در این است که کار انجام شده توسط صافی را بیان می‌کند؛ اما در حالتی که فقط به میزان کاهش غلظت اوره نگاه شود، مشخص نیست چقدر کار انجام شده است، چرا که اگر میزان جریان خون صافی خیلی کم باشد (جریان خون آهسته باشد) غلظت اوره، در صافی کاهش بیشتری پیدا می‌کند، زیرا زمان برای انتقال اوره از خون به مایع دیالیز بیشتر است، اما در نهایت، کل اورهٔ برداشت شده ممکن است ناچیز باشد.

ب) T: زمان جلسه دیالیز است.

ج) V: عبارت است از حجم انتشار اوره در بدن که حدوداً ۵۵ درصد وزن پس از دیالیز است (یعنی تقریباً برابر با حجم آب بدن که حدود ۶۰ درصد است)

محاسبه R یا URR: نسبت اوره پس از دیالیز به اوره قبل از دیالیز که با R نشان داده می‌شود رابطه مستقیم (و معکوس) با (KT/V) دارد.

$$\text{R} = \frac{\text{اوره پس از دیالیز}}{\text{اوره قبل از دیالیز}}$$

[1] urea kinetic modeling
[2] Clearance

مشخص است که هر چه نسبت R کوچک‌تر باشد، یعنی اوره بیشتری از بیمار در طی دیالیز برداشت شده، سطح اورهٔ پس از دیالیز کاهش یافته کفایت دیالیز بهتر است. این نسبت R، به شکل دیگری نیز نشان داده می‌شود یعنی R-۱ که با URR[1] نمایش داده می‌شود. هر چه URR بزرگ‌تر باشد، کفایت دیالیز بهتر است(۱۰۳).

کفایت دیالیز یک شاخص مهم بقا در بیماران مبتلا به همودیالیز مزمن است دستورالعمل‌های اخیر توصیه می‌کند که دوز دیالیز باید با استفاده از آزمایش خون حداقل یک‌بار در ماه تنظیم شود و پیشنهاد می‌کند که یک واحدKt/V ۱/۴ در هر جلسه همودیالیز برای بیماران تحت درمان سه بار در هفته قرار گیرد(۲۱).

اگر همودیالیز از کفایت لازم برخوردار نباشد سطح سموم خونی و علائم بالینی بیمار به‌خوبی کنترل نشده و بنابراین میزان ناتوانی و مرگ‌ومیر افزایش می‌یابد. همچنین دیالیز ناکافی می‌تواند موج افزایش عوارض بیماری، طول مدت بستری شدن و هزینه‌های تحمیلی بر بیماران شود(۱۱۵).

۲-۱-۲-۸-۲. عوامل تأثیرگذار بر کفایت دیالیز

۱- تعداد جلسات هفتگی دیالیز: انجام سه جلسه دیالیز در هفته به عنوان دیالیز استاندارد محسوب می‌شود(۱۰۳).

۲- افزایش مدت زمان دیالیز: افزایش مدت زمان دیالیز منجر به بهبود کفایت دیالیز خواهد شد. حداقل زمان انجام دیالیز ۴ ساعت در هر جلسه می‌باشد و اگر به هر دلیلی دیالیز متوقف شود باید به زمان دیالیز اضافه شود(۱۰۳).

۳- نوع بافر: استفاده از بافر بیکربنات یکی از مداخلات ممکن در ارتقاء کیفیت دیالیز می‌باشد(۱۱۴).

۴- نوع صافی: جهت دستیابی به یک دیالیز مؤثر انتخاب صافی بسیار اهمیت دارد و همیشه انتخاب صافی‌های High Flux وHigh Efficiency و بزرگتر منجر به بهبود کفایت دیالیز نخواهد شد، چرا که در افراد با جثه کوچک این صافیها منجر به بروز عوارض حین دیالیز خواهند شد. لذا انتخاب

[1] Urea reduction rate

صافی بر اساس شرایط بیمار می‌باشد. همچنین نحوه آماده کردن صافی و عدم وجود هوا در صافی دارای اهمیت می‌باشد. وجود هوا در صافی باعث ایجاد فضای مرده شده و با ایجاد لخته در آن فضا، از کلیرانس مواد کاسته خواهد شد(۱۱۶).

۵- میزان جریان خون: میزان جریان خون با میزان پاک شدن آن از یک ماده رابطه مستقیم دارد و افزایش میزان جریان خون باعث افزایش میزان پاک‌شدگی خون از یک ماده خاص خواهد شد. افزایش جریان خون تا ۲۵ درصد در افزایش کفایت دیالیز مؤثر است(۲۰).

۶- اثر افزایش جریان محلول: دیالیز میزان انتشار اوره را از خون به محلول دیالیز افزایش می‌دهد. البته این اثر خیلی زیاد نیست. جریان محلول ۵۰۰ میلی لیتر در دقیقه است(۲۰).

از علل دیگر می‌توان دلایل زیر را ذکر کرد:

۱- تجویز ناکافی دیالیز به علت اشتباه در محاسبات.

۲- به‌کارگیری صافی نامناسب و کوچک.

۳- کم بودن میزان کارایی صافی نسبت به آنچه کارخانه ادعا کرده است

۴- کم بودن سرعت گردش خون به دلیل عدم کارایی فیستول یا عدم تحمل بیمار

۵- کم بودن زمان دیالیز به علت عدم تحمل یا همکاری بیمار

۶- قطع کردن یا کم کردن سرعت دیالیز به دلیل عوارض حین دیالیز مانند: افت فشارخون، کرامپ عضلانی و درد قفسه سینه.

۷- عدم کفایت دسترسی عروقی یا تعبیه نامناسب سوزن فیستول

۸- عدم رعایت رژیم غذایی توصیه شده(۱۰۳, ۱۱۷, ۱۱۸).

لذا افزایش کفایت دیالیز علاوه بر فاکتورهای مربوط به دستگاه و بخش همودیالیز به عواملی مرتبط با بیمار نیز بستگی دارد، عواملی مانند: رعایت رژیمهای غذایی، دارویی و محافظت از دسترسی عروقی، که بیمار با رعایت این موارد در انجام دیالیز مؤثر در زمان تعیین شده با کنترل عوارض و جریان خون مناسب

نقش دارد. یکی دیگر از مداخلاتی که توسط بیمار قابل انجام می‌باشد و می‌تواند در افزایش کفایت دیالیز و از بین بردن عوارض دیالیز مؤثر می‌باشد، انجام فعالیت فیزیکی می‌باشد(۱۱۹).

۲-۱-۳. فعالیت فیزیکی

۲-۱-۳-۱. تعریف فعالیت فیزیکی

فعالیت فیزیکی به هرگونه فعالیت یا حرکت بدن که در اثر انقباض و انبساط عضلات اسکلتی ایجاد شده و نیازمند صرف انرژی است، گفته می‌شود(۱۲۰). اصطلاح «فعالیت فیزیکی» را نباید با «ورزش» اشتباه گرفت، ورزش، زیر مجموعه‌ای از فعالیت فیزیکی است که برنامه‌ریزی شده، ساختار یافته، تکراری است و هدف آن بهبود یا حفظ یک یا چند فاکتور آمادگی جسمانی است. فراتر از ورزش، هر فعالیت فیزیکی دیگری که در طول اوقات فراغت، برای گشت‌وگذار و یا به عنوان بخشی از کار فرد انجام شود، دارای یک مزیت سلامتی است. به علاوه، فعالیت بدنی با شدت متوسط و شدید باعث بهبود سلامت می‌شود (۱۲۱). مقدار فعالیت بدنی توصیه شده توسط سازمان بهداشت جهانی عبارت است:

کودکان و نوجوانان ۵-۱۷ ساله: حداقل باید روزانه ۶۰ دقیقه فعالیت بدنی با شدت متوسط تا شدید انجام دهند. فعالیت بدنی با مقادیر بیشتر از ۶۰ دقیقه در روز، مزایای بیشتری برای سلامتی فراهم می‌کند و باید حداقل ۳ بار در هفته شامل فعالیت‌هایی باشد که عضلات و استخوان را تقویت می‌کنند (۱۲۱).

بزرگسالان ۱۸ تا ۶۴ ساله: باید حداقل ۱۵۰ دقیقه فعالیت بدنی با شدت متوسط در طول هفته، یا حداقل ۷۵ دقیقه فعالیت بدنی با شدت زیاد در طول هفته یا ترکیبی معادل از فعالیت با شدت متوسط و شدید را انجام دهند. برای مزایای اضافی سلامتی، بزرگسالان باید فعالیت بدنی با شدت متوسط را به ۳۰۰ دقیقه در هفته یا معادل آن برسانند. فعالیت‌های تقویت عضله باید شامل گروه‌های اصلی عضلانی در ۲ یا بیشتر از هفته باشد.

بزرگسالان ۶۵ سال به بالا: باید حداقل ۱۵۰ دقیقه فعالیت بدنی با شدت متوسط در طول هفته یا حداقل ۷۵ دقیقه فعالیت بدنی با شدت زیاد در طول هفته یا ترکیبی معادل از فعالیت با شدت متوسط و شدید انجام دهند. برای مزایای اضافی سلامتی، آن‌ها باید فعالیت بدنی با شدت متوسط را به ۳۰۰ دقیقه در هفته

یا معادل آن افزایش دهند. کسانی که تحرک ضعیفی دارند، باید سه روز یا بیشتر در هفته برای تقویت تعادل و جلوگیری از زمین خوردن فعالیت بدنی انجام دهند. فعالیت‌های تقویت عضله باید شامل گروه‌های اصلی عضلانی، دو یا بیشتر از هفته باشد. شدت اشکال مختلف فعالیت بدنی در افراد متفاوت است. برای اینکه برای سلامت قلب و تنفس مفید باشد، تمام فعالیت‌ها باید در دوره‌هایی با حداقل ده دقیقه انجام شود (۱۲۱).

۲-۱-۳-۲. فواید فعالیت بدنی و وضعیت فعالیت بدنی در دنیا

فعالیت بدنی منظم با شدت متوسط - مانند پیاده‌روی، دوچرخه‌سواری یا ورزش، فواید قابل توجهی برای سلامتی دارد. در تمام سنین، مزایای فعالیت بدنی بیشتر از آسیب احتمالی به عنوان مثال در اثر تصادفات است. برخی از فعالیت‌های بدنی بهتر از انجام هیچ کاری است. با فعال‌تر شدن در طول روز به روش‌های نسبتاً ساده، افراد به راحتی می‌توانند به سطح فعالیت توصیه شده برسند. مزایای سطح منظم و کافی فعالیت بدنی به شرح ذیل می‌باشد:

۱- تناسب‌اندام عضلانی و بهبود عملکرد قلبی تنفسی

۲- بهبود استخوان و سلامت عملکرد

۳- کاهش خطر فشارخون بالا، بیماری عروق کرونر قلب، سکته مغزی، دیابت، انواع مختلف سرطان (از جمله سرطان پستان و سرطان روده بزرگ) و افسردگی

۴- کاهش خطر سقوط و همچنین شکستگی مفصل ران یا مهره‌ها

۵- تعادل انرژی و کنترل وزن

فعالیت بدنی ناکافی یکی از مهم‌ترین عوامل خطر در مرگ‌ومیر در جهان است و در بسیاری از کشورها در حال افزایش است که بر بار بیماری‌های غیرواگیر[1] افزوده و بر سلامت عمومی در سراسر جهان تأثیر می‌گذارد. افرادی که فعالیت کافی ندارند، در مقایسه با افرادی که فعالیت کافی دارند، ۲۰ تا ۳۰ درصد خطر مرگ دارند. میزان فعالیت بدنی ناکافی در سطح جهان، حدود ۲۳ درصد از بزرگسالان ۱۸ ساله

[1] Non-Communicable Diseases

و بالاتر در سال ۲۰۱۰ به‌اندازه کافی فعال نبودند (مردان ۲۰ درصد و زنان ۲۷ درصد) در کشورهای با درآمد بالا، ۲۶ درصد از مردان و ۳۵ درصد از زنان از نظر جسمی فعالیت کافی نداشتند، در حالی که در کشورهای کم‌درآمد ۱۲ درصد از مردان و ۲۴ درصد از زنان از نظر جسمی فعال نبودند. سطح فعالیت‌های بدنی کم یا کاهش یافته اغلب با تولید ناخالص ملی بالا یا در حال افزایش مطابقت دارد. کاهش فعالیت بدنی تا حدودی به دلیل عدم فعالیت در اوقات فراغت و رفتار کم تحرک در محل کار و خانه است. به همین ترتیب، افزایش استفاده از حالت‌های «غیرفعال» حمل‌ونقل نیز به فعالیت بدنی ناکافی کمک می‌کند. در سطح جهانی، ۸۱ درصد از نوجوانان ۱۱-۱۷ ساله از نظر جسمی در سال ۲۰۱۰ فعالیت کافی نداشتند. دختران نوجوان نسبت به پسران نوجوان فعالیت کمتری داشتند، ۸۴ درصد در مقابل ۷۸ درصد، توصیه‌های سازمان بهداشت جهانی را رعایت نمی‌کردند (۱۲۱).

پیروی از توصیه‌ها و داشتن حداقل ۱۵۰ دقیقه فعالیت هوازی در هفته با شدت متوسط، می‌تواند شانس خطر برای بیماران قلبی و سکته مغزی را کاهش دهد. فعالیت بدنی منظم همچنین می‌تواند باعث کاهش فشارخون و بهبود سطح کلسترول شود. بررسی‌های سیستماتیک اخیر نشان داد که بین فعالیت بدنی و وضعیت سلامتی رابطه دوز-پاسخ وجود دارد. این رابطه عموماً منحنی است و بیشترین مزایای مربوط به‌سلامتی در حجم فعالیت‌های بدنی نسبتاً کم رخ می‌دهد. اکثر راهنمایی‌های بین‌المللی، ۱۵۰ دقیقه در هفته فعالیت بدنی را با شدت متوسط را توصیه می‌کنند (۱۲۲).

۲-۱-۳-۳. ورزش و فعالیت فیزیکی در بیماران تحت دیالیز

ورزش و فعالیت فیزیکی می‌تواند به عنوان یک جنبه مهم از درمان در تمام مراحل نارسایی مزمن کلیه به شمار آید. فعالیت ورزشی در بیماران نارسایی مزمن کلیه به طور مشخص کمتر از افراد سالم است. مکانیسم‌های مسئول کاهش ظرفیت فیزیکی در این جمعیت بیمار به طور کامل روشن نشده است. در حالی که عواملی مانند کم‌خونی و التهاب ممکن است نقش داشته باشند، اما اختلال عملکرد فیزیکی مشاهده شده با کاهش عملکرد کلیه مستقل از سن، کم‌خونی و بیماری همراه است(۱۲۳, ۱۲۴). کاهش

در ظرفیت فیزیکی نه تنها به کاهش کیفیت زندگی منجر می‌شود(۱۲۵) بلکه خطر مرگ‌ومیر را در تمام مراحل CKD افزایش می‌دهد(۱۲۶).

ورزش را می‌توان هرگونه فعالیت بدنی شامل پیاده‌روی، کوه‌نوردی، پله نوردی و غیره که توسط بیماران همودیالیزی انجام می‌شود و می‌تواند آمادگی جسمانی و ظرفیت هوازی را بهبود بخشد، تعریف کرد(۱۲۷). ورزش برای سلامت جسمانی بیماران دیالیزی مفید است(۱۲۸) و عملکرد قلب و عروق، فشارخون، قدرت ماهیچه‌ها، وضعیت تغذیه‌ای و کیفیت دیالیز را بهبود می‌بخشد. همچنین احساسات منفی مانند اضطراب و افسردگی را کاهش می‌دهد، آن‌ها را بهتر می‌کند و تعامل اجتماعی بیماران و خانواده‌های آن‌ها را بهبود می‌بخشد(۱۲۹, ۱۳۰)؛ بنابراین، ارزیابی فعالیت بدنی بیماران تحت همودیالیز و تشویق آن‌ها برای انجام ورزش در زندگی ضروری است.

بسیاری از مطالعات، نتایج بهتری را در بیماران مبتلا به CKD با فعالیت بدنی بیشتر مشاهده کرده‌اند.

علاوه بر این، بررسی‌های منظم سیستماتیک اثرات مفید مداخله ورزشی بر تحمل ورزش، توانایی بدنی و کیفیت زندگی در بیماران دیالیزی را نشان داده است، اگرچه تأثیر مفید بر مرگ‌ومیر کلی نامشخص است(۱۳۱).

اکثر بیماران تحت همودیالیز تصور مثبتی از ورزش دارند، اما اکثر آنها ورزش نمی‌کنند. این می‌تواند منجر به خستگی، خستگی عضلانی و ترس از آسیب فیستول شریانی شود(۱۳۲).

۲-۱-۴. خستگی

۲-۱-۴-۱. تعریف خستگی

خستگی مفهومی است که دارای تعاریف و برداشت‌های متفاوتی می‌باشد، برخی از آن به عنوان فروماندگی یا ناتوانی و یک احساس ناخوشایند و شکایت ذهنی از کسالت ذکر می‌کنند که با فقدان انرژی ناشی از بی‌انگیزه بودن و خواب‌آلودگی متفاوت است (۱۳۳). خستگی را می‌توان به عنوان فقدان انرژی فیزیکی یا ذهنی و شناختی در نظر گرفت که منجر به افت فعالیت‌های فرد می‌شود (۱۳۴). خستگی یک

پدیده پیچیده است که شامل جنبه‌های بسیاری از وجود مسائل جسمی و روحی و عاطفی تعیین می‌شود که می‌تواند به عنوان یک وضـــعیت که فرد دچار ناراحتی و کاهش توانایی عملکرد به دلیل کمبود انرژی می‌شـود، توصـیف شـود (۵۶). اخیراً خســتگی را عارضـه‌ای پویا تعریف می‌کنند که می‌تواند در مواقع یا شـرایط مختلفی بروز کرده و یا ناپدید شود. در برخی افراد این عوارض خفیف و آزاردهنده نیست، اما در برخی دیگر کاملاً انرژی فرد را تحلیل می‌برد (۱۳۵). انجمن تشخیص پرستاری آمریکا، تشخیص پرستاری خســتگی را به صـورت احســاس ضـعف، بی‌حالی، فرسودگی، نداشتن انرژی و کاهش ظرفیت برای انجام فعالیت‌های فکری و فیزیکی تعریف کرده است (۸۱).

۲-۱-۴-۲. انواع خستگی

خستگی می‌تواند به جسمی یا روانی، حاد یا مزمن دسته‌بندی شود:

۱- خستگی فیزیکی یا جسمی: یک ناتوانی گذرا از ماهیچه است که به خاطر نگه‌داشتن سطح بهینه عملکرد است (۱۳۶).

۲- خستگی ذهنی: هرگونه خستگی جسمی نمود ذهنی هم دارد و اغلب این دو به هم مرتبط‌اند. این خستگی نوعی رکود در عملکرد شناختی است که در نتیجه یک دوره‌ی طولانی از فعالیت شناختی پیش می‌آید. شروع خستگی ذهنی و روانی در خلال هر فعالیت شناختی تدریجی است و به ظرفیت شناختی افراد وابسته است. اگرچه تأثیر خستگی ذهنی بر عملکردها و مهارت‌های شناختی مشاهده شده است؛ اما هنوز تأثیر آن بر عملکردهای فیزیکی مورد تحقیق قرار نگرفته است. خستگی روانی می‌توانند با کاهش عملکرد فیزیکی نشان داده شود (۱۳۷).

۳- خستگی حاد: معمولاً از یک بی‌توجهی مشخص به سلامت فرد ناشی می‌شود که با استراحت از بین نمی‌رود.

۴- خستگی مزمن: به طور معمول خستگی،که شش ماه متوالی یا بیشتر به درازا می‌کشد و با استراحت نیز از بین نمی‌رود (۱۳۸).

۲-۱-۴-۳. خستگی در بیماران همودیالیز

خستگی و فقدان انرژی یکی از نشانه‌های شایع و زجرآور در بیماران همودیالیزی می‌باشد که ماهیت مزمن و ناتوان‌کننده آن باعث کاهش فعالیت‌های مربوط به مراقبت از خود، محدودیت ایفای نقش و کاهش توانایی فرد برای انجام فعالیت‌های روزانه زندگی می‌گردد(۱۳۹).

شیوع خستگی از ۱۷ تا ۳۰٪ در بیماران متغیر است و به معنای ناتوانی برای انجام فعالیت‌های فیزیکی است. احساسی شبیه بیزاری، بی‌رمقی، بی‌حوصلگی از درد عضلانی در شخص مشهود شده که ناشی از وضعیت‌های دشواری است که نیازمند قابلیت‌هایی بیش از توانایی فرد است(۱۴۰).

علل خستگی در بیمارانی که همودیالیز دریافت می‌کنند بسیار پیچیده و چندعاملی است و ممکن است شامل اورمی، کم‌خونی، افسردگی و بی‌حسی جسمی باشد(۱۴۱). همچنین ماهیت مزمن، تهاجمی و سنگین دیالیز ممکن است به معنای آن باشد که خستگی بیمارانی که همودیالیز را تجربه می‌کنند در مقایسه با سایر بیماری‌ها متمایز است(۱۴۲).

فاکتورهایی فیزیولوژیکی شامل آنمی، سن و سایز بدن، سوءتغذیه و اورمی، بالا بودن سطح کلسترول خون و عوامل دیگر همچون افسردگی، عوامل رفتاری، عوامل مرتبط با درمان و خصوصیات شخصی افراد از علت‌های وجود خستگی در این بیماران است(۱۴۳, ۱۴۴).

جکبسون[1] و همکاران در یک مطالعه مرور سیستماتیک چهار موضوع مربوط به خستگی را در بیماران همودیالیز شناسایی کردند(۱۴۵):

۱. ناتوان و خسته‌کنندگی بار[2] دیالیز (کاهش وزن بدن، گرفتار در چرخه نادرست از خستگی پس از دیالیز، بی‌خوابی و نگرانی مانع استراحت، رژیم خسته‌کننده و آزاردهنده و بدون درمان و تسکین)

۲. زندگی محدود (محروم از وقت، مدیریت ذخایر انرژی، نیاز ناامیدکننده به استراحت و شادی پیش رو).

[1] Jacobson
[2] burden

۳. کاهش ظرفیت برای تحقق نقش‌ها و روابط (از دست دادن توانایی کار و تأمین خانواده، عدم موفقیت در نقش والدین، عدم استقامت در رابطه جنسی و تکیه بر دیگران).

۴. در برابر سوءتفاهم آسیب‌پذیر هستند (به دلیل نیاز به استراحت و عدم تحقق انتظارات موردانتقاد قرار می‌گیرند).

۲-۲. مروری بر متون

در این بخش از پایان‌نامه به بررسی و معرفی مطالعات داخلی و خارجی انجام شده در رابطه با فعالیت فیزیکی حین دیالیز بر کفایت دیالیز، شاخص‌های همودینامیک و خستگی در بیماران تحت همودیالیز پرداخته می‌شود. از کلیدواژه‌های نارسایی کلیه، همودیالیز، فعالیت فیزیکی، خستگی و کفایت دیالیز و شاخص‌های همودینامیک و معادل انگلیسی این واژه‌ها جهت جستجوی پایگاه‌های اطلاعاتی SID, Irandoc, ScienceDirect, PubMed, Google Scholar, Scopusاستفاده شد. به منظور دستیابی به مقالات پایگاه‌های فوق در محدوده ده سال اخیر (از سال ۲۰۰۵ تا ۲۰۲۱) مورد بررسی قرار گرفتند. در این بخش متون مرتبط به ترتیب از جدید به قدیم گزارش گردیده است.

۲-۲-۱. مطالعات داخلی

صالحی و همکاران در سال ۱۳۹۹ مطالعه‌ای با هدف بررسی تأثیر ورزش بر خستگی بیماران همودیالیزی انجام دادند. در این مطالعه تجربی، سی‌وهفت بیمار همودیالیزی در این مطالعه شرکت کردند. بیماران به طور تصادفی به دو گروه مداخله (۲۰ نفر) و گروه کنترل (۱۷ نفر) تقسیم شدند. شرکت‌کنندگان در گروه مداخله دو بار در هفته به مدت ۳ ماه به مدت ۲۰ دقیقه با دوچرخه کوچک ورزش کردند. خستگی بیماران در طول و بعد از مداخله چهار بار اندازه‌گیری شد. برای اندازه‌گیری میزان خستگی از پرسشنامه خستگی چند بعدی استفاده شد. نتایج نشان داد که توان‌بخشی از طریق ورزش با استفاده از مینی دوچرخه تأثیر بسزایی در جلوگیری از افزایش خستگی بیشتر در بیماران همودیالیزی دارد و باعث می‌شود که دوچرخه مینی دوچرخه به عنوان یک مداخله مؤثر غیردارویی مؤثر باشد که از افزایش خستگی بیماران تحت همودیالیز جلوگیری می‌کند(۱۴۶).

نصیری زاده و همکاران در سال ۱۳۹۸ مطالعه‌ای با هدف بررسی تأثیر تکنیک آرام‌سازی عضلانی بر تهوع و کفایت دیالیز بیماران تحت درمان با همودیالیز انجام دادند. در این مطالعه‌ی تجربی ۴۵ بیمار همودیالیزی در گروه کنترل و ۴۴ بیمار در گروه مداخله از بیمارستان‌های گناباد و زاهدان در سال‌های ۹۴-۱۳۹۳ در آن شرکت داشتند. نمونه‌ها به صورت تخصیص تصادفی در دو گروه کنترل و مداخله قرار گرفتند. تکنیک آرام‌سازی عضلانی به گروه آزمون آموزش داده و اجرا شد. نتایج حاصل با نرم‌افزار SPSS نسخه ۲۱ و با استفاده از آزمون‌های آماری کای اسکوئر، تی مستقل و من ویتنی در سطح معنی‌داری کمتر از ۰/۰۵ مورد تجزیه و تحلیل قرار گرفت. یافته‌ها نشان داد که اختلاف میانگین بین کفایت دیالیز بعد از مداخله در گروه آزمون، با توجه به آزمون آماری تی زوجی معنادار بود. سطح کفایت دیالیز در گروه آزمون قبل مداخله ۰/۳۹±۱/۳۵ و بعد مداخله و ۰/۴۳±۱/۵۰ بود. نتیجه آزمون آماری ویلکاکسون درخصوص مقایسه شدت تهوع قبل (۱/۰۴±۰/۲۲) و بعد از مداخله در گروه آزمون تفاوت آماری (۰/۱۵±۰/۰۲) معناداری نشان نداد. با توجه به نتایج این مطالعه، می‌توان بیان کرد که آموزش و اجرای تکنیک آرام‌سازی عضلانی بنسون باعث کاهش میزان تهوع و افزایش کفایت دیالیز بیماران تحت درمان با همودیالیز می‌شود(۱۴۷).

شایانی ممتاز و همکاران در سال ۱۳۹۷ مطالعه‌ای از نوع شبه تجربی با هدف اثربخشی برنامه توان‌بخشی بر خستگی بیماران تحت همودیالیز انجام دادند. در آن از طرح پیش‌آزمون- پس‌آزمون با گروه کنترل استفاده شد. از طریق روش نمونه‌گیری مبتنی بر هدف و بر اساس معیارهای ورود ۴۰ نفر انتخاب و به صورت تصادفی با استفاده از جدول اعداد تصادفی در دو گروه مداخله و کنترل قرار گرفتند. برای گروه مداخله اجرای برنامه‌ی توان‌بخشی شامل انجام ماساژ کف پا در حین همودیالیز به مدت ۱۰ دقیقه در ۱۲ جلسه حین همودیالیز به طول مدت یک ماه همراه با آموزش الگوی فعالیت روزانه، آموزش رژیم غذایی و داروها با حضور خانواده مددجو انجام شد. نمونه‌ها قبل و یک ماه بعد از مداخله، پرسشنامه‌ی خستگی MFI-20 را تکمیل کردند. داده‌ها با نرم‌افزار آماری SPSS19 و با استفاده از آزمون‌های آماری کای اسکوئر، دقیق فیشر، تی مستقل و زوجی و کوواریانس تجزیه‌وتحلیل شدند. نتایج نشان داد که بین دو گروه مداخله

و کنترل از نظر توزیع متغیرهای زمینه‌ای تفاوتی وجود نداشت. میانگین میزان خستگی قبل از مداخله در گروه مداخله (۵/۵۴ ± ۵۶/۶۸) و در گروه کنترل (۴/۵۲ ± ۵۵/۷۲) بود که بعد از مداخله در گروه مداخله به (۴/۲۶ ± ۴۹/۲۵) و در گروه کنترل به (۴/۶۶ ± ۵۵/۱۱) رسید و این اختلاف در گروه مداخله معنی‌دار بود. **با** توجه به نتایج به دست آمده می‌توان ذکر نمود که اجرای برنامه توان‌بخشی در کاهش خستگی بیماران همودیالیزی مؤثر است؛ بنابراین می‌توان روش به کار گرفته شده در پژوهش حاضر را به عنوان یک روش ساده، غیرتهاجمی، کم‌هزینه و مؤثر در کاهش خستگی بیماران همودیالیزی به پرستاران پیشنهاد نمود(۱۴۸).

شفیع پور در سال ۱۳۹۶(۲۰۱۷)، مطالعه‌ای روی ۶۰ بیمار دیالیزی به صورت تصادفی از دو بیمارستان در استان مازندران با عنوان بررسی تأثیر تمرینات ورزشی حین دیالیز و پیاده‌روی در منزل بر توانایی جسمانی و کفایت دیالیز بیماران همودیالیزی، انجام داد. این مطالعه روی بیماران مزمن بیش از ۳ ماه دیالیز و با مداخله دو هفته فعالیت حین دیالیز در ابتدای همودیالیز و ۶ هفته پیاده‌روی در منزل انجام شد. در نتایج حاصله در این پژوهش، کفایت دیالیز در بیماران گروه مداخله نسبت به گروه کنترل افزایش نشان داد هرچند از نظر آماری این تغییرات معنی‌دار نبود. شفیع پور، انجام برنامه فعالیت طراحی‌شده حین دیالیز را جهت بالا بردن وضعیت جسمانی و کفایت دیالیز بیماران همودیالیزی توصیه می‌کند(۷۴).

در سال ۲۰۱۶ یک مطالعه مقطعی، با عنوان کفایت دیالیز، پاکسازی صافی و راهکارها برای رسیدن به هدف در سطح کل کشور انجام داد که طی آن حدود ۲۰۰۹ نفر از بیماران تحت همودیالیز در سطح کشور موردمطالعه و بررسی قرار گرفتند. در این پژوهش عوامل زیادی مانند دسترسی‌های عروقی، سرعت جریان خون، ضریب صافی‌ها و Kt/V مورد بررسی قرار گرفتند که در نتایج حاصله، بیماران به کفایت دیالیز مناسب دست نمی‌یافتند. از این‌رو برای دستیابی به کیفیت استاندارد و در حد جهانی نیاز به انتخاب صافی‌های مناسب، گردش خون مناسب و دسترسی عروقی با کیفیت و یا افزایش تعداد جلسات می‌باشد که در برخی موارد رفع موانع از دست دیالیز کاران و مراقبین بهداشتی خارج می‌باشد؛ بنابراین توصیه به آموزش بهتر پرستاران و انجام راهکارهایی جهت بالا بردن کیفیت دیالیز شد(۹).

بصیری مقدم و همکاران در سال ۲۰۱۶ تأثیر حرکات توأم ایزوتونیک و ایزومتریک حین همودیالیز بر میزان کفایت دیالیز بیماران همودیالیزی را بررسی کردند. در این کارآزمایی بالینی، ۵۰ بیمار همودیالیزی در دو مرکز همودیالیز شهرستان گناباد و قاین در سال ۱۳۹۳ به‌روش نمونه‌گیری تصادفی ساده و با درنظرگرفتن معیارهای ورود به مطالعه انتخاب و مورد بررسی قرار گرفتند. این بیماران به‌صورت تصادفی ساده به دو گروه مساوی آزمون و شاهد تقسیم شدند. بیماران گروه آزمون تمرینات توأم ایزوتونیک و ایزومتریک را هماهنگ با فیلم آموزشی به مدت ۶۰ دقیقه در ۲ ساعت اول همودیالیز انجام دادند. این برنامه ورزشی سه بار در هفته به مدت ۶ هفته انجام شد. میزان کفایت دیالیز، قبل و بعد از مداخله در هر دو گروه محاسبه شد. داده‌ها با نرم‌افزار SPSS 14.5 و آزمون‌های T مستقل، مجذور کای و ضریب همبستگی پیرسون و اسپیرمن مورد تحلیل قرار گرفت. یافته‌ها نشان داد که پس از مداخله، از نظر کفایت دیالیز تفاوت آماری معنی‌داری در دو گروه وجود داشت (p=۰/۰۱)، به‌طوری که در گروه آزمون قبل از مداخله، ۸۰ درصد بیماران کفایت دیالیز کمتر از ۱/۲ داشتند که پس از مداخله به ۴۰ درصد رسید. در صورتی که در گروه شاهد تعداد افرادی که کفایت دیالیز کمتر از ۱/۲ داشتند، از ۷۲درصد به ۷۶ درصد افزایش یافت. لذا انجام حرکات توأم ایزوتونیک و ایزومتریک حین همودیالیز میزان کفایت دیالیز بیماران همودیالیزی را بهبود می‌بخشد(۱۴۹).

ریاحی در سال ۱۳۹۱ یک مطالعه نیمه تجربی با عنوان تأثیر تمرینات ورزشی منظم بر کیفیت دیالیز، آتروفی عضلانی و عملکرد جسمانی بیماران همودیالیزی در حین دیالیز در بیمارستان شریعتی اصفهان از بین ۳۰ نفر از بیماران با نارسایی مزمن کلیوی تحت درمان با همودیالیز بالاتر از ۳ سال انجام داد. بیماران ۳ جلسه در هفته و هر جلسه ۴ ساعت دیالیز می‌شدند. مداخله به صورت ۲۰ هفته تمرینات ورزشی با استفاده از دوچرخه در حین دیالیز انجام شد. نمونه‌گیری به صورت هدفمند و در دسترس انجام شد و به طور تصادفی به دو گروه مداخله و کنترل تقسیم شدند. تمرینات در ۲ ساعت ابتدایی دیالیز انجام گردید. در نتیجه حاصله از این پژوهش تفاوت معناداری در کفایت دیالیز دیده نشد(۷۳).

در پژوهشی توصیفی که قربانی مقدم به همراه سایر همکاران در سال ۱۳۹۳ روی ۹۳ بیمار همودیالیزی مراجعه‌کننده به بخش همودیالیز بیمارستان شهدای خلیج‌فارس بوشهر با هدف بررسی و تعیین میزان کفایت دیالیز در بیماران تحت درمان با همودیالیز در این بیمارستان پرداخت. محقق به این نتیجه دست یافت که بیش از ۴۰ درصد بیماران به Kt/V حداقل ۱/۲ دست نمی‌یابند و کفایتی کمتر از آن دارند و تنها حدود ۶۰ درصد از آن‌ها به شاخص باکیفیتی از دیالیز (Kt/V>1/2) دست می‌یابند. هرچند نسبت به مطالعات گذشته این کفایت پیشرفت داشته است، اما پژوهشگر بهبود این شاخص را ضروری می‌داند. از طرفی با توجه به رشد روزافزون بیماری‌هایی مانند دیابت و پرفشاری خون و افزایش خطر ابتلا به نارسایی مزمن کلیوی، آموزش در پیشگیری از ابتلا به این بیماری‌ها و بهبود شاخص‌های کیفیت دیالیز حائز اهمیت می‌باشد(۳۲).

۲-۲-۲. مطالعات خارجی

راچمواتی[1] و همکاران در سال ۲۰۲۱، به بررسی بهبود کفایت دیالیز و کیفیت زندگی در بیماران تحت همودیالیز با دو هفته تمرینات حرکتی پرداختند. این مطالعه با هدف تعیین اثرات انجام دامنه حرکتی (ROM) دو بار در هفته به عنوان یک تمرین درون دیالیتیک بر کفایت و کیفیت زندگی بیماران انجام شد. در مجموع ۴۸ شرکت کننده واجد شرایط جذب و این مطالعه را تکمیل کردند. گروه مداخله (۲۴ نفر) دو بار در هفته تمرینات حرکتی را در مدت ۴ هفته دریافت کردند. نتایج در پیش آزمون و در پایان مداخله ارزیابی شد. نتایج نشان داد که دامنه تمرینات حرکتی در بهبود کفایت دیالیز مؤثر است که شامل نسبت احتباس اوره و Kt/V است. کیفیت زندگی به ویژه در زمینه علائم و اثر بیماری کلیوی به میزان قابل توجهی بهبود یافته است. ادغام دامنه حرکت در مراقبت‌های دیالیز به عنوان یک مداخله بالقوه برای بهبود کفایت و کیفیت زندگی دیالیز برای بیماران تحت همودیالیز نشان داد(۱۵۰).

هارگرو[2] و همکاران در سال ۲۰۲۱، به بررسی تأثیر تمرینات هوازی بر علائم مربوط به دیالیز در بیماران همودیالیز پرداختند. در این مطالعه مرور سیستماتیک ومتاآنالیز، از ۳۰۴۸ مطالعه شناسایی شده،

[1] Rochmawati
[2] Hargrove

۱۵ کارآزمایی تصادفی کنترل شده دارای معیارهای واجد شرایط بودن بودند. این مطالعات تأثیر ورزش هوازی را بر سندرم پای بی قرار (دو مطالعه)، اختلال خواب (چهار مطالعه)، اضطراب (چهار مطالعه)، افسردگی (نه مطالعه)، گرفتگی عضلات (یک مطالعه) و خستگی (یک مطالعه) بررسی کردند. متاآنالیز علائم افسردگی در مطالعات با استفاده از پرسشنامه افسردگی بک، کاهش بیشتری را در نمره افسردگی بک با ورزش در مقایسه با کنترل نشان داد. به طور کلی نتایج نشان داد که در بزرگسالان تحت درمان با همودیالیز، ورزش هوازی چندین علائم مرتبط با همودیالیز از جمله سندرم پای بی قرار، علائم افسردگی، گرفتگی عضلات و خستگی. را بهبود می‌بخشد(۱۵۱).

ششادری[1] و همکاران در سال ۲۰۱۹، به بررسی همراهی فعالیت بدنی بالاتر با خستگی کمتر و بی‌خوابی در بین بیماران مبتلا به همودیالیز پرداختند. این مطالعه بر روی ۴۸ بیمار که در ۳ کلینیک دیالیز سانفرانسیسکو تحت درمان همودیالیز قرار گرفتند، طراحی شده است. فعالیت بدنی با استفاده از گام شمار اندازه‌گیری شد و طی ۱ هفته ارزیابی علائم ثبت شد. علائم با استفاده از بار و شدت علائم کل بر روی شاخص علائم دیالیز، علائم فردی بر DSI، نمرات کیفیت زندگی بیماری کلیه ارزیابی شد. نتایج نشان داد که هفتادوسه درصد از بیماران خستگی را گزارش کرده‌اند. بعد از تنظیم سن، جنس، دیابت و آلبومین سرم، فعالیت بدنی با ۰/۲ امتیاز شدت خستگی کمتر در ۱۰۰۰ مرحله در روز همراه بود. فعالیت بدنی همچنین در مدل‌های تنظیم شده ما با نمره شادابی و بالاتر بودن و بی‌خوابی پایین‌تر همراه بود. فعالیت بدنی با علائم دیگر همراه نبود(۱۵۲).

دیزیوبک[2] و همکاران در سال ۲۰۱۵، یک مطالعه مقطعی تحت عنوان تأثیر ورزش‌های آبی بر آمادگی جسمانی و عملکرد عضلات در بیماران دیالیز انجام دادند. هدف از این مطالعه بررسی تأثیر یک برنامه تمرینی بدنی ۳ ماهه، در یک محیط آبی با بیماران مبتلا به بیماری کلیوی مرحله نهایی (ESRD)، بر آمادگی جسمانی و پارامترهای عملکردی عضلات مفصل زانو است. این مطالعه شامل ۲۰ بیمار ESRD بود که در مرکز دیالیز بیمارستان دانشگاه در وروکلاو تحت درمان با همودیالیز قرار گرفتند. قبل و ۳ ماه

---
[1] Sheshadri
[2] Dziubek

بعد از تمرین بدنی در آب، آزمایش برای ارزیابی آمادگی جسمانی هر بیمار انجام شد. علاوه بر این، یک اندازه‌گیری از پارامترهای نیروی سرعت گرفته شد. برنامه آموزش ۳ ماهه در روزهای غیر همودیالیزی، در استخر تفریحی دانشگاه تربیت‌بدنی در وروکلاو برگزار شد. در ارزیابی آمادگی جسمانی زنان موردمطالعه، بیشترین پیشرفت در آزمایش‌های ارزیابی قدرت اندام فوقانی و تحتانی و همچنین انعطاف‌پذیری پایین بدن حاصل شد. مقادیر بالاتر پارامترهای نیرو- سرعت باعث دستیابی به نتایج تست آمادگی جسمانی بهتر می‌شود(۱۵۳).

در پژوهش گروسارد (۲۰۱۴)، مطالعه‌ای تجربی در فرانسه روی ۲۰ بیمار مزمن کلیوی تحت درمان همودیالیز با عنوان، اثرات مفید برنامه پدال زدن حین دیالیز در بیماران با نارسایی مزمن کلیوی، انجام شد که در آن ۵ زن و ۱۵ مرد انتخاب شدند. این بیماران تحت درمان با همودیالیز معمولی سه بار در هفته قرار داشتند. بیماران به صورت تصادفی به دو گروه تقسیم شدند و در یک دوره سه ماهه تحت نظر قرار گرفتند. گروه مداخله، در دو ساعت ابتدایی دیالیز با پدال زدن فعالیت داشتند و در پایان مطالعه به بررسی آمادگی جسمانی، پروفایل چربی و وضعیت آنتی‌اکسیدان پرداخته شد که آمادگی جسمانی در گروه مداخله افزایش داشت و پروفایل چربی در آن‌ها با نسبت به گروه کنترل کاهش نشان داد. گروسارد در پایان مطالعه نتیجه گرفت که انجام فعالیت هوازی در حین دیالیز جهت بیماران مزمن کلیوی می‌تواند اختلالات همراه با این بیماری را کاهش دهد و توصیه به انجام سایر مطالعات با گروه هدف بیشتر و بزرگ‌تر دارد(۳).

کرکمن در سال ۲۰۱۳ در ولز انگلستان، مطالعه‌ی مشابهی را تحت عنوان تعامل بین فعالیت حین دیالیز و کفایت دیالیز انجام داد و در آن به مقایسه بین عملکرد فعالیت حین دیالیز، افزایش زمان دیالیز در بهبود کفایت دیالیز و برداشت فسفات پرداخت. در این پژوهش تعداد ۱۱ بیمار در سه دوره مختلف تحت دیالیز معمولی، دیالیز با فعالیت طی یک ساعت انتهایی دیالیز و افزایش زمان دیالیز به مدت ۳۰ دقیقه پرداخت که نتایج به شرح زیر است: فعالیت، اثر معناداری روی تغییر و بهبود کفایت دیالیز نسبت به زمان دیالیز معمولی نشان نداد ولی افزایش زمان در آن‌ها نسبت به زمان دیالیز معمولی، باعث بهبود کفایت دیالیز (Kt/V) گردید. در عوض انجام فعالیت باعث برداشت بهتر فسفات نسبت به افزایش زمان،

جهت کاهش نسبت فسفات گردید؛ کرکمن نتیجه گرفت که انجام فعالیت حین دیالیز نمی‌تواند جایگزین افزایش زمان دیالیز گردد و نمی‌تواند باعث بهبود کفایت دیالیز گردد(۷۱).

تنتوری در سال ۲۰۱۲، یک مطالعه مقطعی تحت عنوان اثرات مثبت جلسات دیالیز طولانی‌تر همراه با پیامدهای میانجی بهتر و زنده ماندن بیماران دیالیزی ۳ بار در هفته، انجام داد که در آن به بررسی اثرات طول مدت انجام همودیالیز روی ۳۷۴۱۴ بیمار در ۱۲ کشور از نقاط مختلف جهان پرداخت. این بیماران سه بار در هفته تحت همودیالیز قرار می‌گرفتند و از ۱۲۰ دقیقه تا ۴۲۰ دقیقه تحت این درمان قرار داشتند. متوسط این زمان از ۲۱۴ دقیقه در ایالات‌متحده تا ۲۵۶ دقیقه در استرالیا و نیوزلند متفاوت برآورد شد. در نتایج حاصله، عوارض و پیامدهایی مانند فشارخون سیستولیک قبل و بعد از دیالیز پایین‌تر، وزن گیری حین دیالیز بالاتر، هموگلوبین بالاتر و سطح گلبول‌های سفید و فسفات کمتر در بیماران با زمان دیالیز طولانی‌تر دیده شد(۱۵۴).

پارسونز در سال ۲۰۰۶ مطالعه‌ای با عنوان اثربخشی تمرینات ورزشی در طی همودیالیز بر کفایت دیالیز و آمادگی جسمانی، در بیمارستان کینگ استون کانادا، روی ۱۳ بیمار (۶ نفر گروه مداخله و ۷ نفر گروه کنترل) انجام داد. مداخله به صورت فعالیت حین دیالیز به مدت ۶۰ دقیقه در ۲ ساعت ابتدایی دیالیز به صورت پدال زدن انجام شد. این مداخله به مدت ۲۰ هفته و سه بار در هفته برای بیماران سیکل صبح و بعدازظهر انجام شد. انتخاب ۶۰ دقیقه در دو ساعت اول به دو دلیل بود، چرا که در مطالعات قبل اولاً سه دوره ۱۵ دقیقه‌ای اثر چندانی در برداشت اوره نشان نمی‌داد و ۲ دوره ۳۰ دقیقه‌ای فعالیت اثر بهتری در افزایش اوره محلول همودیالیز دارد و در ثانی، در انتهای دیالیز بیماران دچار علائم هیپوتانسیون خواهند شد و انجام فعالیت مقدور نمی‌باشد. در نتایج به دست آمده در این پژوهش، افزایش میزان کفایت دیالیز (Kt/V) به میزان حدود ۱۱ درصد ($P<0.05$) در پایان ماه اول فعالیت نشان داد و تا پایان مداخله حدود ۱۹-۱۸ درصد بالاتر باقی ماند. پژوهشگر مداخله را در بالا بردن کفایت دیالیز مؤثر می‌داند(۴۳).

در پژوهشی که وایتی‌لینگهام در سال ۲۰۰۴ در استرالیا انجام داد؛ تعداد ۹ بیمار همودیالیزی مزمن با سابقه حداقل شش ماه دیالیز در مطالعه‌ای با عنوان بررسی اثربخشی طول مدت زمان دیالیز و فعالیت بر

برداشت فسفات در بیماران همودیالیزی، تحت نظر و موردمطالعه قرار داد. این ۹ بیمار به طور تصادفی به دو گروه تقسیم شدند و به صورت متقاطع تحت همودیالیز معمولی و دیالیز با زمان ۵ ساعت قرار گرفتند. از طرفی در مطالعه دوم تعداد ۱۲ بیمار غیر بیماران مطالعه قبل (شامل ۶ مرد و ۶ زن) انتخاب شدند و به صورت تصادفی به سه گروه که شامل بدون انجام فعالیت، یک هفته فعالیت بلافاصله قبل از شروع دیالیز و یک هفته پدال زدن به مدت ۳۰ تا ۶۰ دقیقه حین دیالیز بود، تقسیم شدند. در نتایج حاصله ۱۳ درصد افزایش برداشت فسفات در گروه با دیالیز طولانی (۵ ساعت) دیده شد (P<0.02). ولی در برداشت اوره و Kt/V تفاوت معناداری دیده نشد. در مطالعه انجام فعالیت، هرچند افزایش برداشت فسفات مشاهده شد ولی از لحاظ آماری معنادار نبود (P=0.055)؛ و در برداشت اوره (URR) هرچند تمایل به افزایش در گروه فعالیت دیده شد ولی تفاوت معناداری وجود نداشت (P=0.08)؛ و در بررسی Kt/V نیز در گروه فعالیت تأثیر معناداری نسبت به گروه کنترل یافت نشد (P=0.79)؛ بنابراین طبق این مطالعه می‌توان گفت که افزایش زمان باعث بهبود برداشت فسفات می‌گردد ولی در بهبود کفایت نمی‌توان گفت که انجام فعالیت مؤثر است(۷۵).

کونگ در سال ۱۹۹۹، مطالعه‌ای تجربی در بیماران تحت همودیالیز با عنوان اثر فعالیت حین دیالیز بر روی برداشت مواد محلول، در لستر انگلستان انجام داد که در آن تعداد ۱۱ بیمار (۱۰ مرد و یک زن) انتخاب شد و به صورت تصادفی تقسیم‌بندی شده و مداخله روی آن‌ها انجام شد و نتایج با دیالیز بدون مداخله مقایسه گردید. در این مطالعه، Kt/V و URR و همچنین ریباند اوره و میزان برداشت پتاسیم مورد بررسی قرار گرفت. در نتایج به دست آمده، Kt/V و URR هر دو به طرز معناداری افزایش یافتند(۴۲).

۲-۳. جمع‌بندی

مطالعات پیشین نشان داد که فعالیت فیزیکی در بهبود کفایت دیالیز اثر داشته است؛ اما با توجه به جستجوهای انجام شده، تاکنون در هیچ یک از مطالعات یا مقالات در دسترس، در زمینه بررسی اثرات فعالیت فیزیکی در بیماران همودیالیزی و تأثیر آن بر شاخص‌های همودینامیک و خستگی در این بیماران مطالعه‌ای انجام نشده است. در این مطالعه در یک گروه از بیماران مداخله فعالیت فیزیکی به همراه دیالیز

و در گروه دیگر روش روتین دیالیز پرداخته شد و تأثیر همزمان این دو با هم سنجیده شد. از طرفی دیگر از محدودیت‌های مطالعات گذشته این بوده است که انجام آن‌ها با حجم نمونه پایین باعث کاهش توان مطالعه شده است. سعی شد برای افزایش توان مطالعه از نمونه بیشتر استفاده گردد. لذا این مطالعه با هدف مقایسه اثربخشی فعالیت فیزیکی حین دیالیز بر کفایت دیالیز، شاخص‌های همودینامیک و خستگی در بیماران تحت همودیالیز در بیمارستان‌های شهر بوشهر در سال ۱۳۹۹ انجام گرفت.

فصل سوم

روش‌شناسی پژوهش

۳. روش‌شناسی پژوهش

در این فصل روش پژوهش، نوع پژوهش، جامعه و واحد پژوهش، محیط پژوهش، حجم نمونه و محاسبه آن، روش نمونه‌گیری، ابزارهای مورداستفاده در پژوهش و تعیین روایی و پایایی آن‌ها، مراحل انجام پژوهش، روش تجزیه‌وتحلیل داده‌ها و در پایان ملاحظات اخلاقی پژوهش حاضر ارائه می‌گردد.

۳-۱. مواد و روش پژوهش

۳-۱-۱. نوع پژوهش

پژوهش حاضر یک مطالعه کارآزمایی بالینی شاهد دار تصادفی[1] می‌باشد که در آن به مقایسه اثربخشی فعالیت فیزیکی حین دیالیز بر کفایت دیالیز، شاخص‌های همودینامیک و خستگی در بیماران تحت همودیالیز در بیمارستان‌های شهر بوشهر در سال ۱۳۹۹ پرداخته شد.

۳-۱-۲. جامعه و واحد پژوهش

جامعه پژوهش شامل بیماران تحت همودیالیز در بیمارستان‌های شهر بوشهر در سال ۱۳۹۹ بود.

۳-۱-۳. محیط پژوهش

محیط پژوهش بخش دیالیز بیمارستان‌های آموزشی شهر بوشهر بود.

۳-۱-۴. معیارهای ورود به مطالعه

معیارهای ورود به مطالعه شامل موارد زیر می‌باشد:

۱. سن ۱۸ تا ۶۵ سال

[1] Randomized Clinical Trial(RCT)

۲. سابقه همودیالیز بیش‌تر از ۳ ماه

۳. توانایی انجام پیاده‌روی به شکل مستقل (حداقل ۲ دقیقه)

۴. عدم سابقه آنژین صدری ناپایدار تنگی نفس و حمله قلبی در یک ماه گذشته

۵. عدم سابقه نارسایی احتقانی قلب

۶. عدم درمان با داروهای اعصاب و روان

۷. تحمل دیالیز ۴ ساعته در جلسات گذشته

۸. نداشتن سابقه هیپوکالمی (K<3.5 Meq/L)

۳-۱-۵. معیارهای خروج از مطالعه

معیارهای خروج شامل موارد زیر بود:

۱. بروز عوارض قلبی عروقی مانند آنژین ناپایدار قلبی، افزایش فشارخون سیستولیک بیشتر یا مساوی ۱۶۰ میلی‌متر جیوه یا فشارخون دیاستولیک بیشتر یا مساوی ۱۲۰ میلی‌متر جیوه

۲. بروز هیپوتانسیون به شکل کاهش فشارخون سیستولیک کمتر از ۹۰ میلی‌متر جیوه، یا افت بیش از ۳۰ میلی‌متر جیوه در فشارخون سیستولیک نسبت به فشارخون اولیه

۳. بروز هرگونه عارضه‌ای که مانع ادامه انجام مداخله شود مانند عوارض نورولوژیکی، عضلانی اسکلتی، عروقی و ...) به تائید پزشک

۴. شرکت نکردن در ۴ جلسه یا بیشتر از جلسات در طی مطالعه

۵. عدم انجام همودیالیز به علت فوت یا پیوند کلیه

۶. بروز اشکال در دسترسی عروقی

۷. عدم تمایل به ادامه شرکت در مطالعه

۳-۱-۶. محاسبه حجم نمونه

با به‌کارگیری نرم‌افزار Gpower 3.1.9.2 و با در نظر گرفتن سطح اطمینان ۹۵ درصد، توان آزمون ۸۰ درصد، میانگین و انحراف معیار شاخص کیفیت دیالیز در گروه کنترل به میزان ۱/۳۳ و در گروه ورزش

به میزان ۱/۴۵ و انحراف معیار یکسان در ۲ گروه به میزان ۰/۱۸، حجم نمونه کل ۷۶ نفر بود. همچنین با احتساب ۱۰ درصد ریزش (q) و فرمول $n' = \frac{n}{(1-q)}$ حجم نمونه کل درنهایت ۸۴ نفر و در هر گروه ۴۲ نفر بود.

۳-۱-۷. روش نمونه‌گیری

نمونه‌گیری از بیماران در دسترس و به صورت آسان بود اما تقسیم بیماران در ۲ گروه مداخله و کنترل به صورت تخصیص تصادفی بود.

۳-۱-۸. ابزار گردآوری داده‌ها

۳-۱-۸-۱. فرم بررسی اطلاعات جمعیت شناختی

یک فرم محقق ساخته بود که با توجه به متغیرهای جمعیت شناختی موردنیاز در پژوهش طراحی گردید. متغیرهای جمعیت شناختی شامل جنس، سن، سطح تحصیلات، وضعیت تأهل و وزن بیمار قبل و بعد از انجام همودیالیز بود.

۳-۱-۸-۲. پرسشنامه شدت خستگی (FSS)[1]

پرسشنامه شدت خستگی توسط کروپ[2] و همکاران (۱۹۸۹) به‌منظور سنجش خستگی در بیماران ام اس ساخته شده و شامل ۹ سؤال است که از پرسشنامه ۲۸ آیتمی خستگی استخراج شده است. پرسشنامه FSS از جمله ابزارهای بسیار معتبر است که برای ارزیابی شدت خستگی استفاده می‌شود و خستگی را به صورت کلی و به سرعت در این بیماران می‌سنجد، به‌طوری که نمره حاصل از آن با میزان و شدت خستگی بیمار کاملاً متناسب است و برای تمام بیماران قابل‌فهم و ۹۸ درصد بیماران بدون نیاز به کمک قادر به پاسخگویی به سؤالات آن هستند. سؤالات پرسشنامه بر اساس طیف لیکرت هفت‌درجه‌ای (کاملاً موافقم نمره ۷، موافقم نمره ۶، نسبتاً موافقم نمره ۵، متوسط نمره ۴، نسبتاً مخالفم نمره ۳، مخالفم نمره ۲ و کاملاً مخالفم نمره ۱) نمره‌گذاری می‌شوند. نمره کل از تقسیم جمع نمرات بر ۹ محاسبه می‌شود.

---
[1] Fatigue severity scale
[2] Krupp

این نمره نیز بین ۱ تا ۷ می‌باشد که امتیاز ۷ نشان‌دهنده بالاترین میزان خستگی و امتیاز ۱ بیانگر فقدان خستگی می‌باشد. عدد ۱ نشانگر عدم وجود خستگی، ۲-۴ خستگی متوسط و بالاتر از ۴ نشانگر خستگی شدید خواهد بود.

کروپ و همکاران (۱۹۸۹) روایی پرسشنامه شدت خستگی را مناسب و قابل‌قبول ذکر کرده و پایایی آن را به روش آلفای کرونباخ برابر ۰/۹۱ به دست آورده است(۱۵۵). روایی محتوی و صوری آن نیز در مطالعات ذاکری مقدم و رسولی تائید شده است(۱۵۶, ۱۵۷). پایایی این ابزار در مطالعات گوناگون توسط رسولی، ذاکری مقدم با ضریب آلفای کرونباخ ۰/۹۱، ۰/۹۴ تائید شده است.

۳-۱-۸-۲. پرسشنامه کفایت دیالیز

برای محاسبه کفایت دیالیز از معیار Kt/V[1] از طریق فرمول

$$Kt/V = -\ln(R - 0.008\ 3\ t) + (4 - 3.5\ 3\ R)\ 3\ UF/W$$

(کلیرانس اوره، T مدت زمان دیالیز، V: توزیع حجم اوره، R: ضریب تولید اوره و تابعی از طول جلسه است که در یک جلسه دیالیز معادل ۲۴ تا ۳۲ هزارم می‌باشد) و نسبت کاهش اوره[2] که با فرمول BUN قبل از دیالیز منهای BUN بعد از دیالیز تقسیم بر BUN قبل از دیالیز محاسبه و استفاده شد. نمونه‌گیری قبل از دیالیز بلافاصله قبل از شروع همودیالیز و از لاین شریانی و نمونه‌گیری بعد از دیالیز با قطع محلول دیالیز جهت توقف برداشت اوره به مدت سه دقیقه و کاهش دور پمپ به زیر ۱۰۰ سی‌سی در دقیقه در ۵ دقیقه پایانی دیالیز انجام گرفت. نمونه‌ها که بی‌نام بوده و با کد مشخص شده‌اند، بلافاصله به آزمایشگاه مرکزی فرستاده شده و با دستگاه DIRUI 1200 توسط یک تکنسین مشخص انجام شد. جهت اندازه‌گیری وزن از ترازوی دیجیتال مارک seca که توسط شرکت «پایش ابزار برتر» به صورت سالانه کالیبره می‌شود استفاده شد.

۳-۱-۸-۳. اندازه گیری شاخص‌های همو دینامیک

1 K : dialyzer clearance of urea
t : dialysis time
V : volume of distribution of urea, approximately equal to patient's total body water
[2] Urea Reduction Ratio

اندازه‌گیری شاخص‌های همودینامیک شامل درجه حرارت، نبض، فشارخون، تنفس و درصد اشباع اکسیژن خون شریانی بود. اندازه‌گیری درجه حرارت با دماسنج دیجیتال و به صورت زیرزبانی، فشارخون و پالس اکسی متری با دستگاه SAADAT(که به صورت سالانه توسط شرکت «آزمایشگاه آزمون کیفیت افق» کالیبره می‌شود) و نبض و تنفس با شمارش توسط محقق در یک دقیقه انجام شد. دیالیز در بیماران در بیمارستان تأمین اجتماعی بوشهر با دستگاه Fresenius 4008B و Fresenius S Classic و در بیمارستان شهدای خلیج‌فارس بوشهر با دستگاه B-Braun dialog plus و Nipro 550 و در مرکز فاطمه زهرا (س) وابسته به بیمارستان شهدای خلیج‌فارس بوشهر با دستگاه Gambro AK 95/96 و Nipro انجام شد.

۳-۱-۹. روش پژوهش

مطالعه حاضر یک کارآزمایی بالینی شاهد دار تصادفی است که در مرکز همودیالیز وابسته به دانشگاه علوم پزشکی و مدیریت درمان تأمین اجتماعی بوشهر در سال ۱۳۹۹ انجام شد. پژوهشگر پس از تصویب پروپوزال و تائید کمیته اخلاق دانشگاه و ثبت پژوهش در سامانه ثبت کارآزمایی بالینی بین‌المللی، با اخذ معرفی‌نامه از معاونت محترم پژوهشی دانشگاه و پس از انجام هماهنگی‌های لازم به بخش دیالیز بیمارستان‌های آموزشی بوشهر و سلمان فارسی بوشهر مراجعه نمود؛ سپس ضمن معرفی خود و اهداف مطالعه به واحدهای موردپژوهش، رضایت آنان را برای مشارکت در اجرای طرح جلب نموده و رضایت‌نامه کتبی آگاهانه برای شرکت را اخذ و نمونه‌گیری شروع گردید. به‌تمامی شرکت کننده‌گان اطمینان داده شد که اطلاعات آنان محرمانه باقی خواهد ماند و هر زمان که تمایل داشتند و بدون هیچ پیامدی، می‌توانند از مطالعه خارج شوند. ۸۴ بیمار از بیماران بستری در بخش‌های همودیالیز بیمارستان‌های شهر بوشهر با تخصیص تصادفی و به صورت قرعه‌کشی به دو گروه (یک گروه آزمون و یک گروه کنترل) تقسیم شد. در گروه آزمون مداخله فعالیت فیزیکی حین دیالیز بود؛ و گروه کنترل نیز تحت روش روتین، همودیالیز شد. در هردو گروه آزمون و کنترل زمان دیالیز یکسان و ۴ ساعت بود. نحوه انجام فعالیت فیزیکی بیمار به صورت پدال زدن در بستر به مدت ۱۵ دقیقه در ۲ نوبت و با در نظر گرفتن ۱۵ دقیقه استراحت در بین

تمرینات انجام گردید. قبل از شروع مداخله مشارکت‌کنندگان به مدت پنج دقیقه خود را گرم کرده و پس از پایان نیز با پدال زدن آهسته خود را سرد کردند. شدت پدال زدن طوری بود که بیمار ۱۰ درصد افزایش نبض نسبت به نبض قبل از شروع فعالیت پیدا کند. در ابتدا قبل از شروع تمرین، فشارخون و نبض بیمار اندازه‌گیری و ثبت و در پایان ۱۵ دقیقه نیز فشارخون اندازه‌گیری گردید. نبض بیمار با استفاده از مونیتورینگ در طول فعالیت به صورت مداوم پایش گردید، نبض بیمار پس از انجام تمرین حداقل باید ۱۰ درصد نسبت به نبض قبل از شروع فعالیت افزایش پیدا کند، در غیر این صورت بیمار باید با سرعت بیشتری پدال بزند. محدوده ایمن تعداد نبض در بیماران هنگام فعالیت حداقل ۵۰ و حداکثر ۱۲۵ ضربان در دقیقه در نظر گرفته شد. پدال از مارک «ATMED» محصول تایوان می‌باشد. این پدال ۳/۵ کیلوگرم وزن داشته و به‌راحتی قابل‌حمل می‌باشد. پدال توسط مجری پژوهش در اختیار بیمار قرار گرفته و بیمار در حین فعالیت تحت کنترل تیم درمان قرار داشت. برنامه فعالیت فیزیکی در گروه آزمون به مدت ۴ هفته و در هر هفته ۳ جلسه بود. همه بیماران شرکت‌کننده در مطالعه، ۳ روز در هفته و در هر جلسه به مدت ۴ ساعت دیالیز شدند.

قبل و بعد از آزمون (یک ماه) شاخص‌های همودینامیک و کفایت دیالیز و خستگی بیماران اندازه‌گیری شد. داده‌ها پس از جمع‌آوری با استفاده از نرم‌افزار spss ویرایش ۲۴ کاربرد آمار توصیفی و استنباطی، مورد تجزیه‌وتحلیل قرار گرفت.

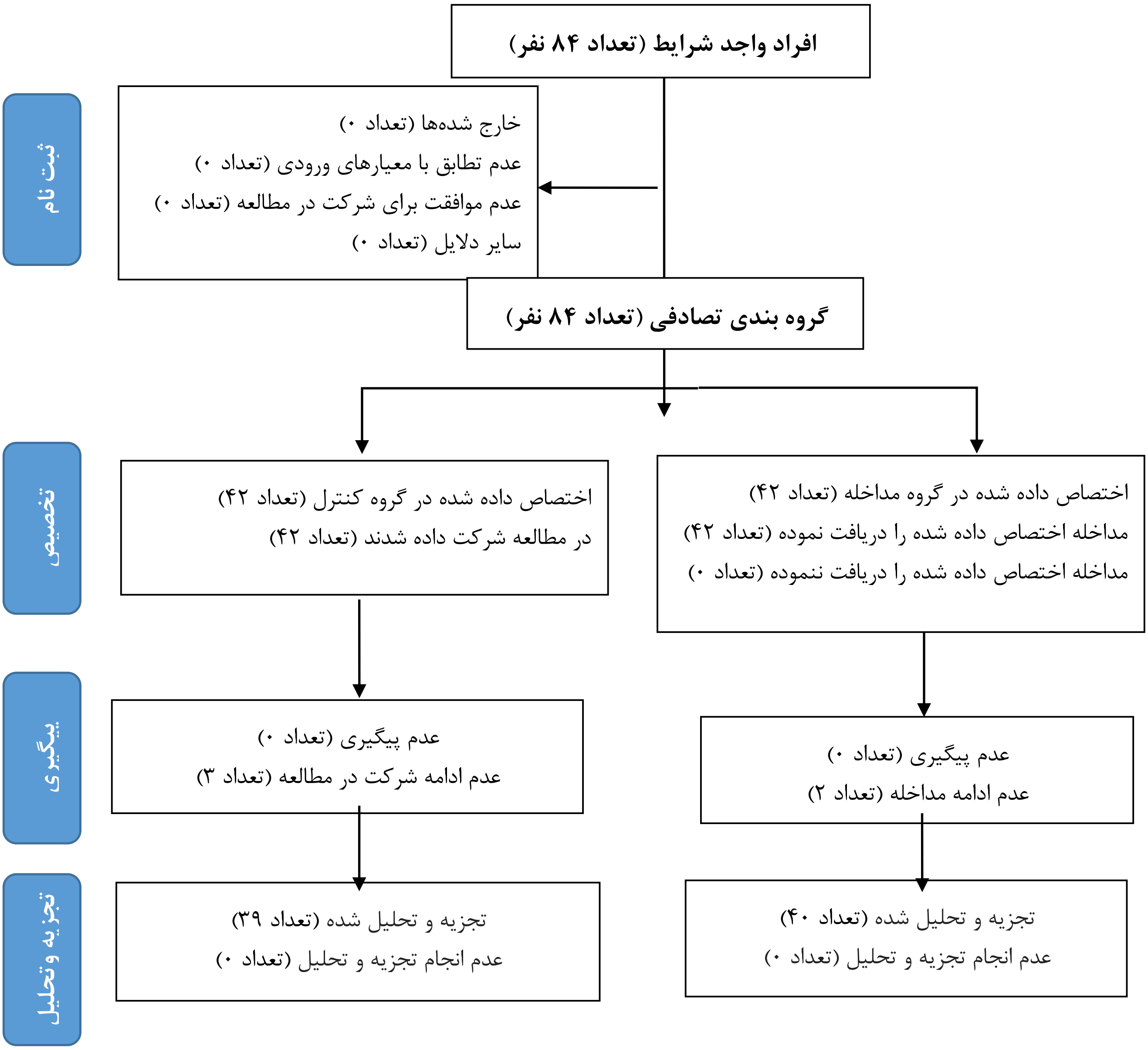

شکل ۳-۱. نمودار کنسورت[1] مراحل اجرای تحقیق

[1] Consort

۳-۱-۱۱. روش تجزیه‌وتحلیل داده‌ها[1]

پیامد اولیه در این مطالعه شاخص کیفیت دیالیز می‌باشد. با استفاده از آمار توصیفی (فراوانی، میانگین، انحراف معیار و فاصله اطمینان ۹۵ درصد) و آزمون‌های آماری کای مربع (برای مقایسه متغیرهای دموگرافیک بین گروه‌ها)، آنالیز واریانس یک‌طرفه (مقایسه میانگین متغیرهای کمی بین گروه‌های مختلف)، آزمون تعقیبی توکی در صورت معنی‌دار بودن آزمون آنالیز واریانس یک‌طرفه (برای مقایسه دوبه‌دو بین گروه‌ها) و آزمون تی زوج (مقایسه میانگین متغیرهای کمی قبل و بعد در هر گروه) با کمک نرم‌افزار SPSS ویرایش ۲۴ انجام شد. Effect size (d) با استفاده از روش Cohen محاسبه شد و مقادیر کمتر از ۰/۳، ۰/۳ تا کمتر از ۰/۵ و بیشتر از ۰/۸ به ترتیب کوچک، متوسط و بزرگ در نظر گرفته شد (۷۴). در کلیه تجزیه‌وتحلیل‌ها سطح معنی‌داری ۰/۰۵ در نظر گرفته شد. جهت تعیین نرمالیتی داده‌ها از آزمون شاپیرو-ویلک و نسبت چولگی به خطای معیار آن استفاده شد.

۳-۲. ملاحظات اخلاقی

این مطالعه طرح مصوب شورای پژوهشی دانشگاه علوم پزشکی بوشهر بوده و در کمیته اخلاق معاونت پژوهشی دانشگاه علوم پزشکی بوشهر با کد اخلاق IR.BPUMS.REC.1398.130 و کد RCT 20150529022466N15 که در مرکز ثبت کارآزمایی های بالینی ایران مورد تائید و ثبت قرار گرفت. نکات اخلاقی که در طی پژوهش موردنظر قرار گرفت (پیوست شماره ۱). عبارت بودند از:

۱) پژوهش حاضر پس از کسب مجوزهای قانونی لازم از دانشگاه علوم پزشکی بوشهر، انجام شد.

۲) در تجزیه‌وتحلیل نتایج، رعایت صداقت به عمل آمده و تحریفی در داده‌ها صورت نگرفت.

۳) نتایج این تحقیق در صورت نیاز در اختیار مسئولین قرار خواهد گرفت تا در جهت ارتقا سلامت افراد جامعه گام‌های مؤثری برداشته شود.

۴) رعایت نکات اخلاقی در استفاده از منابع (کتاب، پژوهش‌ها و مجلات و...) در نظر گرفته شد.

[1] Statistical Analysis

۵) نتایج استخراج یافته از مطالعه در صورت درخواست واحدهای پژوهش در اختیار آنان قرار داده خواهد شد.

۶) اطمینان به واحدهای پژوهش به‌منظور داشتن اختیار کامل جهت خروج از مطالعه در هر مرحله‌ای از پژوهش داده شد.

فصل چهارم

یافته‌های پژوهش

۴. یافته‌های پژوهش

در این فصل به بیان یافته‌های حاصل از تجزیه‌وتحلیل اطلاعات و داده‌های گرد آوری شده می‌پردازیم. به منظور بیان راحت و درک بیشتر، برخی جزئیات یافته‌ها در قالب ۲۲ جدول مربوط به واحدهای موردپژوهش ارائه شده است.

در راستای هدف اول پژوهش " تعیین ویژگی‌های جمعیت شناختی در دو گروه آزمون و کنترل در بیمارستان‌های شهر بوشهر در سال ۱۳۹۹" جداول (۴-۱) (۴-۲) ارائه شده است.

در راستای هدف دوم " تعیین و مقایسه میزان اوره خون و وزن در بیماران قبل و بعد از مداخله در دو گروه آزمون و کنترل به تفکیک در بیماران تحت همودیالیز در بیمارستان‌های شهر بوشهر در سال ۱۳۹۹" و هدف سوم پژوهش " تعیین و مقایسه میزان اوره خون و وزن در بیماران قبل و بعد از مداخله بین دو گروه آزمون و کنترل در بیمارستان‌های شهر بوشهر در سال ۱۳۹۹ " و هدف چهارم پژوهش "مقایسه میانگین تغییرات اوره خون و وزن در بیماران در طول مداخله بین دو گروه آزمون و کنترل در بیمارستان‌های شهر بوشهر در سال ۱۳۹۹" جدول (۴-۲) و جدول (۴-۳) ارائه شده است.

در راستای هدف پنجم " تعیین و مقایسه Kt/V در بیماران بعد از مداخله بین دو گروه آزمون و کنترل در بیمارستان‌های شهر بوشهر در سال ۱۳۹۹" جدول (۴-۴) ارائه شده است.

هدف ششم " تعیین و مقایسه شاخص‌های همودینامیک (درجه حرارت، نبض، فشارخون، تنفس و درصد اشباع اکسیژن خون شریانی) در بیماران قبل و بعد از مداخله در هر دو گروه آزمون و کنترل به تفکیک در بیمارستان‌های شهر بوشهر در سال ۱۳۹۹" و هدف هفتم " تعیین و مقایسه شاخص‌های

همودینامیک (درجه حرارت، نبض، فشارخون، تنفس و درصد اشباع اکسیژن خون شریانی) در بیماران قبل و بعد از مداخله بین دو گروه آزمون و کنترل در بیمارستان‌های شهر بوشهر در سال ۱۳۹۹" و هدف هشتم " مقایسه میانگین تغییرات شاخص‌های همودینامیک (درجه حرارت، نبض، فشارخون، تنفس و درصد اشباع اکسیژن خون شریانی) در بیماران در طول مداخله بین گروه آزمون و کنترل در بیمارستان‌های شهر بوشهر در سال ۱۳۹۹" جدول (۴-۵) تا جدول (۴-۱۲) ارائه شده است.

در راستای هدف نهم " تعیین و مقایسه میزان خستگی در بیماران، قبل و بعد از مداخله در هر دو گروه آزمو و کنترل به تفکیک در بیماران تحت همودیالیز در بیمارستان‌های شهر بوشهر در سال ۱۳۹۹" و هدف دهم " تعیین و مقایسه میزان خستگی در بیماران قبل و بعد از مداخله بین دو گروه آزمون و کنترل در بیمارستان‌های شهر بوشهر در سال ۱۳۹۹ " در جدول (۴-۱۳) ارائه شده است.

در راستای هدف یازدهم " مقایسه میانگین تغییرات خستگی در بیماران، در طول مداخله بین گروه در بیماران تحت همودیالیز در بیمارستان‌های شهر بوشهر در سال ۱۳۹۹" در جدول (۴-۱۴) و جدول (۴-۱۵) ارائه شده است.

در این مطالعه که با هدف کلی مقایسه اثربخشی فعالیت فیزیکی حین دیالیز بر کفایت دیالیز، شاخص‌های همودینامیک و خستگی در بیماران تحت همودیالیز در بیمارستان‌های شهر بوشهر در سال ۱۳۹۹ انجام گردید؛ تعداد ۸۴ بیمار (۴۲ آزمون/۴۲ کنترل) وارد مطالعه شدند. پس از شروع مطالعه دو نفر در گروه آزمون و سه نفر در گروه از مطالعه خارج شدند و درنهایت اطلاعات ۴۰ نفر در گروه آزمون و ۳۹ نفر در گروه کنترل مورد تجزیه و تحلیل قرار گرفت.

جدول ۴-۱. مقایسه متغیرهای جمعیت شناختی بیماران بین گروه آزمون و کنترل

متغیر		آزمون	کنترل	کل	X^2	P- value
		تعداد (درصد)	تعداد (درصد)	تعداد (درصد)		
جنسیت	مرد	۲۹(۷۲/۵)	۲۲(۵۶/۴)	۵۱(۶۴/۶)	۲/۲۳	۰/۱۳
	زن	۱۱(۲۷/۵)	۱۷(۴۳/۶)	۲۸(۳۵/۴)		
وضعیت تأهل	مجرد	۶(۱۵)	۱(۲/۶)	۷(۸/۹)	*۳/۷۸	۰/۱۱
	متأهل	۳۴(۸۵)	۳۸(۹۷/۴)	۷۲(۹۱/۱)		
شغل	بیکار	۱۵(۸۵)	۱۹(۴۸/۷)	۳۴(۴۳)		
	شاغل	۱۶(۴۰)	۱۶(۴۱)	۳۲(۴۰/۵)	۲/۴۲	۰/۲۹
	بازنشسته	۹(۲۲/۵)	۴(۱۰/۳)	۱۳(۱۶/۵)		
میزان تحصیلات	زیر دیپلم	۱۹(۴۷/۵)	۳۱(۷۹/۵)	۵۰(۶۳/۳)		
	دیپلم	۱۰(۲۵)	۶(۱۵/۴)	۱۶(۲۰/۳)	۱۰/۱۰۰	۰/۰۰۶
	دانشگاهی	۱۱(۲۷/۵)	۲(۵/۱)	۱۳(۱۶/۵)		
سن		۵۰/۲۵±۱۰/۷۱**	۵۸/۶۴±۸/۵۵**		-۳/۸۴	۰/۰۰۱

** میانگین و انحراف معیار

* آزمون دقیق فیشر

طبق نتایج آورده شده در جدول ۴-۱ از ۷۹ بیمار وارد شده به مطالعه اکثر بیماران مرد (۵۱ نفر، ۶۴/۶ درصد) و متأهل (۷۲ نفر، ۹۱/۲ درصد) بودند. همچنین ۳۲ نفر از بیماران (۴۰/۵ درصد) شاغل و ۵۰ نفر از آنان (۶۳/۳ درصد) در سطح زیر دیپلم بود؛ میانگین سن گروه بیماران کنترل به صورت معنی‌داری بیشتر از گروه آزمون بود (p=۰/۰۰۱) (جدول ۴-۳). بین بیماران گروه کنترل و آزمون از نظر وضعیت تأهل (p=۰/۱۱)، شغل (p=۰/۲۹) تفاوت معناداری وجود نداشت اما بین بیماران گروه کنترل و آزمون از نظر تحصیلات تفاوت معناداری وجود داشت (p=۰/۰۰۶) (جدول ۴-۳).

جدول ۴-۲. مقایسه میزان اوره خون و وزن در بیماران قبل و بعد از مداخله بین دو گروه آزمون و کنترل در بیمارستان‌های شهر بوشهر در سال ۱۳۹۹

گروه		آزمون Mean±SD	کنترل Mean±SD	t	p-value
میزان اوره خون		۰/۶۱۶±۰/۰۴۹	۰/۶۰۱±۰/۰۴۰	۱/۵۲	۰/۱۳
وزن	قبل	۷۲/۹۶±۱۳/۸۵	۷۳/۶۱±۱۱/۹۳	-۰/۲۲۳	۰/۸۲
	بعد	۷۰/۹۹±۱۳/۹۵	۷۱/۵۹±۱۱/۸۵	-۰/۲۰۳	۰/۸۴
T		۱۱/۰	۱۳/۸۰		
p-value		۰/۰۰۰۱	۰/۰۰۰۱		
نیتروژن اوره خون (BUN)	قبل	۵۸/۶۶±۹/۶۵	۵۵/۷۳±۸/۹۲	۱/۴۰	۰/۱۷
	بعد	۲۲/۵۳±۴/۶۹	۲۲/۱۸±۴/۵۱	۰/۳۴	۰/۷۴
T		۳۴/۰۸	۳۷/۴۹		
p-value		۰/۰۰۱	۰/۰۰۱		

طبق نتایج آورده شده در جدول ۴-۲ مقایسه میزان اوره خون بیماران بین گروه آزمون و کنترل نشان داد که تفاوت معناداری بین دو گروه وجود ندارد (p=۰/۱۳)؛ (جدول ۴-۲).

طبق نتایج آورده شده در جدول ۴-۲ در مقایسه میانگین وزن بیمار قبل و بعد از مداخله و میزان تغییر نمره قبل به بعد در گروه آزمون و کنترل؛ میانگین وزن بیمار قبل از مداخله (p=۰/۸۲) و پس از مداخله (p=۰/۸۴) بین گروه آزمون و کنترل تفاوت معناداری وجود نداشت؛ اما میانگین وزن بیمار قبل به بعد در گروه آزمون و کنترل تفاوت معناداری نشان داد؛ بطوریکه میانگین وزن بیمار پس از مداخله در گروه آزمون و کنترل کمتر از قبل از مداخله بود (p<۰/۰۰۰۱) (جدول ۴-۲).

جدول ۴-۳. مقایسه میانگین تغییرات اوره خون و وزن در بیماران در طول مداخله بین دو گروه آزمون و کنترل در بیمارستان‌های شهر بوشهر در سال ۱۳۹۹

گروه	آزمون	کنترل	t	p-value
	Mean±SD	Mean±SD		
تغییرات اوره خون	۰/۶۱۶±۰/۰۴۹	۰/۶۰۱±۰/۰۴۰	۱/۵۲	۰/۱۳
تغییرات وزن	۱/۹۶±۱/۱۳	۲/۰۲±۰/۹۱	-۰/۲۵	۰/۸۰
تغییرات نیتروژن اوره خون (BUN)	۳۶/۱۳±۶/۷۰	۳۳/۵۴±۵/۵۹	۱/۸۵	۰/۰۷

طبق نتایج آورده شده در جدول ۴-۳، مقایسه میانگین تغییرات اوره خون بیماران بین گروه آزمون و کنترل نشان داد که تفاوت معناداری بین دو گروه وجود ندارد (p=۰/۱۳)؛ همچنین مقایسه میانگین تغییرات وزن بیماران بین گروه آزمون و کنترل نشان داد که تفاوت معناداری بین دو گروه وجود ندارد (p=۰/۸۲)؛ (جدول ۴-۳). مقایسه میانگین تغییرات نیتروژن اوره خون (BUN) بیماران بین گروه آزمون و کنترل نشان داد که تفاوت معناداری بین دو گروه وجود نداشت (p=۰/۰۷)؛ (جدول ۴-۳).

جدول ۴-۴. مقایسه میانگین Kt/V در بیماران بعد از مداخله بین دو گروه آزمون و کنترل در بیمارستان‌های شهر بوشهر در سال ۱۳۹۹

گروه	آزمون	کنترل	t	p-value
	Mean±SD	Mean±SD		
میانگین Kt/V	۱/۱۶۳±۰/۱۴	۱/۱۱۵±۰/۱۲	۱/۶۲	۰/۱۱

طبق نتایج آورده شده در جدول ۴-۳، مقایسه میانگین Kt/V بیماران بین گروه آزمون و کنترل نشان داد که تفاوت معناداری بین دو گروه وجود ندارد (p=۰/۱۱)؛ (جدول ۴-۴).

جدول ۴-۵. مقایسه شاخص‌های همودینامیک درصد اشباع اکسیژن خون شریانی در بیماران قبل و بعد از مداخله در هر دو گروه آزمون و کنترل به تفکیک

گروه		آزمون Mean±SD	کنترل Mean±SD	t	p-value
درصد اشباع اکسیژن خون شریانی	قبل	۹۷/۳۹±۱/۴۷	۹۶/۵۰±۱/۳۷	۲/۷۷	۰/۰۰۷
	۱۵ دقیقه	۹۷/۴۱±۰/۵۷	۹۶/۷۹±۱/۳۳	۲/۷۳	۰/۰۰۸
	بعد	۹۷/۸۵±۰/۵۷	۹۷/۲۶±۱/۵۸	۲/۱۹	۰/۰۳۱
T		-۲/۲۰۴	-۴/۶۹		
p-value*		۰/۰۵۰	۰/۰۰۱		
F		۰/۲۵			
p-value**		۰/۷۸			

* مقایسه قبل به بعد

** آزمون اندازه‌های تکراری

طبق نتایج آورده شده در جدول ۴-۵؛ میانگین درصد اشباع اکسیژن خون شریانی بیمار قبل از مداخله (p=۰/۰۰۷) و ۱۵ دقیقه بعد از مداخله (p=۰/۰۰۸) و در بعد از اتمام مداخله (p=۰/۰۳۱) بین گروه آزمون و کنترل تفاوت معناداری وجود داشت. میانگین درصد اشباع اکسیژن خون شریانی بیمار قبل به بعد در گروه آزمون تفاوت معناداری را نشان نداد (p=۰/۰۵۰) اما میانگین درصد اشباع اکسیژن خون شریانی بیمار قبل به بعد در گروه کنترل تفاوت معناداری نشان داد (p=۰/۰۰۱)؛ بطوریکه میانگین درصد اشباع اکسیژن خون شریانی بیمار پس از مداخله در گروه کنترل بیشتر از قبل از مداخله بود (جدول ۴-۵).

از آزمون اندازه‌های تکراری برای مقایسه روند تغییرات درصد اشباع اکسیژن بین دوگروه با تعدیل اثر سن، تحصیلات و درصد اشباع اکسیژن قبل از مداخله استفاده شد. همانطور که در جدول ۴-۵ آمده است روند تغییرات درصد اشباع اکسیژن بین دو گروه تفاوت معنی داری ندارد (p=۰/۷۸)

جدول ۴-۶. مقایسه شاخص همودینامیک درجه حرارت در بیماران قبل و بعد از مداخله در هر دو گروه آزمون و کنترل به تفکیک

گروه		آزمون	کنترل	t	p-value
		Mean±SD	Mean±SD		
درجه حرارت	قبل	۳۶/۹۶±۱/۰۳	۳۶/۷۰±۰/۱۶	۱/۶۰	۰/۱۱۲
	۱۵ دقیقه	۳۶/۹۱±۱/۰۲۶	۳۶/۶۲±۰/۱۴	۱/۷۵	۰/۰۸۴
	بعد	۳۶/۸۲±۱/۰۹	۳۶/۵۰±۰/۱۱	۱/۸۳	۰/۰۷۵
T		۳/۹۵	۷/۳۲		
p-value*		۰/۰۰۱	۰/۰۰۱		
F		۱/۷۵			
p-value**		۰/۱۸			

* مقایسه قبل به بعد

**آزمون اندازه‌های تکراری

طبق نتایج آورده شده در جدول ۴-۶؛ میانگین درجه حرارت بیمار قبل از مداخله (p=۰/۱۱۲) و ۱۵ دقیقه بعد از مداخله (p=۰/۰۸۴) و در بعد از اتمام مداخله (p=۰/۰۷۵) بین گروه آزمون و کنترل تفاوت معناداری را نشان نداد. میانگین درصد درجه حرارت بیمار قبل به بعد در گروه آزمون و کنترل تفاوت معناداری را نشان داد (p=۰/۰۰۱) بطوریکه در گروه آزمون و کنترل میانگین درجه حرارت بیمار پس از مداخله کمتر از قبل از مداخله بود (جدول ۴-۶).

از آزمون اندازۀهای تکراری برای مقایسه روند تغییرات درجه حرارت بین دوگروه با تعدیل اثر سن، تحصیلات و درصد درجه حرارت قبل از مداخله استفاده شد. همانطور که در جدول ۴-۶ آمده است روند تغییرات درجه حرارت بین دو گروه تفاوت معنی داری ندارد (p=۰/۱۸)

جدول ۴-۷. مقایسه شاخص همودینامیک RR در بیماران قبل و بعد از مداخله در هر دو گروه آزمون و کنترل به تفکیک

گروه		آزمون Mean±SD	کنترل Mean±SD	t	p-value
	قبل	۱۷/۹۵±۲/۱۸	۲۰/۳۱±۲/۱۹	۴/۸۱	۰/۰۰۱
تعداد تنفس	۱۵ دقیقه	۱۶/۹۱±۱/۴۹	۱۷/۷۴±۱/۶۷	۰/۴۸۱	۰/۰۲۲
	بعد	۱۵/۹۵±۱/۴۲	۱۷/۳۱±۱/۷۵	-۳/۷۹	۰/۰۰۱
T		۷/۷۳	۱۱/۷۵		
p-value*		۰/۰۰۱	۰/۰۰۱		
F		۲/۸۵			
p-value**		۰/۰۶			

* مقایسه قبل به بعد

**آزمون اندازه‌های تکراری

طبق نتایج آورده شده در جدول ۴-۷؛ میانگین تعداد تنفس بیمار قبل از مداخله ($p=۰/۰۰۱$) و ۱۵ دقیقه بعد از مداخله ($p=۰/۰۲۲$) و در بعد از اتمام مداخله ($p=۰/۰۰۱$) بین گروه آزمون و کنترل تفاوت معناداری را نشان داد. میانگین تعداد تنفس بیمار قبل به بعد در گروه آزمون و کنترل تفاوت معناداری را نشان داد ($p<۰/۰۰۰۱$) بطوریکه در گروه آزمون و کنترل، میانگین تعداد تنفس بیمار بعد از مداخله کمتر از قبل از مداخله بود (جدول ۴-۷).

از آزمون اندازه‌های تکراری برای مقایسه روند تغییرات تعداد تنفس بین دوگروه با تعدیل اثر سن، تحصیلات و درصد تعداد تنفس قبل از مداخله استفاده شد. همانطور که در جدول ۴-۷ آمده است روند تغییرات تعداد تنفس بین دو گروه تفاوت معنی داری ندارد ($p=۰/۰۶$)

جدول ۴-۸. مقایسه شاخص همودینامیک PR در بیماران قبل و بعد از مداخله در هر دو گروه آزمون و کنترل به تفکیک

گروه		آزمون<br>Mean±SD	کنترل<br>Mean±SD	t	p-value
	قبل	۷۷/۸۱±۹/۷۵	۸۰/۴۹±۵/۷۳	۱/۴۹	۰/۱۴
ضربان قلب	۱۵ دقیقه	۸۰/۵۸±۱۰/۶۷	۸۴/۰۸±۸/۱۵	۱/۶۳	۰/۱۱
	بعد	۷۸/۷۰±۹/۳۸	۸۰/۹۷±۵/۸۱	۱/۳۰	۰/۲۰
T		-۰/۹۳	-۰/۶۶		
p-value*		۰/۳۶	۰/۵۱		
F		۱/۶۲			
p-value**		۰/۲۰			

❊ مقایسه قبل به بعد

❊❊آزمون اندازه‌های تکراری

طبق نتایج آورده شده در جدول ۴-۸؛ میانگین **ضربان قلب** بیمار قبل از مداخله (p=۰/۱۴) و ۱۵ دقیقه بعد از مداخله (p=۰/۱۱) و در بعد از اتمام مداخله (p=۰/۲۰) بین گروه آزمون و کنترل تفاوت معناداری را نشان نداد. میانگین **ضربان قلب** بیمار قبل به بعد در گروه آزمون و کنترل تفاوت معناداری را نشان نداد (p>۰/۰۵) (جدول ۴-۸).

از آزمون اندازه‌های تکراری برای مقایسه روند تغییرات **ضربان قلب** بین دوگروه با تعدیل اثر سن، تحصیلات و درصد **ضربان قلب** قبل از مداخله استفاده شد. همانطور که در جدول ۴-۸ آمده است روند تغییرات **ضربان قلب** بین دو گروه تفاوت معنی داری ندارد (p=۰/۲۰)

جدول ۴-۹. مقایسه شاخص همودینامیک فشارخون سیستول در بیماران قبل و بعد از مداخله در هر دو گروه آزمون و کنترل به تفکیک

گروه		آزمون Mean±SD	کنترل Mean±SD	t	p-value
فشارخون سیستول	قبل	۱۴۲/۲۶±۱۳/۵۰	۱۴۲/۱۴±۱۱/۱۷	۰/۰۴۱	۰/۹۷
	۱۵ دقیقه	۱۳۲/۲۲±۱۳/۹۲	۱۲۶/۸۷±۱۴/۱۶	۱/۶۹	۰/۰۹
	بعد	۱۳۳/۱۹±۱۳/۶۴	۱۳۰/۸۶±۱۲/۲۹	۰/۸۰	۰/۴۳
T		۵/۸۵	۸/۴۶		
p-value*		۰/۰۰۱	۰/۰۰۱		
F		۶/۸۱			
p-value**		۰/۰۰۱			

* مقایسه قبل به بعد

**آزمون اندازه‌های تکراری

طبق نتایج آورده شده در جدول ۴-۹؛ میانگین فشارخون سیستول بیمار قبل از مداخله (p=۰/۹۷) و ۱۵ دقیقه بعد از مداخله (p=۰/۰۹) و در بعد از اتمام مداخله (p=۰/۴۳) بین گروه آزمون و کنترل تفاوت معناداری را نشان نداد. میانگین فشارخون سیستول بیمار قبل به بعد، در گروه آزمون و کنترل تفاوت معناداری را نشان داد (p<۰/۰۰۰۱) بطوریکه در گروه آزمون و کنترل، میانگین فشارخون سیستول بیمار بعد از مداخله کمتر از قبل از مداخله بود (جدول ۴-۹).

از آزمون اندازه‌های تکراری برای مقایسه روند تغییرات فشارخون سیستول بین دوگروه با تعدیل اثر سن، تحصیلات و درصد فشارخون سیستول قبل از مداخله استفاده شد. همانطور که در جدول ۴-۹ آمده است روند تغییرات فشارخون سیستول بین دو گروه تفاوت معنی داری داشت (p=۰/۰۰۱)

جدول ۴-۱۰. مقایسه شاخص همودینامیک فشارخون دیاستول در بیماران قبل و بعد از مداخله در هر دو گروه آزمون و کنترل به تفکیک

گروه		آزمون	کنترل	T	p-value
		Mean±SD	Mean±SD		
فشارخون دیاستول	قبل	۸۲/۳۶±۱۰/۸۴	۷۶/۹۴±۵/۹۹	۲/۷۴	۰/۰۰۸
	۱۵ دقیقه	۸۱/۸۷±۱۱/۵۷	۷۳/۷۱±۷/۲۴	۳/۷۷	۰/۰۰۱
	بعد	۷۷/۷۲±۱۰/۳۶	۷۲/۹۹±۶/۵۱	۲/۴۳	۰/۰۱۸
T		۴/۸۱	۶/۱۹		
p-value*		۰/۰۰۱	۰/۰۰۱		
F		۸/۰۳			
p-value**		۰/۰۰۱			

* مقایسه قبل به بعد

** آزمون اندازه‌های تکراری

طبق نتایج آورده شده در جدول ۴-۱۰؛ میانگین فشارخون دیاستول بیمار قبل از مداخله (p=۰/۰۰۸) و ۱۵ دقیقه بعد از مداخله (p=۰/۰۰۱) و در بعد از اتمام مداخله (p=۰/۰۱۸) بین گروه آزمون و کنترل تفاوت معناداری را نشان داد. به طوریکه میانگین فشارخون دیاستول بیماران در گروه آزمون بالاتر از گروه کنترل بود.

میانگین فشارخون دیاستول بیمار قبل به بعد، در گروه آزمون و کنترل تفاوت معناداری را نشان داد (p<۰/۰۰۰۱) بطوریکه در گروه آزمون و کنترل، میانگین فشارخون دیاستول بیمار بعد از مداخله کمتر از قبل از مداخله بود (جدول ۴-۱۰).

از آزمون اندازه‌های تکراری برای مقایسه روند تغییرات فشارخون دیاستول بین دوگروه با تعدیل اثر سن، تحصیلات و درصد فشارخون دیاستول قبل از مداخله استفاده شد. همانطور که در جدول ۴-۱۰ آمده است روند تغییرات فشارخون دیاستول بین دو گروه تفاوت معنی داری داشت (p=۰/۰۰۱)

جدول ۴-۱۱. مقایسه شاخص همودینامیک فشارخون متوسط شریانی در بیماران قبل و بعد از مداخله در هر دو گروه آزمون و کنترل به تفکیک

		آزمون	کنترل	t	p-value
		Mean±SD	Mean±SD		
فشارخون متوسط شریانی (MAP)	قبل	۱۰۳/۰۶±۱۰/۹۳	۹۹/۰۸± ۷/۵۲	۱/۸۹	۰/۰۶۳
	۱۵ دقیقه	۹۵/۹۵±۱۲/۲۶	۸۸/۴۶±۹/۱۳	۳/۰۷	۰/۰۰۳
	بعد	۹۶/۹۲±۱۱/۶۷	۹۲/۹۶±۸/۴۸	۱/۷۲	۰/۰۸۹
T		۵/۴۰	۸/۳۶		
p-value*		۰/۰۰۱	۰/۰۰۱		
F		۷/۰۴			
p-value**		۰/۰۰۱			

❊ مقایسه قبل به بعد

❊❊ آزمون اندازه‌های تکراری

طبق نتایج آورده شده در جدول ۴-۱۱؛ میانگین فشارخون متوسط شریانی بیمار قبل از مداخله (p=۰/۰۶۳) و در بعد از اتمام مداخله (p=۰/۰۸۹) بین گروه آزمون و کنترل تفاوت معناداری را نشان نداد اما میانگین فشارخون متوسط شریانی بیمار در ۱۵ دقیقه بعد از مداخله تفاوت معناداری را نشان داد (p=۰/۰۰۳) به طوریکه میانگین فشارخون متوسط شریانی بیمار در گروه آزمون بالاتر از گروه کنترل بود.

میانگین فشارخون متوسط شریانی بیمار قبل به بعد، در گروه آزمون و کنترل تفاوت معناداری را نشان داد (p<۰/۰۰۰۱) بطوریکه در گروه آزمون و کنترل، میانگین فشارخون متوسط شریانی بیمار بعد از مداخله کمتر از قبل از مداخله بود (جدول ۴-۱۱).

از آزمون اندازه‌های تکراری برای مقایسه روند تغییرات فشارخون متوسط شریانی بین دوگروه با تعدیل اثر سن، تحصیلات و درصد فشارخون متوسط شریانی قبل از مداخله استفاده شد. همانطور که در جدول ۴-۱۱ آمده است روند تغییرات فشارخون متوسط شریانی بین دو گروه تفاوت معنی داری داشت (p=۰/۰۰۱)

جدول ۴-۱۲. مقایسه میانگین تغییرات شاخص‌های همودینامیک (درجه حرارت، نبض، فشارخون، تنفس و درصد اشباع اکسیژن خون شریانی) در بیماران در طول مداخله بین گروه آزمون و کنترل

گروه	آزمون	کنترل	T	p-value
	Mean±SD	Mean±SD		
درصد اشباع اکسیژن خون شریانی	-۰/۷۰±۱/۰۴	-۱/۰۱±۱/۱۴	۱/۲۵	۰/۲۱
درجه حرارت	۰/۲۰±۰/۴۵	۰/۳۳±۰/۴۳	-۱/۳۲	۰/۱۹
RR	۲/۳۷±۳/۷۱	۲/۶۱±۲/۷۳	-۰/۳۳	۰/۷۴
PR	-۰/۸۹±۶/۱۰	-۰/۴۸±۴/۵۸	-۰/۳۳	۰/۷۴
فشارخون سیستول	۷/۴۷±۱۷/۶۶	۲/۴۶±۱۵/۶۱	۱/۳۳	۰/۱۹
فشارخون دیاستول	۴/۶۴±۶/۱۰	۳/۹۵±۳/۹۸	۰/۶۰	۰/۵۵
فشارخون متوسط شریانی MAP	۶/۱۴±۷/۲۰	۶/۱۵±۴/۵۷	۰/۰۲	۰/۹۸

طبق نتایج آورده شده در جدول ۴-۱۲، مقایسه میانگین تغییرات شاخص‌های همودینامیک (درجه حرارت، نبض، فشارخون، تنفس و درصد اشباع اکسیژن خون شریانی) بین گروه آزمون و کنترل نشان داد که تفاوت معناداری بین دو گروه وجود ندارد ($p>۰/۰۵$)؛ (جدول ۴-۱۲).

جدول ۴-۱۳. مقایسه خستگی در بیماران قبل و بعد از مداخله در هر دو گروه آزمون و کنترل به تفکیک

گروه		آزمون Mean±SD	کنترل Mean±SD	t	p-value
خستگی	قبل	۳/۶۸±۱/۲۷	۴/۷۰±۱/۱۵	-۳/۷۲	۰/۰۰۱
	بعد	۳/۰۴±۱/۲۵	۴/۷۰۲±۱/۱۷	-۶/۱۲	۰/۰۰۱
T		۱۱/۶۸	۰/۰۹۹		
p-value		۰/۰۰۱	۰/۹۲۲		

طبق نتایج آورده شده در جدول ۴-۱۳؛ میانگین خستگی بیمار قبل از مداخله (p=۰/۰۰۱) و در بعد از اتمام مداخله (p=۰/۰۰۱) بین گروه آزمون و کنترل تفاوت معناداری را نشان داد. به طوریکه میانگین خستگی بیمار در گروه آزمون کمتر از گروه کنترل بود.

میانگین خستگی بیمار قبل به بعد، در گروه آزمون تفاوت معناداری را نشان داد (p<۰/۰۰۰۱) بطوریکه در گروه آزمون، میانگین خستگی بیمار بعد از مداخله کمتر از قبل از مداخله بود (جدول ۴-۱۳).

۱) مقایسه میانگین تغییرات خستگی در بیماران، در طول مداخله بین گروه در بیماران تحت همودیالیز در بیمارستان‌های شهر بوشهر در سال ۱۳۹۹

جدول ۴-۱۴. مقایسه میانگین تغییرات خستگی در بیماران، در طول مداخله بین گروه در بیماران تحت همودیالیز در بیمارستان‌های شهر بوشهر در سال ۱۳۹۹

گروه	آزمون<br>Mean±SD	کنترل<br>Mean±SD	t	p-value
**تغییرات خستگی**	۰/۶۵±۰/۳۵	-۰/۰۰۷±۰/۴۵	۷/۲۲	۰/۰۰۱

طبق نتایج آورده شده در جدول ۴-۱۴، مقایسه میانگین تغییرات خستگی بیماران بین گروه آزمون و کنترل نشان داد که تفاوت معناداری بین دو گروه وجود دارد (p=۰/۰۰۱)؛ به طوریکه تغییرات خستگی بیماران در گروه آزمون بیشتر از گروه کنترل بود و افزایش یافته بود (جدول ۴-۱۴).

جدول ۴-۱۵. تعیین و مقایسه سطح خستگی در بیماران قبل و بعد از مداخله بین دو گروه آزمون و کنترل در بیمارستان‌های شهر بوشهر در سال ۱۳۹۹

متغیر		آزمون	کنترل	کل	X^2	P- value
		تعداد (درصد)	تعداد (درصد)	تعداد (درصد)		
سطح خستگی قبل از مداخله	متوسط	۲۳(۷۶/۷)	۱۲(۳۷/۵)	۳۵(۵۶/۵)	۹/۶۶	۰/۰۰۲
	شدید	۷(۲۳/۳)	۲۰(۶۲/۵)	۲۷(۴۳/۵)		
سطح خستگی بعد از مداخله	متوسط	۱۷(۸۵/۰)	۱۱(۳۶/۷)	۲۸(۵۶/۰)	۱۱/۳۸	۰/۰۰۱
	شدید	۳(۱۵/۰)	۱۹(۶۳/۳)	۲۲(۴۴/۰)		

طبق نتایج آورده شده در جدول ۴-۱۵؛ سطح خستگی بیمار قبل از مداخله (p=۰/۰۰۲) و در بعد از اتمام مداخله (p=۰/۰۰۱) بین گروه آزمون و کنترل تفاوت معناداری را نشان داد. به طوریکه در قبل از مداخله، فراوانی خستگی اکثریت بیماران در گروه آزمون متوسط (٪۷۶/۷) و در گروه کنترل شدید (٪۶۲/۵) بوده است. در بعد از مداخله، فراوانی خستگی اکثریت بیماران در گروه آزمون متوسط (٪۸۵) و در گروه کنترل شدید (٪۶۳/۳) بوده است.

فصل پنجم

بحث و نتیجه‌گیری

۵-۱ بحث و بررسی یافته‌ها و پیشنهادات

در این فصل به بحث و نتیجه‌گیری پیرامون یافته‌های پژوهش پرداخته شده است و کاربرد یافته‌های پژوهش در پرستاری و پیشنهادات برای مطالعات آینده ارائه شده است.

شیوع بیماری‌های غیر واگیر و بروز عوارض ناشی از این بیماری‌ها، سیاست‌گذاران عرصه سلامت را بیش از پیش متوجه نارسایی مزمن کلیوی و درمان‌های جایگزین کرده است(۹۵). بیماری مزمن کلیه؛ اختلال برگشت‌ناپذیر کلیه بوده که بیش از سه ماه طول کشیده است و با از دست دادن دائمی عملکرد کلیه‌ها مشخص می‌شود. این کاهش عملکرد کلیه منجر به تجمع مواد زاید متابولیک، اختلالات الکترولیتی و مواد معدنی در بدن می‌گردد (۱۶۰). همودیالیز، یکی از درمان‌های مهم و رایج برای بیماران مبتلا به ESRD، محسوب می‌شود. بیماران تحت درمان با همودیالیز به علت نارسایی کلیه و عوارض مربوط به درمان، همواره با مشکلات فراوانی دست و پنجه نرم می‌کنند. از جمله این مشکلات می‌توان به اختلالات الکترولیتی، خستگی، افت فشارخون، اسپاسم عضلانی، مشکلات گوارشی، خارش، کاهش عملکرد جنسی و کیفیت زندگی جنسی، اشاره کرد که هرکدام از این مشکلات به‌نوبه خود تأثیرات منفی بر کیفیت زندگی بیماران تحت همودیالیز می‌گذارند (۱۶۱). در همین راستا مطالعه حاضر به عنوان یک پژوهش تجربی بر آن بوده است تا به مقایسه اثربخشی فعالیت فیزیکی حین دیالیز بر کفایت دیالیز، شاخص‌های همودینامیک و خستگی در بیماران تحت همودیالیز در بیمارستان‌های شهر بوشهر در سال ۱۳۹۹ بپردازد. تعداد ۸۴ بیمار (۴۲ آزمون/۴۲ کنترل) وارد مطالعه شدند. پس از شروع مطالعه دو نفر در گروه آزمون و سه نفر در گروه از مطالعه خارج شدند و درنهایت اطلاعات ۴۰ نفر در گروه آزمون و ۳۹ نفر در گروه کنترل

تجزیه‌وتحلیل شد.

تجزیه و تحلیل یافته‌ها در رابطه با هدف اول پژوهش «تعیین ویژگی‌های جمعیت شناختی در دو گروه آزمون و کنترل در بیمارستان‌های شهر بوشهر در سال ۱۳۹۹» که در جداول (۴-۱) ارائه شده است، نشان داد که اکثر بیماران مرد و متأهل بودند. نیمی از بیماران شاغل و بیش از شصت درصد آنها دارای تحصیلات زیر دیپلم بودند. تفاوت بین بیماران دو گروه مطالعه از نظر وضعیت تأهل و شغل معنی‌دار نبوده است اما میانگین سن گروه بیماران کنترل به صورت معنی‌داری بیشتر از گروه آزمون بود (p=۰/۰۰۱). همچنین بین بیماران گروه کنترل و آزمون از نظر تحصیلات تفاوت معناداری وجود داشت (p=۰/۰۰۶).

تجزیه و تحلیل یافته‌ها در رابطه با هدف دوم " تعیین و مقایسه میزان اوره خون و وزن در بیماران قبل و بعد از مداخله در دو گروه آزمون و کنترل به تفکیک در بیماران تحت همودیالیز در بیمارستان‌های شهر بوشهر در سال ۱۳۹۹" و هدف سوم پژوهش " تعیین و مقایسه میزان اوره خون و وزن در بیماران قبل و بعد از مداخله بین دو گروه آزمون و کنترل در بیمارستان‌های شهر بوشهر در سال ۱۳۹۹ " و هدف چهارم " مقایسه میانگین تغییرات اوره خون و وزن در بیماران در طول مداخله بین دو گروه آزمون و کنترل در بیمارستان‌های شهر بوشهر در سال ۱۳۹۹" که در جدول (۴-۲) و جدول (۴-۳) ارائه شده است، نشان داد که بین دو گروه آزمون و کنترل، از نظر میزان اوره خون بیماران تفاوت وجود ندارد که این گویای مؤثر نبودن فعالیت فیزیکی حین دیالیز بر اوره خون بیماران تحت همودیالیز بوده است. مرور مطالعات پیشین نتایج ضدونقیضی را نشان داد. در مطالعات مشابه با پژوهش حاضر، وایتی‌لینگهام و همکاران (۲۰۰۴)(۷۵) فعالیت فیزیکی و ورزش را بر میزان اوره خون بیماران مؤثر نداتسته‌اند. در این راستا وایتی‌لینگهام و همکاران (۲۰۰۴)(۷۵) در استرالیا بر روی بیماران همودیالیزی مزمن نشان دادند که تمایل به افزایش اوره خون در گروه فعالیت ورزشی بیشتر بوده اما تفاوت معناداری وجود نداشته است؛ اما برخلاف نتایج (URR) پژوهش حاضر، مطالعه ژائو[1] و همکاران (۲۰۲۰) و سوکال[2] و همکاران (۲۰۱۳)، نشان دهنده تأثیر تمرینات ورزشی، بر میزان اوره خون بیماران همودیالیزی بود(۱۶۲, ۱۶۳). به نظر می‌رسد که با فعالیت حین دیالیز،

[1] Zhao
[2] Sokal

جریان خون و پرفیوژن عضلات افزایش یافته و میزان مبادله بین خون و عضلات افزایش می‌یابد. به دنبال این افزایش تبادل، مواد زائد بیشتری مانند اوره می‌توانند از عضلات وارد گردش خون شوند و از طریق دیالیز دفع شوند و در طول انجام فعالیت حین دیالیز از گردش خون حذف گردند(۴۲, ۴۳). نتایج پژوهش حاضر نشان داد که میانگین نیتروژن اوره خون(BUN) قبل از مداخله و پس از مداخله بین گروه آزمون و کنترل و میزان تغییر نمره قبل به بعد در گروه آزمون و کنترل؛ تفاوت معنادار نبود که این نتیجه، گویای مؤثر نبودن فعالیت فیزیکی حین دیالیز بر BUN بیماران تحت همودیالیز بوده است. در یک مطالعه مشابه، مداخله تمرین ورزشی در مطالعه سلطانی وهمکاران(۲۰۱۹) بر BUN بیماران همودیالیزی مؤثر نبوده است. مداخله ورزشی به صورت حداقل ۳۰ دقیقه دوچرخه‌سواری در طول هر جلسه همودیالیز به مدت شش ماه بوده است.(۱۶۴). بر خلاف نتایج پژوهش حاضر، ژائو و همکاران(۲۰۲۰) و سوکال[1] و همکاران(۲۰۱۳) و سایود[2] و همکاران(۲۰۲۱)(۱۶۵)، فعالیت ورزشی را بر کاهش میانگین نیتروژن اوره خون(BUN) در بیماران همودیالیزی نشان دادند. در مطالعه فوران[3] و همکاران(۲۰۰۳)، سطح BUN، ۴ ساعت پس از ورزش در نتیجه کم‌آبی بدن و کاهش پرفیوژن کلیه افزایش یافته است(۱۶۶). نتایج پژوهش حاضر نشان داد که میانگین وزن بیمار قبل از مداخله و پس از مداخله بین گروه آزمون و کنترل تفاوت معناداری وجود نداشت اما در گروه آزمون و کنترل، میانگین وزن بیمار پس از مداخله به طور معنی‌داری کمتر از قبل از مداخله بود. برخلاف نتایج پژوهش حاضر پژوستوتی[4] و همکاران(۲۰۲۰)(۱۶۷) و راولی و همکاران (۲۰۲۱) (۱۶۸)، ورزش را در کاهش وزن بیماران همودیالیزی مؤثر دانسته‌اند. می‌توان چنین استنباط کرد که پیروی از فعالیت فیزیکی برای برخی افراد مشکل است و از طرفی، فاکتورهای مخدوش‌کننده‌ی متعددی (همچون سن، جنس، بیماری‌های زمینه‌ای و غیره) می‌توانند شاخص‌های آزمایشگاهی خون بیماران را تحت‌الشعاع خود قرار دهند(۱۶۹) و احتمالاً به همین دلیل، نتایج مطالعات متفاوت است.

---

[1] Sokal
[2] Saud
[3] Foran
[4] Pujiastuti

تجزیه و تحلیل یافته‌ها در رابطه با هدف پنجم " تعیین و مقایسه Kt/V در بیماران بعد از مداخله بین دو گروه آزمون و کنترل در بیمارستان‌های شهر بوشهر در سال ۱۳۹۹" که در جدول (۴-۴) ارائه شده است، نشان داد که تفاوت معناداری بین گروه آزمون و کنترل از نظر میانگین Kt/V بیماران وجود ندارد؛ که این نتیجه گویای مؤثر نبودن فعالیت فیزیکی حین دیالیز بر Kt/V بیماران تحت همودیالیز بوده است. نتایج مطالعات انجام شده درگذشته نتایج ضد ونقیصی را نشان داده است. در مطالعات مشابه، شفیع پور (۱۳۹۶)، بر روی بیماران دیالیزی در استان مازندران نشان دادند که تمرینات ورزشی حین دیالیز و پیاده‌روی در منزل بر توانایی جسمانی بر کفایت دیالیز بیماران همودیالیزی، مؤثر نبوده است. این مطالعه روی بیماران مزمن بیش از ۳ ماه دیالیز و با مداخله دو هفته فعالیت حین دیالیز در ابتدای همودیالیز و ۶ هفته پیاده‌روی در منزل انجام شد(۷۴). در مطالعه‌ای دیگر، کرکمن (۲۰۱۳) در ولز انگلستان، فعالیت را دارای اثر معناداری روی تغییر و بهبود کفایت دیالیز نسبت به زمان دیالیز معمولی نشان نداد ولی افزایش زمان در آن‌ها نسبت به زمان دیالیز معمولی، باعث بهبود کفایت دیالیز (Kt/V) گردید و بر این نکته تاکید کردند که انجام فعالیت حین دیالیز نمی‌تواند جایگزین افزایش زمان دیالیز گردد و نمی‌تواند باعث بهبود کفایت دیالیز گردد(۷۱). در مطالعه ریاحی (۱۳۹۱) تأثیر تمرینات ورزشی منظم بر کیفیت دیالیز، بیماران همودیالیزی در بیمارستان شریعتی اصفهان مؤثر ندانسته‌اند و تفاوت معناداری در کفایت دیالیز دیده نشد (۷۳). این پژوهش در بیماران با نارسایی مزمن کلیوی تحت درمان با همودیالیز بالاتر از ۳ سال و مداخله به صورت ۲۰ هفته تمرینات ورزشی با استفاده از دوچرخه در حین دیالیز و در ۲ ساعت ابتدایی دیالیز انجام شده است. از علل مطرح در کاهش کفایت دیالیز می‌توان به تنگی انتهای وریدی فیستول شریانی وریدی، فیستول نامناسب و ضعیف، عدم رعایت برنامه دیالیز منظم از طرف بیمار، بیماری‌های قلبی عروقی، ناپایداری همودینامیک، عفونت‌ها، تغذیه نامناسب، سیرکولاسیون مجدد اشاره کرد(۶۳).

اما این نتایج با نتایج مطالعه نصیری زاده و همکاران (۱۳۹۸)، بصیری مقدم و همکاران (۲۰۱۶)، ریاحی (۱۳۹۱) و پارسونز (۲۰۰۶) در تناقض است. نصیری زاده و همکاران (۱۳۹۸) در بیماران تحت درمان با همودیالیز، نشان دادند که آموزش و اجرای تکنیک آرام‌سازی عضلانی بنسون باعث افزایش کفایت

دیالیز شده است(۱۴۷). این تکنیک از طریق ایجاد تعادل بین هیپوتالاموس خلفی و قدامی و کاهش فعالیت سیستم عصبی سمپاتیک (کاهش فشارخون) باعث کاهش ترشح کانکولآمین ها و استرس می‌شود و از این‌رو انتظار می‌رود کفایت دیالیز که تحت تأثیر مؤلفه‌های فوق است، افزایش یابد. در مطالعه‌ای دیگر، بصیری مقدم و همکاران(۲۰۱۶) نشان دادند که انجام حرکات توأم ایزوتونیک و ایزومتریک حین همودیالیز میزان کفایت دیالیز بیماران همودیالیزی را بهبود می‌بخشد(۱۴۹). مداخله آنها تمرینات توأم ایزوتونیک و ایزومتریک به مدت ۶۰ دقیقه در ۲ ساعت اول همودیالیز و سه بار در هفته به مدت ۶ هفته بوده است. پارسونز (۲۰۰۶) نیز فعالیت به صورت پدال زدن حین دیالیز به مدت ۶۰ دقیقه در ۲ ساعت ابتدایی دیالیز را در بالا بردن کفایت دیالیز مؤثر می‌داند(۴۳). علت تفاوت می‌تواند در ماهیت و مدت زمان فعالیت بدنی و متغیر بودن شدت و دفعات انجام برنامه ورزشی(۱۷۰)، در مطالعات ذکر شده دانست که با پژوهش حاضر متفاوت بوده است و می‌تواند در افزایش کفایت دیالیز مؤثر باشد. یکی دیگر از علل تفاوت را می‌توان در نحوه محاسبه کفایت دیالیز دانست. در پژوهش حاضر محاسبه کفایت دیالیز براساس (Kt/V) محاسبه شده است این درحالیست که در بعضی از مطالعات با فرمول URR محاسبه شده است. در پژوهش حاضر براساس پژوهش‌های گذشته، نحوه انجام فعالیت فیزیکی بیمار به صورت پدال زدن در بستر به مدت ۱۵ دقیقه در ۲ نوبت و با در نظر گرفتن ۱۵ دقیقه استراحت در بین تمرینات و به مدت ۴ هفته و در هر هفته ۳ جلسه بوده است (۱۵۸, ۱۵۹). با این حال در بعضی از مطالعات، ۳ دوره زمانی ۱۵ دقیقه‌ای را برای برداشت اوره سرم ناکافی دانسته‌اند و ۲ دوره زمانی ۳۰ دقیقه‌ای ورزش را باعث افزایش برداشت اوره به واسطه مایع دیالیز گزارش کرده‌اند. از طرفی دیگر، بسیاری از بیماران در ساعت سوم دیالیز به علت هاپیوتانسیون قادر به ورزش کردن نیستند(۴۳)؛ اما در مطالعه، بصیری مقدم وهمکاران(۲۰۱۶) انجام ورزش ۶۰ دقیقه در ۲ ساعت اول دیالیز باعث افزایش معنی‌داری در کفایت دیالیز بیماران شده بود(۱۴۹).

تجزیه و تحلیل یافته‌ها در رابطه با هدف ششم " تعیین و مقایسه شاخص‌های همودینامیک (درجه حرارت، نبض، فشارخون، تنفس و درصد اشباع اکسیژن خون شریانی) در بیماران قبل و بعد از مداخله در هر دو گروه آزمون و کنترل به تفکیک در بیمارستان‌های شهر بوشهر در سال ۱۳۹۹" و هدف هفتم "

تعیین و مقایسه شاخص‌های همودینامیک (درجه حرارت، نبض، فشارخون، تنفس و درصد اشباع اکسیژن خون شریانی) در بیماران قبل و بعد از مداخله بین دو گروه آزمون و کنترل در بیمارستان‌های شهر بوشهر در سال ۱۳۹۹" و هدف هشتم " مقایسه میانگین تغییرات شاخص‌های همودینامیک (درجه حرارت، نبض، فشارخون، تنفس و درصد اشباع اکسیژن خون شریانی) در بیماران در طول مداخله بین گروه آزمون و کنترل در بیمارستان‌های شهر بوشهر در سال ۱۳۹۹" که در جدول (۴-۵) تا (۴-۱۹) ارائه شده است، نشان داد که با تعدیل اثر سن و تحصیلات، اثر شاخص‌های همودینامیک (درجه حرارت، نبض، فشارخون، تنفس و درصد اشباع اکسیژن خون شریانی) معنی‌دار بوده است ولی تنها اثر متقابل فشارخون سیستول * گروه و اثر متقابل فشارخون دیاستول * گروه تفاوت معنی‌دار را نشان داد؛ اما اثر درصد اشباع اکسیژن و اثر متقابل درصد اشباع اکسیژن * گروه معنی‌دار نبوده است. به طور کلی تفاوت معناداری بین دو گروه از نظر میانگین تغییرات شاخص‌های همودینامیک (درجه حرارت، نبض، فشارخون، تنفس و درصد اشباع اکسیژن خون شریانی) وجود نداشت. این نتیجه نشان دهنده ثبات در شاخص‌های همودینامیک در طول زمان مداخله بوده است.

در زمینه بررسی اثرات فعالیت فیزیکی در بیماران همودیالیزی و تأثیر آن بر شاخص‌های همودینامیک مطالعات اندکی انجام شده است. در مطالعاتی مشابه، هنریک[1] و همکاران (۲۰۱۰) نشان دادند که تمرینات هوازی در جلسات همودیالیز به بهبود ظرفیت فیزیکی و کنترل فشارخون بالا در افراد مبتلا به نارسایی مزمن کلیه کمک می‌کند(۱۷۱). ارسی[2] وهمکاران(۲۰۱۴) نیز ۸ هفته تمرین ورزشی را در کاهش فشارخون سیستول مؤثر دانسته‌اند(۱۷۲). در یک مطالعه ایرانی، تمرین ورزشی می‌تواند منجر به کاهش چشمگیر فشارخون سیستولیک و دیاستولیک در بیماران همودیالیزی شود(۱۶۴). اگرچه نتایج فوق با نتایج ما مطابقت داشت، اما در مطالعات انجام شده توسط سلحاب و همکاران(۱۷۳) و پارسونز و همکاران(۴۳) تفاوت معنی‌داری در میانگین فشارخون بیماران مشاهده نشد. این ممکن است به دلیل نوع، شدت و مدت زمان مختلف تمرین در مطالعه ما باشد. از طرفی دیگر، آزمایشات بالینی سیستم گردش

[1] Henrique
[2] Orcy

خون در بیماران همودیالیزی ثبت شده است که ورزش تأثیرات مطلوبی بر عملکرد قلب دارد، تعادل را بر سیستم عصبی خودکار قلب تسهیل می‌کند و به مدیریت فشارخون شریانی کمک می‌کند(۱۷۴).

با توجه به اینکه مشکلات قلبی و عروقی و فشارخون بالا عوامل اصلی ابتلای افراد به مشکلات کلیوی هستند که در نتیجه بر تغییرپذیری ضربان قلب[1] افراد تأثیر می‌گذارد. تغییرات ضربان قلب در طول مداخله فعالیت فیزیکی در پژوهش حاضر ثابت بوده است و این نتیجه می‌تواند قابل توجه باشد. برخلاف نتایج پژوهش حاضر، موریس و همکاران (۲۰۱۹) نشان دادند که تمرینات ورزشی هوازی در طی همودیالیز باعث افزایش کنترل خودکار ضربان قلب در بیماران مبتلا به بیماری مزمن کلیه نشده است؛ زیرا بیماران مبتلا به CKD عملکردهای متعددی را به خطر می‌اندازند و همچنین در مورد غذا و فعالیت فیزیکی محدودیت‌هایی در مقایسه با افراد سالم دارند و توسعه برنامه‌های تمرینات بدنی دشواری بیشتری را برای بیمار ایجاد می‌کند(۱۷۵).

تجزیه‌وتحلیل یافته‌ها در رابطه با هدف نهم " تعیین و مقایسه میزان خستگی در بیماران، قبل و بعد از مداخله در هر دو گروه آزمون و کنترل به تفکیک در بیماران تحت همودیالیز در بیمارستان‌های شهر بوشهر در سال ۱۳۹۹" و هدف دهم " تعیین و مقایسه میزان خستگی در بیماران قبل و بعد از مداخله بین دو گروه آزمون و کنترل در بیمارستان‌های شهر بوشهر در سال ۱۳۹۹ " و هدف یازدهم " مقایسه میانگین تغییرات خستگی در بیماران، در طول مداخله بین گروه در بیماران تحت همودیالیز در بیمارستان‌های شهر بوشهر در سال ۱۳۹۹" که در جدول (۴-۲۰) تا (۴-۲۲) ارائه شده است، نشان داد که مقایسه میانگین تغییرات خستگی بیماران بین گروه آزمون و کنترل نشان داد که تفاوت معناداری بین دو گروه وجود داشت. به‌طوری که تغییرات خستگی بیماران در گروه آزمون بیشتر از گروه کنترل بود و افزایش یافته بود. خستگی اکثریت بیماران در گروه آزمون متوسط (۷۶/۷٪) و در گروه کنترل شدید (۶۲/۵٪) بوده است. در بعد از مداخله، فراوانی خستگی اکثریت بیماران در گروه آزمون متوسط (۸۵٪) و در گروه کنترل شدید (۶۳/۳٪) بوده است. جابلونسکی گزارش داد که ۶۹ تا ۷۷ درصد از بیماران همودیالیزی از خستگی

[1] Heart rate variability

رنج می‌بردند و نیاز به مداخلات غیر دارویی داشتند. بر اساس مطالعه لشکری و همکاران (۱۳۹۵)، در ایران، ۴۲، ۳۶ درصد از بیماران همودیالیزی به ترتیب از خستگی زیاد، متوسط رنج می‌بردند(۱۳۹).

در مطالعاتی مشابه، شایانی ممتاز و همکاران (۱۳۹۷) اجرای برنامه توان‌بخشی در کاهش خستگی بیماران همودیالیزی مؤثر است؛ و از آن می‌توان به عنوان یک روش ساده، غیرتهاجمی، کم‌هزینه و مؤثر در کاهش خستگی بیماران همودیالیزی دانسته‌اند (۱۴۸). برای گروه مداخله اجرای برنامه‌ی توان‌بخشی شامل انجام ماساژ کف پا در حین همودیالیز به مدت ۱۰ دقیقه در ۱۲ جلسه حین همودیالیز به طول مدت یک ماه همراه با آموزش الگوی فعالیت روزانه، آموزش رژیم غذایی و داروها بوده است. ششادری[1] و همکاران (۲۰۱۹)، فعالیت بدنی بالاتر با خستگی کمتر در بین بیماران مبتلا به همودیالیز همراه بوده است(۱۵۲). به‌طوری که به ازا هر ۱۰۰۰ قدم در روز، ۰/۲ واحد از شدت خستگی آنها کم شده است (۱۷۶). در مطالعه شاعرمقدم و همکاران(۱۳۹۴)، ماساژ بازتابی کف دست به مدت هفته‌ای ۲ بار و هر جلسه ۲ دقیقه و به مدت ۲ هفته می‌تواند به عنوان روشی کم‌هزینه و ایمن برای کاهش خستگی در بیماران همودیالیزی مورد استفاده قرار گیرد(۱۷۷). متدین(۱۳۹۳)، اثر فعالیت حین دیالیز در بیماران کلیوی تحت درمان با همودیالیز را در کاهش میزان خستگی در بیماران مؤثر دانسته‌اند(۶۳). صالحی وهمکاران(۲۰۲۰) نیز توانبخشی از طریق ورزش دو بار در هفته به مدت ۳ ماه ورزش به مدت ۲۰ دقیقه با دوچرخه کوچک، دارای تأثیر قابل توجهی در جلوگیری از افزایش خستگی بیشتر در بیماران همودیالیزی دانسته‌اند(۱۴۶).

البته در بعضی دیگر ازمطالعات تأثیر مداخله ورزشی بر آمادگی جسمانی بیماران دیالیز بررسی شده و مطالعات نشان دهنده افزایش آمادگی جسمانی در بیماران دیالیز بوده است. از جمله:

دیزیوبک[2] و همکاران (۲۰۱۵)، تأثیر یک برنامه تمرینی بدنی ۳ ماهه ورزش‌های آبی بر آمادگی جسمانی و عملکرد عضلات در بیماران دیالیز مؤثر دانسته‌اند(۱۵۳). ریاحی (۱۳۹۱) تمرینات ورزشی منظم، آتروفی عضلانی و عملکرد جسمانی بیماران همودیالیزی در حین دیالیز را بهبود بخشیده است(۷۳). در پژوهش گروسارد (۲۰۱۴)، با انجام فعالیت هوازی و پدال زدن در دو ساعت ابتدایی دیالیز در بیماران

[1] Sheshadri
[2] Dziubek

مزمن کلیوی می‌تواند اختلالات همراه با این بیماری را کاهش دهد و آمادگی جسمانی آنها را ارتقا بخشید(۳). با بررسی این مطالعات مشخص می‌شود که انواع برنامه‌های آموزشی و مداخله‌ای بر روی خستگی در بیماران همودیالیز انجام گرفته است که از نظر زمان، محیط پژوهش، جامعه و حتی تعداد نمونه و محتوا و تعداد جلسات مداخله متفاوت می‌باشند اما در کل می‌توان استدلال کرد انجام مطالعاتی که با مداخله فعالیت فیزیکی فعالیت فیزیکی در کاهش خستگی در بیماران مؤثر بوده است. در برخی از مطالعات نتایج متناقضی گزارش شده است از جمله: ریتبرگ[1] و همکاران(۲۰۱۴) برنامه توان‌بخشی چند رشته‌ای در کاهش خستگی مزمن بیماران مبتلا به مولتیپل اسکلروزیس مؤثر نبود و ممکن است خستگی مزمن در بیماران مبتلا به ام اس با توجه به انواع مداخلات و در طول زمان بسیار متفاوت باشد(۱۷۸)؛ بنابراین به نظر می‌رسد که تفاوت در زمان و مکان مطالعه، ابزارهای اندازه‌گیری و سنجش میزان خستگی در مطالعات و ماهیت بیماری‌های مزمن و میزان خستگی ناشی از آنها هم متفاوت است؛ اما به طور کل می‌توان گفت که اجرای برنامه‌های مختلف مانند انواع ماساژها از جمله ماساژ کف پا، تمرینات ورزشی، پیاده‌روی و فعالیت فیزیکی می‌تواند در کاهش خستگی بیماران مزمن و به‌ویژه در بیماران تحت همودیالیز مؤثر باشد.

۵-۲. نتیجه گیری نهایی

نتایج مطالعه حاکی از آن است که فعالیت فیزیکی حین دیالیز بر کفایت دیالیز و تغییرات شاخص‌های همودینامیک در بیماران تحت همودیالیز در بیمارستان‌های شهر بوشهر در سال ۱۳۹۹ مؤثر نبوده است؛ اما فعالیت فیزیکی حین دیالیز موجب شده بود که خستگی بیماران تحت همودیالیز افزایش یابد. از طرفی دیگر فشارخون سیستول و دیاستول در گروه‌های مطالعه متفاوت بوده است اما تغییرات آن‌ها در بیماران تحت همودیالیز معنی‌دار نبوده است. لذا پیشنهاد می‌شود که با انجام آموزش به پرسنل و بیمار می‌توان میزان فشارخون این بیماران را کنترل و به حالت طبیعی برگرداند. لذا به نظر می‌رسد با توجه به ثبات تغییرات شاخص‌های همودینامیک در بیماران تحت همودیالیز فعالیت فیزیکی می‌تواند به عنوان یک

---
[1] Rietberg

مداخله در بهبود شرایط بیماران تحت همودیالیز در نظر گرفته شود اما با توجه به عدم تأثیرگذاری بر کفایت دیالیز و افزایش خستگی بیماران همودیالیز، بایستی به نوع مداخله، مدت و زمان آن توجه ویژه‌ای شود. در این پژوهش مداخله براساس مدل انجام نشده است لذا پیشنهاد می‌شود در مطالعات آتی مداخلات فعالیت فیزیکی برمبنای مدل‌های توانبخشی مانند طب مکمل انجام شود.

۵-۳. کاربرد نتایج

۵-۳-۱. کاربرد نتایج در بالین

با توجه به عدم اثربخشی مداخله فعالیت فیزیکی حین دیالیز بر کفایت دیالیز و افزایش خستگی بیماران تحت همودیالیز، به‌کارگیری این شیوه توسط مربیان پزشکی و پرستاری در محیط‌های بالینی نیازمند بررسی‌های بیشتر و تغییر در نوع و مدت زمان مداخله می‌باشد.

۵-۳-۲. کاربرد نتایج در مدیریت

- مدیران و مسئولین دانشکده‌های پرستاری و پزشکی، می‌توانند جهت آشنایی مربیان بالین دوره‌هایی را برگزار نمایند تا آنها با مداخلاتی آشنا شوند که موجب خستگی کمتر در بیماران شده است.
- مدیران و برنامه‌ریزان نظام سلامت می‌توانند از افراد متخصص که دوره‌های حرفه‌ای فعالیت فیزیکی حین دیالیز را گذرانده‌اند در آموزش استفاده نمایند.
- با توجه به متفاوت بودن شرایط بیماران همودیالیز، نتایج مطالعه حاضر می‌تواند توسط مسئولین دانشکده‌های مربوطه به‌عنوان یک راهنما مورد استفاده قرار گیرد که ممکن است هرنوع فعالیت فیزیکی مناسب بیماران همودیالیز نمی‌باشد.

۵-۳-۳. کاربرد نتایج در آموزش

- آموزش مداوم، دوره‌هایی در راستای آشنایی و آگاهی از مداخلات کاهش‌دهنده خستگی بیماران دیالیز برای دانشجویان پرستاری و پزشکی در نظر گرفته شود.

۵-۴. محدودیت‌های مطالعه

هر تحقیق و پژوهشی در بطن خود با یکسری مشکلات و محدودیت‌ها مواجه خواهد بود. این محدودیت‌ها ممکن است بر سر راه تحقیق ظاهر شود و امر تحقیق را دچار مشکلاتی نماید. پژوهش حاضر نیز دارای محدودیت‌هایی بود که توجه به آنها میزان تعمیم و تکیه بر گستره یافته‌ها را هموار خواهد نمود. از محدودیت‌های پژوهش حاضر به موارد ذیل می‌توان اشاره داشت.

- حجم پایین تعداد نمونه‌ها که خود می‌تواند در نتایج مطالعه تأثیرگذار باشد.
- اجرای دشوار فعالیت فیزیکی حین دیالیز
- امتناع از تکمیل کردن پرسشنامه‌ها به علت صرف زمان و کاهش صبر و تحمل بیمار و همراهان که محقق با توضیح بیشتر در اهداف سعی در جلب همکاری آنها نمود.
- محدودیت جهت افزایش زمان همودیالیز، به علت سیکل‌های پیاپی دیالیز و عدم تحمل بیماران که محقق با هماهنگ کردن زمان سیکل‌ها و جلب رضایت بیماران، سعی در انجام صحیح آن داشت.
- متفاوت بودن ماشین‌های همودیالیز در بخش‌های مختلف احتمال تأثیر گذاری بر کیفیت دیالیز را مطرح می‌نماید؛ که به دلیل تعداد زیاد بیماران امکان انجام دیالیز آنان توسط دستگاه‌های یکسان وجو ندارد؛ لیکن پژوهشگر سعی شد با به‌کارگیری دستگاه‌های یکسان در جلسات دیالیز هر بیمار و همچنین قید نمودن نوع دستگاه به کار گرفته شده در فرم اطلاعات جمعیت شناختی بیماران به تبیین تفاوت‌ها بپردازد.

۵-۵. پیشنهادات پژوهشی برای مطالعات آینده

گزارش هر پژوهشی به امید ادامه یافتن راه تحقیق و پژوهش در مورد آن موضوع نگارش و به جامعه پژوهشگران ارائه شود، بنابراین لازمه هر گزارش پیشنهاداتی است که راه را برای پژوهش‌های آتی هموار سازد. این پژوهش نیز از این امر مستثنی نبوده و پیشنهاداتی به شرح زیر ارائه می‌شود:

۱) بررسی اثربخشی طب مکمل در کنار فعالیت فیزیکی بر کفایت دیالیز، شاخص‌های همودینامیک

و خستگی در بیماران تحت همودیالیز

۲) بررسی موانع اجرای فعالیت فیزیکی مؤثر بر کفایت دیالیز، شاخص‌های همودینامیک و خستگی در بیماران تحت همودیالیز

۳) بررسی اثربخشی فعالیت فیزیکی با اثر واسطه‌ای رژیم غذایی بر کفایت دیالیز، شاخص‌های همودینامیک و خستگی در بیماران تحت همودیالیز بر کفایت دیالیز، شاخص‌های همودینامیک و خستگی

۴) مقایسه تأثیر انواع فعالیت فیزیکی بر کفایت دیالیز، شاخص‌های همودینامیک و خستگی در بیماران تحت همودیالیز

۵) مقایسه اثربخشی فعالیت فیزیکی حین دیالیز بر کفایت دیالیز، شاخص‌های همودینامیک و خستگی در بیماران تحت همودیالیز با استفاده از ابزارهای اندازه‌گیری و سنجش مختلف

منابع و مآخذ

Reference

1- Malekzadeh R, etal. newsresearchacir/content/529/2016 [Internet]. USA: Institute of Health Metrics and Evaluation. 2017. [cited 2018].

2- Maghsoudi A, Abedi K, Omidvari joo F. The study of prevalence of chronic diseases and its association with quality of life in the elderly of Ewaz (South of Fars province). Navid Nouve J. 2014;18(61):35-41.

3- Groussard C, Rouchon-Isnard M, Coutard C, Romain F, Malarde L, Lemoine-Morel S, et al. Beneficial effects of an intradialytic cycling training program in patients with end-stage kidney disease. Applied physiology, nutrition, and metabolism = Physiologie appliquee, nutrition et metabolisme. 2015;40(6):550-6.Doi:10.1139/apnm-2014-0357.

4- Brian Lee, Turley M. Effects of proactive population-based nephrologist oversight on progression of chronic kidney disease: a retrospective control analysis. 2012.

5- Shojamoradi MH, Saberi Isfeedvajani M, Mahdavi-Mazdeh M, Ahmadi F, Gatmiri SM, Abbasi Larki R. Chronic Kidney Disease Progression in Elderly Iranian Patients: A Cohort Study. Nephrourol Mon. 2014;6(5):e20748.Doi:10.5812/numonthly.20748.

6- Sepanlou SG, Barahimi H, Najafi I, Kamangar F, Poustchi H, Shakeri R, et al. Prevalence and determinants of chronic kidney disease in northeast of Iran: Results of the Golestan cohort study. PLoS One. 2017;12(5):e0176540.Doi:10.1371/journal.pone.0176540.

7- Sautenet B, Tong A, Williams G, Hemmelgarn BR, Manns B, Wheeler DC, et al. Scope and Consistency of Outcomes Reported in Randomized Trials Conducted in Adults Receiving Hemodialysis: A Systematic Review. American journal of kidney diseases: the official journal of the National Kidney Foundation. 2018;72(1):62-74.Doi:10.1053/j.ajkd.2017.11.010.

8- Hadi N, Rahmani Z. Health-related quality of life in patients with chronic renal failure under hemodialysis. 2009.

9- Nafar M, Samavat S, Khoshdel A, Alipour Abedi B. Dialysis Adequacy, Dialyzer Clearance, and Strategies to Achieve Target: A Nationwide Multicenter Study. Nephro-Urology Monthly. 2016; 9(1) Doi:10.5812/numonthly.42769.

10- Morfin JA, Fluck RJ, Weinhandl ED, Kansal S, McCullough PA, Komenda P. Intensive Hemodialysis and Treatment Complications and Tolerability.

American journal of kidney diseases: the official journal of the National Kidney Foundation. 2016;68(5S1):S43-S50.Doi:10.1053/j.ajkd.2016.05.021.

11- Iran CoDo. Calender of Dialysis of Iran, 2017. In: Iran CoDo, editor. 1st ed: Cosersium of Dialysis of Iran; 2017. p. 5-16.

12- Alavi NM, Aliakbarzadeh Z, Sharifi K. Depression, Anxiety, Activities Of Daily Living, And Quality Of Life Scores In Patients Undergoing Renal Replacement Therapies. Transplant Proc. 2009;41(9):3693-6.Doi:10.1016/j.transproceed.2009.06.217.

13- Mollazadeh F, Hemati MM. The Comparison Of Self-Steem Of Patients Undergoing Hemodialysis With Kidney Reanal Transplantation Recipients. 2017.

14- Gentile S, Beauger D, Speyer E. Factors associated with health related quality of life in renal transplant recipients results of a national survey in France. 2013.

15- Wong B, Ravani P, Oliver MJ, Holroyd-Leduc J, Venturato L, Garg AX, et al. Comparison of Patient Survival Between Hemodialysis and Peritoneal Dialysis Among Patients Eligible for Both Modalities. American journal of kidney diseases: the official journal of the National Kidney Foundation. 2018;71(3):344-51.Doi:10.1053/j.ajkd.2017.08.028.

16- Yeates K, Zhu N, Vonesh E, Trpeski L, Blake P, Fenton S. Hemodialysis And Peritoneal Dialysis Are Associated With Similar Outcomes For End-Stage Renal Disease Treatment In Canada. Nephrol Dial Transplant. 2012;27(9):3568-75.Doi:10.1093/ndt/gfr674.

17- Khan MI, Adeel FA, Luni FK, Khan AR, Malhotra D, Duggan J. Secondary Peritonitis in Peritoneal Dialysis: A Case Report and Review of Literature. Journal of Medical Cases. 2018;9(9):289-92.Doi:10.14740/jmc3116w.

18- Fayazi M, Javadzadeh H, Maghmoudi S. renal failure and dialysis emergencies. 2nd ed. Tehran: Haidary Publish; 2017 2017. 130 p.

19- Afsari P. Overview of dialysis 1395 2017 updated 06/08/2017. Available from: http://www.pharmed.co/News_Details.html?NewsId=1441.

20- Daugirdas JT, Blake PG, Ing TS. Handbook of Dialysis. 5th, editor. USA: Wolters Kluwer; 2015. 826 p.

21- Daugirdas JT, Depner TA. Kdoqi Clinical Practice Guideline for Hemodialysis Adequacy: 2015 Update. In: Kidney AJ, editor. USA2015. p. 884-930.

22- Kallenbach JZ. Review of Hemodialysis for Nurses and Dialysis Personnel. 9th Edition ed. USA: Mosby; 2016 30th June 2015. 432 p.

23- Ganu VJ, Boima V, Adjei DN, Yendork JS, Dey ID, Yorke E, et al. Depression and quality of life in patients on long term hemodialysis at a national hospital in Ghana: a cross-sectional study. Ghana Medical Journal. 2018;52(1):22-8.

24- Mousavi SSB, Hayati F, Ansari MJA, Valavi E, Cheraghian B, Shahbazian H, et al. Survival at 1, 3, and 5 years in diabetic and nondiabetic patients on hemodialysis. Iranian journal of kidney diseases. 2010;4(1):74-7.

25- Marc M, el Douaihy Y, Daoud M. Predictors of quality of life in patients with end-stage renal disease on hemodialysis. International Journal of Nephrology and Renovascular Disease. 2015;8:119-23.

26- Ashrafi Z, Ebrahimi H, Sarafha J. The relationship between hemodialysis adequacy and quality of life and spiritual wellbeing in hemodialysis patients. Journal of Clinical Nursing and Midwifery. 2014;3.

27- Momeni A, Alibeigi M, Dehghani Z, Kheiri S. Correlation evaluation of sexuality disorders with serum Prolactin, adequacy of dialysis, and some laboratory findings in Hemodialysis patients. Journal of Isfahan Medical School. 2014;32(278).

28- Malekmakan L, Sayadi M, Pakfetrat M. Assessment of pruritus status and its relation to dialysis adequacy and laboratory factors among hemodialysis patients. Journal of Jahrom University of Medical Sciences. 2013;11(1):55-49.

29- Haghighi AN, Broumand B, D'Amico M, Locatelli F, Ritz E. The epidemiology of end-stage renal disease in Iran in an international perspective. Nephrology Dialysis Transplantation. 2002;17(1):28-32.Doi:10.1093/ndt/17.1.28.

30- National Kidney F. KDOQI Clinical Practice Guideline for Hemodialysis Adequacy: 2015 update. American journal of kidney diseases: the official journal of the National Kidney Foundation. 2015;66(5):884-930.Doi:10.1053/j.ajkd.2015.07.015.

31- Keira St. G. Lynda W. D. J. Examining the effect of blood flow rate on hemodialysis urea clearance. 2017.

32- Ghorbani M Z, Sharifi S. Investigation of Hemodialysis Adequacy in Patients Undergoing Hemodialysis in the Shohada Hospital in Bushehr. 2014.

33- Johansen KL, Chertow GM, Jin C, Kutner NG. Significance of frailty among dialysis patients. J Am Soc Nephrol. 2007;18(11):2960-7.Doi:10.1681/ASN.2007020221.

34- Tentori F, Elder SJ, Thumma J, Pisoni RL, Bommer J, Fissell RB, et al. Physical exercise among participants in the Dialysis Outcomes and Practice Patterns

Study (DOPPS): correlates and associated outcomes. Nephrol Dial Transplant. 2010;25(9):3050-62.Doi:10.1093/ndt/gfq138.

35- Go AS, Chertow GM, Fan D. Chronic Kidney Disease and the Risks of Death, Cardiovascular Events, and Hospitalization. N Engl J Med. 2004;41(1):1296-305.Doi:10.1016/j.jvs.2004.10.020.

36- Vanholder R, Massy Z, Argiles A, Spasovski G, Verbeke F, Lameire N, et al. Chronic Kidney Disease As Cause Of Cardiovascular Morbidity And Mortality. Nephrol Dial Transplant. 2005;20(6):1048-56.Doi:10.1093/ndt/gfh813.

37- Chojak K, Smolenski O, Milkowski A, Pitrowski W. The Effects of 6-Month Physical Training Conducted During Hemodialysis In ESRD Patients. Med Rehabil. 2006;10(2):25-41.

38- Reyahi Z, Sfarjani F, Marandi SM, Bayat A, N. K. The Effects of Regular Exercise Program on Dialysis Efficacy, Muscle Atrophy and Physical Performance in Hemodialysis Patients. J Sharekord Univ Med Sci. 2012;14(5):63-73.

39- Tao X, Chow SK, Wong FK. A Nurse-Led Case Management Program On Home Exercise Training For Hemodialysis Patients: A Randomized Controlled Trial. Int J Nurs Stud. 2015;52(6):1029-41.Doi:10.1016/j.ijnurstu.2015.03.013.

40- Morishita S, Tsubaki A, Shirai N. Physical function was related to mortality in patients with chronic kidney disease and dialysis. Hemodial Int. 2017;21(4):483-9.Doi:10.1111/hdi.12564.

41- Dungey M, Young HML, Churchward DR, Burton JO, Smith AC, Bishop NC. Regular Exercise during Haemodialysis Promotes an Anti-Inflammatory Leucocyte Profile. Clin Kidney J. 2017;10(6):813-21.Doi:10.1093/ckj/sfx015.

42- Kong CH, Tattersall JE, Greenwood RN, Farrington K. The effect of exercise during haemodialysis on solute removal. Nephrology Dialysis Transplantation. 1999;14(12):2927-31.Doi:10.1093/ndt/14.12.2927.

43- Parsons TL, Toffelmire EB, King-VanVlack CE. Exercise training during hemodialysis improves dialysis efficacy and physical performance. Arch Phys Med Rehabil. 2006;87(5):680-7.Doi:10.1016/j.apmr.2005.12.044.

44- Chertow G, Levin N, Beck G. In-Center Hemodialysis Six Times per Week versus Three Times per Week. N Engl J Med. 2010;363(24):2287–300.

45- Suri RS, Larive B, Sherer S, Eggers P, Gassman J, James SH, et al. Risk of vascular access complications with frequent hemodialysis. J Am Soc Nephrol. 2013;24(3):498-505.Doi:10.1681/ASN.2012060595.

46- Kraus MA, Kansal S, Copland M, Komenda P, Weinhandl ED, Bakris GL, et al. Intensive Hemodialysis and Potential Risks With Increasing Treatment. American journal of kidney diseases: the official journal of the National Kidney Foundation. 2016;68(5S1):S51-S8.Doi:10.1053/j.ajkd.2016.05.020.

47- Daugirdas JT. Hemodialysis Treatment Time: As Important as it Seems? Seminars in dialysis. 2017;30(2):93-8.Doi:10.1111/sdi.12575.

48- Anees M, Hussain Y, Ibrahim M, Ilahi I, Ahmad S, Asif K, et al. Outcome of Chronic Kidney Disease Patients on the Basis of Referral to Nephrologist. JCPSP. 2018;28(4):304-7.

49- Brasileiro T, Prado A, Assis B, Nogueira D, Lima R, Chaves E. Effects Of Prayer On The Vital Signs Of Patients With Chronic Kidney Disease. Rev Esc Enferm USP. 2017;51(1):1-9.Doi:http://dx.doi.org/10.1590/s1980-220x2016024603236

50- Headley S, Germain M, Wood R, Joubert J, Milch C, Evans E, et al. Blood Pressure Response To Acute And Chronic Exercise In Chronic Kidney Disease. Nephrology. 2015;22(1):72-8.Doi:10.1111/nep.12730.

51- Anderson J, Boivin J, Hatchett L. Effect Of Exercise Training on Interdialytic Ambulatory And Treatment-Related Blood Pressure In Hemodialysis Patients. Ren Fail. 2004;26(5):539-44.Doi:10.1081/JDI-200031735.

52- De Moura Reboredo M, Henrique DM, De Souza Faria R, Chaoubah A, Bastos MG, RB. DP. Exercise Training During Hemodialysis Reduces Blood Pressure And Increases Physical Functioning And Quality Of Life. Artif Organs. 2010;34(7):586-93.Doi:10.1111/j.1525-1594.2009.00929.x.

53- Susumu O. Blood Volume Changes Induced By Low-Intensity Intradialytic Exercise in Long-Term Hemodialysis Patients. ASAIO J. 2016;62(2):190-6.Doi:10.1097/MAT.0000000000000320.

54- Banerjee A, Kong C, Farrington K. The Haemodynamic Response To Submaximal Exercise During Isovolaemic Haemodialysis. Nephrol Dial Transplant. 2004;19(6):1528–32.Doi:10.1093/ndt/gfh518.

55- Murtagh F, Addington-Hall J, Higginson J. The prevalence of symptoms in end-stage renal disease: a systematic review. J Advances in chronic kidney disease. 2007;14(1):82-99.

56- Bossola M, Vulpio C, Tazza L. Fatigue in chronic dialysis patients. Seminars in dialysis. 2011;24(5):550-5.Doi:10.1111/j.1525-139X.2011.00956.x.

57- Caplin B, Kumar S, Davenport A. Patients' Perspective of Haemodialysis-Associated Symptoms. Nephrol Dial Transplant. 2011;26(8):2656-63.

58- Horigan AE. Fatigue in Hemodialysis Patients: A Review of Current Knowledge. J Pain Symptom Manage. 2012;44(5):715-24.

59- Biniaz V, Tayybi A, Nemati E, Sadeghi Shermeh M, Ebadi A. Different aspects of fatigue experienced by patients receiving maintenance dialysis in hemodialysis units. Nephrourol Mon. 2013;5(4):897-900.Doi:10.5812/numonthly.11667.

60- Bossola M, Luciani G, Tazza L. Fatigue and its correlates in chronic hemodialysis patients. Blood Purif. 2009;28(3):245-52.Doi:10.1159/000231985.

61- Bossola M, Di Stasio E, Antocicco M, Panico L, Pepe G, Tazza L. Fatigue Is Associated with Increased Risk of Mortality in Patients on Chronic Hemodialysis. Nephron. 2015;130(2):113-8.Doi:10.1159/000430827.

62- Picariello F, Moss-Morris R, Macdougall IC, Chilcot AJ. The role of psychological factors in fatigue among end-stage kidney disease patients: a critical review. Clin Kidney J. 2017;10(1):79-88.Doi:10.1093/ckj/sfw113.

63- Motedayen Z, Nehrir B, Tayebi A, Ebadi A, Einollahi B. The effect of the physical and mental exercises during hemodialysis on fatigue: a controlled clinical trial. Nephrourol Mon. 2014;6(4):e14686. Doi:10.5812/numonthly.14686.

64- Goodman D, Ballou M. <Perceived barriers and motivators to Exercise in Hemodialysis Patients.pdf>. 2004.

65- Liberatore MJ. The Effects of Exercise Education Intervention on the Exercise Behaviour, Depression, and Fatigue Status of Chronic Kidney Disease Patients. Int J Health Care Qual Assur. 2013;26(7):601-26.Doi:10.1108/IJHCQA-09-2011-0054.

66- Dobsak P, Homolka P, Svojanovsky J, Reichertova A, Soucek M, Novakova M, et al. Intra-dialytic electrostimulation of leg extensors may improve exercise tolerance and quality of life in hemodialyzed patients. Artif Organs. 2012;36(1):71-8.Doi:10.1111/j.1525-1594.2011.01302.x.

67- Kalantar-Zadeh K, Unruh M. Health related quality of life in patients with chronic kidney disease. Int Urol Nephrol. 2005;37(2):367-78.Doi:10.1007/s11255-004-0012-4.

68- Mccann K, Boore JRP. <Fatigue in persons with renal failure who require maintenance HD.pdf>. 2000.

69- Picariello F, Moss-Morris R, Macdougall IC, Chilcot J. Measuring Fatigue in Haemodialysis Patients: The Factor Structure of the Chalder Fatigue

Questionnaire (CFQ). J Psychosom Res. 2016;84:81-3.Doi:10.1016/j.jpsychores.2016.03.124.

70- Zakerimoghadam M, Kazemnezhad A, Tavasoli K. The Effect Of Breathing Exercises On Fatigue In Patients With Chronic Obstructive Pulmonary Disease. J Nurs Midwifery. 2005;12(3):17-25.

71- Kirkman DL, Roberts LD, Kelm M, Wagner J, Jibani MM, Macdonald JH. Interaction between Intradialytic Exercise and Hemodialysis Adequacy. Am J Nephrol. 2013;38(6):475-82.Doi:10.1159/000356340.

72- Van Vilsteren MC, De Greef MH, Huisman RM. The Effects Of A Low-To-Moderate Intensity Pre-Conditioning Exercise Programme Linked With Exercise Counselling For Sedentary Haemodialysis Patients In The Netherlands: Results Of A Randomized Clinical Trial. Nephrol Dial Transplant. 2005;20(1):141-6.Doi:10.1093/ndt/gfh560.

73- Z. R, F. E, SM M. The effects of regular exercise program on dialysis efficacy, muscle atrophy and physical performance in hemodialysis patients. 2012.

74- Shafipour;V, Spahbodi;F, Madani. Effect of Intradialytic and Home-Based Walking Exercises on Physical Function and Dialysis Adequacy in Hemodialysis Patients. 2017.

75- Vaithilingam;I, Polkinghorne;K.R., Atkins;R.C. Time and Exercise Improve Phosphate Removal in Hemodialysis Patients. 2004Doi:10.1053/j.ajkd.2003.09.016.

76- Mcdowell CP, Dishman RK, Hallgren M, Macdonncha C, Herring M. Associations Between Physical Activity and Depression: Results from The Irish Longitudinal Study on Ageing. Medicine & Science in Sports & Exercise. 2018;50(5S):251.

77- De Oliveira GD, Oancea SC, Nucci LB, Vogeltanz-Holm N. The association between physical activity and depression among individuals residing in Brazil. Social psychiatry and psychiatric epidemiology. 2018;53(4):373-83.

78- Brunner LS. Brunner & Suddarth's textbook of medical-surgical nursing: Lippincott Williams & Wilkins; 2017.

79- Eshghizadeh M, Basiri Moghadam K, Baloochi Beydokhti T, Safarpoor Gharib Z, Mokhtari S. Dialysis adequacy in diabetic and non-diabetic patients admitted to a hospital in Gonabad, Iran in 2012.. Med Surg Nurs J. 2014;3(2):77-83.

80- Hafen BQ, Karren KJ, Mistovich JJ. Prehospital emergency care. 11 ed: Brady, Prentice Hall; 1996.

81- Goransson K, Von Rosen R. Interrater Agreement: A Comparison between Two Emergency Department Triage Scales. Eur J Emerg Med. 2010;18(2):68-72.Doi:10.1097/MEJ.0b013e32833ce4eb.

82- Aghababaeian H, Taheri N, AraghiAhvazi L, Sadeghi Moghaddam A. Fundamental of triage. Tehran: Jameenegar pub; 2012.

83- Organization WH. Guidelines on physical activity, sedentary behaviour and sleep for children under 5 years of age Printed in Switzerland: World Health Organization 2019 3rd: [Guidelines on physical activity, sedentary behaviour and sleep for children under 5 years of age]. Available from: http://www.who.int/iris/handle/10665/311664.

84- Ajzen I. The theory of planned behavior. Organizational Behavior and Human Decision Processes. 1991;50(2):179-211.Doi:https://doi.org/10.1016/0749-5978(91)90020-T.

85- Ajzen I. Consumer attitudes and behavior: the theory of planned behavior applied to food consumption decisions. Italian Review of Agricultural Economics. 2015;70(2):121-38.

86- Abraham C, Michie S. A taxonomy of behavior change techniques used in interventions. Health Psychol. 2008;27(3):379-87. [eng] Doi:10.1037/0278-6133.27.3.379.

87- Pakpour AH, Zeidi IM, Chatzisarantis N, Molsted S, Harrison AP, Plotnikoff RC. Effects of action planning and coping planning within the theory of planned behaviour: A physical activity study of patients undergoing haemodialysis. Psychology of Sport and Exercise. 2011;12(6):609-14.Doi:https://doi.org/10.1016/j.psychsport.2011.06.008.

88- Steinmetz H, Knappstein M, Ajzen I, Schmidt P, Kabst R. How Effective are Behavior Change Interventions Based on the Theory of Planned Behavior? Zeitschrift für Psychologie. 2016;224(3):216-33.Doi:10.1027/2151-2604/a000255.

89- Dormanesh B, Daryabeigi R, Hadi S, Sepahvand V, Sarkhosh Tonekaboni A. Application of the Theory of Planned Behavior in Dietary and Physical Activity Behaviors among Hemodialysis Patients Visiting Army Hospitals. umsha-psj. 2018;16(2):19-27.Doi:10.21859/psj.16.2.19.

90- Hinkle J, Cheever K. Internal nursing and kidney and urinary tract surgery. Tehran: Jame_Negar; 2018.

91- Zakeri M, Ali Asgharpour M. Special nursing care in CCU, ICU and dialysis departments: Andishe Rafie; 2013.

92- Asgari MR, Soleimani M. Intensive Nursing Care in ICU, CCU and Dialysis Wards. Tehran: Boshra Publisher; 2017.

93- Khalili M. Kidney physiology: Dibaj Publisher; 2010.

94- Goldman L, Ausiello DA. Cecil medicine. 26 ed: Saunders Elsevier Philadelphia; 2019.

95- Hinkle JL, Cheever KH. Study Guide for Brunner & Suddarth's Textbook of Medical-surgical Nursing: Lippincott Williams & Wilkins; 2013.

96- Kallenbach JZ. Review of Hemodialysis for Nurses and Dialysis Personnel-E-Book: Elsevier Health Sciences; 2020.

97- Hinkle J. Adult and Elderly Care for Bruner and Sodarth 2018: Kidney and Urinary Tract Diseases. Tehran: Heidari; 2018.

98- Glover C, Banks P, Carson A, Martin CR, Duffy T. Understanding and assessing the impact of end-stage renal disease on quality of life. The Patient: Patient-Centered Outcomes Research. 2011;4(1):19-30.

99- Molsted S, Prescott L, Heaf J, Eidemak I. Assessment and clinical aspects of health-related quality of life in dialysis patients and patients with chronic kidney disease. Nephron Clinical Practice. 2007;106(1):c24-c33.

100- Bare BG, Hinkle JL, Cheever KH, Smeltzer SCC. Instructor's Resource DVD for" Brunner & Suddarth's Textbook of Medical-surgical Nursing": Lippincott Williams & Wilkins.; 2010.

101- Webster A, Nagler EV, Morton R, Masson P. Seminar Chronic kidney disease. Lancet [Internet]; 2017. Contract No.: 10075.

102- Hayat S. pretoneal dialysis. 2012:13-20.

103- Daugirdas JT, Blake PG, Ing TS. Handbook of dialysis: Lippincott Williams & Wilkins; 2017.

104- Asgari M, Soleimani M. Comprehensive book of special nursing care in CCU, ICU and Dialysis departments. Tehran: Human propaganda; 2011.

105- Nemati E. Dialysis in Iran. Tehran: Iran University of Medical Sciences; 2008.

106- Lima EQ, Silva RG, Donadi EL, Fernandes AB, Zanon JR, Pinto KR, et al. Prevention of intradialytic hypotension in patients with acute kidney injury submitted to sustained low-efficiency dialysis. Ren Fail. 2012;34(10):1238-43.Doi:10.3109/0886022x.2012.723581.

107- Lambers Heerspink HJ, Navis G, Ritz E. Salt intake in kidney disease--a missed therapeutic opportunity? Nephrol Dial Transplant. 2012;27(9):3435-42.Doi:10.1093/ndt/gfs354.

108- Weiner DE, Brunelli SM, Hunt A, Schiller B, Glassock R, Maddux FW, et al. Improving clinical outcomes among hemodialysis patients: a proposal for a "volume first" approach from the chief medical officers of US dialysis providers. American journal of kidney diseases: the official journal of the National Kidney Foundation. 2014;64(5):685-95. Doi:10.1053/j.ajkd.2014.07.003.

109- Zoccali C, Moissl U, Chazot C, Mallamaci F, Tripepi G, Arkossy O, et al. Chronic Fluid Overload and Mortality in ESRD. J Am Soc Nephrol. 2017;28(8):2491-7.Doi:10.1681/asn.2016121341.

110- Diroll D. Oxygen as an adjunct to treat intradialytic hypotension during hemodialysis. Nephrol Nurs J. 2014;41(4):420-3.

111- Authers Ago. Nursing and Kidney Failure. Tehran: Tandis; 2008.

112- Dunne N, Campbell M, Fitzpatrick M, Callery P. Comparison of Kt/V and urea reduction ratio in measuring dialysis adequacy in paediatric haemodialysis in England. J Ren Care. 2014;40(2):117-24.Doi:10.1111/jorc.12059.

113- Hemayati R, Lesanpezeshki M, Seifi S. Association of dialysis adequacy with nutritional and inflammatory status in patients with chronic kidney failure. Saudi J Kidney Dis Transpl. 2015;26(6):1154-60.Doi:10.4103/1319-2442.168593.

114- Mohseni R, Emami Zeydi A, Ilali E, Adib-Hajbaghery M, Makhlough A. The effect of intradialytic aerobic exercise on dialysis efficacy in hemodialysis patients: a randomized controlled trial. Oman Med J. 2013;28(5):345-9.Doi:10.5001/omj.2013.99.

115- Jindal K, Chan CT, Deziel C, Hirsch D, Soroka SD, Tonelli M, et al. Hemodialysis clinical practice guidelines for the Canadian Society of Nephrology. Journal of the American Society of Nephrology: JASN. 2006;17(3 Suppl 1):S1-27.

116- Bassam Pour S, Asadi Neghabi A, Zolfaghari M. Special Nursing Care, ICU, CCU, Dialysis. Tehran Publication Salemi. 2011:366-297.

117- El-Sheikh M, El-Ghazaly G. Assessment of hemodialysis adequacy in patients with chronic kidney disease in the hemodialysis unit at Tanta University Hospital in Egypt. Indian J Nephrol. 2016;26(6):398-404.Doi:10.4103/0971-4065.168141.

118- Ebrahimi H, Khosravi A, Bolbolhaghighi N. Relationship between the Dose of Erythropoietin and the Dialysis Adequecy. Knowledge and Health in Basic Medical Sciences. 2008;3(2):7-12.Doi:10.22100/jkh.v3i2.208.

119- Wilund KR, Jeong JH, Greenwood SA. Addressing myths about exercise in hemodialysis patients. Seminars in dialysis. 2019;32(4):297-302.Doi:https://doi.org/10.1111/sdi.12815.

120- WHO. Guidelines on physical activity, sedentary behaviour and sleep for children under 5 years of age[Internet]. Switzerland: World Health Organization; 2019 [Available from: http://www.who.int/iris/handle/10665/311664.

121- WHO. Physical activity[internet] Geneva: World Health Organization; 2018 [Available from: https://www.who.int/news-room/fact-sheets/detail/physical-activity.

122- Warburton DER, Bredin SSD. Health benefits of physical activity: a systematic review of current systematic reviews. Current opinion in cardiology. 2017;32(5):541-56.Doi:10.1097/hco.0000000000000437.

123- Odden MC, Whooley MA, Shlipak MG. Association of chronic kidney disease and anemia with physical capacity: the heart and soul study. J Am Soc Nephrol. 2004;15(11):2908-15.Doi:10.1097/01.Asn.0000143743.78092.E3.

124- Kirkman DL, Edwards DG, Lennon-Edwards S. Exercise as an Adjunct Therapy In Chronic Kidney Disease. Renal Nutr Forum. 2014;33(4):1-8.

125- Pagels AA, Söderkvist BK, Medin C, Hylander B, Heiwe S. Health-related quality of life in different stages of chronic kidney disease and at initiation of dialysis treatment. Health Qual Life Outcomes. 2012;10:71.Doi:10.1186/1477-7525-10-71.

126- Painter P, Roshanravan B. The association of physical activity and physical function with clinical outcomes in adults with chronic kidney disease. Curr Opin Nephrol Hypertens. 2013;22(6):615-23. Doi:10.1097/MNH.0b013e328365b43a.

127- Jhamb M, McNulty ML, Ingalsbe G, Childers JW, Schell J, Conroy MB, et al. Knowledge, barriers and facilitators of exercise in dialysis patients: a qualitative study of patients, staff and nephrologists. BMC Nephrol. 2016;17(1):192.Doi:10.1186/s12882-016-0399-z.

128- Kontos P, Grigorovich A, Colobong R, Miller KL, Nesrallah GE, Binns MA, et al. Fit for Dialysis: a qualitative exploration of the impact of a research-based film for the promotion of exercise in hemodialysis. BMC Nephrol. 2018;19(1):195.Doi:10.1186/s12882-018-0984-4.

129- Zheng J, You LM, Lou TQ, Chen NC, Lai DY, Liang YY, et al. Development and psychometric evaluation of the Dialysis patient-perceived Exercise Benefits and Barriers Scale. Int J Nurs Stud. 2010;47(2):166-80. Doi:10.1016/j.ijnurstu.2009.05.023.

130- Rosa CSC, Bueno DR, Souza GD, Gobbo LA, Freitas IF, Sakkas GK, et al. Factors associated with leisure-time physical activity among patients undergoing hemodialysis. BMC nephrology. 2015;16(1):1-7.

131- Hoshino J. Renal Rehabilitation: Exercise Intervention and Nutritional Support in Dialysis Patients. Nutrients. 2021;13(5)Doi:10.3390/nu13051444.

132- Ghafourifard M, Mehrizade B, Hassankhani H, Heidari M. Hemodialysis patients perceived exercise benefits and barriers: the association with health-related quality of life. BMC Nephrology. 2021;22(1):94.Doi:10.1186/s12882-021-02292-3.

133- Sajjadi A, Farmahini Farahani B, Esmailpoor Zanjani S, Dormanesh B, Zare M. Effective factors on fatigue in patients with chronic renal failure undergoing hemodialysis. J Crit Care Nurs. 2010;3(1):13-4.

134- Krupp LB. Fatigue in multiple sclerosis: a guide to diagnosis and management: Demos Medical Publishing; 2004.

135- Ennis M. Living with fatigue: Fatigue management for people with MS. Letchworth Garden: Multiple Sclerosis Trust; 2015.

136- Chehrehgosha M, Dastourpour M, Sanagu A, Mohamadi A. Cancer-related fatigue and its relationship with demographic and clinical characteristics. Jorjani Biomedicine Journal. 2013;1(2):24-31.

137- Marcora SM, Staiano W, Manning V. Mental fatigue impairs physical performance in humans. Journal of applied physiology. 2009;106(3):857-64.

138- Watanabe N, Stewart R, Jenkins R, Bhugra DK, Furukawa TA. The epidemiology of chronic fatigue, physical illness, and symptoms of common mental disorders: a cross-sectional survey from the second British National Survey of Psychiatric Morbidity. Journal of psychosomatic research. 2008;64(4):357-62.

139- Farhad L, Brazparandjani S, Latifi SM, Chahkhoei M, Khalili A, Paymard A, et al. The Effect of Collaborative Care model on the Fatigue in Patients Undergoing Maintenance Hemodialysis: A Randomized Clinical Trial. Qom Univ Med Sci J. 2016;10(8):71-9. [persian].

140- Rejeh N, Hearavi KM, Bahrami T, Raeesi R, D. T. The assessment of factors affecting fatigue in older people with hemodialysis. Iranian Journal of Nursing Research. 2015;10(3):108-17. [Persian].

141- Chilcot J, Moss-Morris R, Artom M, Harden L, Picariello F, Hughes H, et al. Correction to: Psychosocial and Clinical Correlates of Fatigue in Haemodialysis Patients: the Importance of Patients' Illness Cognitions and Behaviours.

International journal of behavioral medicine. 2017;24(6):958.Doi:10.1007/s12529-017-9692-x.

142- Sakkas G, Karatzaferi C. Hemodialysis Fatigue: Just “Simple” Fatigue or a Syndrome on Its Own Right? Frontiers in Physiology. 2012;3(306). [English] Doi:10.3389/fphys.2012.00306.

143- Tavakoli M, M R, A Z, A Dm. Evaluation of Fatigue in Hemodialysis Patients in AJA Selected Hospitals. Military Caring Sciences. 2016;2(4):197-205.Doi:10.18869/acadpub.mcs.2.4.197.

144- Madadkardehkordi S, M B. Fatigue and factors affecting it in patients with chronic renal failure undergoing hemodialysis in two hemodialysis centers in Gonabad and Yazd. Nursing of the VulnerableJournal. 2017;4(10):35-43. [persian].

145- Jacobson J, Ju A, Baumgart A, Unruh M, O’Donoghue D, Obrador G, et al. Patient perspectives on the meaning and impact of fatigue in hemodialysis: a systematic review and thematic analysis of qualitative studies. American Journal of Kidney Diseases. 2019;74(2):179-92. Doi:10.1053/j.ajkd.2019.01.034.

146- Salehi F, Dehghan M, Mangolian Shahrbabaki P, Ebadzadeh MR. Effectiveness of exercise on fatigue in hemodialysis patients: a randomized controlled trial. BMC Sports Science, Medicine and Rehabilitation. 2020;12(1):19.Doi:10.1186/s13102-020-00165-0.

147- Biabani F, Moghrrab M, Nasirizadeh M. The effect of muscle relaxation on nausea and dialysis adequacy in hemodialysis patient. Journal of Sabzevar University of Medical Sciences. 2019;26(3):303-9. [persian].

148- Shayanimomtaz M, Arsalani N, Mohammadishahbolaghi F, Biglaryan A. The effect of rehabilitation plan on fatigue in hemodialysis patients. IJRN. 2019;5(3):23-30. [persian].

149- Basiri Moghaddam M., Mohamadpour A., Mottaghi M.R., Jahani Maghani F. Impact of Isometric and Isotonic Movements during Hemodialysis on Dialysis Adequacy. Quarterly of Horizon of Medical Sciences. 2016;22(1):35-41. [Persian] Doi:10.18869/acadpub.hms.22.1.35.

150- Rochmawati E, Utomo EK, Makiyah SNN. Improving dialysis adequacy and quality of life in patients undergoing hemodialysis with twice a week range of motion exercise. Therapeutic Apheresis and Dialysis. 2021;n/a(n/a)Doi:https://doi.org/10.1111/1744-9987.13701.

151- Hargrove N, El Tobgy N, Zhou O, Pinder M, Plant B, Askin N, et al. Effect of Aerobic Exercise on Dialysis-Related Symptoms in Individuals Undergoing

Maintenance Hemodialysis. A Systematic Review and Meta-Analysis of Clinical Trials. 2021;16(4):560-74.Doi:10.2215/cjn.15080920.

152- Sheshadri A, Kittiskulnam P, Johansen KL. Higher Physical Activity Is Associated With Less Fatigue and Insomnia Among Patients on Hemodialysis. Kidney Int Rep. 2019;4(2):285-92.Doi:10.1016/j.ekir.2018.10.014.

153- Dziubek W, Bulińska K, Rogowski Ł, Gołębiowski T, Kusztal M, Grochola M, et al. The effects of aquatic exercises on physical fitness and muscle function in dialysis patients. BioMed research international. 2015;2015(Special Issue)Doi:10.1155/2015/912980.

154- Tentori F, Zhang J, Li Y, Karaboyas A, Kerr P, Saran R, et al. Longer dialysis session length is associated with better intermediate outcomes and survival among patients on in-center three times per week hemodialysis: results from the Dialysis Outcomes and Practice Patterns Study (DOPPS). Nephrology Dialysis Transplantation. 2012;27(11):4180-8.Doi:10.1093/ndt/gfs021.

155- Krupp LB, LaRocca N, Muir-Nash J, Steinberg A. The Fatigue Severity Scale: Application to Patients with Multiple Sclerosis and Systemic Lupus Erythematosus. J Archives of neurology. 1989;46(10):1121-3.

156- Zakeri Moghaddam M, Shaban M, Kazemnezhad A, Tavassoli K. Effect of Exercise Utilizing the Rate of Respiratory On Fatigue In Patient With Chronic Obstructive Pulmonary Disease. J Hayat. 2006;3(30):17-25.

157- Rasouli N, Ahmadi F, Nabavi SM, Haji-Zadeh E. Effect of Energy Saving Technique on the Rate of Multiple Sclerotic Fatigue. J Journal of Rehabilitation. 2006;7(1):43-8.

158- nezamlo A, Oshvand K, Ghlyaf M, Homayounfar S. The Effects of Physical Training during Dialysis on the Prevention of Anemia in Hemodialysis Patients J Scientific Journal of Hamadan Nursing & Midwifery Faculty. 2015;23(4):65-76.

159- Walter R, Gordon N, Pescatello L. ACSM's guidelines for exercise testing and prescription. Lippincott Williams & Wilkins, Philadelphia; 2010.

160- Kulikowski E, Halliday C, Johansson J, Sweeney M, Lebioda K, Wong N, et al. Apabetalone mediated epigenetic modulation is associated with favorable kidney function and alkaline phosphatase profile in patients with chronic kidney disease. Kidney Blood Press Res. 2018;43(2):449-57. Doi:10.1159/000488257.

161- Saglimbene V, Natale P, Palmer S, Scardapane M, Craig JC, Ruospo M, et al. The Prevalence And Correlates Of Low Sexual Functioning In Women On Hemodialysis: A Multinational, Cross-Sectional Study. PloS one. 2017;12(6)Doi:10.1371/journal.pone.0179511.

162- Sokal P, Jastrzębski Z, Jaskulska E, Sokal K, Jastrzębska M, Radzimiński L, et al. Differences in Blood Urea and Creatinine Concentrations in Earthed and Unearthed Subjects during Cycling Exercise and Recovery. Evid Based Complement Alternat Med. 2013;2013:382643.Doi:10.1155/2013/382643.

163- Zhao J, Qi Q, Xu S, Shi D. Combined aerobic resistance exercise improves dialysis adequacy and quality of life in patients on maintenance hemodialysis Clin Nephrol. 2020;93(6):275-82.Doi:10.5414/cn110033.

164- Soltani P, Saeedi N, Mashaykhi N, Rostami A, Tajfar M. Evaluating the effect of intradialytic exercise on blood pressure, inflammatory markers and dialysis adequacy. J Prev Epidemiol. 2020;5(2):e21-e. Doi:10.34172/jpe.2020.21.

165- Saud A, Luiz RS, Leite APO, Muller CR, Visona I, Reinecke N, et al. Resistance exercise training ameliorates chronic kidney disease outcomes in a 5/6 nephrectomy model. Life Sciences. 2021;275:119362. Doi:https://doi.org/10.1016/j.lfs.2021.119362.

166- Foran SE, Lewandrowski KB, Kratz A. Effects of exercise on laboratory test results. Laboratory medicine. 2003;34(10):736-42.

167- Pujiastuti TT, Aima H, Lokonathan S, Chiew L. Effectiveness of intradialytic exercise to reduce interdialytic weight gain in patients on hemodialysis at Private Hospital Yogyakarta. Enferm Clin. 2020;30 Suppl 5:17-20.Doi:10.1016/j.enfcli.2020.01.007.

168- Rowley S, Montgomery E, Avery L, O'Brien N, Flynn D, editors. Dietary and physical activity/exercise behavioural interventions for weight loss in adults with chronic kidney disease: a systematic review2020 2020.

169- Jafari F, Hashemi N, Reisi M. The effect of diet training on variations in blood pressure, weight, and some biochemical factors in hemodialysis patients: a clinical trial. J Clin Nurs Midwifery. 2015;3(4):13-9.

170- Hatef M, Esmaeili R, Mousavinasab N, Madani Z, Spahbodi F, Shafipour V. Effect of Intradialytic and Home-Based Walking Exercises on Physical Function and Dialysis Adequacy in Hemodialysis Patients. Journal of Mazandaran University of Medical Sciences. 2017;27(154):83-93.

171- Henrique DM, Reboredo Mde M, Chaoubah A, Paula RB. [Aerobic exercise improves physical capacity in patients under chronic hemodialysis]. Arq Bras Cardiol. 2010;94(6):823-8. [por] Doi:10.1590/s0066-782x2010005000043.

172- Orcy R, Antunes MF, Schiller T, Seus T, Böhlke M. Aerobic exercise increases phosphate removal during hemodialysis: a controlled trial. Hemodial Int. 2014;18(2):450-8.Doi:10.1111/hdi.12123.

173- Salhab N, Alrukhaimi M, Kooman J, Fiaccadori E, Aljubori H, Rizk R, et al. Effect of Intradialytic Exercise on Hyperphosphatemia and Malnutrition. Nutrients. 2019;11(10)Doi:10.3390/nu11102464.

174- Deligiannis A, D'Alessandro C, Cupisti A. Exercise training in dialysis patients: impact on cardiovascular and skeletal muscle health. Clinical Kidney Journal. 2021;14(Supplement_2):ii25-ii33.Doi:10.1093/ckj/sfaa273.

175- Morais MJD, de Abreu LC, Santana de Oliveira F, Pinheiro Bezerra IM, Raimundo RD, Paulo Martins Silva R, et al. Is aerobic exercise training during hemodialysis a reliable intervention for autonomic dysfunction in individuals with chronic kidney disease? A prospective longitudinal clinical trial. J Multidiscip Healthc. 2019;12:711-8.Doi:10.2147/jmdh.S202889.

176- Sheshadri A, Kittiskulnam P, Johansen KL. Higher Physical Activity Is Associated With Less Fatigue and Insomnia Among Patients on Hemodialysis. Kidney Int Rep. 2019;4(2):285-92. Doi:https://doi.org/10.1016/j.ekir.2018.10.014.

177- Moghadam S, Shahdadi H, Hodki R, Shamsizadeh M, Shrafi E. The Effect of Hand Reflexology on Fatigue in Patients Undergoing Hemodialysis. Journal of Zabol University of Medical Sciences and Health Services. 2016;8(1):1-10.

178- Rietberg MB, van Wegen EE, Eyssen IC, Kwakkel G. Effects of multidisciplinary rehabilitation on chronic fatigue in multiple sclerosis: a randomized controlled trial. PLoS One. 2014;9(9):e107710. Doi:10.1371/journal.pone.0107710.

پیوست ها

پیوست ۱. فرم اطلاعات نمونه

به نام آن‌که اسمش دوا و ذکرش شفاست

فرم اطلاعات نمونه **نمونه شماره . . . . .**

قبل از مداخله	وزن قبل از دیالیز	وزن بعد از دیالیز	BP	MAP	PR
	RR	Tem	SaO2%	BUN	

جلسه اول		وزن قبل از دیالی	وزن بعد از دیالیز	قبلBUN	بعدBUN	KT/V	
		BP	MAP	SaO2%	Tem	RR	PR
	قبل						
	بعد						

جلسه دوم		وزن قبل از دیالیز	وزن بعد از دیالیز	قبلBUN	بعدBUN	KT/V	
		BP	MAP	SaO2%	Tem	RR	PR
	قبل						
	بعد						

جلسه سوم		وزن قبل از دیالیز	وزن بعد از دیالیز	قبلBUN	بعدBUN	KT/V	
		BP	MAP	SaO2%	Tem	RR	PR
	قبل						
	بعد						

جلسه چهارم							
		وزن قبل	وزن بعد از دیالیز	BUNقبل	BUNبعد	KT/V	
		BP	MAP	SaO2%	Tem	RR	PR
	قبل						
	بعد						

جلسه پنجم							
		وزن قبل از دیالیز	وزن بعد از دیالیز	BUNقبل	BUNبعد	KT/V	
		BP	MAP	SaO2%	Tem	RR	PR
	قبل						
	بعد						

جلسه ششم							
		وزن قبل از دیالیز	وزن بعد از دیالیز	BUN قبل	BUNبعد	KT/V	
		BP	MAP	SaO2%	Tem	RR	PR
	قبل						
	بعد						

جلسه هفتم							
		وزن قبل از دیالیز	وزن بعد از دیالیز	BUN قبل	BUNبعد	KT/V	
		BP	MAP	SaO2%	Tem	RR	PR
	قبل						
	بعد						

جلسه هشتم		وزن قبل از دیالیز	وزن بعد از دیالیز	BUN قبل	بعدBUN	KT/V	
		BP	MAP	SaO2%	Tem	RR	PR
	قبل						
	بعد						

جلسه نهم		وزن قبل از د یالیز	وزن بعد از دیالیز	BUN قبل	بعدBUN	KT/V	
		BP	MAP	SaO2%	Tem	RR	PR
	قبل						
	بعد						

جلسه دهم		وزن قبل از دیالیز	وزن بعد از دیالیز	BUN قبل	بعدBUN	KT/V	
		BP	MAP	SaO2%	Tem	RR	PR
	قبل						
	بعد						

KT/V	BUN بعد	قبل BUN	وزن بعد از دیالیز	وزن قبل از دیالیز			جلسه یازدهم
PR	RR	Tem	SaO2%	MAP	BP		
						قبل	
						بعد	

KT/V	BUN بعد	قبل BUN	وزن بعد از دیالیز	وزن قبل از دیالیز			جلسه دوازدهم
PR	RR	Tem	SaO2%	MAP	BP		
						قبل	
						بعد	

پیوست ۲. پرسشنامه شدت خستگی

بسمه تعالی

پاسخگوی محترم، با سلام

پرسشنامه ای که در اختیار دارید به منظور یک کار تحقیقاتی تنظیم گردیده است. بدیهی است کلیه پاسخ‌ها و اظهارات شما محرمانه تلقی شده و صرفاً در قالب ارقام آماری مورد استفاده قرار می گیرد و نیازی به ذکر نام نمی باشد. بدون شک پاسخگویی صحیح شما موجب درستی نتایج حاصل از پرسشنامه حاضر خواهد شد. نهایتاً از همکاری صمیمانه حضرتعالی کمال تشکر را می نمایم.

مشخصات پاسخگو:

۱-وضع جنسیت: مرد☐ زن☐

۲-وضعیت تأهل: مجرد☐ متأهل ☐

۳-تحصیلات: زیردیپلم☐ دیپلم☐ فوق‌دیپلم☐ لیسانس☐ فوق‌لیسانس☐ دکتری☐

۴- سن(سال): ....

پرسشنامه شدت خستگی FSS (Fatigue severity scale)

ردیف	عبارات	۱	۲	۳	۴	۵	۶	۷
۱	وقتی که من خسته هستم انگیزه من کمتر است.							
۲	ورزش در من خستگی می آورد.							
۳	من به آسانی خسته می شوم.							
۴	خستگی در عملکرد فیزیکی من اختلال ایجاد می کند.							
۵	خستگی باعث بروز مشکلات مکرر برای من می شود.							
۶	خستگی من مانع عملکرد مداوم بدنی می شود.							
۷	خستگی مانع انجام وظایف و مسئولیت های خاص من است.							
۸	خستگی جزء ۳ تا از علایم ناتوان کننده من است.							
۹	خستگی در کار، خانواده و زندگی اجتماعی من اختلال ایجاد می کند.							

پیوست ۳. مصوبه اخلاق

دانشگاه علوم پزشکی بوشهر

مصوبه اخلاق در پژوهش

شناسه:	IR.BPUMS.REC.1398.130	تاریخ تصویب: ۱۳۹۸/۱۰/۱
محل بررسی:	دانشگاه علوم پزشکی بوشهر	
تصمیم صادره:	مصوب	
مصوبه کمیته اخلاق:	پروپوزال طرح، در جلسه‌ی دانشگاه علوم پزشکی بوشهر مورد بررسی قرارگرفت و بر اساس مدارک ارسالی مورد تصویب کمیته‌ی اخلاق واقع گردید.<br>توجه:<br>۱. علی‌رغم تصویب مطالعه در کمیته‌ی اخلاق در پژوهش، کلیه‌ی مسؤولیت‌های حقوقی و حرفه‌ای اجرای طرح برعهده‌ی شما و همکارانتان باقی خواهد ماند.<br>۲. کلیه‌ی مستندات مطروحه در این مصوبه، بر اساس مدارک دریافت شده در تاریخ ۱۳۹۸/۱۰/۱ می‌باشد و ضروری است هرگونه تغییرات و اصلاحات اعمال شده در این مستندات، توسط متقاضی محترم طرح فورا به کمیته‌ی اخلاق اطلاع داده شود.	
عنوان پایان‌نامه (فارسی):	مقایسه اثربخشی فعالیت فیزیکی حین دیالیز بر کفایت دیالیز، شاخص‌های همودینامیک و خستگی در بیماران تحت همودیالیز در بیمارستان‌های شهر بوشهر در سال 1398	
عنوان پایان‌نامه (انگلیسی):	Comparison of the Effectiveness Physical Activity During Dialysis on the Dialysis Adequacy, Hemodynamic Indices and Fatigue in Hemodialysis Patients in Bushehr Hospitals in 2019	
مشخصات استاد راهنما (محقق اصلی):	نام و نام خانوادگی: دکتر شهناز پولادی<br>آدرس الکترونیک: pouladi2008@yahoo.com	
مشخصات دانشجو:	نام و نام خانوادگی: محمود محمدی زاده<br>آدرس الکترونیک: mohamadizadeh55@gmail.com	

دکتر سعید کشمیری
رئیس کمیته دانشگاهی/منطقه ای اخلاق در پژوهش‌های زیست پزشکی
دانشگاه علوم پزشکی بوشهر

دکتر غلامرضا خمیسی پور
دبیر کمیته دانشگاهی/منطقه ای اخلاق در پژوهش‌های زیست پزشکی
دانشگاه علوم پزشکی بوشهر

Abstract

Background: Patients with chronic renal failure undergoing hemodialysis are associated with decreasing function and reduceing quality of life .Activity and rehabilitation are very important to prevent a decrease in muscle strength and performance of the patient on dialysis. On the other hand, the long-term prognosis of dialysis patients depends entirely on the quality of their dialysis. Therefore, the present study was designed to Compared of the Effectiveness Physical Activity during Dialysis on the Dialysis Adequacy, Hemodynamic Indices and Fatigue in Hemodialysis Patients in Bushehr Hospitals in 2019

Methods: This study is a randomized clinical trial study that was performed on 84 patients undergoing hemodialysis in Bushehr hospitals by simple random Allocation in experimental (n = 42) and control (n = 42) groups. Dialysis time was 4 hours in both groups. The patient's physical activity was performed by pedaling for fifteen seconds in two shifts, taking into account fifteen rests between exercises. Study variables were assessed before and after the intervention by using the tools whose validity and reliability were assessed. data analyzed by using descriptive and inferential statistical methods including chi-square test, Fisher's exact test, Mann-Whitney U test and t-test by using SPSS.ver24 software.

Result: The results showed that there was no statistically significant difference between patients in the experimental and control groups in terms of demographic information except patients' age ($p = 0.02$) and education ($p = 0.02$). Also there was no significant difference between the two groups in terms of blood urea level ($p = 0.13$), changes in blood urea nitrogen (BUN) ($p = 0.07$) and weight ($p = 0.82$) of patients ;By adjusting the effect of age and education, the mean changes in hemodynamic parameters (temperature, pulse, blood pressure, respiration and arterial blood oxygen saturation) between the experimental and control groups showed that there was no significant difference between the two groups ($p <0.05$);Also, comparison of patients' mean Kt / V did not show a significant difference between the experimental and control groups ($p = 0.11$); The mean of patient fatigue before the intervention ($p = 0.001$) and after the intervention ($p = 0.001$) showed a significant difference between the experimental and control groups. The mean of patient fatigue in the experimental group was less than the control group.

Conclusion: The results of the present study showed that physical activity during hemodialysis has not been effective on dialysis adequacy and changes in hemodynamic parameters in patients undergoing hemodialysis; however, physical activity during hemodialysis increased the fatigue of hemodialysis patients. Therefore, it seems that physical activity can be considered as an intervention to improve the condition of hemodialysis patients, but due to the lack of effect on dialysis adequacy and increased fatigue of hemodialysis patients, special attention should be paid to the type of intervention, duration and duration.

Keywords: chronic renal failure, Dialysis adequacy, Hemodialysis, Fatigue, Hemodynamic Parameters, Physical activity,

Bushehr University of Medical Sciences and Health Services
School of Nursing and Midwifery
Nursing education group

MSc Thesis in Medical- Surgical Nursing

Comparison of the Effectiveness Physical Activity during Dialysis on the Dialysis Adequacy, Hemodynamic Indices and Fatigue in Hemodialysis Patients in Bushehr Hospitals in 2019

Student
Mahmoud Mohamadizadeh

Supervisor
Dr. Shahnaz Pouladi

Consultant Professors
Sharif Sharifi
Dr. Niloufar Motamed

January 2022
