# Supplementary material for: Effect of intra-dialytic pedaling exercise on dialysis adequacy: A randomized controlled trial
Source: PLoS One. 2026 May 15;21(5):e0348063. doi: 10.1371/journal.pone.0348063 (PMC13178916; doi:10.1371/journal.pone.0348063)
Supplement: S3 File — This document outlines the original protocol approved by the institutional ethics committee, including study design, intervention procedures, and outcome measures. (DOCX) [file pone.0348063.s003.docx]

Protocol for the Study on the Effects of Physical Activity During Hemodialysis on Dialysis Adequacy

**1. Introduction**

Chronic kidney disease (CKD) patients undergoing hemodialysis often experience reduced physical function and quality of life. Intradialytic physical activity and rehabilitation are essential to mitigate muscle weakness and functional decline in these patients. This study aims to assess the effectiveness of intradialytic physical activity, specifically pedaling, on dialysis adequacy in hemodialysis patients.

**2. Objectives**

2.1 Primary Objective

To determine the impact of intradialytic pedaling on the quality of dialysis in hemodialysis patients, as measured by Kt/V.

**3. Research Question**

1. Does intradialytic pedaling improve the quality of dialysis in hemodialysis patients?

**4. Hypothesis**

1. Intradialytic pedaling will improve the quality of dialysis in hemodialysis patients.

**5. Methods**

5.1 Study Design

This study is a randomized controlled trial (RCT) to evaluate the impact of intradialytic pedaling on dialysis adequacy in patients undergoing hemodialysis. The study was conducted in the hospitals of Bushehr in 2020. The study protocol was approved by the Ethics Committee of Bushehr University of Medical Sciences before commencement (Approval number: IR.BPUMS.REC.1398.130).

5.2 Participants

A total of 84 patients undergoing hemodialysis were recruited for this study. Participants were randomly assigned to either the intervention group (n = 42) or the control group (n = 42) using simple randomization.

5.3 Inclusion Criteria

1. Patients undergoing hemodialysis.

2. Signed informed consent.

5.4 Exclusion Criteria

1. Unwillingness to participate in the study.

5.5 Sample Size and Participant Flow

Initially, 84 patients (42 in the intervention group and 42 in the control group) were enrolled in the study. During the intervention, two participants from the intervention group and three from the control group withdrew due to a lack of willingness to continue. The final analysis included data from 40 patients in the intervention group and 39 patients in the control group.

5.6 Data Collection

Before the intervention, demographic data were collected. Dialysis adequacy, as measured by Kt/V, was assessed before and after the intervention.

5.7 Intervention

The intervention group participated in intradialytic pedaling during their regular 4-hour hemodialysis sessions. The exercise protocol consisted of pedaling in bed for two 15-minute sessions per dialysis session, with a 15-minute rest period between exercise bouts. The exercise was performed three times per week (during hemodialysis sessions) at a light intensity. The control group received standard care without any structured physical activity during their hemodialysis sessions.

5.8 Data Analysis

Data were analyzed using SPSS version 24. Descriptive statistics were used to summarize demographic and clinical characteristics. Independent t-tests and Chi-square tests were used to compare baseline characteristics between the two groups. Paired t-tests were used to assess changes within each group from baseline to post-intervention. Analysis of covariance (ANCOVA) was used to compare the differences between the intervention and control groups, adjusting for potential confounding variables (age and education level). A p-value of less than 0.05 was considered statistically significant.

5.9 Ethical Considerations

The study was conducted under the ethical principles stated in the Declaration of Helsinki. The study was approved by the Ethics Committee of Bushehr University of Medical Sciences (Approval number: IR.BPUMS.REC.1398.130). All participants provided written informed consent before enrollment.

**6. Deviations from the approved protocol**

There were no significant deviations from the approved study protocol.

**7. Supporting Information**

The original study protocol in Persian, as approved by the Ethics Committee of Bushehr University of Medical Sciences, is submitted as a supporting information file. An English translation of the protocol is also provided. The Ethics Committee approval letter is also provided as a supporting document.

**8. Expected Outcomes**

This study is expected to provide evidence on the effectiveness of intradialytic physical activity in improving dialysis adequacy in hemodialysis patients. The findings will inform clinical practice and contribute to the development of guidelines for intradialytic exercise programs.
