# Supplementary material for: Effect of intra-dialytic pedaling exercise on dialysis adequacy: A randomized controlled trial
Source: PLoS One. 2026 May 15;21(5):e0348063. doi: 10.1371/journal.pone.0348063 (PMC13178916; doi:10.1371/journal.pone.0348063)
Supplement: S4 File — This file contains the official ethics approval letter issued by the Research Ethics Committee of Bushehr University of Medical Sciences for the submitted study. (PDF) [file pone.0348063.s004.pdf]

Bushehr province university of medical sciences

Research Ethics Certificate

Approval ID:	IR.BPUMS.REC.1398.130	Approval Date:	2019-12-22
Evaluated by:	Bushehr province university of medical sciences		
Status:	Approved		
Approval Statement:	The project was found to be in accordance to the ethical principles and the national norms and standards for conducting Medical Research in Iran.<br>Notice:<br>1. Although the proposal has been approved by the research ethics committee, meeting the professional and legal requirements is the sole responsibility of the PI and other project collaborators.<br>2. This certificate is reliant on the proposal/documents received by this committee on 2019-12-22. The committee must be notified by the PI as soon as the proposal/documents are modified.		
Thesis Title:	Comparison of the Effectiveness Physical Activity During Dialysis on the Dialysis Adequacy, Hemodynamic Indices and Fatigue in Hemodialysis Patients in Bushehr Hospitals in 2019		
Thesis Adviser (PI):	Name: shahnaz pouladi<br>Email: pouladi2008@yahoo.com		
Student:	Name: mahmod mohamadizadeh<br>Email: mohamadizadeh55@gmail.com		

Dr. Saeed Keshmiri
Director of University/Regional Research Ethics Committee
Bushehr province university of medical sciences

Dr. GHolamreza KHamisipour
Secretary of University/Regional Research Ethics Committee
Bushehr province university of medical sciences
