# Supplementary material for: Effect of intra-dialytic pedaling exercise on dialysis adequacy: A randomized controlled trial
Source: PLoS One. 2026 May 15;21(5):e0348063. doi: 10.1371/journal.pone.0348063 (PMC13178916; doi:10.1371/journal.pone.0348063)
Supplement: Attachment — Submitted filename: PONE-D-25-17351_reviewer (1).pdf [file pone.0348063.s006.pdf]

PLOS ONE

Effect of Intra-Dialytic Pedaling Exercise on Dialysis Adequacy: A Randomized Controlled Trial

--Manuscript Draft--

**Manuscript Number:**	PONE-D-25-17351
**Article Type:**	Clinical Trial
**Full Title:**	Effect of Intra-Dialytic Pedaling Exercise on Dialysis Adequacy: A Randomized Controlled Trial
**Short Title:**	Short Title: Intra-Dialytic Pedaling Exercise and Dialysis Adequac
**Corresponding Author:**	Shahnaz Pouladi, ph.D<br>Bushehr University of Medical Sciences<br>Bushehr, Bushehr IRAN, ISLAMIC REPUBLIC OF
**Keywords:**	physical activity, dialysis adequacy, chronic kidney disease, hemodialysis
**Abstract:**	Background: In patients with chronic kidney disease undergoing hemodialysis, physical activity and rehabilitation are crucial to prevent the reduction of muscle strength and functional capacity. This study aimed to determine the effectiveness of physical activity during hemodialysis on dialysis adequacy in hemodialysis patients.<br>Methods: This randomized controlled trial (RCT) investigated the impact of pedaling exercise on dialysis effectiveness in 84 hemodialysis patients at Bushehr hospitals. Participants were randomly assigned to either an experimental group (n=42) that performed 30 minutes of pedaling exercise during their 4-hour dialysis sessions or a control group (n=42) that received routine hemodialysis. Dialysis adequacy was assessed by comparing pre-and post-dialysis blood samples taken from the arterial line. A standardized protocol was used for collecting blood samples, and patient weight was measured using a calibrated digital scale. Data analysis was performed using SPSS.ver24 software.<br>Results: The experimental and control groups were similar in demographic characteristics, except for age (X2= -3.84, p=0.001) and education levels (X2= 10.100, p=0.006). While there was no significant difference in weight before and after the intervention between the groups (t=0.223, p=0.82 before; t=0.203, p=0.84 after), both groups showed a substantial weight reduction overall (p<0.0001). There were no statistically significant differences in weight change (t=0.80, p=0.25), blood urea nitrogen (BUN) (t=0.13, p=1.52), or Kt/V (t=1.62, p=0.11) between the experimental and control groups.<br>Conclusion: This study found that incorporating pedaling exercise during hemodialysis did not significantly improve dialysis effectiveness, as measured by weight change, BUN levels, or Kt/V. While both groups showed weight loss, there were no statistically significant differences between them. However, the study had limitations due to its small sample size and the specific type of exercise used. Further research with larger cohorts and a wider variety of physical activities is needed to determine if physical activity during hemodialysis can benefit dialysis adequacy and overall patient outcomes.<br>Trial registration: IRCT code number 20150529022466N15 and trial Code of Ethics IR.BPUMS.REC.1398.130
**Order of Authors:**	Shahnaz Pouladi, ph.D
	Mahmoud Mohamadizadeh, M.Sc. student
	Sharif Sharifi, lecturer
	Niloufar Motamed, Associate professor
	Mohammad Amin Shadman, lecturer
**Additional Information:**	
**Question**	**Response**
**Financial Disclosure**	The author(s) received no specific funding for this work.

Powered by Editorial Manager® and ProduXion Manager® from Aries Systems Corporation

Enter a financial disclosure statement that describes the sources of funding for the work included in this submission. Review the submission guidelines for detailed requirements. View published research articles from *PLOS ONE* for specific examples.<br><br>This statement is required for submission and **will appear in the published article** if the submission is accepted. Please make sure it is accurate.<br><br>**Funded studies**<br>Enter a statement with the following details:<br>• Initials of the authors who received each award<br>• Grant numbers awarded to each author<br>• The full name of each funder<br>• URL of each funder website<br>• Did the sponsors or funders play any role in the study design, data collection and analysis, decision to publish, or preparation of the manuscript?<br><br>Did you receive funding for this work?	
**Competing Interests**<br><br>Use the instructions below to enter a competing interest statement for this submission. On behalf of all authors, disclose any competing interests that could be perceived to bias this work—acknowledging all financial support and any other relevant financial or non-financial competing interests.<br><br>This statement is required for submission and **will appear in the published article** if the submission is accepted. Please make sure it is accurate and that any funding sources listed in your Funding Information later in the submission form are also declared in your Financial Disclosure statement.	The authors have declared that no competing interests exist.

View published research articles from *PLOS ONE* for specific examples.<br><br>**NO authors have competing interests**<br><br>Enter: *The authors have declared that no competing interests exist.*<br><br>**Authors with competing interests**<br><br>Enter competing interest details beginning with this statement:<br><br>*I have read the journal's policy and the authors of this manuscript have the following competing interests: [insert competing interests here]*<br><br>* typeset	
**Ethics Statement**<br><br>Enter an ethics statement for this submission. This statement is required if the study involved:<br><br>• Human participants<br>• Human specimens or tissue<br>• Vertebrate animals or cephalopods<br>• Vertebrate embryos or tissues<br>• Field research<br><br>Write "N/A" if the submission does not require an ethics statement.<br><br>General guidance is provided below. Consult the submission guidelines for detailed instructions. **Make sure that all information entered here is included in the Methods section of the manuscript.**	The study protocol was approved by the Ethics Committee of Bushehr University of Medical Sciences (ethics code number IR.BPUMS.REC.1398.130). Written informed consent was obtained from all participants prior to their enrollment in the study. Participants were informed of their right to withdraw from the study at any time without any negative consequences. The confidentiality of their data was strictly maintained throughout the study."

Powered by Editorial Manager® and ProduXion Manager® from Aries Systems Corporation

**Format for specific study types**<br><br>**Human Subject Research (involving human participants and/or tissue)**<br>• Give the name of the institutional review board or ethics committee that approved the study<br>• Include the approval number and/or a statement indicating approval of this research<br>• Indicate the form of consent obtained (written/oral) or the reason that consent was not obtained (e.g. the data were analyzed anonymously)<br><br>**Animal Research (involving vertebrate animals, embryos or tissues)**<br>• Provide the name of the Institutional Animal Care and Use Committee (IACUC) or other relevant ethics board that reviewed the study protocol, and indicate whether they approved this research or granted a formal waiver of ethical approval<br>• Include an approval number if one was obtained<br>• If the study involved *non-human primates*, add *additional details* about animal welfare and steps taken to ameliorate suffering<br>• If anesthesia, euthanasia, or any kind of animal sacrifice is part of the study, include briefly which substances and/or methods were applied<br><br>**Field Research**<br><br>Include the following details if this study involves the collection of plant, animal, or other materials from a natural setting:<br>• Field permit number<br>• Name of the institution or relevant body that granted permission	
**Data Availability**<br><br>Authors are required to make all data underlying the findings described fully available, without restriction, and from the time of publication. PLOS allows rare exceptions to address legal and ethical concerns. See the PLOS Data Policy and FAQ for detailed information.	Yes - all data are fully available without restriction

A Data Availability Statement describing where the data can be found is required at submission. Your answers to this question constitute the Data Availability Statement and **will be published in the article**, if accepted.<br><br>**Important:** Stating 'data available on request from the author' is not sufficient. If your data are only available upon request, select 'No' for the first question and explain your exceptional situation in the text box.<br><br>Do the authors confirm that all data underlying the findings described in their manuscript are fully available without restriction?	
**Describe where the data may be found in full sentences. If you are copying our sample text, replace any instances of XXX with the appropriate details.**<br><br>• If the data are **held or will be held in a public repository**, include URLs, accession numbers or DOIs. If this information will only be available after acceptance, indicate this by ticking the box below. For example: *All XXX files are available from the XXX database (accession number(s) XXX, XXX.*).<br>• If the data are all contained **within the manuscript and/or Supporting Information files**, enter the following: *All relevant data are within the manuscript and its Supporting Information files.*<br>• If neither of these applies but you are able to provide **details of access elsewhere**, with or without limitations, please do so. For example:<br><br>*Data cannot be shared publicly because of [XXX]. Data are available from the XXX Institutional Data Access / Ethics Committee (contact via XXX) for researchers who meet the criteria for access to confidential data.*<br><br>*The data underlying the results presented in the study are available from (include the name of the third party*	"The datasets generated and analyzed during the current study are available in the library of Bushehr University of Medical Sciences, in the thesis of a Master of Nursing student named Mahmoud Mohammadizadeh. The full trial protocol is also available in this thesis. Interested researchers may contact the corresponding author to request access to this data

and contact information or URL).<br>• This text is appropriate if the data are owned by a third party and authors do not have permission to share the data.<br><br>* typeset	
Additional data availability information:	Tick here if the URLs/accession numbers/DOIs will be available only after acceptance of the manuscript for publication so that we can ensure their inclusion before publication.

Powered by Editorial Manager® and ProduXion Manager® from Aries Systems Corporation

Manuscript Click here to access/download;Manuscript;Manuscript (8).docx

Effect of Intra-Dialytic Pedaling Exercise on Dialysis Adequacy: A Randomized Controlled Trial

Mahmoud Mohamadizadeh (MSc)[1], Sharif Sharifi (MSc)[2], Niloufar Motamed (Ph.D)[3], Mohammad Amin Shadman(MSc)[4], Shahnaz Pouladi(Ph.D)*[5]

1. Master's student in medical-surgical nursing, student research committee, Bushehr University of Medical Sciences, Bushehr, Islamic Republic of Iran. Mahmoud.2580@yahoo.com
2. MSc in medical-surgical nursing, Nursing and Midwifery Faculty of Bushehr University of Medical Sciences, Bushehr, Islamic Republic of Iran.
3. PhD in community medicine, Department of Community Medicine, Medicine faculty of Bushehr University of Medical Sciences, Bushehr, Islamic Republic of Iran.
4. MSc in medical-surgical nursing, Nursing and Midwifery Faculty of Bushehr University of Medical Sciences, Bushehr, Islamic Republic of Iran.
5. PhD in Nursing Education, Associate Professor, Nursing and Midwifery Faculty of Bushehr University of Medical Sciences, Bushehr, Islamic Republic of Iran. pouladi2008@gmail.com

Abstract

Background: In patients with chronic kidney disease undergoing hemodialysis, physical activity and rehabilitation are crucial to prevent the reduction of muscle strength and functional capacity. This study aimed to determine the effectiveness of physical activity during hemodialysis on dialysis adequacy in hemodialysis patients.

Methods: This randomized controlled trial (RCT) investigated the impact of pedaling exercise on dialysis effectiveness in 84 hemodialysis patients at Bushehr hospitals. Participants were randomly assigned to either an experimental group (n=42) that performed 30 minutes of pedaling exercise during their 4-hour dialysis sessions or a control group (n=42) that received routine hemodialysis. Dialysis adequacy was assessed by comparing pre-and post-dialysis blood samples taken from the arterial line. A standardized protocol was used for collecting blood samples, and patient weight was measured using a calibrated digital scale. Data analysis was performed using SPSS.ver24 software.

Results: The experimental and control groups ~~were similar~~ in demographic characteristics, except for age (X2= -3.84, p=0.001) and education levels (X2= 10.100, p=0.006). While there was no significant difference in weight before and after the intervention between the groups (t=0.223, p=0.82 before; t=0.203, p=0.84 after), both groups showed a substantial weight reduction overall (p<0.0001). There were no statistically significant differences in weight change (t=0.80, p=0.25), blood urea nitrogen (BUN) (t=0.13, p=1.52), or Kt/V (t=1.62, p=0.11) between the experimental and control groups.

Conclusion: This study found that incorporating pedaling exercise during hemodialysis did not significantly improve dialysis effectiveness, as measured by weight change, BUN levels, or Kt/V. While both groups showed weight loss, there were no statistically significant differences between them. However, the study had limitations due to its small sample size and the specific type of exercise used. Further research with larger cohorts and a wider variety of physical activities is needed to determine if physical activity during hemodialysis can benefit dialysis adequacy and overall patient outcomes.

Trial registration: IRCT code number 20150529022466N15 and trial Code of Ethics IR.BPUMS.REC.1398.130

Keywords: physical activity, dialysis adequacy, chronic kidney disease, hemodialysis

Introduction

Chronic kidney disease (CKD) is a significant and dangerous global health concern, causing short- and long-term complications and reducing patients' quality of life (1, 2). Recent studies have demonstrated that the prevalence of CKD varies across different regions (3, 4), with estimates of 11.4% in Iran (5), 9% in Mexico (6), and 13.1% among adults in the United States (7). Various treatment options exist to manage CKD, including kidney transplantation, peritoneal dialysis, and hemodialysis (8). Nearly 4 million people worldwide are on kidney replacement therapy (KRT), with hemodialysis (HD) remaining the most common form, accounting for approximately 69% of all KRT and 89% of all dialysis (9). CKD is projected to become the fifth leading cause of death worldwide by 2040 (10). Compared to population growth, an annual growth rate of 5-6% in CKD prevalence poses a challenge for all countries globally. In Iran, this growth rate is higher than the average global rate (11). Although kidney transplantation is the preferred treatment for end-stage renal disease, hemodialysis remains the most common treatment worldwide (12).

Different hemodialysis programs exist, including home hemodialysis (3-6 times per week), in-center hemodialysis, short daily hemodialysis, and long nocturnal hemodialysis (7-9 hours). In-center hemodialysis is typically performed three times per week, with each session lasting between three and five hours (13). Although hemodialysis prevents death due to uremia, the survival rate of patients with CKD is significantly lower than that of the general population (14-16). Several factors influence the survival rate of these patients, including the cause of renal failure, the treatment method, the presence of comorbidities such as cardiovascular disease, diabetes, and hypertension, and the adequacy of dialysis (17, 18).

The long-term prognosis of patients undergoing dialysis depends on the efficacy of their dialysis treatment, serving as an indicator of mortality rates and a primary outcome of dialysis (19). Dialysis adequacy is defined by the National Kidney Foundation Kidney Disease Outcomes Quality Initiative (NKF KDOQI) as adequate urea clearance (20). Inadequate dialysis adequacy leads to an increased risk of complications, including sexual dysfunction, cardiovascular diseases, pruritus, prolonged hospitalization, and higher treatment costs (21, 22). Conversely, improved dialysis adequacy enhances patients' quality of life and physical and spiritual health (23).

The amount of dialysis performed during a session is calculated using Kt/V and the urea reduction ratio (URR) (24, 25). Kt/V is a measure of how well dialysis removes waste products, reflecting the ratio of urea clearance and dialysis time to urea volume of distribution (total body water) (26). The kinetic model of urea indicates that the Kt/V index and the urea reduction ratio are used for urea purification. The minimum Kt/V index is 1.2, and the target urea reduction ratio is approximately 65%. Kt/V represents the fraction of body water volume cleared of urea during a dialysis session (20).

Patients with CKD undergoing hemodialysis often experience functional decline, reduced quality of life, and psychological disorders such as depression and anxiety related to their treatment (27-30). Furthermore, hemodialysis is typically administered in a supine position, with patients remaining in this position for approximately 800 hours per year. This lack of physical activity can result in complications such as reduced physical endurance, muscle atrophy, and muscle weakness (31). Therefore, regular exercise during dialysis can enhance physical performance and reduce muscle atrophy in hemodialysis patients (32, 33).

Previous studies on physical activity during dialysis have yielded conflicting results. For example, Kirkman (2013) reported that pedaling exercise during dialysis did not significantly improve dialysis adequacy, although dialysis adequacy increased in patients with longer dialysis times (34). Riahi's 2012 study found that 40 to 60 minutes of pedaling exercise at the start of dialysis did not improve dialysis adequacy (35). However, Groussard's 2015 study demonstrated that 30 minutes of cycling increased physical fitness, improved lipid profiles, and enhanced antioxidant status (36). Given the crucial role of dialysis nurses in providing continuous and integrated care and monitoring patients and their significant influence in educating hemodialysis patients, the presence of dialysis nurses could be leveraged to implement effective interventions to promote and improve dialysis quality and patient outcomes, including physical, mental, and psychological well-being. Therefore, this study was conducted to investigate the effectiveness of physical activity during hemodialysis on dialysis adequacy in hemodialysis patients in Bushehr.

Methods

Study design

This study was a randomized controlled clinical trial (RCT), identified by the code IRCT20150529022466N15, conducted in 2020 with hemodialysis patients in the dialysis department of Bushehr hospitals in southern Iran. The study period lasted from January 10, 2020, to March 30, 2020.

Inclusion and exclusion criteria

Inclusion criteria were: age from 18 to 65 years, a history of hemodialysis for more than 3 months, ability to walk independently (at least 2 minutes), no history of unstable angina, dyspnea, or

myocardial infarction in the previous month, and no history of congestive heart failure; no treatment with neuropsychiatric drugs; tolerance of 4-hour dialysis sessions in previous treatments, and no history of hypokalemia ($K<3.5$ Meq /L).

Exclusion criteria included: the occurrence of cardiovascular complications such as unstable angina, an increase in systolic blood pressure greater than or equal to 160 mmHg or diastolic blood pressure greater than or equal to 120 mmHg, the occurrence of hypotension in the form of a decrease in systolic blood pressure of less than 90 mmHg or a decrease of more than 30 mmHg, or a fall in systolic blood pressure of more than 30 mmHg compared to initial blood pressure, the occurrence of any complication that prevents the continuation of the intervention, such as neurological, musculoskeletal, or vascular complications (with the doctor's consent), failure to attend 4 or more sessions during the trial, failure to perform hemodialysis due to death or kidney transplant, problems in vascular access, and unwillingness to continue participating in the trial.

Sample size and sampling method

Using G*Power 3.1.9.2 software, with a confidence level of 95% and a statistical power of 80%, the calculated sample size was 76 participants. The mean and standard deviation of the dialysis quality index in the control group were 1.33 and 0.18, respectively, while in the exercise group, the mean was 1.45 with the same standard deviation. Considering a 10% dropout rate (q) and applying the formula $n' = n/((1-q))$, the total sample size was increased to 84 participants, with 42 participants in each group. A convenience sampling method was used to recruit 84 patients admitted to the hemodialysis departments of Bushehr hospitals. To ensure random assignment to the experimental or control group, a simple randomization method was employed. A researcher not involved in patient recruitment used a computer-generated random number table to create the

allocation sequence. The allocation sequence was concealed from the researchers' enrolling participants until the point of assignment by using sequentially numbered, sealed opaque envelopes. Each envelope contained a card indicating the group assignment (experimental or control). After obtaining informed consent and confirming eligibility, the next sequentially numbered envelope was opened, and the participant was assigned to the corresponding group. This process ensured that the allocation was random and unbiased. The study was a single-blind trial, meaning that the participants were not aware of their group assignment (Figure 1). While participants were blinded, it was impossible to blind care providers due to the nature of the

Data collection tools

Data collection instruments included a demographic information form and a dialysis adequacy form. Patient demographics, including sex, age, education level, marital status, and weight before and after hemodialysis, were recorded. Dialysis adequacy was calculated from the Kt/V criterion using the formula:

$$Kt / V = -\ln (R - 0.008 * t) + (4 - 3.5 * R) * UF/W$$

Where: K: urea clearance, T: duration of dialysis, V: urea volume distribution, R: urea reduction coefficient (a function of the length of the session, approximately 0.024 to 0.032 in a dialysis session), and UF/W: ultrafiltration volume/weight.

The urea reduction ratio (URR) was calculated using the formula: (BUN before dialysis - BUN after dialysis) / BUN before dialysis.

Blood samples were collected immediately before the start of hemodialysis and from the arterial line. Post-dialysis samples were obtained by halting the dialysis solution flow for three minutes to cease urea extraction and reducing the pump speed to below 100 cc/min for the final five minutes of dialysis. The samples, which were anonymous and coded, were immediately sent to the central laboratory and processed by a designated technician using a DIRUI 1200 machine. Weight was measured using a Seca digital scale, calibrated annually by Peish Instrumental Monitoring.

Implementation

Following the approval of the study protocol by the Ethics Committee of Bushehr University of Medical Sciences (Code of Ethics IR.BPUMS.REC.1398.130), the researcher obtained a letter of introduction from the research assistant of the university and visited the dialysis departments of Bushehr Teaching Hospitals. After making the necessary arrangements, the researcher introduced the study and its objectives to potential participants, obtained written informed consent, and initiated the sampling process. All participants were assured that their data would be kept strictly confidential and that they could withdraw from the study without any adverse consequences. Eighty-four patients admitted to the hemodialysis departments of Bushehr hospitals were randomly assigned to either an experimental or a control group. The experimental group participated in physical activity during dialysis, while the control group underwent hemodialysis using the routine method. The duration of dialysis was identical in both groups (four hours). The physical activity consisted of pedaling on a stationary bike while in bed for 15 minutes in two sessions, with 15 minutes of rest between sessions. Before starting the intervention, participants warmed up for five minutes and cooled down by pedaling slowly. The intensity of the pedaling was adjusted to achieve a 10% increase in heart rate compared to the pre-exercise heart rate.

Before starting the exercise, the patient's blood pressure and pulse were measured and recorded. Blood pressure was also measured at the end of each 15-minute session. The patient's pulse was continuously monitored during the activity and was expected to increase by at least 10 percent compared to the pre-activity pulse. If this was not achieved, the patient was instructed to pedal faster. The safe range of pulse rate during exercise was set between 50 and 125 beats per minute. (The full trial protocol is available in the library of Bushehr University of Medical Science, in the thesis of a Master of Nursing student named Mahmoud Mohammadizadeh.). The stationary bike was from ATMED (Taiwan), weighed 3.5 kg, and was easily portable. The researcher provided the patient with a bike, and the patient was monitored by the treatment team during the activity.

The program in the experimental group lasted 4 weeks, with three sessions per week (37). All patients in the study underwent dialysis three days a week for four hours per session (38). The patients' dialysis adequacy was measured before and after the intervention (one month). After the start of the study, two participants in the experimental group and three in the control group were excluded. Finally, data from 40 participants in the experimental group and 39 in the control group were analyzed.

Ethical Considerations

This study originated from a master's thesis in medical-surgical nursing at the Faculty of Midwifery Nursing, Bushehr University of Medical Sciences (ethics code number IR.BPUMS.REC.1398.130) and is registered with the Iranian Clinical Trial Centre under IRCT 20150529022466N15. All participants were provided comprehensive information about the study, including its purpose, procedures, and potential risks and benefits. Written informed consent was obtained from all participants before their enrollment. Participants were informed of their right to

withdraw from the study at any time without any negative consequences. The confidentiality of all participants' data was maintained throughout the study. All methods were performed under the relevant guidelines and regulations, including the Declaration of Helsinki.

Data analysis

Following data collection, statistical analysis was conducted using SPSS version 24 (IBM Corp., Armonk, NY, USA). Descriptive statistics, including means, standard deviations, 95% confidence intervals, and frequencies, were calculated to summarize the data. Group comparisons of demographic variables were performed using chi-squared tests. One-way analysis of variance (ANOVA) was employed to compare means of continuous variables across groups, and Tukey's post hoc test was used for pairwise comparisons. Paired t-tests were used to assess within-group changes in continuous variables before and after the intervention. The normality of data was assessed using the Shapiro-Wilk test, and the ratio of skewness to its standard error was examined. The level of statistical significance was set at $p < 0.05$ for all analyses.

Results

Throughout the 4-week study period, no significant adverse events or complications were observed in either the experimental or control group. Specifically, there were no reports of increased blood pressure requiring intervention, hypotensive episodes, or any neurological, musculoskeletal, or vascular complications related to the exercise intervention. Two participants in the experimental group and three in the control group withdrew from the study due solely to a general unwillingness to continue participation. Therefore, data from 40 participants in the experimental group and 39 in the control group were analyzed.

The study included 79 participants, mostly male (n = 51, 64.6%) and married (n = 72, 91.2%). Regarding employment, 33 participants (43.1%) were employed, and 50 (63.3%) had attained an education level below a diploma. Baseline demographic characteristics, as summarized in Table 1, revealed a statistically significant difference in mean age between the control and experimental groups (p = 0.001), with the control group being older. There were no significant differences between the groups regarding marital status (p = 0.11) or occupation (p = 0.29). However, a significant difference was observed in education level between the control and experimental groups (p = 0.006).

Table 1. Comparison of patient demographic variables between test and control groups

Variable		Test	Control	Total	X^2	P-value
		**Number (percentage)**	**Number (percentage)**	**Number (percentage)**		
Gender	Male	29(72/5)	22(56/4)	51(64/6)	2/23	0/13
	Female	11(27/5)	17(43/6)	28(35/4)		
Marital status	Single	6(15)	1(2/6)	7(8/9)	*3/78	0/11
	Married	34(85)	38(97/4)	72(91/1)		
Occupation	Unemployed	15(85)	19(47/5)	34(42/5)	2/42	0/29
	Employed	16(40)	17(42/5)	33(41/3)		
	Retired	9(22/5)	4(10)	13(16/3)		
Level of education	Under diploma	19(47/5)	31(79/5)	50(63/5)	10/100	0/006
	diploma	10(25)	6(15/4)	16(20/3)		
	Academic	11(27/5)	2(5/1)	13(16/5)		
Age		**50/25±10/71	**58/64±8/55		-3/84	0/001

** Mean and standard deviation
*Fisher exact test

Table 2 shows no significant difference in blood urea nitrogen (BUN) levels between the experimental and control groups ($p = 0.13$). Furthermore, while both groups exhibited a significant reduction in weight from pre- to post-intervention ($p < 0.0001$), there were no statistically significant differences between the experimental and control groups in either pre-intervention weight ($p = 0.82$) or post-intervention weight ($p = 0.84$).

Table 2. Comparison of blood urea and weight in patients before and after the intervention between the test and control groups

Group		Test	Control	t	p-value
		Mean ± SD	Mean ± SD		
**Blood urea level**		0/616±0/049	0/601±0/040	1/52	0/13
**Weight**	**Before**	72/96±13/85	73/61±11/93	-0/223	0/82
	**After**	70/99±13/95	71/59±11/85	-0/203	0/84
**T**		11/0	13/80		
**p-value**		0/0001	0/0001		
**Blood urea nitrogen (BUN)**	**Before**	58/66±9/65	55/73±8/92	1/40	0/17
	**After**	22/53±4/69	22/18±4/51	0/34	0/74
**T**		34/08	37/49		
**p-value**		0/001	0/001		

Table 3 presents an analysis of Kt/V values, which indicates no significant difference between the experimental and control groups ($p = 0.11$).

Table 3. Comparison of mean Kt /V in patients after intervention between two test and control groups

Group	Test	Control	t	p-value
	Mean ± SD	Mean ± SD		
**Average Kt / V**	1/163±0/14	1/115±0/12	1/62	0/11

Discussion

Hemodialysis is a critical and widely used treatment for patients with end-stage renal disease (ESRD). These patients often experience a multitude of challenges stemming from kidney failure and treatment-related complications, including electrolyte imbalances, fatigue, hypotension, muscle cramps, digestive issues, pruritus, and diminished sexual function and quality of life (39). These complications collectively exert a detrimental impact on the overall quality of life for individuals undergoing hemodialysis (39). The present study, designed as an experimental investigation, sought to evaluate and compare the effectiveness of intradialytic physical activity on dialysis adequacy in hemodialysis patients within Bushehr hospitals during 2020.

The findings of this study indicated that physical activity during dialysis did not significantly affect blood urea levels in hemodialysis patients. This observation is consistent with certain prior investigations, yet it contrasts with others, highlighting the complexity of this issue. Vaithilingam et al.. (2004) (40) in Australia, for instance, reported a non-significant trend toward increased urea reduction ratio (URR) in the exercise group among chronic hemodialysis patients. Conversely, Zhao et al. (2020) (41) demonstrated a positive impact of exercise on blood urea levels in hemodialysis patients, thereby contradicting the present study's results.

A plausible explanation for the observed impact of physical activity on blood urea levels lies in the physiological response to exercise. During intradialytic activity, increased blood flow and enhanced muscle perfusion promote a greater exchange of substances between blood and muscle tissue. Consequently, waste products such as urea can more readily enter the bloodstream from the muscles, facilitating their subsequent removal via dialysis (42).

The present study also revealed that intradialytic physical activity had no significant effect on the average blood urea nitrogen (BUN) levels in hemodialysis patients. Similarly, Soltani et al. (2020) (43) found that an exercise intervention consisting of at least 30 minutes of cycling during each hemodialysis session for six months did not significantly impact BUN levels in hemodialysis patients. However, these results diverge from those of Zhao et al. (2020) (41), who reported a reduction in mean blood urea nitrogen (BUN) with exercise in hemodialysis patients. Furthermore, Foran et al. (2003) (44) observed increased BUN levels 4 hours post-exercise, potentially attributable to dehydration and decreased renal perfusion.

In the present study, no significant difference was found in the mean weight of patients before and after the intervention between the test and control groups. However, within both groups, the mean weight of patients after the intervention was significantly lower than before the intervention. These results contrast with those of Pujiastuti et al. (2020) (45), who demonstrated that exercise was effective in reducing weight in hemodialysis patients.

Several confounding factors, such as age, gender, and underlying comorbidities, can obscure the influence of physical activity on laboratory indicators of patients' blood (46), which may explain the heterogeneity in findings across different studies. Additionally, the challenges associated with

implementing and adhering to physical activity regimens among hemodialysis patients can contribute to variability in outcomes.

Regarding dialysis adequacy, the present study indicated that intradialytic physical activity had no significant effect on Kt/V levels in hemodialysis patients. Previous investigations have yielded conflicting results in this area. Hatef et al. (2017) (47) reported that intradialytic exercise and home-based walking did not significantly improve physical ability or dialysis adequacy in hemodialysis patients. This study, conducted on chronic patients undergoing dialysis for more than 3 months, involved 2 weeks of intradialytic activity at the beginning of hemodialysis and 6 weeks of home-based walking (47). Kirkman (2013) (34) similarly found that activity did not significantly affect changes in dialysis adequacy compared to normal dialysis time, but emphasized that increased dialysis time improved dialysis adequacy (Kt/V), suggesting that exercise during dialysis cannot replace or enhance the benefits of increased dialysis duration. Riahi (2012) (35) also found no significant effect of regular exercise on dialysis quality in hemodialysis patients, with no significant difference in dialysis adequacy observed. Among the causes of reducing dialysis efficiency, we can include end-venous stenosis, venous-arterial fistula, inappropriate and weak fistula, non-compliance with the regular dialysis program by the patient, cardiovascular diseases, hemodynamic instability, Infections, improper nutrition, and recirculation indicated (48).

However, these findings stand in contrast to those of Biabani et al. (2019) (49) and Parsons (2006) (42). Biabani et al. (2019) (49) demonstrated that training and implementation of Benson's muscle relaxation technique increased dialysis adequacy in patients undergoing hemodialysis, potentially by reducing catecholamine secretion and stress, creating a balance between the posterior and anterior hypothalamus, and reducing sympathetic nervous system activity, ultimately leading to

improved dialysis adequacy. Parsons (2006) (42) also found that pedaling for 60 minutes during the first 2 hours of dialysis was effective in increasing dialysis adequacy. However, in a study by Basiri Moghadam et al. (2016), 60 minutes of exercise in the first 2 hours of dialysis significantly increased dialysis adequacy in patients (50).

The reasons for these conflicting findings may lie in the nature and duration of the physical activity, as well as the variability in the intensity and frequency of exercise programs (47). The present study employed 15-minute pedaling sessions in 2 shifts, with 15 minutes of rest between exercises, for 4 weeks and 3 sessions per week (37, 38). However, some studies suggest that 3 sessions of 15 minutes may be insufficient for serum urea removal, with 2 sessions of 30 minutes potentially increasing urea removal by dialysis fluid. The difficulty in performing physical activity during dialysis, the limitations of increasing hemodialysis time due to patient intolerance, and the potential influence of different hemodialysis machines on dialysis quality are also important considerations.

Conclusion

In conclusion, the findings of this randomized controlled trial indicate that a four-week intradialytic exercise intervention, consisting of pedaling exercises performed during hemodialysis sessions, did not result in a ~~statistically significant~~ improvement in dialysis adequacy as measured by Kt/V ($p = 0.11$). Therefore, in light of the observed lack of influence of this specific physical activity protocol on dialysis adequacy, future research endeavors must prioritize a comprehensive re-evaluation of the intervention parameters. Key considerations for subsequent investigations should include the careful selection of exercise modalities, optimization of session duration, and determination of the most efficacious timing of exercise relative to the hemodialysis procedure.

Furthermore, rigorous control of confounding variables, such as patient age and educational attainment, is essential to minimize potential bias and ensure the validity of future findings. By systematically addressing these factors, future studies may be better positioned to identify effective strategies for integrating physical activity into the hemodialysis routine to improve patient outcomes.

Limitations

Several limitations should be considered when interpreting these findings, which affect both the internal and external validity of the study. First, the relatively small sample size (n=79 after dropouts) may have limited the study's power to detect smaller, but potentially clinically significant, differences between the groups. Future research should aim for larger sample sizes to increase statistical power. Second, the study focused solely on pedaling exercise performed at a relatively low intensity. The effects of other exercise modalities, such as resistance training, higher intensity aerobic exercise, or combined exercise programs, were not investigated, and the results may not apply to these interventions. Different exercise types and intensities might have different effects on dialysis adequacy and other patient outcomes. Third, the single-blinded design, in which participants were unaware of their group assignment, may have reduced the risk of participant-related bias. However, the lack of blinding for care providers and/or outcome assessors could have introduced some performance or detection bias. Fourth, the study population consisted of hemodialysis patients in Bushehr, Iran, with specific inclusion criteria (age 18-65, no severe comorbidities). Therefore, the results may not be generalizable to patients in other geographic locations, with different demographic characteristics, or with more complex medical conditions. The experimental and control groups were similar in demographic characteristics, except for age (X2=-3.84, p=0.001) and education levels (X2=10.100, p=0.006), which may have introduced

additional bias. Fifth, the use of convenience sampling may limit the generalizability of the findings to other hemodialysis populations. Participants were recruited from a single center in Bushehr. Finally, this study was conducted in a single center, and all patients were monitored by the same treatment team during the activity. The researcher provided the patient with a bike, and the patient was monitored by the treatment team during the activity, making the results difficult to generalize given different treatment teams, dialysis protocols, or staffing levels.

Acknowledgments

This article results from the Nursing Master's thesis with the financial support of Bushehr University of Medical Sciences. We would like to thank the hemodialysis patients in the Bushehr hospitals' dialysis department.

Conflict of interest :None declared.

References

1. Kumela Goro K, Desalegn Wolide A, Kerga Dibaba F, Gashe Fufa F, Wakjira Garedow A, Edilu Tufa B, et al. Patient Awareness, Prevalence, and Risk Factors of Chronic Kidney Disease among Diabetes Mellitus and Hypertensive Patients at Jimma University Medical Center, Ethiopia. BioMed Research International. 2019;2019:2383508.

2. Wang J, Yao J, Zhu X, Wang T, Lu J, Wei Q, et al. Impact of frequent intradialytic hypotension on quality of life in patients undergoing hemodialysis. BMC Nephrology. 2023;24(1):209.

3. Kovesdy CP. Epidemiology of chronic kidney disease: an update 2022. Kidney International Supplements. 2022;12(1):7-11.

4. Valdivia-Cerda V, Alvarez-Zavala M, Sánchez-Reyes K, Cabrera-Silva RI, Ruiz-Herrera VV, Loza-Salazar AD, et al. Prevalence and risk factors of chronic kidney disease in an HIV positive Mexican cohort. BMC Nephrology. 2021;22(1):317.

5. Alvirdizadeh S, Yuzbashian E, Mirmiran P, Eghtesadi S, Azizi F. A prospective study on total protein, plant protein and animal protein in relation to the risk of incident chronic kidney disease. BMC nephrology. 2020;21(1):1-7.

6. Vasquez-Jimenez E, Madero M. Global Dialysis Perspective: Mexico. Kidney360. 2020;1(6):534-7.

7. Ammirati AL. Chronic kidney disease. Revista da Associação Médica Brasileira. 2020;66:s03-s9.

8. Zhang L, Guo Y, Ming H. Effects of hemodialysis, peritoneal dialysis, and renal transplantation on the quality of life of patients with end-stage renal disease. Revista da Associação Médica Brasileira. 2020;66:1229-34.

9. Bello AK, Okpechi IG, Osman MA, Cho Y, Htay H, Jha V, et al. Epidemiology of haemodialysis outcomes. Nature Reviews Nephrology. 2022;18(6):378-95.

10. Council E-E, Group EW. Chronic kidney disease is a key risk factor for severe COVID-19: a call to action by the ERA-EDTA. Nephrology Dialysis Transplantation. 2020;36(1):87-94.

11. Izadi Avanji FS, Masoudi Alavi N, Akbari H, Saroladan S. Self-Care and Its Predictive Factors in Hemodialysis Patients. J Caring Sci. 2021;10(3):153-9.

12. Lee H-J, Son Y-J. Prevalence and associated factors of frailty and mortality in patients with end-stage renal disease undergoing hemodialysis: a systematic review and meta-analysis. International Journal of Environmental Research and Public Health. 2021;18(7):3471.

13. Daugirdas JT, Depner TA, Inrig J, Mehrotra R, Rocco MV, Suri RS, et al. KDOQI clinical practice guideline for hemodialysis adequacy: 2015 update. American Journal of Kidney Diseases. 2015;66(5):884-930.

14. Himmelfarb J, Vanholder R, Mehrotra R, Tonelli M. The current and future landscape of dialysis. Nature Reviews Nephrology. 2020;16(10):573-85.

15. Rhee EP, Guallar E, Hwang S, Kim N, Tonelli M, Moe SM, et al. Prevalence and persistence of uremic symptoms in incident dialysis patients. Kidney360. 2020;1(2):86.

16. Ozturk S, Turgutalp K, Arici M, Odabas AR, Altiparmak MR, Aydin Z, et al. Mortality analysis of COVID-19 infection in chronic kidney disease, haemodialysis and renal transplant patients compared with patients without kidney disease: a nationwide analysis from Turkey. Nephrology Dialysis Transplantation. 2020;35(12):2083-95.

17. Dehghani M, Rahimi B, Makhdomi K, Jebraeily M. Determining Key Performance Indicators in Dialysis Management Dashboard for Monitoring Service Quality. Journal of Modern Medical Information Sciences. 2023;9(1):46-55.

18. Hong W-p, Lee Y-J. The association of dialysis adequacy, body mass index, and mortality among hemodialysis patients. BMC Nephrology. 2019;20(1):382.

19. Saad MM, El Douaihy Y, Boumitri C, Rondla C, Moussaly E, Daoud M, et al. Predictors of quality of life in patients with end-stage renal disease on hemodialysis. Int J Nephrol Renovasc Dis. 2015;8:119-23.

20. KDOQI Clinical Practice Guideline for Hemodialysis Adequacy: 2015 update. Am J Kidney Dis. 2015;66(5):884-930.

21. Kalantar-Zadeh K, Lockwood MB, Rhee CM, Tantisattamo E, Andreoli S, Balducci A, et al. Patient-centred approaches for the management of unpleasant symptoms in kidney disease. Nature Reviews Nephrology. 2022;18(3):185-98.

22. Dehvan F, Monjazebi F, Erfani Khanghahi M, Mohammadi H, Ghanei Gheshlagh R, Kurdi A. Adequacy of Dialysis in Iranian Patients Undergoing Hemodialysis: A Systematic Review and Meta-Analysis. Nephro-Urol Mon. 2018;10(5):e82235.

23. Aghili SM, Hadidi Z, Asghari A, namazi m. The relationship between dialysis adequacy and happiness and life expectancy and spiritual well-being in hemodialysis patients. Zanko Journal of Medical Sciences. 2022;22(75):37-47.

24. Colussi G, Brunati CCM, Gervasi F, Montoli A, Vergani D, Curci F, et al. A simple method for the calculation of dialysis Kt factor as a quantitative measure of removal efficiency of uremic retention solutes: Applicability to high-dialysate vs low-dialysate volume technologies. PLOS ONE. 2020;15(5):e0233331.

25. Liang KV, Zhang JH, Palevsky PM. Urea reduction ratio may be a simpler approach for measurement of adequacy of intermittent hemodialysis in acute kidney injury. BMC nephrology. 2019;20:1-7.

26. Rees L. Assessment of dialysis adequacy: beyond urea kinetic measurements. Pediatric Nephrology. 2019;34(1):61-9.

27. Ma S-J, Wang W-J, Tang M, Chen H, Ding F. Mental health status and quality of life in patients with end-stage renal disease undergoing maintenance hemodialysis. Annals of Palliative Medicine. 2021;10(6):6112-21.

28. Al-Nashri F, Almutary H. Impact of anxiety and depression on the quality of life of haemodialysis patients. Journal of clinical nursing. 2022;31(1-2):220-30.

29. Hall RK, Cary MP, Washington TR, Colón-Emeric CS. Quality of life in older adults receiving hemodialysis: a qualitative study. Quality of Life Research. 2020;29(3):655-63.

30. Ishiwatari A, Yamamoto S, Fukuma S, Hasegawa T, Wakai S, Nangaku M. Changes in quality of life in older hemodialysis patients: a cohort study on dialysis outcomes and practice patterns. American Journal of Nephrology. 2020;51(8):650-8.

31. Bastani M, Ghasemi G, Sadeghi M, Afshon A, Sadeghi H. The effect of selected core stability exercises on balance and muscle endurance in the elderly patients undergoing hemodialysis. Physical Treatments-Specific Physical Therapy Journal. 2017;7(2):89-96.

32. Deligiannis A, D'Alessandro C, Cupisti A. Exercise training in dialysis patients: impact on cardiovascular and skeletal muscle health. Clinical Kidney Journal. 2021;14(Supplement_2):ii25-ii33.

33. Torres E, Aragoncillo I, Moreno J, Vega A, Abad S, García-Prieto A, et al. Exercise training during hemodialysis sessions: Physical and biochemical benefits. Therapeutic Apheresis and Dialysis. 2020;24(6):648-54.

34. Kirkman DL, Roberts LD, Kelm M, Wagner J, Jibani MM, Macdonald JH. Interaction between intradialytic exercise and hemodialysis adequacy. Am J Nephrol. 2013;38(6):475-82.

35. Riahi Z, Esfarjani F, Marandi SM, Bayat A, Kalali N. The effects of regular exercise program on dialysis efficacy, muscle atrophy and physical performance in hemodialysis patients Journal of Shahrekord University of Medical Sciences 2012;14(5):63-73.

36. Groussard C, Rouchon-Isnard M, Coutard C, Romain F, Malardé L, Lemoine-Morel S, et al. Beneficial effects of an intradialytic cycling training program in patients with end-stage kidney disease. Appl Physiol Nutr Metab. 2015;40(6):550-6.

37. nezamlo a, Oshvand K, Ghlyaf M, Homayounfar S. The effects of physical training during dialysis on the prevention of anemia in hemodialysis patients. Avicenna Journal of Nursing and Midwifery Care. 2015;23(4):65-76.

38. American College of Sports M, Thompson WR, Gordon NF, Pescatello LS. ACSM's guidelines for exercise testing and prescription. 8th ed ed. Philadelphia: Wolters Kluwer/Lippincott Williams & Wilkins Philadelphia; 2010.

39. Saglimbene V, Natale P, Palmer S, Scardapane M, Craig JC, Ruospo M, et al. The prevalence and correlates of low sexual functioning in women on hemodialysis: A multinational, cross-sectional study. PLoS One. 2017;12(6):e0179511.

40. Vaithilingam I, Polkinghorne KR, Atkins RC, Kerr PG. Time and exercise improve phosphate removal in hemodialysis patients. American Journal of Kidney Diseases. 2004;43(1):85-9.

41. Zhao J, Qi Q, Xu S, Shi D. Combined aerobic resistance exercise improves dialysis adequacy and quality of life in patients on maintenance hemodialysisClin Nephrol. 2020;93(6):275-82.

42. Parsons TL, Toffelmire EB, King-VanVlack CE. Exercise training during hemodialysis improves dialysis efficacy and physical performance. Arch Phys Med Rehabil. 2006;87(5):680-7.

43. Soltani P, Saeedi N, Mashaykhi N, Rostami A, Tajfar M. Evaluating the effect of intradialytic exercise on blood pressure, inflammatory markers and dialysis adequacy. J Prev Epidemiol. 2020;5(2):e21-e.

44. Foran SE, Lewandrowski KB, Kratz A. Effects Of Exercise On Laboratory Test Results. Laboratory Medicine. 2003;34(10):736-42.

45. Pujiastuti TT, Aima H, Lokonathan S, Chiew L. Effectiveness of intradialytic exercise to reduce interdialytic weight gain in patients on hemodialysis at Private Hospital Yogyakarta. Enfermería Clínica. 2020;30:17-20.

46. Jafari F, Hashemi N, Reisi M. The effect of diet training on variations in blood pressure, weight, and some biochemical factors in hemodialysis patients: a clinical trial. Journal of Clinical Nursing and Midwifery. 2015;3(4):13-9.

47. Hatef M, Esmaeili R, Mousavinasab N, Madani Z, Spahbodi F, Shafipour V. Effect of Intradialytic and Home-Based Walking Exercises on Physical Function and Dialysis Adequacy in Hemodialysis Patients. Journal of Mazandaran University of Medical Sciences. 2017;27(154):83-93.

48. Motedayen Z, Nehrir B, Tayebi A, Ebadi A, Einollahi B. The effect of the physical and mental exercises during hemodialysis on fatigue: a controlled clinical trial. Nephrourol Mon. 2014;6(4):e14686.

49. biabani f, Moghrrab M, Nasirizadeh M. The effect of muscle relaxation on nausea and dialysis adequacy in hemodialysis patient. Journal of Sabzevar University of Medical Sciences. 2019;26(3):303-9.

50. Basiri Moghaddam M., Mohamadpour A., Mottaghi M.R., Jahani Maghani F. Impact of Isometric and Isotonic Movements during Hemodialysis on Dialysis Adequacy. Internal Medicine Today. 2016;22(1):35-41.

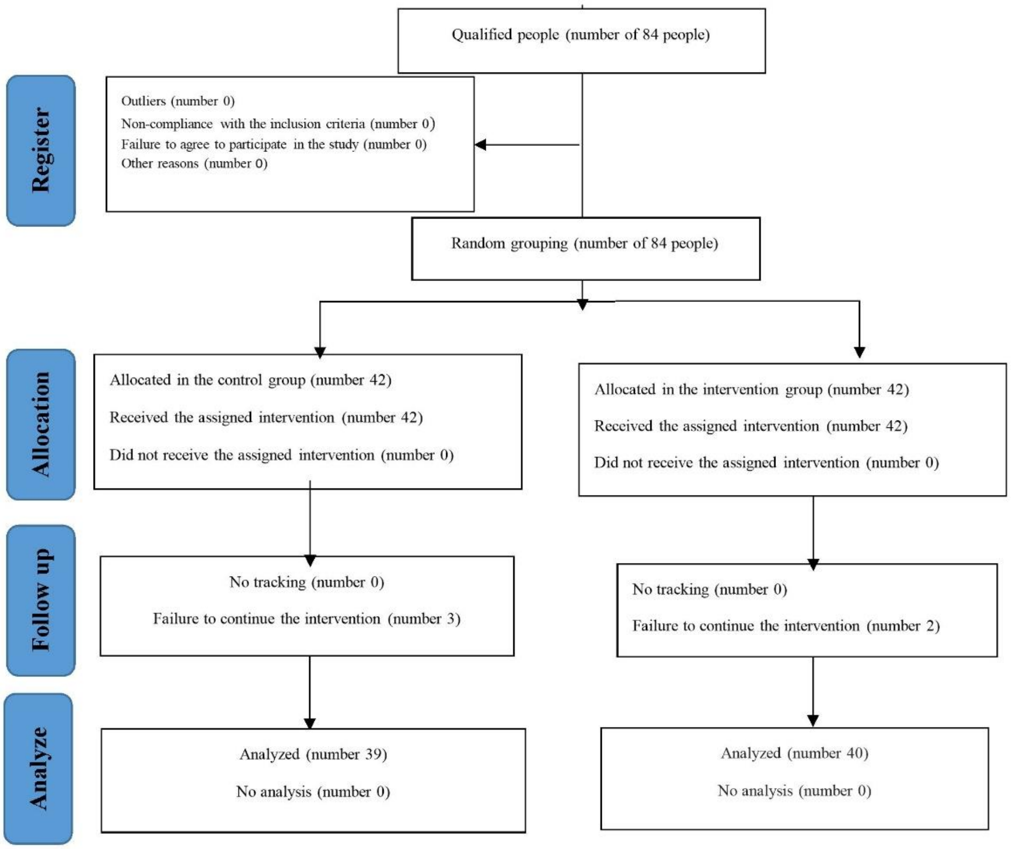

Figure 1: Consortium diagram

Figure

Click here to access/download;Figure;consort diagram jpg..jpg

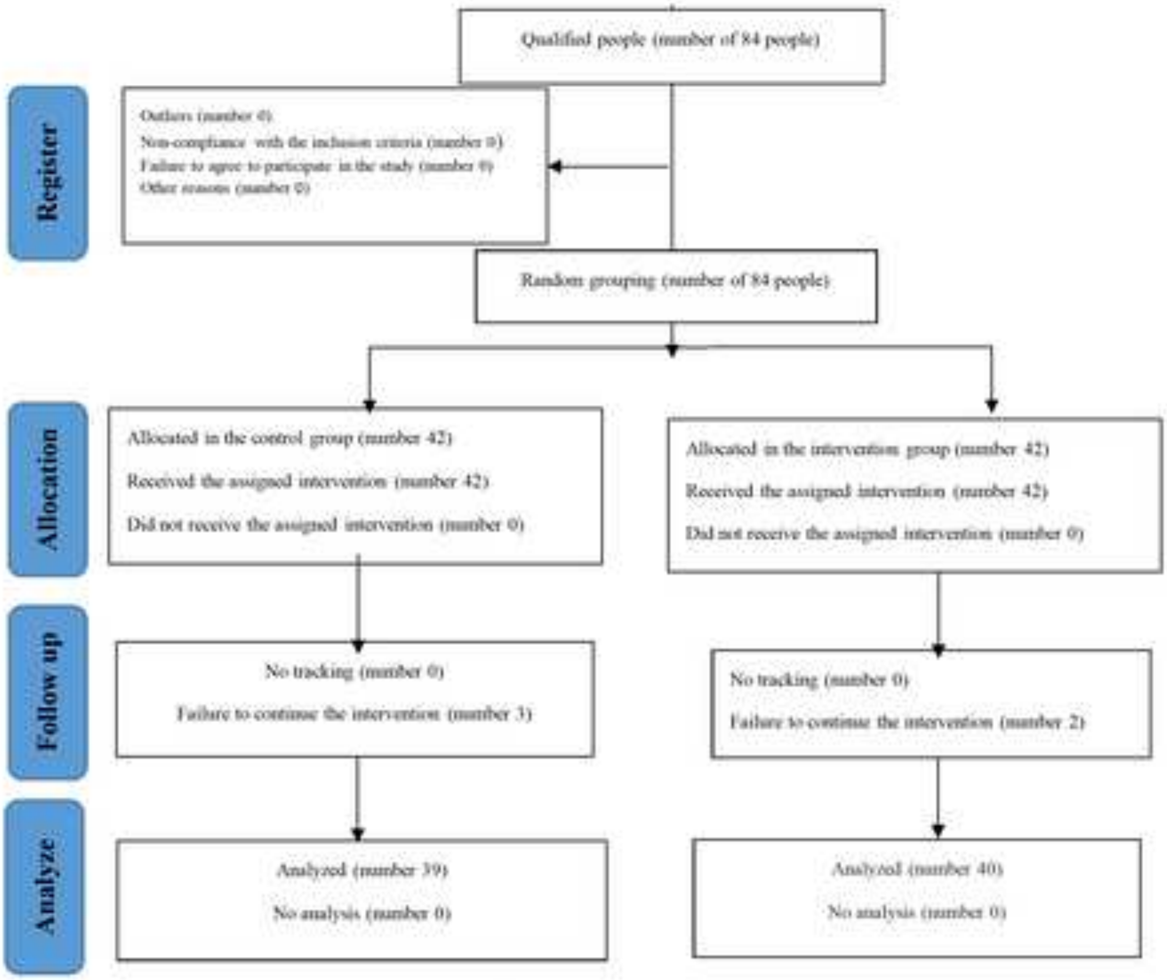

Figure 1: Consortium diagram

Click here to access/download
Supporting Information
CONSORT-2010-Checklist (1).doc

Click here to access/download
Supporting Information
persian original thesis.pdf

Click here to access/download
Supporting Information
PROTOCOL.docx

Click here to access/download
Other
other.pdf
