# Supplementary material for: Effect of intra-dialytic pedaling exercise on dialysis adequacy: A randomized controlled trial
Source: PLoS One. 2026 May 15;21(5):e0348063. doi: 10.1371/journal.pone.0348063 (PMC13178916; doi:10.1371/journal.pone.0348063)
Supplement: Attachment — Submitted filename: Summary of Suggestions & Comments.pdf [file pone.0348063.s007.pdf]

Dear Editor,

Thank you for the opportunity to review this manuscript. The study titled "Effect of Intra-Dialytic Pedaling Exercise on Dialysis Adequacy: A Randomized Controlled Trial" examined whether one month of intradialytic cycling sessions could impact dialysis adequacy parameters.

While the manuscript shows potential for publication in your journal, I have significant methodological and presentation concerns that must be addressed. Below I outline these issues with constructive suggestions to enhance the study's rigor and clarity.

General Comments

The acronym usage rules throughout the document appear inconsistent. If terms are consistently presented alongside their acronyms, the purpose of using acronyms becomes questionable. Similarly, some acronyms are introduced but then never used again, creating unnecessary variation.

A notable structural issue emerged during manuscript review: Substantive methodological and analytical information is consistently positioned at the end of sections. This organizational approach forces readers to retain unanswered questions unnecessarily. I recommend restructuring to present critical details in logical sequence based on their conceptual importance.

Was the type of vascular access not recorded for patients? Were all undergoing conventional hemodialysis rather than hemodiafiltration? Was the treatment uniformly administered three days per week? Since these were not mandatory inclusion/exclusion criteria, readers cannot anticipate these factors. Regarding vascular access, this variable should be included and considered based on its distribution - both in chi-square tests and as a potential study limitation.

Was the sample probabilistic or non-probabilistic? This crucial information must be included in the Methods section. There appears to be a substantial selection bias in patient recruitment that isn't properly documented - particularly in the flowchart which fails to show:

a) The total pool of potentially eligible patients
b) The exact exclusion cascade

I strongly recommend: Restructuring the flowchart to reflect all screening stages

Providing details on the number of physical activity sessions and average adherence would strengthen the methods. Additionally, clarifying if dropouts or missed hemodialysis sessions occurred—despite no adverse events—would enhance transparency.

All tables should include captions clearly stating the statistical tests used, ensuring consistency and reader accessibility. The CONSORT flowchart appears duplicated in the manuscript, and both versions suffer from poor resolution that compromises readability. This requires correction.

The opening of the Discussion section should be revised to first summarize the study's key findings. In its current form, it reads more like an introductory/contextual opening rather than a discussion of results.

The study's justification requires major revision. Neither the introduction convincingly establishes the research's importance, nor does the discussion adequately explain how the divergent methodology resolves existing limitations. These fundamental aspects need urgent attention. How does this study address this gap? The authors should reconsider and enhance the study's justification within its proper context.

The authors should avoid conflating the identification of a literature gap with automatic justification for their study's urgency. A gap's mere existence does not inherently validate the research's importance – the specific value and timeliness of addressing it must be explicitly demonstrated.

INTRODUCTION

Line 66

HD → This is the second time this term has appeared. I suggest that the acronym be introduced at the first occurrence, and once this rule is established, it should be consistently followed. More than once, 'hemodialysis' has reappeared without being presented as an acronym.

Line 71

Reference 12 → Seek a more recent reference regarding the epidemiology of the treatment itself—it should be easily found. In fact, some of the references already cited may contain this information.

Line 72

Once again, 'hemodialysis' appears without being abbreviated. Consistency with the established rule is required.

Lines 87 – 93

The paragraph spanning lines 87-93 may present comprehension challenges for readers with limited subject-matter expertise. I recommend both revising the content and reorganizing its structure for improved clarity.

Line 97

I propose including both the average weekly sedentary time imposed by the treatment and, immediately following this, the annualized measure. Presenting more tangible, daily-life metrics would significantly strengthen the argument's impact.

Line 101

While the preceding paragraph introduces physical inactivity as negatively impacting various aspects of hemodialysis patients' lives, it notably ceases to mention dialysis adequacy - despite this being the paper's primary focus as indicated in the title. Subsequently, when discussing conflicting results regarding intradialytic exercise, the text fails to specify that these contradictions relate almost solely to dialysis adequacy outcomes. Given that conflicting evidence exists for other aspects of intradialytic exercise as well, I strongly recommend clarifying at line 101 that the discussion refers exclusively to dialysis adequacy.

Lines 101 – 112

Beyond emphasizing physical activity's general importance, the introduction must specifically justify: (1) why cycling was selected as the intervention modality over other exercise types, and (2) why intradialytic cycling protocols deserve particular focus despite existing studies employing alternative modalities. This rationale requires substantial strengthening to properly contextualize your study's design choices.

Please clarify: (1) What was the intervention duration in these cited studies? (2) How does your protocol differ from existing approaches? (3) What novel contribution does your study provide compared to current literature? These justifications are critical for readers to contextualize your work's significance.

METHODS

Study Desgin

The CONSORT guidelines must be explicitly cited and followed in the main text - merely attaching them as supplementary material is insufficient.

Inclusion and exclusion criteria

Line 120

We must distinguish between cumulative hemodialysis duration (3 months) versus continuous current hemodialysis treatment (minimum 3 months) as inclusion criteria. I would recommend revising

Lines 125 – 133

The exclusion criteria section (line 125) should explicitly state upfront that these apply only to participants who first met all inclusion criteria. Currently this crucial context appears only inferentially in lines 129-130.

Sample size and sampling method

Line 151

Attention needed when revising: the current paragraph remains incomplete (line 151).

Implementation

Line 180-184

Given the methodological importance of the intervention, I strongly advise adding a distinct subsection detailing the physical activity protocol.

I strongly recommend the authors consult the 'Consensus on Exercise Reporting Template (CERT): Explanation and Elaboration Statement' to enhance their training protocol description. Critical methodological details are currently missing from the reported methods.

Article link: https://bjsm.bmj.com/content/50/23/1428

RESULTS

Lines 222 – 224

This is a very important point regarding the adverse effects that could have occurred during the intervention but did not. This information should be included in the Methods section, specifying how and when this monitoring was recorded.

Line 237

The acronym for blood urea nitrogen (BUN) should have been first included in the 'Data collection tools' subsection of the Methods.

Table 3

The Kt/V values from both time points could be presented, even without statistical comparison, to properly characterize the groups.

DISCUSSION

Lines 256 – 262

This section presents a somewhat vague discussion. Describing 'exercise by exercise' does not adequately characterize the specific modalities or types employed. It is problematic to compare simple pedaling with concurrent exercise (aerobic + resistance) without explicitly addressing this distinction. Furthermore, what was the intervention duration in these studies? Were they chronic or acute? In my assessment, there appears to be substantial inconsistency in this discussion.

Lines 298 – 299

Therefore, what justifies the complete absence of reported vascular access outcomes?

Lines 310 – 318

The discussion points and information presented in this paragraph should have been introduced earlier and systematically developed throughout the Discussion section.

CONCLUSION

Line 328

Did the authors perform any sensitivity analysis by matching groups for age? This potential confounding variable warrants examination given its known influence on [relevant outcome measures

REFERENCES

Lines 462 – 464

I was unable to locate this reference. Could the authors please provide the direct DOI or URL link to the cited source?
