# Supplementary material for: Effect of intra-dialytic pedaling exercise on dialysis adequacy: A randomized controlled trial
Source: PLoS One. 2026 May 15;21(5):e0348063. doi: 10.1371/journal.pone.0348063 (PMC13178916; doi:10.1371/journal.pone.0348063)
Supplement: Attachment — Submitted filename: Summary of Suggestions & Comments_R3.pdf [file pone.0348063.s009.pdf]

Dear Editor and Authors,

Thank you for the opportunity to review again this manuscript.

The study titled "Effect of Intra-Dialytic Pedaling Exercise on Dialysis Adequacy: A Randomized Controlled Trial" examined whether one month of intradialytic cycling sessions could impact dialysis adequacy parameters.

The manuscript has improved exponentially since the previous review round. The authors have been highly responsive and have successfully incorporated the majority of the suggestions and critiques from all reviewers. This diligent revision has unequivocally strengthened the work. The manuscript now demonstrates significantly greater technical precision and methodological clarity, enhancing its overall rigor and reproducibility. The authors are to be commended for their efforts.

Please find below my comments for this revised version of the manuscript. I have organized the feedback by priority level—Major and Minor—as was done in the previous review round.

The authors have undertaken substantial revisions, integrating a high volume of new text and data throughout the manuscript. As is natural with such significant changes, this new iteration has surfaced additional points requiring correction and refinement. All comments, regardless of priority, share the singular objective of further strengthening the manuscript's clarity, accuracy, and overall impact.

I commend the authors for their continued commitment to improving this work and trust they will find these final observations constructive.

Major Comments

- My primary concern regarding vascular access is not its functional status per se, but rather whether its type differed systematically between the randomized groups at baseline. Given that different access types can directly and indirectly impact various physical and psychosocial patient outcomes, this constitutes a potential source of bias. I consider this a significant methodological limitation. Therefore, I strongly recommend that the authors retrieve and report this baseline characteristic. This information should be obtainable from the hemodialysis centers' records or the researchers' own documentation. Presenting a comparison of vascular access type between study groups is essential to assess the randomization integrity and the interpretability of the findings.

- The primary objective of the study, which is prominently reflected in its title, is currently addressed only as the third topic within the Discussion section. This structural choice diminishes the prominence and analytical focus warranted by the main research aim. I strongly recommend restructuring the Discussion to address the primary objective first. The logical flow should follow the hierarchy of the study's aims: the central question should be discussed with the greatest depth and priority, followed by secondary and exploratory findings.
- The discussion treats the choice of exercise intensity with insufficient depth. While it is briefly mentioned as a potential hypothesis for the absence of significant results, this aspect warrants a far more substantial and critical exploration, comparable to the detailed treatment given to exercise volume.
- The criteria used to define "moderate intensity" require clarification and justification. The described parameter—an increase of 10% above baseline heart rate—is not consistent with standard exercise prescription guidelines for this intensity domain. It seems to me to be more of a light/low intensity.
- A fundamental methodological consideration must be addressed: the study's design places it closer to an acute rather than a chronic exercise intervention paradigm. This critical aspect is currently absent from the discussion. The absence of significant changes cannot be attributed solely to the parameters of volume and intensity. The insufficient duration of the intervention to induce chronic physiological adaptations is a primary, and likely predominant, explanatory factor.

Minor Comments

Introduction

Line 72

The issue with acronyms has recurred, this time concerning kidney replacement therapy (KRT). I strongly advise systematically reviewing this throughout all new sections of the manuscript. Please ensure that all acronyms are explicitly defined upon first use (e.g., "kidney replacement therapy (KRT)"). Implementing this standard practice preemptively will enhance manuscript clarity and eliminate the need for repetitive requests on this matter during the review process.

Lines 111 – 112

The justification for selecting cycling as the primary intervention modality need not be explicitly stated to the reader. There may have been some confusion stemming from my previous comments. The intention is for the presented data and methodological rationale to inherently justify the choice of exercise modality. I believe this is primarily a matter of rephrasing the introductory clause of this paragraph to allow the subsequent information to stand as its own justification.

As a suggested rephrasing for exemplification:

> Cycling represents a practical and safe intervention modality that is well-suited to the physical constraints commonly experienced by patients undergoing hemodialysis.

Lines 119 – 122

Exercise volume is reported only for the intervention group that showed significant improvement, which may be perceived as somewhat selective reporting. To ensure completeness and transparency, I recommend standardizing this information.

Results

Table 1

The asterisk (*) is used within the table to denote specific statistical comparisons (e.g., $p < 0.05$). It is therefore inappropriate and potentially confusing to reuse the same symbol for the general table footnote. Please assign a distinct symbol (e.g., †, ‡, §) for the general legend or present the footnote using a different format (e.g., a separate, non-symbol key or a descriptive note below the table title).

Line 309

I suggest removing the entire text on line

Discussion

Lines 333 – 335

Avoid using value-laden language when describing outcomes that showed no statistically significant difference. If the statistical analysis confirms the absence of a significant difference, the results should be presented neutrally. Describing non-significant data with terms that imply a trend or a qualitative distinction (e.g., "slightly higher," "appeared better," "marginally lower") can be misleading and is not supported by the statistical evidence presented.

Lines 371 – 373

I would like to respectfully reinforce the possibility that the inefficacy of the protocol to generate significant changes may be attributed as much, if not more, to the exercise intensity prescribed rather than solely to the total exercise volume.

Line 379

When citing other studies for comparative purposes, it is essential to provide the reader with sufficient information to understand the similarities and key differences between your protocol and those referenced. Throughout this section, a critical gap is evident: the discussion lacks a concise comparative analysis of the very exercise parameters (volume and intensity) that are central to your hypothesis.

Lines 386 – 388

The discussion of vascular access as a limitation should be substantially shortened and made more factual. Rather than a speculative narrative, simply report the relevant baseline characteristic. For example: state the number and percentage of patients using an arteriovenous fistula versus a central venous catheter in each study group.

Paragraph that starts in line 399

How similar? Intensity? Volume? There are dozens of other studies with prescribed aerobic exercise that report Kt/V, although they may not be concerned with focusing on that, they need to characterize very well to what extent an intervention should be similar to yours to be considered in the discussion.

Line 478

This section assumes and proceeds with the classification of the exercise modality as low intensity. This represents a direct contradiction to its description elsewhere in the manuscript (e.g., as "moderate intensity"). This internal inconsistency must be resolved. Furthermore, the definitive classification of exercise intensity must be explicitly and unambiguously stated within the Methods section, specifically in the description of the exercise protocol.

Flowchart

I recommend modifying the participant flowchart to clearly separate individuals who were ineligible (did not meet the inclusion/exclusion criteria) from those who were eligible but declined to participate (refused). This distinction is methodologically important.

Providing this granularity enhances the transparency of your recruitment process and the reproducibility of your study. Furthermore, it increases the potential utility and citation frequency of your work. Future research—for instance, studies analyzing acceptance rates or

barriers to participation in interventional trials among the hemodialysis population—would rely on such clearly disaggregated data. A mere detail in presentation can determine whether a subsequent systematic review or meta-analysis can (or cannot) incorporate your findings.
