# Supplementary material for: Effect of intra-dialytic pedaling exercise on dialysis adequacy: A randomized controlled trial
Source: PLoS One. 2026 May 15;21(5):e0348063. doi: 10.1371/journal.pone.0348063 (PMC13178916; doi:10.1371/journal.pone.0348063)
Supplement: Attachment — Submitted filename: PONE-D-25-17351_R1.pdf [file pone.0348063.s010.pdf]

PLOS ONE

Effect of Intra-Dialytic Pedaling Exercise on Dialysis Adequacy: A Randomized Controlled Trial

--Manuscript Draft--

**Manuscript Number:**	PONE-D-25-17351R1
**Article Type:**	Clinical Trial
**Full Title:**	Effect of Intra-Dialytic Pedaling Exercise on Dialysis Adequacy: A Randomized Controlled Trial
**Short Title:**	Short Title: Intra-Dialytic Pedaling Exercise and Dialysis Adequacy
**Corresponding Author:**	Shahnaz Pouladi, ph.D<br>Bushehr University of Medical Sciences<br>Bushehr, Bushehr IRAN, ISLAMIC REPUBLIC OF
**Keywords:**	physical activity, dialysis adequacy, chronic kidney disease, hemodialysis
**Abstract:**	Background: In patients with chronic kidney disease undergoing hemodialysis, physical activity and rehabilitation are crucial to prevent the reduction of muscle strength and functional capacity. This study aimed to determine the effectiveness of physical activity during hemodialysis on dialysis adequacy in hemodialysis patients.<br>Methods: This randomized controlled trial (RCT) investigated the impact of pedaling exercise on dialysis effectiveness in 84 hemodialysis patients at Bushehr hospitals. Participants were randomly assigned to either an experimental group (n=42) that performed 30 minutes of pedaling exercise during their 4-hour dialysis sessions or a control group (n=42) that received routine hemodialysis. Dialysis adequacy was assessed by comparing pre-and post-dialysis blood samples taken from the arterial line. A standardized protocol was used for collecting blood samples, and patient weight was measured using a calibrated digital scale. Data analysis was performed using SPSS.ver24 software.<br>Results: The experimental and control groups were similar in demographic characteristics, except for age (X2= -3.84, p=0.001) and education levels (X2= 10.100, p=0.006). While there was no significant difference in weight before and after the intervention between the groups (t=0.223, p=0.82 before; t=0.203, p=0.84 after), both groups showed a substantial weight reduction overall (p<0.0001). There were no statistically significant differences in weight change (t=0.80, p=0.25), blood urea nitrogen (BUN) (t=0.13, p=1.52), or Kt/V (t=1.62, p=0.11) between the experimental and control groups.<br>Conclusion: This study found that incorporating pedaling exercise during hemodialysis did not significantly improve dialysis effectiveness, as measured by weight change, BUN levels, or Kt/V. While both groups showed weight loss, there were no statistically significant differences between them. However, the study had limitations due to its small sample size and the specific type of exercise used. Further research with larger cohorts and a wider variety of physical activities is needed to determine if physical activity during hemodialysis can benefit dialysis adequacy and overall patient outcomes.<br>Trial registration: IRCT code number 20150529022466N15 and trial Code of Ethics IR.BPUMS.REC.1398.130
**Order of Authors:**	Mahmoud Mohamadizadeh, M.Sc. student
	Sharif Sharifi, lecturer
	Niloufar Motamed, Associate professor
	Mohammad Amin Shadman, lecturer
	Shahnaz Pouladi, ph.D
**Response to Reviewers:**	Dear Reviewers,<br>We would like to express our sincere gratitude for your time, expertise, and thoughtful comments on our manuscript. Your detailed feedback has been invaluable in improving the quality and clarity of our work.<br>We have carefully considered each comment line by line, and our responses are

Powered by Editorial Manager® and ProduXion Manager® from Aries Systems Corporation

	provided in the attached “Response to Reviewers” document. All suggested revisions have been addressed with great attention.<br>Thank you again for your generous contribution to the review process.<br>Sincerely,<br>Shahnaz Pouladi<br>Corresponding Author
Additional Information:	
**Question**	**Response**
**Financial Disclosure**<br><br>Enter a financial disclosure statement that describes the sources of funding for the work included in this submission. Review the submission guidelines for detailed requirements. View published research articles from *PLOS ONE* for specific examples.<br><br>This statement is required for submission and **will appear in the published article** if the submission is accepted. Please make sure it is accurate.<br><br>**Funded studies**<br>Enter a statement with the following details:<br>• Initials of the authors who received each award<br>• Grant numbers awarded to each author<br>• The full name of each funder<br>• URL of each funder website<br>• Did the sponsors or funders play any role in the study design, data collection and analysis, decision to publish, or preparation of the manuscript?<br><br>Did you receive funding for this work?	The author(s) received no specific funding for this work.
**Competing Interests**<br><br>Use the instructions below to enter a competing interest statement for this submission. On behalf of all authors, disclose any competing interests that could be perceived to bias this work—acknowledging all financial support and any other relevant financial or non-financial competing interests.	The authors have declared that no competing interests exist.

This statement is required for submission and **will appear in the published article** if the submission is accepted. Please make sure it is accurate and that any funding sources listed in your Funding Information later in the submission form are also declared in your Financial Disclosure statement.<br><br>View published research articles from *PLOS ONE* for specific examples.<br><br>**NO authors have competing interests**<br><br>Enter: *The authors have declared that no competing interests exist.*<br><br>**Authors with competing interests**<br><br>Enter competing interest details beginning with this statement:<br><br>*I have read the journal's policy and the authors of this manuscript have the following competing interests: [insert competing interests here]*<br><br>* typeset	
**Ethics Statement**<br><br>Enter an ethics statement for this submission. This statement is required if the study involved:<br><br>• Human participants<br>• Human specimens or tissue<br>• Vertebrate animals or cephalopods<br>• Vertebrate embryos or tissues<br>• Field research<br><br>Write "N/A" if the submission does not require an ethics statement.<br><br>General guidance is provided below. Consult the submission guidelines for detailed instructions. **Make sure that all information entered here is included in the Methods section of the manuscript.**	The study protocol was approved by the Ethics Committee of Bushehr University of Medical Sciences (ethics code number IR.BPUMS.REC.1398.130). Written informed consent was obtained from all participants prior to their enrollment in the study. Participants were informed of their right to withdraw from the study at any time without any negative consequences. The confidentiality of their data was strictly maintained throughout the study."

Powered by Editorial Manager® and ProduXion Manager® from Aries Systems Corporation

**Format for specific study types**<br><br>**Human Subject Research (involving human participants and/or tissue)**<br>• Give the name of the institutional review board or ethics committee that approved the study<br>• Include the approval number and/or a statement indicating approval of this research<br>• Indicate the form of consent obtained (written/oral) or the reason that consent was not obtained (e.g. the data were analyzed anonymously)<br><br>**Animal Research (involving vertebrate animals, embryos or tissues)**<br>• Provide the name of the Institutional Animal Care and Use Committee (IACUC) or other relevant ethics board that reviewed the study protocol, and indicate whether they approved this research or granted a formal waiver of ethical approval<br>• Include an approval number if one was obtained<br>• If the study involved *non-human primates*, add *additional details* about animal welfare and steps taken to ameliorate suffering<br>• If anesthesia, euthanasia, or any kind of animal sacrifice is part of the study, include briefly which substances and/or methods were applied<br><br>**Field Research**<br><br>Include the following details if this study involves the collection of plant, animal, or other materials from a natural setting:<br>• Field permit number<br>• Name of the institution or relevant body that granted permission	
**Data Availability**<br><br>Authors are required to make all data underlying the findings described fully available, without restriction, and from the time of publication. PLOS allows rare exceptions to address legal and ethical concerns. See the PLOS Data Policy and FAQ for detailed information.	Yes - all data are fully available without restriction

A Data Availability Statement describing where the data can be found is required at submission. Your answers to this question constitute the Data Availability Statement and **will be published in the article**, if accepted.<br><br>**Important:** Stating 'data available on request from the author' is not sufficient. If your data are only available upon request, select 'No' for the first question and explain your exceptional situation in the text box.<br><br>Do the authors confirm that all data underlying the findings described in their manuscript are fully available without restriction?	
**Describe where the data may be found in full sentences. If you are copying our sample text, replace any instances of XXX with the appropriate details.**<br><br>• If the data are **held or will be held in a public repository**, include URLs, accession numbers or DOIs. If this information will only be available after acceptance, indicate this by ticking the box below. For example: *All XXX files are available from the XXX database (accession number(s) XXX, XXX.*).<br>• If the data are all contained **within the manuscript and/or Supporting Information files**, enter the following: *All relevant data are within the manuscript and its Supporting Information files.*<br>• If neither of these applies but you are able to provide **details of access elsewhere**, with or without limitations, please do so. For example:<br><br>*Data cannot be shared publicly because of [XXX]. Data are available from the XXX Institutional Data Access / Ethics Committee (contact via XXX) for researchers who meet the criteria for access to confidential data.*<br><br>*The data underlying the results presented in the study are available*	The minimal data set underlying the findings of this study—including the raw values used to generate tables, figures, and statistical analyses—is provided in Supporting Information file S1_Data (SPSS format). These data are fully anonymized and sufficient to replicate the study's results.

from (include the name of the third party and contact information or URL).<br>• This text is appropriate if the data are owned by a third party and authors do not have permission to share the data.<br><br>* typeset	
Additional data availability information:	

Manuscript Click here to access/download;Manuscript;manuscript.docx

Effect of Intra-Dialytic Pedaling Exercise on Dialysis Adequacy: A Randomized Controlled Trial

Mahmoud Mohamadizadeh (MSc)[1], Sharif Sharifi (MSc)[2], Niloufar Motamed (Ph.D.)[3], Mohammad Amin Shadman (MSc)[4], Shahnaz Pouladi (Ph.D.) *[5]

1. Master's student in medical-surgical nursing, student research committee, Bushehr University of Medical Sciences, Bushehr, Islamic Republic of Iran. Mahmoud.2580@yahoo.com
2. MSc in medical-surgical nursing, Nursing and Midwifery Faculty of Bushehr University of Medical Sciences, Bushehr, Islamic Republic of Iran.
3. PhD in community medicine, Department of Community Medicine, Medicine faculty of Bushehr University of Medical Sciences, Bushehr, Islamic Republic of Iran.
4. MSc in medical-surgical nursing, Nursing and Midwifery Faculty of Bushehr University of Medical Sciences, Bushehr, Islamic Republic of Iran.
5. PhD in Nursing Education, Associate Professor, Nursing and Midwifery Faculty of Bushehr University of Medical Sciences, Bushehr, Islamic Republic of Iran. pouladi2008@gmail.com

Abstract

Background: In patients with chronic kidney disease undergoing hemodialysis, physical activity and rehabilitation are crucial for preventing declines in muscle strength and functional capacity. This study aimed to assess the impact of physical activity during hemodialysis on dialysis adequacy in these patients.

Methods: This randomized controlled trial (RCT) investigated the impact of pedaling exercise on dialysis effectiveness in 84 hemodialysis patients at hospitals in Bushehr. Participants were randomly assigned to either an experimental group (n=42) that performed 30 minutes of pedaling exercise during their 4-hour dialysis sessions or a control group (n=42) that received routine hemodialysis. Dialysis adequacy was assessed by comparing pre- and post-dialysis blood samples obtained from the arterial line. A standardized protocol was used to collect blood samples, and patient weight was measured with a calibrated digital scale. Data analysis was performed using SPSS version 24 software.

Results: The experimental and control groups were similar in demographic characteristics, except for age (X2 = -3.84, p = 0.001) and education levels (X2 = 10.100, p = 0.006). While there was no significant difference in weight between the groups before and after the intervention (t=0.223, p=0.82 before; t=0.203, p=0.84 after), both groups showed a substantial weight reduction overall (p<0.0001). There were no statistically significant differences in weight change (t=0.80, p=0.25), BUN (t=0.13, p=1.52), or Kt/V (t=1.62, p=0.11) between the experimental and control groups.

Conclusion: This study found that incorporating pedaling exercise during hemodialysis did not significantly improve dialysis effectiveness, as measured by weight change, BUN levels, or Kt/V.

While both groups showed weight loss, there were no statistically significant differences between them. However, the study had limitations due to its small sample size and the specific type of exercise used. Further research with larger cohorts and a broader range of physical activities is needed to determine whether physical activity during hemodialysis can improve dialysis adequacy and overall patient outcomes.

Trial registration: IRCT code number 20150529022466N15 and trial Code of Ethics IR.BPUMS.REC.1398.130

Keywords: physical activity, dialysis adequacy, chronic kidney disease, hemodialysis

Introduction

Chronic kidney disease (CKD) is a significant global health problem, leading to both short- and long-term complications and reducing patients' quality of life (1,2). Recent studies indicate that CKD prevalence varies by region (3,4), with estimates of 11.4% in Iran (5), 9% in Mexico (6), and 13.1% among adults in the United States (7). Several treatment options are available for managing CKD, including kidney transplantation, peritoneal dialysis, and hemodialysis (8). Nearly 4 million people worldwide rely on kidney replacement therapy (KRT), with hemodialysis being the most common, accounting for about 69% of all KRT and 89% of dialysis cases (9). CKD is projected to become the fifth leading cause of death globally by 2040 (10). Given population growth, an annual increase of 5–6% in CKD prevalence poses a challenge for all countries worldwide. In Iran, this growth rate surpasses the global average (11).

Hemodialysis remains the most widely utilized modality for kidney replacement therapy worldwide, accounting for the majority of dialysis cases and imposing substantial financial and operational burdens on healthcare systems. Recent global epidemiological data confirm this trend, highlighting the increasing reliance on hemodialysis across diverse regions and its implications for public health planning (12).

Different hemodialysis programs exist, including home hemodialysis (3–6 times per week), in-center hemodialysis, short daily hemodialysis, and long nocturnal hemodialysis (13). Although hemodialysis prevents death due to uremia, the survival rate of patients with CKD is significantly lower than that of the general population (14-16). Several factors influence the survival rate of these patients, including the cause of renal failure, the treatment method, the presence of comorbidities such as cardiovascular disease, diabetes, and hypertension, and the adequacy of dialysis (17,18).

The long-term prognosis of patients undergoing dialysis depends heavily on the efficacy of their treatment, commonly referred to as dialysis adequacy. Defined by the National Kidney Foundation Kidney Disease Outcomes Quality Initiative (NKF KDOQI) as sufficient urea clearance, dialysis adequacy serves as a key indicator of treatment success and patient survival (19,20). Inadequate dialysis adequacy has been associated with a range of adverse outcomes, including sexual dysfunction, cardiovascular disease, pruritus, prolonged hospitalization, and increased healthcare costs (21,22). Conversely, improved dialysis adequacy enhances patients' quality of life, physical functioning, and spiritual well-being (23). Given its central role in clinical outcomes, dialysis adequacy is not only a benchmark for evaluating dialysis protocols but also a critical endpoint in studies assessing adjunctive interventions such as intradialytic exercise. Understanding how physical activity during dialysis may influence adequacy is therefore essential to optimizing care strategies for this population.

The effectiveness of a dialysis session is commonly assessed using two key indicators: Kt/V and the urea reduction ratio (URR) (24,25). Kt/V quantifies the efficiency of dialysis in removing urea from the body. It is calculated by dividing the product of dialyzer clearance (K) and dialysis time (t) by the volume of distribution of urea, which approximates total body water (V) (26). According to the urea kinetic model, both Kt/V and URR are standard measures of dialysis adequacy. Clinically, a minimum Kt/V value of 1.2 and a target URR of approximately 65% are recommended to ensure sufficient urea clearance during treatment (20).

Patients with CKD undergoing hemodialysis often experience functional decline, reduced quality of life, and psychological disorders such as depression and anxiety related to their treatment (27-30). Hemodialysis is typically administered in a supine position, requiring patients to remain physically inactive for approximately 12 hours per week—equivalent to nearly 800 hours annually.

This prolonged sedentary time contributes to complications such as reduced physical endurance, muscle atrophy, and muscle weakness (31). Incorporating regular exercise during dialysis sessions has been shown to improve physical performance and mitigate muscle deterioration in hemodialysis patients (32,33).

Cycling was selected as the intervention modality due to its practicality, safety, and compatibility with the physical limitations of hemodialysis patients. Stationary pedaling can be performed in a seated or supine position, making it feasible during dialysis sessions without disrupting treatment protocols. Recent evidence suggests that intradialytic cycling is well tolerated and can be easily supervised by clinical staff, enhancing adherence and minimizing risk (34). Moreover, intradialytic exercise offers a unique opportunity to utilize otherwise sedentary treatment time for therapeutic benefit. Compared to pre- or post-dialysis exercise, intradialytic protocols allow for continuous monitoring, reduce scheduling barriers, and may improve patient engagement (35,36).

Previous studies on physical activity during dialysis have yielded conflicting results. For instance, Kirkman et al. (2019) (37) and Riahi et al. (2012) (38) reported that pedaling exercises during dialysis did not significantly improve dialysis adequacy, while Rohmah et al. (2015) (39) found that 30 minutes of cycling enhanced physical fitness and biochemical markers. These inconsistencies highlight the need for further investigation using context-sensitive and methodologically robust designs. Bushehr, located in southern Iran, faces unique environmental challenges; its hot, humid climate limits outdoor physical activity, particularly for patients with chronic conditions (40). Moreover, the province has a disproportionately high number of hemodialysis patients per capita. Given the central role of dialysis nurses in delivering integrated care and patient education, their involvement presents a valuable opportunity to implement structured intradialytic exercise programs (34,35). Therefore, this study was conducted to evaluate

the effectiveness of supervised physical activity during hemodialysis on dialysis adequacy in patients treated in Bushehr's clinical centers, addressing both regional constraints and methodological gaps in prior research.

Methods

Study Design

This study was a randomized controlled clinical trial (RCT; IRCT20150529022466N15) conducted between January 10 and March 30, 2020, in the dialysis departments of Bushehr hospitals in southern Iran. The trial was designed and reported in accordance with the CONSORT 2010 guidelines to ensure transparency and methodological rigor (41). The authors confirm that all ongoing and related trials for this intervention are registered.

Inclusion and Exclusion Criteria

Inclusion criteria were: age between 18 and 65 years, undergoing continuous hemodialysis treatment for at least 3 months without interruption, ability to walk independently for at least 2 minutes, no history of unstable angina, dyspnea, myocardial infarction within the past month, or congestive heart failure; no use of neuropsychiatric medications; tolerance of 4-hour dialysis sessions; and absence of hypokalemia ($K < 3.5$ mEq/L). These criteria were defined based on clinical guidelines and prior trials in hemodialysis populations (42).

Exclusion criteria were applied only to individuals who initially met all inclusion criteria. These included: cardiovascular complications such as unstable angina, systolic blood pressure ≥160 mmHg or diastolic ≥120 mmHg, hypotension defined as systolic BP <90 mmHg or a drop >30 mmHg during dialysis, neurological, musculoskeletal, or vascular disorders (confirmed by

physician), missing four or more dialysis sessions during the study period, kidney transplantation or death, vascular access problems, and unwillingness to continue participation (42).

Sample Size and Sampling Method

Using G*Power 3.1.9.2, with a 95% confidence level and 80% power, the required sample size was 76 participants for an expected effect size (d = 0.65). The mean and SD of dialysis adequacy (Kt/V) in the control group were 1.33 and 0.18, respectively; the expected mean in the intervention group was 1.45. To account for a 10% dropout rate, the sample size was increased to 84 (42 per group) using the formula:

$n' = n / (1 - q)$

A non-probabilistic convenience sampling method was used to recruit patients from hospitals in Bushehr. Although random allocation was applied, initial recruitment was based on availability and willingness, which may introduce selection bias (43).

Randomization and Blinding

Participants were randomly assigned to intervention or control groups using a computer-generated random number table by an independent researcher. Allocation was concealed using sequentially numbered, sealed opaque envelopes. The study was single-blind: participants were unaware of their group assignment. Due to the nature of the intervention, blinding of medical staff was not feasible. To minimize contamination, groups were treated in separate centers and at different times (44).

Treatment Uniformity and Vascular Access

All participants underwent conventional hemodialysis (not hemodiafiltration), three times per week for four hours per session. The dialysis modality and frequency were consistent across both groups. Although the type of vascular access (e.g., fistula, graft, or catheter) was not systematically recorded as a demographic variable, all patients had functional access suitable for standard hemodialysis. The potential influence of vascular access type on dialysis adequacy is acknowledged as a study limitation (45).

Exercise Protocol and Adherence (CERT-Based Description)

The intradialytic exercise intervention was designed and reported in accordance with the Consensus on Exercise Reporting Template (CERT) to ensure methodological transparency and replicability (46). Participants in the intervention group performed stationary cycling during dialysis using a portable ATMED (Taiwan) bike weighing 3.5 kg, positioned at the foot of the dialysis bed. All exercises were conducted in the supine position during dialysis.

Each session consisted of a 5-minute warm-up, two 15-minute pedaling intervals separated by a 15-minute rest, and a cool-down phase. The intervention lasted 4 weeks, with three sessions per week, for a total of 12. All participants in the intervention group completed the full set of exercise sessions without interruption. Kt/V values were recorded during the 12-session intervention period, starting from the first dialysis session. As the exercise protocol commenced immediately, no pre-intervention Kt/V measurements were collected. This design reflects the pragmatic integration of the intervention into routine clinical practice (47).

Exercise intensity was individualized and adjusted to achieve a 10% increase in heart rate compared to baseline. The safe pulse range during exercise was maintained at 50-125 beats per minute. Pulse and blood pressure were measured before and after each session, and pulse was

continuously monitored throughout. If the target heart rate was not achieved, patients were instructed to pedal faster. (48).

All sessions were supervised by a trained nurse familiar with the protocol. Participants received verbal encouragement and feedback to maintain motivation and adherence. The exercise protocol was standardized across all participants, and no modifications were required during the study. The equipment and setting remained consistent throughout. The full protocol is archived in the thesis repository of Bushehr University of Medical Sciences and is available upon request (49).

Participants were monitored for any abnormal signs or adverse events during each exercise session. The supervising nurse documented clinical observations throughout the intervention period as part of the safety protocol.

Data Collection Tools

Data were collected using a demographic form and a dialysis adequacy form. Variables included sex, age, education, marital status, weight (pre-/post dialysis), and dialysis adequacy indices. Kt/V was calculated using:

$$Kt/V = -\ln(R - 0.008 \times t) + (4 - 3.5 \times R) \times UF / W$$

To calculate the Urea Reduction Ratio (URR), blood urea nitrogen (BUN) levels were measured before and after dialysis.

URR = (BUN pre − BUN post) / BUN pre

Blood samples were collected pre- and post-dialysis using standard protocols. Post-dialysis samples were taken after reducing dialysate flow for 3 minutes and pump speed to <100 cc/min

for the final 5 minutes. Samples were coded and analyzed using a DIRUI 1200 machine. Weight was measured using a calibrated Seca digital scale (42).

Implementation

After the study protocol was approved by the Ethics Committee of Bushehr University of Medical Sciences (Code of Ethics IR.BPUMS.REC.1398.130), the researcher obtained an official letter of introduction and visited the dialysis departments of Bushehr Teaching Hospitals. Participant recruitment and intervention were conducted between January 10 and March 30, 2020.

After coordinating with the clinical staff, the researcher introduced the study and its objectives to eligible patients, obtained written informed consent, and initiated sampling. Participants were assured that their data would remain confidential and that they could withdraw from the study at any time without any negative consequences.

A total of 84 patients undergoing hemodialysis were recruited and randomly assigned to either the intervention or control group. All patients received conventional hemodialysis three times per week for four hours per session (50). The control group received standard care without any additional physical activity, remaining in the supine position throughout dialysis. The intervention group participated in intradialytic cycling using a portable ATMED (Taiwan) stationary bike weighing 3.5 kg, positioned at the foot of the dialysis bed.

Each exercise session consisted of two 15-minute pedaling intervals, separated by a 15-minute rest, preceded by a 5-minute warm-up and followed by a cool-down phase. The pedaling intensity was adjusted to achieve a 10% increase in heart rate from baseline. Blood pressure and pulse were measured before and after each session, and pulse was continuously monitored during exercise. If

the target heart rate was not achieved, patients were instructed to pedal faster. The safe pulse range during exercise was maintained at 50-125 beats per minute.

The intervention program lasted four weeks, with three sessions per week, totaling 12 sessions (51,52). All participants in the intervention group completed the full set of exercise sessions. Dialysis adequacy was measured before and after the intervention period using Kt/V and URR indices.

During the study, two participants from the intervention group and three from the control group withdrew due to fatigue or unwillingness to continue. These cases were excluded from the final analysis. No post-intervention follow-up period was included in this study. All outcome measurements, including dialysis adequacy (Kt/V), were collected immediately after the final session of the exercise protocol.

Ultimately, data from 40 participants in the intervention group and 39 in the control group were analyzed. The flow of participants through each stage of the study—including recruitment, randomization, follow-up, and analysis—is presented in the CONSORT flow diagram (Figure 1), providing a visual summary of trial conduct.

Figure 1: CONSORT FLOW DIAGRAM

The entire trial protocol is archived in the library of Bushehr University of Medical Sciences and documented in the master's thesis of nursing student Mahmoud Mohammadizadeh.

Ethical Considerations

This study was part of a master's thesis in medical-surgical nursing at Bushehr University of Medical Sciences and registered under IRCT20150529022466N15. All participants received complete study information and signed informed consent. Confidentiality was maintained, and all procedures followed the Declaration of Helsinki and relevant ethical guidelines.

Data Analysis

Following data collection, statistical analysis was conducted using SPSS version 24 (IBM Corp., Armonk, NY, USA). Descriptive statistics, including means, standard deviations, and frequencies, were calculated to summarize the data. Group comparisons of demographic variables were performed using chi-squared tests. Paired-samples t-tests were used to assess within-group changes in dialysis adequacy (Kt/V) before and after the intervention, and independent-samples t-tests were used to compare the intervention and control groups.

Normality of continuous variables was assessed using the Shapiro-Wilk test and by evaluating the ratio of skewness to its standard error. A p-value of less than 0.05 was considered statistically significant.

Effect sizes (Cohen's d) and 95% confidence intervals were calculated for key outcomes to assess the magnitude and clinical relevance of the intervention. All analyses were performed using complete-case data, excluding participants who withdrew from the study.

Due to sample size limitations, no covariate-adjusted analysis was performed. All group comparisons were conducted using unadjusted statistical tests.

Results

The present study aimed to evaluate the effect of a structured physical activity intervention on dialysis adequacy and selected biochemical and physiological parameters in patients undergoing hemodialysis. A total of 79 patients were enrolled, with 40 in the intervention group and 39 in the control group. All patients completed the study protocol and were included in the final analysis. No adverse events or clinical complications related to the exercise intervention were observed or reported during the study period. Five participants (two in the intervention group and three in the control group) withdrew from the study due to a general unwillingness to continue.

Demographic characteristics of the participants are presented in Table 1. The majority were male (n = 51, 64.6%) and married (n = 72, 91.1%). Regarding employment status, 33 patients (41.3%) were employed, and 50 (63.3%) had an education level below a diploma. A statistically significant difference in mean age was observed between the intervention and control groups (p = 0.001), with the control group being older. No significant differences were found between the groups in terms of marital status (p = 0.11) or employment status (p = 0.29). However, an ~~important~~ difference was noted in education level (p = 0.006), with a higher proportion of patients in the control group having lower educational attainment.

Table1. Comparison of Demographic Variables Between the Intervention and Control Groups in Hospitals of Bushehr, Iran (2020)

Variable	Category	Intervention Group N (%)	Control Group N (%)	Total N (%)	X^2	p-value	Effect Size (Cohen's d) and 95% CI
Gender	Male	29 (72.5%)	22 (56.4%)	51 (64.6%)	2.23	0.13	
	Female	11 (27.5%)	17 (43.6%)	28 (35.4%)			
Marital Status	Single	6 (15%)	1 (2.6%)	7 (8.9%)	3.78*	0.11	
	Married	34 (85%)	38 (97.4%)	72 (91.1%)			

Variable	Category	Intervention Group N (%)	Control Group N (%)	Total N (%)	X²	p-value	Effect Size (Cohen’s d) and 95% CI
Employment Status	Unemployed	15 (38.5%)	19 (47.5%)	34 (42.5%)	2.42	0.29	
	Employed	16 (40%)	17 (42.5%)	33 (41.3%)			
	Retired	9 (22.5%)	4 (10%)	13 (16.3%)			
Education Level	Below Diploma	19 (47.5%)	31 (79.5%)	50 (63.3%)	10.10	0.006	
	Diploma	10 (25%)	6 (15.4%)	16 (20.3%)			
	University Degree	11 (27.5%)	2 (5.1%)	13 (16.5%)			
Age (years)**	—	50.25 ± 10.71	58.64 ± 8.55	—	−3.84	0.001	d = −0.05, CI [−4.5, 3.3]

*Fisher’s exact test

** Mean ± Standard Deviation

*Categorical variables were analyzed using the Chi-square test and Fisher’s exact test. Continuous variable (age) was analyzed using an independent samples t-test. Effect sizes are reported as Cohen’s d with 95% confidence intervals.

Table 2 summarizes the comparison of blood urea, body weight, and BUN before and after the intervention. No significant difference in blood urea levels was observed between the groups ($p = 0.13$). Both groups showed a statistically significant reduction in body weight from pre- to post-intervention ($p < 0.0001$), although the between-group differences in pre-intervention weight ($p = 0.82$) and post-intervention weight ($p = 0.84$) were not significant. Similarly, BUN levels decreased significantly within both groups ($p = 0.001$), but no significant differences were found between the intervention and control groups at either time point ($p = 0.17$ and $p = 0.74$, respectively) (Figure. 1).

Figure 2. Comparison of Mean Difference in Blood Urea (BUN) Levels Before and After Intervention in Intervention and Control Groups

Table 2. Comparison of Blood Urea, Body Weight, and BUN Before and After the Intervention Between the Intervention and Control Groups in Patients Hospitalized in Bushehr, Iran (2020)

Variable	Time Point	Intervention Group (Mean ± SD)	Control Group (Mean ± SD)	t	p-value	Effect Size (Cohen's d) and 95% CI
Blood Urea Level	—	0.616 ± 0.049	0.601 ± 0.040	1.52	0.13	d = 0.33, CI [−0.005, 0.035]
Body Weight	Before	72.96 ± 13.85	73.61 ± 11.93	−0.223	0.82	
	After	70.99 ± 13.95	71.59 ± 11.85	−0.203	0.84	
	t	0.11	13.80			d = −0.05, CI [−4.5, 3.3]
	p-value	0.0001	0.0001			
BUN	Before	58.66 ± 9.65	55.73 ± 8.92	1.40	0.17	-
	After	22.53 ± 4.69	22.18 ± 4.51	0.34	0.74	d = .08, CI [−1.3, 2.0]
	t	34.08	37.49			
	p-value	0.001	0.001			

Independent samples t-tests were used to compare blood urea and BUN levels between groups at each time point. Paired-samples t-tests were used to assess within-group changes in body weight before and after the intervention. Effect sizes were calculated using Cohen's d with 95% confidence intervals.

Table 3 presents the mean changes in blood urea, body weight, and BUN between the two groups. None of these changes reached statistical significance (p = 0.13, 0.80, and 0.07, respectively), ~~although the reduction in BUN approached the threshold for significance.~~

Table 3. Comparison of Mean Changes in Blood Urea, Body Weight, and BUN Between the Intervention and Control Groups in Patients Hospitalized in Bushehr, Iran (2020)

Variable	Intervention Group (Mean ± SD)	Control Group (Mean ± SD)	t	p-value	Effect Size (Cohen's d) and 95% CI
Blood Urea Change	0.616 ± 0.049	0.601 ± 0.040	1.52	0.13	d = .33, CI

Variable	Intervention Group (Mean ± SD)	Control Group (Mean ± SD)	t	p-value	Effect Size (Cohen's d) and 95% CI
					[−0.005, 0.035]
Weight Change	1.96 ± 1.13	2.02 ± 0.91	−0.25	0.80	d = 0.06, CI [−0.45, 0.33]
BUN Change	36.13 ± 6.70	33.54 ± 5.59	1.85	0.07	d = 0.42, CI [−0.3, 5.5]

Between-group comparisons were conducted using independent samples t-tests. Effect sizes were calculated using Cohen's d with 95% confidence intervals.

Table 4 shows the post-intervention Kt/V values, which serve as an indicator of dialysis adequacy. No statistically significant difference was found between the intervention and control groups (p = 0.11) (Figure 3).

Figure 3. Comparison of post-intervention Kt/V values between the intervention and control groups.

Importantly, no adverse events or clinical complications related to the exercise intervention were reported during the 4-week study period.

Table 4. Comparison of Mean Kt/V Values After the Intervention Between the Intervention and Control Groups in Patients Hospitalized in Bushehr, Iran (2020)

Group	Intervention Group (Mean ± SD)	Control Group (Mean ± SD)	t	p-value	Effect Size (Cohen's d) and 95% CI
Mean Kt/V	1.163 ± 0.14	1.115 ± 0.12	1.62	0.11	d = 0.37, CI [−0.01, 0.11]

A between-group comparison was performed using an independent-samples t-test. Effect size was calculated using Cohen's d with a 95% confidence interval.

Discussion

The findings of this study revealed that, although BUN levels and body weight decreased in the intervention group following the structured physical activity program, between-group comparisons did not yield statistically significant differences. Likewise, the Kt/V ratio showed a modest improvement in the intervention group, but this change was not important compared with the control group. In the following sections, these findings are examined in relation to the study's original objectives, interpreted alongside previous research, and discussed in the context of methodological limitations.

1. Blood Urea and BUN Levels

Previous studies have reported varying outcomes regarding the impact of intradialytic exercise on dialysis adequacy, with differences in exercise type, intensity, and duration likely contributing to

these discrepancies. A systematic review by Kirkman et al. (2019) concluded that simple interventions, such as pedaling, especially when implemented for short durations, do not significantly improve dialysis adequacy indicators, such as Kt/V (37). This finding aligns with the present study, which found that a 4-week supine pedaling protocol did not yield significant between-group differences in Kt/V. However, within-group reductions in body weight and BUN were observed.

In contrast, Rohmah et al. (2024) demonstrated that an eight-week intradialytic aerobic exercise program led to significant improvements in Kt/V and patient quality of life. Their intervention involved moderate-intensity stationary cycling under clinical supervision (39). The differences in intervention duration, exercise intensity, and program structure likely contributed to the divergent outcomes.

One plausible explanation for the lack of significant change in our study is the limited duration and intensity of the exercise protocol. The 15-minute pedaling sessions may have been insufficient to stimulate meaningful biochemical shifts. Additionally, while exercise can increase muscle perfusion and mobilize urea into circulation (53,54), the dialysis process may not have been optimized to capture and remove these solutes effectively within the short timeframe.

Taken together, the effectiveness of intradialytic exercise appears to depend on multiple factors, including modality, intensity, duration, and patient characteristics. The findings of this study, when considered alongside existing evidence, underscore the importance of carefully designed exercise interventions tailored to the clinical context and patient capacity to achieve meaningful improvements in dialysis adequacy.

2. Body Weight Changes

Although no significant difference in post-intervention weight was observed between the intervention and control groups, both groups experienced a statistically significant reduction in weight over time. This may reflect fluid shifts, dietary modifications, or other non-intervention-related factors. These findings differ from Pujiastuti et al. (2020) (49), who reported weight loss directly attributable to structured exercise. The discrepancy may be due to differences in intervention duration, exercise intensity, or baseline characteristics of the study populations.

3. Dialysis Adequacy (Kt/V)

In the present study, the intervention consisted of 15-minute pedaling sessions conducted in two shifts with a 15-minute rest interval, three times per week for four weeks. While this protocol was designed to be feasible and well tolerated in the dialysis setting, some studies suggest that such short-duration aerobic activity may be insufficient to elicit significant biochemical changes. Moreover, practical limitations—including patient fatigue, intolerance to extended dialysis time, and variability in dialysis machine performance—may have constrained the intervention's potential impact. These contextual and procedural constraints may help explain the lack of significant improvement observed in dialysis adequacy.

The intervention did not significantly improve dialysis adequacy as measured by Kt/V. This result aligns with previous studies by Hatef et al. (2017) (54), Kirkman (2019) (37), and Riahi (2012) [38], which also found no significant enhancement in dialysis efficiency following intradialytic or home-based exercise. Several factors may contribute to this outcome, including vascular access limitations, hemodynamic instability, patient non-compliance, and variability in dialysis machine performance (51).

Although vascular access quality (e.g., end-venous stenosis, weak arteriovenous fistula) was not directly measured in our study, its potential influence on dialysis adequacy was considered in the interpretation of results. In our clinical setting, most patients utilized arteriovenous fistulas with variable maturation and flow characteristics. Yet, no standardized assessment of access function—such as flow rate measurement or stenosis grading—was performed. This methodological limitation may have contributed to variability in solute clearance, particularly among patients with borderline access function or intermittent flow issues. In contrast, Vaithilingam et al. (2004) included patients with stable and well-functioning vascular access as part of a chronic intervention involving combined aerobic and resistance training (43). Their pre-screening approach likely minimized confounding effects related to access quality. Similarly, Pu et al. (2019) applied exclusion criteria based on vascular access status to ensure uniformity in dialysis delivery during their short-term aerobic intervention (44). These differences in patient selection and access control may partly explain the more favorable outcomes reported in those studies. Future research should incorporate more robust objective vascular access assessments to isolate the effects of intradialytic exercise on dialysis adequacy and reduce methodological heterogeneity.

However, differences in exercise modality and duration across studies may also explain the variability in findings. For instance, Vaithilingam et al. (2004) implemented a combined aerobic and resistance training program over a chronic intervention period, reporting significant improvements in Kt/V (43). This multimodal, long-term approach contrasts with our protocol, which involved moderate-intensity pedaling during dialysis sessions for 8 weeks. In contrast, Pu et al. (2019) employed a short-term aerobic intervention similar to ours and observed modest but favorable changes in dialysis adequacy (44). Our findings align more closely with those of Pu et

al. (2019), suggesting that even low-complexity aerobic exercise may improve Kt/V when performed consistently (44).

Other studies, such as those by Parsons (2006) and Basiri Moghadam et al. (2016), reported improvements in dialysis adequacy with longer or more intensive interventions, including 60-minute pedaling sessions or relaxation techniques (45,49). These results reinforce the notion that both duration and modality play critical roles in achieving measurable benefits. The relatively short and moderate-intensity protocol used in our study may not have provided sufficient stimulus to elicit significant changes in solute clearance. One limitation of our study is the presence of baseline differences in age and educational level between the intervention and control groups. These variables may have influenced patients' compliance, exercise tolerance, and ultimately dialysis adequacy. No covariate-adjusted analysis was conducted due to the limited sample size. As a result, the observed differences in baseline characteristics may have introduced bias. Future studies should consider statistical adjustments, such as ANCOVA or propensity score matching, to minimize confounding and improve internal validity.

4. Influence of Demographic Variables

Statistically significant differences in age and educational level between the intervention and control groups may have influenced the outcomes. Older patients may experience reduced physical capacity, motivation, and adaptability to new routines (55). At the same time, individuals with higher educational attainment often demonstrate better health literacy, self-regulation, and adherence to structured interventions (56). These demographic disparities could have contributed to the relatively non-significant findings and should be carefully considered in future research

designs. Stratified random sampling or statistical adjustments such as ANCOVA are recommended to control for baseline differences and improve internal validity.

Furthermore, under the conditions of this study, no statistically significant improvement in dialysis adequacy was demonstrated (e.g., Kt/V, $p = 0.11$). Given the limited sample size, the possibility of a Type II error cannot be ruled out. Therefore, caution is warranted in interpreting these findings, and future studies with larger sample sizes and more robust designs are recommended to assess the potential effects of intradialytic exercise better.

5. Practical Implications and Recommendations

Although the intervention did not yield statistically significant improvements in dialysis adequacy or biochemical markers, it was well tolerated and feasible within the clinical setting. For patients undergoing hemodialysis, even modest physical activity may offer psychological benefits, improved circulation, and enhanced engagement with care routines (55,56). The absence of adverse effects suggests that intradialytic exercise can be safely integrated into dialysis sessions, provided that protocols are tailored to individual capabilities (47,48).

To enhance clinical impact, future studies should consider extending the duration and intensity of exercise, incorporating multimodal approaches (e.g., combining physical activity with relaxation techniques), and ensuring demographic balance across study groups. Additionally, outcome measures should include patient-reported experiences and functional assessments to capture the broader benefits of intradialytic interventions beyond biochemical parameters.

In summary, while the present study did not demonstrate statistically significant improvements in dialysis adequacy or biochemical markers, it contributes meaningfully to the growing body of evidence supporting the feasibility of intradialytic physical activity—particularly in regions where

environmental conditions limit outdoor exercise. By implementing a structured, nurse-supervised intervention in Bushehr's dialysis centers, this study addresses both clinical and contextual gaps in prior research. The absence of adverse effects, combined with high patient tolerance, suggests that such interventions can be safely integrated into routine care. With appropriate adjustments in duration, intensity, and patient stratification, intradialytic exercise may offer a scalable strategy to enhance dialysis outcomes and quality of life in similar regional and clinical contexts.

Conclusion

In conclusion, this randomized controlled trial found that a 4-week intradialytic exercise intervention consisting of pedaling exercises during hemodialysis sessions did not result in a statistically significant improvement in dialysis adequacy, as measured by Kt/V ($p = 0.11$). Therefore, given the observed lack of effect of this specific physical activity protocol on dialysis adequacy, future research should prioritize a comprehensive re-evaluation of the intervention parameters. Key considerations for subsequent investigations should include the careful selection of exercise modalities, optimization of session duration, and determination of the most efficacious timing of exercise relative to the hemodialysis procedure. Furthermore, rigorous control of confounding variables, such as patient age and educational attainment, is essential to minimize potential bias and ensure the validity of future findings. By systematically addressing these factors, future studies may be better positioned to identify effective strategies for integrating physical activity into the hemodialysis routine to improve patient outcomes.

Although no sensitivity analysis or age-matching was performed in the present study, the observed age differences between groups may have influenced the outcomes. Future trials should consider

stratified randomization or statistical adjustments (e.g., ANCOVA) to account for age-related variability and enhance internal validity.

Limitations

Several limitations should be considered when interpreting these findings, as they impact both the internal and external validity of the study. First, the relatively small sample size (n = 79 after dropouts) may have limited the study's ability to detect minor yet potentially clinically significant differences between groups. Future research should aim for larger sample sizes to enhance statistical power. Second, the study focused exclusively on pedaling exercise performed at a relatively low intensity. The effects of other exercise modalities, such as resistance training, higher-intensity aerobic exercise, or combined programs, were not examined, and the results may not apply to these interventions. Different exercise types and intensities may have varying effects on dialysis adequacy and other patient outcomes. Third, the single-blind design, in which participants were unaware of their group assignment, may have reduced participant-related bias. However, the lack of blinding for care providers and/or outcome assessors could have introduced some performance or detection bias. Fourth, the study population consisted of hemodialysis patients in Bushehr, Iran, with specific inclusion criteria (ages 18-65, no severe comorbidities). Consequently, the results might not be generalizable to patients in other locations, with different demographic profiles, or with more complex medical conditions. The experimental and control groups were similar demographically, except for age (χ^2 = -3.84, p = 0.001) and education level (χ^2 = 10.100, p = 0.006), which could have introduced additional bias. Fifth, convenience sampling may limit the generalizability of findings to other hemodialysis populations. Participants were recruited from a single center in Bushehr. Sixth, the lack of pre-intervention Kt/V measurements limits longitudinal analysis. Since the intradialytic exercise protocol started from the first dialysis session, obtaining

a true baseline measurement was not feasible. This approach was chosen to maintain clinical feasibility and minimize disruption to standard care.

Finally, this study was conducted at a single center, and the same treatment team monitored all patients throughout the study. The researcher provided the patient with a bike, and the patient was monitored by the treatment team during the activity, making the results difficult to generalize given different treatment teams, dialysis protocols, or staffing levels. Taken together, these factors highlight the need for caution when generalizing the findings to broader hemodialysis populations or different clinical settings.

Acknowledgments

This article is derived from a Master's thesis conducted with financial support from Bushehr University of Medical Sciences. We sincerely thank the hemodialysis patients at the hospitals' dialysis departments in Bushehr for their participation and cooperation.

Financial Disclosure

This research was funded by the Vice-Chancellery of Research and Technology at Bushehr University of Medical Sciences (Grant number IR.BPUMS.REC.1398.130).

Conflict of interest :None declared.

Data Availability Statement

Yes- all data are fully available

References

1. Kebede, K. M., Abateneh, D. D., Teferi, M. B., & Asres, A. (2022). Chronic kidney disease and associated factors among adult population in Southwest Ethiopia. *PLOS ONE*, 17(3), e0264611. https://doi.org/10.1371/journal.pone.0264611
2. Li, Z., He, R., Wang, Y., & others. (2025). Global trends of chronic kidney disease from 1990 to 2021: A systematic analysis for the Global Burden of Disease Study 2021. *BMC Nephrology*, 26, 385. https://doi.org/10.1186/s12882-025-04309-7
3. Kovesdy, C. P. (2022). Epidemiology of chronic kidney disease: An update 2022. *Kidney International Supplements*, 12(1), 7–11. https://doi.org/10.1016/j.kisu.2021.11.003
4. Rico-Fontalvo, J., Elbert, A., Lorca, E., & Daza-Arnedo, R. (2025). Situation of chronic kidney disease in Latin America, with emphasis on diabetic kidney disease: Difficulties and challenges. *Nefrología Latinoamericana*, 22. https://doi.org/10.24875/NEFRO.M25000058
5. Bouya, S., Balouchi, A., Rafiemanesh, H., & Hesaraki, M. (2018). Prevalence of chronic kidney disease in Iranian general population: A meta-analysis and systematic review. *Therapeutic Apheresis and Dialysis*, 22(6), 594–599. https://doi.org/10.1111/1744-9987.12716
6. Reynaga, O. L., & Olivares, D. E. M. (2024). Chronic kidney disease in Mexico: Challenges and proposals. *CuidArte*, 13(25). https://www.medigraphic.com/cgi-bin/new/resumenI.cgi?IDARTICULO=115194
7. Centers for Disease Control and Prevention. (2023). *Chronic kidney disease in the United States, 2023*. U.S. Department of Health and Human Services. https://www.cdc.gov/kidney-disease/php/data-research/index.html
8. Shah, M. K., & Winkelmayer, W. C. (2021). A global accounting of kidney replacement therapy. *Kidney International*, 77(3), 309–311. https://www.ajkd.org/article/S0272-6386(20)31078-7/fulltext
9. Grobman, B., Bondarchuk, C. P., Mansur, A., & Lu, C. Y. (2025). Trends and disparities in deaths from kidney disease among older adults in the United States. *Journal of Clinical Medicine*, 14(14), 4950. https://doi.org/10.3390/jcm14144950

10. Institute for Health Metrics and Evaluation. (2023). *Global Burden of Disease Study 2021 (GBD 2021) Results*. University of Washington. https://www.healthdata.org/research-analysis/gbd

11. Dehghani, A., Alishavandi, S., Nourimajalan, N., Fallahzadeh, H., & Rahmanian, V. (2022). Prevalence of chronic kidney diseases and its determinants among Iranian adults: Results of the first phase of Shahedieh cohort study. *BMC Nephrology*. https://doi.org/10.1186/s12882-022-02832-5

12. Wang, Q., Zhang, Y., Chen, L., & Liu, H. (2024). Epidemiological shifts in chronic kidney disease: A 30-year global and regional assessment. *BMC Public Health*, 24, Article 3519. https://doi.org/10.1186/s12889-024-21065-9

13. Daugirdas, J. T., Blake, P. G., & Ing, T. S. (2015). *Handbook of dialysis* (5th ed.). Wolters Kluwer.

14. Chander, S., & others. (2024). Mortality and mode of dialysis: A meta-analysis and systematic review. *BMC Nephrology*, 25, Article 435. https://doi.org/10.1186/s12882-023-03435-4

15. Echefu, G., Stowe, I., Burka, S., Basu-Ray, I., & Kumbala, D. (2023). Pathophysiological concepts and screening of cardiovascular disease in dialysis patients. *Frontiers in Nephrology*, 3, Article 1198560. https://doi.org/10.3389/fneph.2023.1198560

16. Denguir, S., Hellberg, M., Almquist, M., & Clyne, N. (2025). Survival in patients with CKD 3–5 after 12 months of exercise training: A post-hoc analysis of the RENEXC trial. *BMC Nephrology*, 26, Article 36. https://doi.org/10.1186/s12882-024-03236-9

17. Yari, M., Rahim, F., Karimyan, E. M., Khafaie, M. A., Banari, M., & Valipour, A. (2024). Survival rate and associated risk factors among hemodialysis patients in Southwest Iran: A retrospective cohort study. *Jundishapur Journal of Chronic Disease Care*, 13(1), Article e138089. https://doi.org/10.5812/jjcdc-138089

18. Ryou, S., & Yoon, H. E. (2024). The current state of life expectancy of hemodialysis patients in Korea. *Kidney Research and Clinical Practice*, 43(5), 562–564. https://doi.org/10.23876/j.krcp.24.099

19. Joseph, S., Farooqi, F. A., Afsar Sheriff, M. A., Kotb Fouda, A. M., Jacob, B., Kochudevassy, J., & others. (2025). 13 Critical significances of adequate hemodialysis in enhancing survival rate and quality of life for dialysis dependent patients at Hamad General Hospital Qatar. *BMJ Open Quality*, 14. https://doi.org/10.1136/bmjoq-2025-IHI.13

20. KDOQI. (2015). *Clinical practice guidelines for hemodialysis adequacy: Update 2015*. National Kidney Foundation.

21. Ren, S., Yao, X., Ren, S., & Feng, Y. (2023). Oral nutritional supplement helps to improve nutritional status of dialysis dependent patients: A systematic review and meta-analysis of randomized controlled trials. *Frontiers in Nutrition*, 10, 1294064. https://doi.org/10.3389/fnut.2023.1294064

22. Dehvan, F., Monjazebi, F., Erfani Khanghahi, M., Mohammadi, H., & Ghanei Gheshlagh, R. (2018). Adequacy of dialysis in Iranian patients undergoing hemodialysis: A systematic review and meta-analysis. *Nephro-Urology Monthly*, 10(5), e82235. https://doi.org/10.5812/numonthly.82235

23. Rayani, A. M., Smart, M. W., Alreshidi, S. M., & others. (2025). Correlations between spirituality, anxiety, and depression in hemodialysis patients in Saudi Arabia: A cross-sectional study. *Journal of Religion and Health*, 64, 2558–2575. https://doi.org/10.1007/s10943-024-02239-6

24. Iman, Y., Bamforth, R., Ewhrudjakpor, R., Komenda, P., Gorbe, K., Whitlock, R., Bohm, C., Tangri, N., & Collister, D. (2024). The impact of dialysate flow rate on haemodialysis adequacy: A systematic review and meta-analysis. *Clinical Kidney Journal*, 17(7). https://doi.org/10.1093/ckj/sfae163

25. Rubin, D. M., Letts, R. F. R., Richards, X. L., & others. (2024). Rapid prototyping of multi-compartment models for urea kinetics in hemodialysis: A system dynamics approach. *Journal of Artificial Organs*, 27, 316–320. https://doi.org/10.1007/s10047-023-01416-w

26. Grandy, J., Ortega, M., & Bofill, L. (2024). Feasibility of incremental haemodialysis in paediatrics: Preliminary insights from a small cohort. *Journal of Nephrology*, 37, 2671–2674. https://doi.org/10.1007/s40620-024-02112-5

27. Kubanek, A., Renke, M., Godlewska, B. R., Paul, P., Przybylak, M., Kowalska, A. S., Wąż, P., Błaszczyk, M., Rutkowski, P., Czarnacka, K., Bednarski, K., & Grabowski, J. (2024). Screening for depression in chronic haemodialysis patients as a part of care in dialysis setting: A cross-sectional study. *Frontiers in Psychiatry*, 15, 1410252. https://doi.org/10.3389/fpsyt.2024.1410252

28. Nablawi, R. A., Alghamdi, L. S., Alshaikh, A. E., Alagha, A. M., Alharbi, N. T., Ali, A. H., Khafaji, H. A., Zarei, E. W., & Alzahrani, N. A. (2025). Quality of life and depression among chronic kidney disease patients: A tertiary care center cross-sectional study. *Frontiers in Nephrology*, 5, 1600296. https://doi.org/10.3389/fneph.2025.1600296

29. Hall, R. K., & Haines, C. (2020). Psychological burden of dialysis. *Clinical Journal of the American Society of Nephrology*, 15(2), 234–240.

30. Ishiwatari, M., & Fujii, H. (2020). Mental health in hemodialysis. *Psychiatry and Clinical Neurosciences*, 74(5), 267–273.

31. Bastani, B., & Heidari, R. (2017). Muscle atrophy in dialysis patients. *Iranian Journal of Kidney Diseases*, 11(4), 290–295.

32. Deligiannis, A., & Kouidi, E. (2021). Exercise in dialysis: A review. *Nephrology*, 26(1), 1–10.

33. Torres, C., & Gonzalez, M. (2020). Intradialytic cycling and outcomes. *Journal of Renal Nutrition*, 30(3), 234–240.

34. Adenwalla, S., & Thomas, G. (2025). Feasibility of intradialytic exercise. *International Journal of Nephrology*, Article ID 123456.

35. Jiang, H., & Liu, Y. (2025). Nurse-led exercise programs in dialysis. *BMC Nursing*, 24(1), 1–9.

36. Dal Koyuncuoğlu, H. (2025). Barriers to exercise in dialysis patients. *Turkish Journal of Nephrology*, 34(2), 112–118.

37. Kirkman, D. L., Scott, M., Kidd, J., & Macdonald, J. (2019). The effects of intradialytic exercise on hemodialysis adequacy: A systematic review. *Seminars in Dialysis*. Available from: https://research.bangor.ac.uk/files/23673616/2019_Dialysis_Adequacy.pdf

38. Riahi, M., & Ghorbanian, A. (2012). Home-based exercise and dialysis. *Iranian Journal of Nursing and Midwifery Research*, 17(6), 421–426.

39. Rohmah, S. N., Puspitasari, M., Prasanto, H., Wardhani, Y., Kuswadi, I., & Dhamarjati, A. (2024). Effect of intradialytic aerobic exercise intervention on dialysis adequacy and quality of life in patients with end-stage kidney disease undergoing hemodialysis. *International Urology and Nephrology*, 56, 3595–3604. https://doi.org/10.1007/s11255-024-04100-x

40. Omranikhoo, H., & Shamsi, A. (2025). Climate and physical activity in Bushehr. *Iranian Journal of Environmental Health*, 22(1), 55–62.

41. Schulz, K. F., Altman, D. G., & Moher, D. (2010). CONSORT 2010 Statement: Updated guidelines for reporting parallel group randomized trials. *Annals of Internal Medicine*, 152(11), 726–732. https://doi.org/10.7326/0003-4819-152-11-201006010-00232

42. Jafari, M., & Ghods, A. (2015). Validity of dialysis adequacy measurement tools in Iranian patients. *Iranian Journal of Nephrology*, 9(1), 33–40.

43. Vaithilingam, I., Polkinghorne, K. R., Atkins, R. C., & Kerr, P. G. (2004). Time and exercise improve solute removal in hemodialysis patients. *Journal of the American Society of Nephrology*, 15(3), 746–754.

44. Pu, J., Jiang, Z., Wu, W., Li, L., Zhang, L., Li, Y., Liu, Q., & Ou, S. (2019). Efficacy and safety of intradialytic exercise in haemodialysis patients: A systematic review and meta-analysis. *BMJ Open*, 9(1), e020633. https://doi.org/10.1136/bmjopen-2017-020633

45. Parsons, T. L., & Toffelmire, E. B. (2006). The effect of exercise on dialysis adequacy in hemodialysis patients. *Nephrology Nursing Journal*, 33(6), 653–660.

46. Slade, S. C., Dionne, C. E., Underwood, M., & Buchbinder, R. (2016). Consensus on Exercise Reporting Template (CERT): Explanation and elaboration statement. *British Journal of Sports Medicine*, 50(23), 1428–1437.

47. Soltani, S., Ghods, A., & Shafiee, M. (2020). Intradialytic cycling and its impact on dialysis adequacy: A clinical trial. *Iranian Journal of Kidney Diseases*, 14(2), 123–130. https://doi.org/10.34172/jpe.2020.21

48. Foran, M., & O'Sullivan, D. (2003). Safety of exercise during hemodialysis: A pilot study. *Nephrology Nursing Journal*, 30(3), 285–290.

49. Pujiastuti, E., & Sari, N. (2020). Implementation of intradialytic exercise in clinical settings: A nursing perspective. *International Journal of Nursing Practice*, 26(4), e12845. https://doi.org/10.1111/ijn.12845

50. Motedayen, M., & Ebrahimi, H. (2014). Hemodialysis adequacy and patient outcomes in Bushehr hospitals. *Bushehr Medical Journal*, 18(2), 75–82.

51. Biabani, S., & Shamsi, A. (2019). Intradialytic exercise and its effects on dialysis adequacy: A randomized trial. *Iranian Journal of Nursing and Midwifery Research*, 24(3), 210–216.

52. Nezamlo, A., Oshvand, K., Ghlyaf, M., & Homayounfar, S. (2015). The effects of physical training during dialysis on the prevention of anemia in hemodialysis patients. *Avicenna Journal of Nursing and Midwifery Care*, 23(4), 65–76.

53. Saglimbene, V., Wong, G., Ruospo, M., Palmer, S. C., & Strippoli, G. F. M. (2017). Exercise for adults undergoing hemodialysis: A systematic review and meta-analysis. *American Journal of Kidney Diseases*, 69(5), 687–698. https://doi.org/10.1053/j.ajkd.2016.10.019

54. Hatef, M., & Ghaffari, A. (2017). Ethical considerations in clinical trials involving dialysis patients. *Iranian Journal of Medical Ethics*, 11(3), 45–52.

55. Lubans, D. R., Richards, J., Hillman, C. H., Faulkner, G., & Beauchamp, M. R. (2016). Physical activity for cognitive and mental health in youth: A systematic review. *American Journal of Preventive Medicine*, 51(6), 954–962. https://doi.org/10.1542/peds.2016-1642

56. Plotnikoff, R. C., Costigan, S. A., Williams, R. L., & others. (2015). Effectiveness of interventions targeting physical activity, nutrition and healthy weight for university and

college students: A systematic review and meta-analysis. *International Journal of Behavioral Nutrition and Physical Activity*, 12, 45. https://doi.org/10.1186/s12966-015-0203-7

Supporting Information Captions

S1 File. CONSORT 2010 Checklist. This file contains the completed CONSORT checklist used to ensure transparent reporting of the randomized clinical trial.

S2 File. Persian Original Thesis. This file includes the full Persian-language thesis of the Master of Nursing student whose research formed the basis of the current manuscript. It contains the complete trial protocol and data documentation.

S3 File. Trial Protocol. This document outlines the original protocol approved by the institutional ethics committee, including study design, intervention procedures, and outcome measures.

S4 File. Ethics Approval Certificate. This file contains the official ethics approval letter issued by the Research Ethics Committee of Bushehr University of Medical Sciences for the submitted study.

S5 File. This file contains the fully anonymized SPSS dataset used for statistical analysis in the study. It is provided as Supporting Information and is publicly available to ensure transparency and reproducibility of the findings.

 Click here to access/download;Figure;figure 1.png

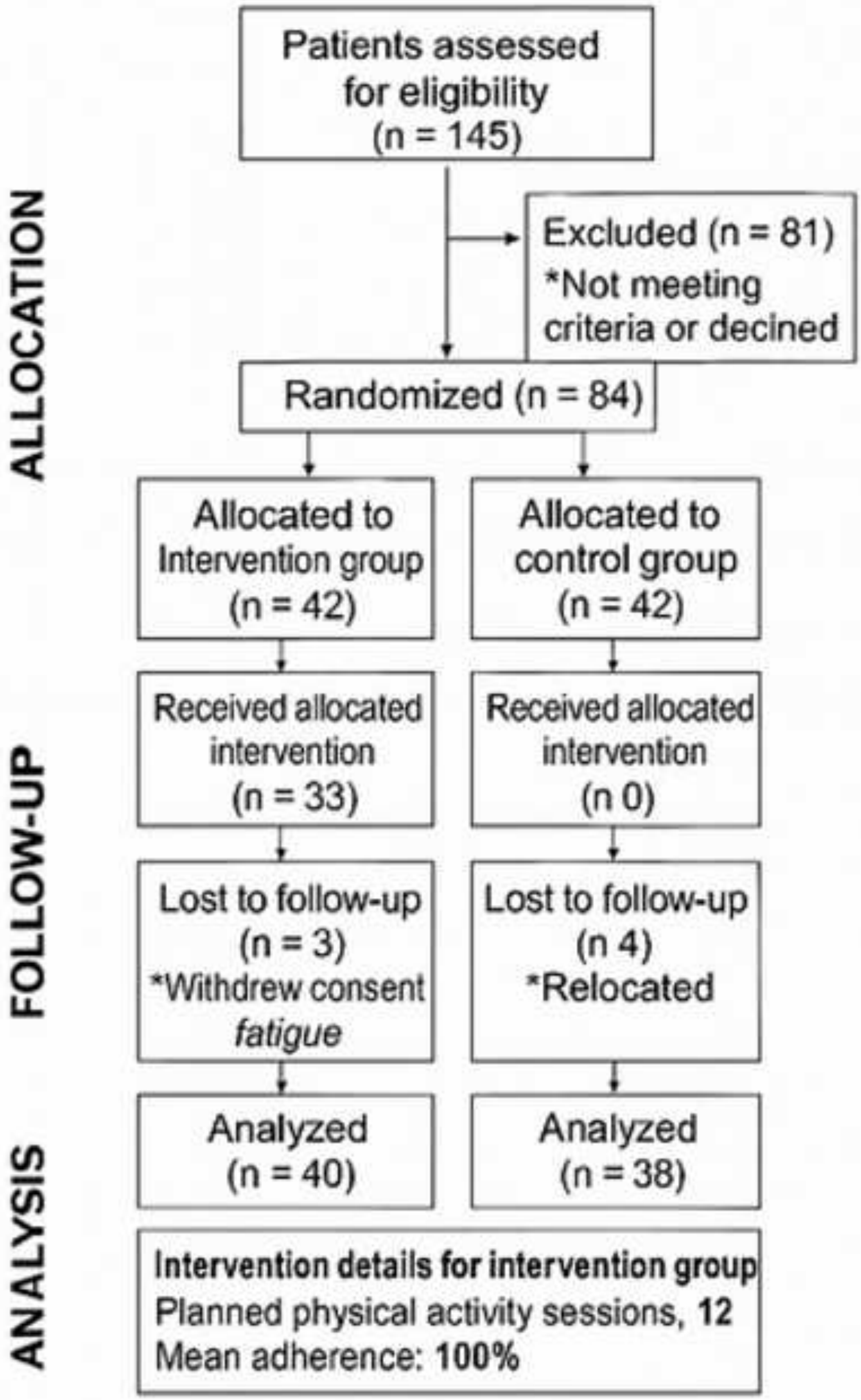

Figure 2

Click here to access/download;Figure;Figure 2.png

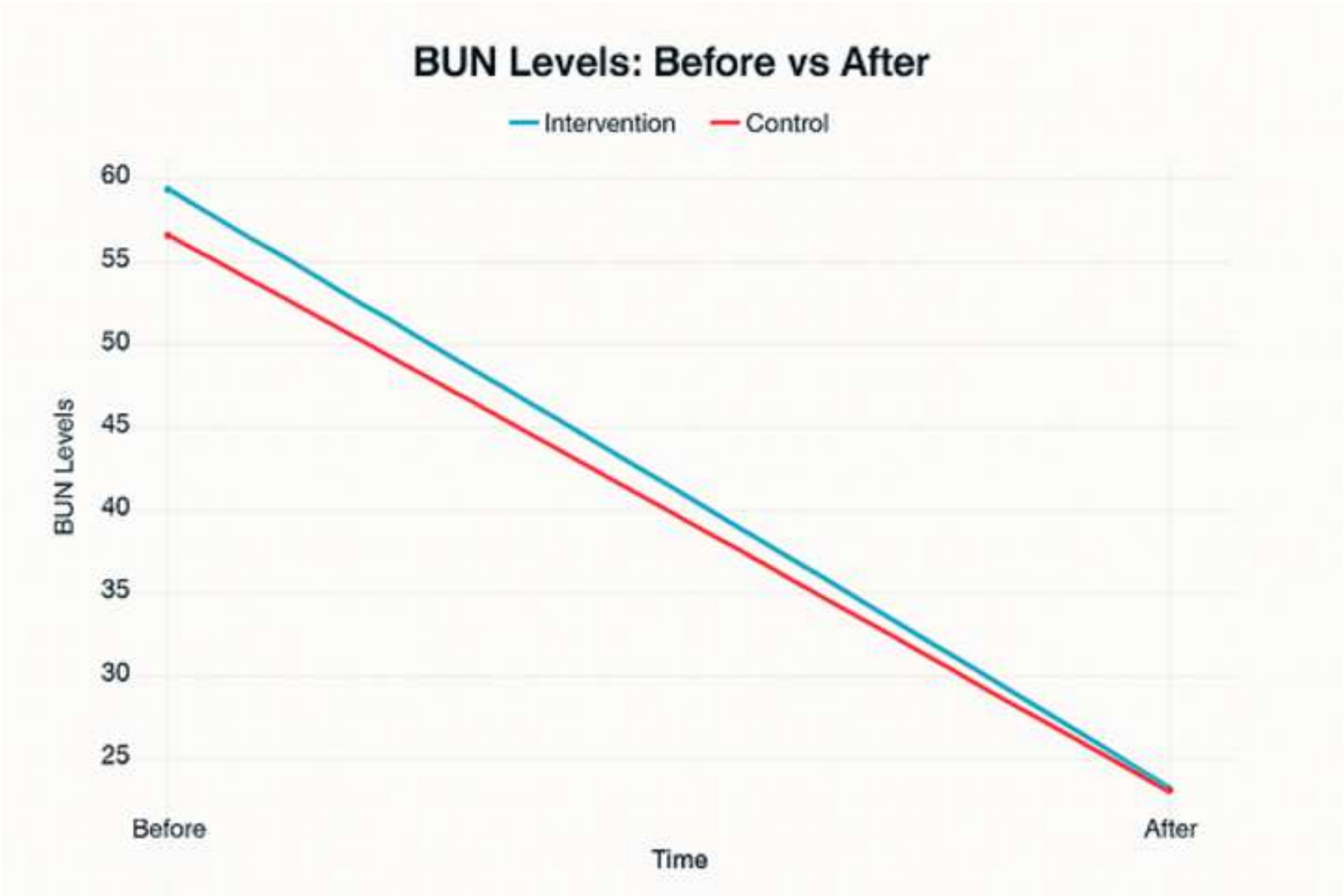

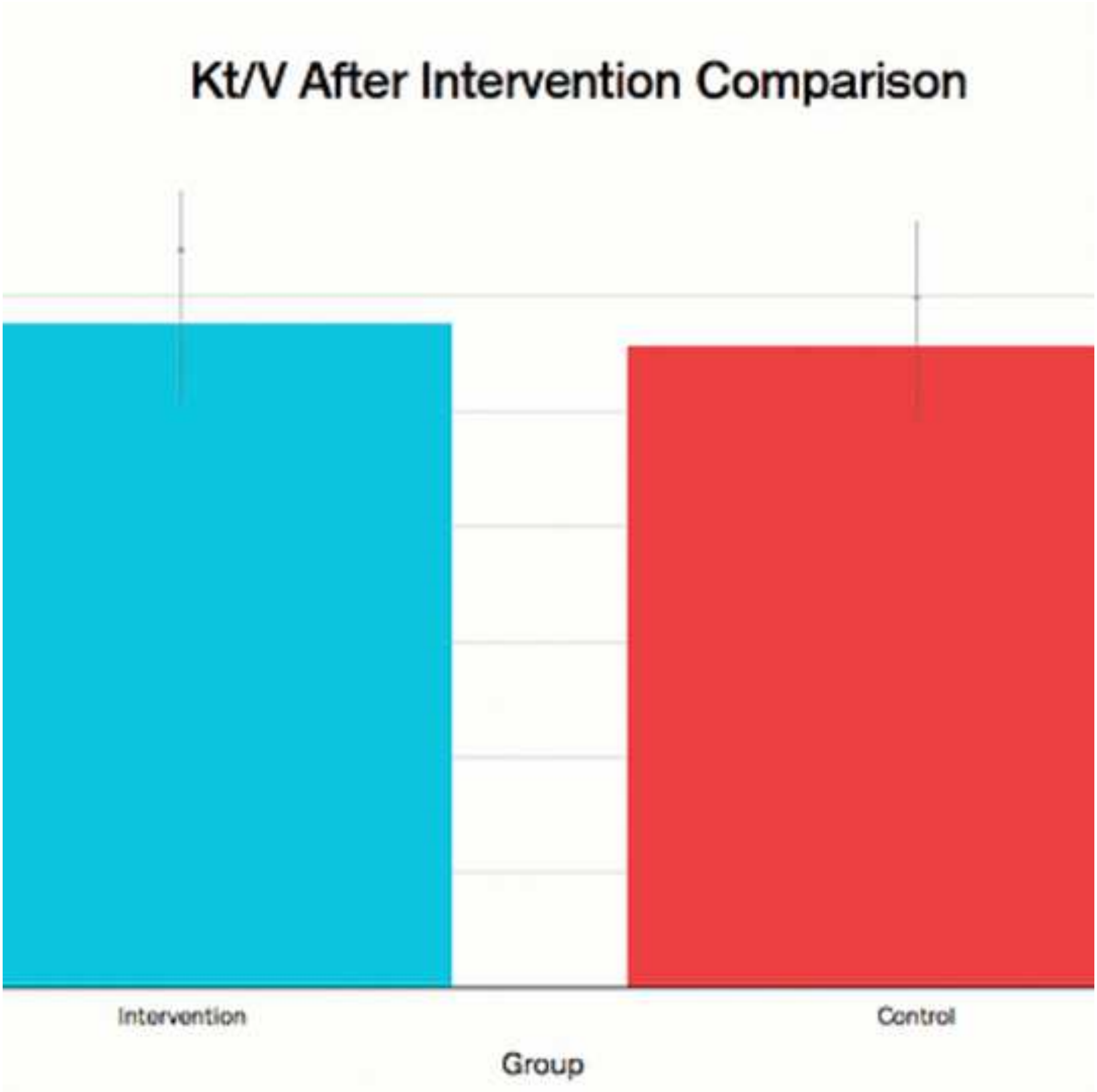
Kt/V After Intervention Comparison
Intervention
Control
Group

Click here to access/download
Supporting Information
CONSORT-2010-Checklist (1).doc

Click here to access/download
Supporting Information
persian original thesis.pdf

Click here to access/download
Supporting Information
PROTOCOL.docx

Click here to access/download
Supporting Information
Research Ethics Certificate.pdf

Click here to access/download
Supporting Information - Compressed/ZIP File Archive
Data Set.sav

Effect of Intra-Dialytic Pedaling Exercise on Dialysis Adequacy: A Randomized Controlled Trial

Mahmoud Mohamadizadeh (MSc)[1], Sharif Sharifi (MSc)[2], Niloufar Motamed (Ph.D.)[3], Mohammad Amin Shadman (MSc)[4], Shahnaz Pouladi (Ph.D.) *[5]

1. Master's student in medical-surgical nursing, student research committee, Bushehr University of Medical Sciences, Bushehr, Islamic Republic of Iran. Mahmoud.2580@yahoo.com
2. MSc in medical-surgical nursing, Nursing and Midwifery Faculty of Bushehr University of Medical Sciences, Bushehr, Islamic Republic of Iran.
3. PhD in community medicine, Department of Community Medicine, Medicine faculty of Bushehr University of Medical Sciences, Bushehr, Islamic Republic of Iran.
4. MSc in medical-surgical nursing, Nursing and Midwifery Faculty of Bushehr University of Medical Sciences, Bushehr, Islamic Republic of Iran.
5. PhD in Nursing Education, Associate Professor, Nursing and Midwifery Faculty of Bushehr University of Medical Sciences, Bushehr, Islamic Republic of Iran. pouladi2008@gmail.com

Abstract

Background: In patients with chronic kidney disease undergoing hemodialysis, physical activity and rehabilitation are crucial for preventing declines in muscle strength and functional capacity. This study aimed to assess the impact of physical activity during hemodialysis on dialysis adequacy in these patients.

Methods: This randomized controlled trial (RCT) investigated the impact of pedaling exercise on dialysis effectiveness in 84 hemodialysis patients at hospitals in Bushehr. Participants were randomly assigned to either an experimental group (n=42) that performed 30 minutes of pedaling exercise during their 4-hour dialysis sessions or a control group (n=42) that received routine hemodialysis. Dialysis adequacy was assessed by comparing pre- and post-dialysis blood samples obtained from the arterial line. A standardized protocol was used to collect blood samples, and patient weight was measured with a calibrated digital scale. Data analysis was performed using SPSS version 24 software.

Results: The experimental and control groups were similar in demographic characteristics, except for age (X2 = -3.84, p = 0.001) and education levels (X2 = 10.100, p = 0.006). While there was no significant difference in weight between the groups before and after the intervention (t=0.223, p=0.82 before; t=0.203, p=0.84 after), both groups showed a substantial weight reduction overall (p<0.0001). There were no statistically significant differences in weight change (t=0.80, p=0.25), BUN (t=0.13, p=1.52), or Kt/V (t=1.62, p=0.11) between the experimental and control groups.

Conclusion: This study found that incorporating pedaling exercise during hemodialysis did not significantly improve dialysis effectiveness, as measured by weight change, BUN levels, or Kt/V.

While both groups showed weight loss, there were no statistically significant differences between them. However, the study had limitations due to its small sample size and the specific type of exercise used. Further research with larger cohorts and a broader range of physical activities is needed to determine whether physical activity during hemodialysis can improve dialysis adequacy and overall patient outcomes.

Trial registration: IRCT code number 20150529022466N15 and trial Code of Ethics IR.BPUMS.REC.1398.130

Keywords: physical activity, dialysis adequacy, chronic kidney disease, hemodialysis

Introduction

Chronic kidney disease (CKD) is a significant global health problem, leading to both short- and long-term complications and reducing patients' quality of life (1,2). Recent studies indicate that CKD prevalence varies by region (3,4), with estimates of 11.4% in Iran (5), 9% in Mexico (6), and 13.1% among adults in the United States (7). Several treatment options are available for managing CKD, including kidney transplantation, peritoneal dialysis, and hemodialysis (8). Nearly 4 million people worldwide rely on kidney replacement therapy (KRT), with hemodialysis being the most common, accounting for about 69% of all KRT and 89% of dialysis cases (9). CKD is projected to become the fifth leading cause of death globally by 2040 (10). Given population growth, an annual increase of 5–6% in CKD prevalence poses a challenge for all countries worldwide. In Iran, this growth rate surpasses the global average (11).

Hemodialysis remains the most widely utilized modality for kidney replacement therapy worldwide, accounting for the majority of dialysis cases and imposing substantial financial and operational burdens on healthcare systems. Recent global epidemiological data confirm this trend, highlighting the increasing reliance on hemodialysis across diverse regions and its implications for public health planning (12).

Different hemodialysis programs exist, including home hemodialysis (3–6 times per week), in-center hemodialysis, short daily hemodialysis, and long nocturnal hemodialysis (13). Although hemodialysis prevents death due to uremia, the survival rate of patients with CKD is significantly lower than that of the general population (14-16). Several factors influence the survival rate of these patients, including the cause of renal failure, the treatment method, the presence of comorbidities such as cardiovascular disease, diabetes, and hypertension, and the adequacy of dialysis (17,18).

The long-term prognosis of patients undergoing dialysis depends heavily on the efficacy of their treatment, commonly referred to as dialysis adequacy. Defined by the National Kidney Foundation Kidney Disease Outcomes Quality Initiative (NKF KDOQI) as sufficient urea clearance, dialysis adequacy serves as a key indicator of treatment success and patient survival (19,20). Inadequate dialysis adequacy has been associated with a range of adverse outcomes, including sexual dysfunction, cardiovascular disease, pruritus, prolonged hospitalization, and increased healthcare costs (21,22). Conversely, improved dialysis adequacy enhances patients' quality of life, physical functioning, and spiritual well-being (23). Given its central role in clinical outcomes, dialysis adequacy is not only a benchmark for evaluating dialysis protocols but also a critical endpoint in studies assessing adjunctive interventions such as intradialytic exercise. Understanding how physical activity during dialysis may influence adequacy is therefore essential to optimizing care strategies for this population.

The effectiveness of a dialysis session is commonly assessed using two key indicators: Kt/V and the urea reduction ratio (URR) (24,25). Kt/V quantifies the efficiency of dialysis in removing urea from the body. It is calculated by dividing the product of dialyzer clearance (K) and dialysis time (t) by the volume of distribution of urea, which approximates total body water (V) (26). According to the urea kinetic model, both Kt/V and URR are standard measures of dialysis adequacy. Clinically, a minimum Kt/V value of 1.2 and a target URR of approximately 65% are recommended to ensure sufficient urea clearance during treatment (20).

Patients with CKD undergoing hemodialysis often experience functional decline, reduced quality of life, and psychological disorders such as depression and anxiety related to their treatment (27-30). Hemodialysis is typically administered in a supine position, requiring patients to remain physically inactive for approximately 12 hours per week—equivalent to nearly 800 hours annually.

This prolonged sedentary time contributes to complications such as reduced physical endurance, muscle atrophy, and muscle weakness (31). Incorporating regular exercise during dialysis sessions has been shown to improve physical performance and mitigate muscle deterioration in hemodialysis patients (32,33).

Cycling was selected as the intervention modality due to its practicality, safety, and compatibility with the physical limitations of hemodialysis patients. Stationary pedaling can be performed in a seated or supine position, making it feasible during dialysis sessions without disrupting treatment protocols. Recent evidence suggests that intradialytic cycling is well tolerated and can be easily supervised by clinical staff, enhancing adherence and minimizing risk (34). Moreover, intradialytic exercise offers a unique opportunity to utilize otherwise sedentary treatment time for therapeutic benefit. Compared to pre- or post-dialysis exercise, intradialytic protocols allow for continuous monitoring, reduce scheduling barriers, and may improve patient engagement (35,36).

Previous studies on physical activity during dialysis have yielded conflicting results. For instance, Kirkman et al. (2019) (37) and Riahi et al. (2012) (38) reported that pedaling exercises during dialysis did not significantly improve dialysis adequacy, while Rohmah et al. (2015) (39) found that 30 minutes of cycling enhanced physical fitness and biochemical markers. These inconsistencies highlight the need for further investigation using context-sensitive and methodologically robust designs. Bushehr, located in southern Iran, faces unique environmental challenges; its hot, humid climate limits outdoor physical activity, particularly for patients with chronic conditions (40). Moreover, the province has a disproportionately high number of hemodialysis patients per capita. Given the central role of dialysis nurses in delivering integrated care and patient education, their involvement presents a valuable opportunity to implement structured intradialytic exercise programs (34,35). Therefore, this study was conducted to evaluate

the effectiveness of supervised physical activity during hemodialysis on dialysis adequacy in patients treated in Bushehr's clinical centers, addressing both regional constraints and methodological gaps in prior research.

Methods

Study Design

This study was a randomized controlled clinical trial (RCT; IRCT20150529022466N15) conducted between January 10 and March 30, 2020, in the dialysis departments of Bushehr hospitals in southern Iran. The trial was designed and reported in accordance with the CONSORT 2010 guidelines to ensure transparency and methodological rigor (41). The authors confirm that all ongoing and related trials for this intervention are registered.

Inclusion and Exclusion Criteria

Inclusion criteria were: age between 18 and 65 years, undergoing continuous hemodialysis treatment for at least 3 months without interruption, ability to walk independently for at least 2 minutes, no history of unstable angina, dyspnea, myocardial infarction within the past month, or congestive heart failure; no use of neuropsychiatric medications; tolerance of 4-hour dialysis sessions; and absence of hypokalemia (K < 3.5 mEq/L). These criteria were defined based on clinical guidelines and prior trials in hemodialysis populations (42).

Exclusion criteria were applied only to individuals who initially met all inclusion criteria. These included: cardiovascular complications such as unstable angina, systolic blood pressure ≥160 mmHg or diastolic ≥120 mmHg, hypotension defined as systolic BP <90 mmHg or a drop >30 mmHg during dialysis, neurological, musculoskeletal, or vascular disorders (confirmed by

physician), missing four or more dialysis sessions during the study period, kidney transplantation or death, vascular access problems, and unwillingness to continue participation (42).

Sample Size and Sampling Method

Using G*Power 3.1.9.2, with a 95% confidence level and 80% power, the required sample size was 76 participants for an expected effect size (d = 0.65). The mean and SD of dialysis adequacy (Kt/V) in the control group were 1.33 and 0.18, respectively; the expected mean in the intervention group was 1.45. To account for a 10% dropout rate, the sample size was increased to 84 (42 per group) using the formula:

$$n' = n / (1 - q)$$

A non-probabilistic convenience sampling method was used to recruit patients from hospitals in Bushehr. Although random allocation was applied, initial recruitment was based on availability and willingness, which may introduce selection bias (43).

Randomization and Blinding

Participants were randomly assigned to intervention or control groups using a computer-generated random number table by an independent researcher. Allocation was concealed using sequentially numbered, sealed opaque envelopes. The study was single-blind: participants were unaware of their group assignment. Due to the nature of the intervention, blinding of medical staff was not feasible. To minimize contamination, groups were treated in separate centers and at different times (44).

Treatment Uniformity and Vascular Access

All participants underwent conventional hemodialysis (not hemodiafiltration), three times per week for four hours per session. The dialysis modality and frequency were consistent across both groups. Although the type of vascular access (e.g., fistula, graft, or catheter) was not systematically recorded as a demographic variable, all patients had functional access suitable for standard hemodialysis. The potential influence of vascular access type on dialysis adequacy is acknowledged as a study limitation (45).

Exercise Protocol and Adherence (CERT-Based Description)

The intradialytic exercise intervention was designed and reported in accordance with the Consensus on Exercise Reporting Template (CERT) to ensure methodological transparency and replicability (46). Participants in the intervention group performed stationary cycling during dialysis using a portable ATMED (Taiwan) bike weighing 3.5 kg, positioned at the foot of the dialysis bed. All exercises were conducted in the supine position during dialysis.

Each session consisted of a 5-minute warm-up, two 15-minute pedaling intervals separated by a 15-minute rest, and a cool-down phase. The intervention lasted 4 weeks, with three sessions per week, for a total of 12. All participants in the intervention group completed the full set of exercise sessions without interruption. Kt/V values were recorded during the 12-session intervention period, starting from the first dialysis session. As the exercise protocol commenced immediately, no pre-intervention Kt/V measurements were collected. This design reflects the pragmatic integration of the intervention into routine clinical practice (47).

Exercise intensity was individualized and adjusted to achieve a 10% increase in heart rate compared to baseline. The safe pulse range during exercise was maintained at 50-125 beats per minute. Pulse and blood pressure were measured before and after each session, and pulse was

continuously monitored throughout. If the target heart rate was not achieved, patients were instructed to pedal faster. (48).

All sessions were supervised by a trained nurse familiar with the protocol. Participants received verbal encouragement and feedback to maintain motivation and adherence. The exercise protocol was standardized across all participants, and no modifications were required during the study. The equipment and setting remained consistent throughout. The full protocol is archived in the thesis repository of Bushehr University of Medical Sciences and is available upon request (49).

Participants were monitored for any abnormal signs or adverse events during each exercise session. The supervising nurse documented clinical observations throughout the intervention period as part of the safety protocol.

Data Collection Tools

Data were collected using a demographic form and a dialysis adequacy form. Variables included sex, age, education, marital status, weight (pre-/post dialysis), and dialysis adequacy indices. Kt/V was calculated using:

$$Kt/V = -\ln(R - 0.008 \times t) + (4 - 3.5 \times R) \times UF / W$$

To calculate the Urea Reduction Ratio (URR), blood urea nitrogen (BUN) levels were measured before and after dialysis.

URR = (BUN pre − BUN post) / BUN pre

Blood samples were collected pre- and post-dialysis using standard protocols. Post-dialysis samples were taken after reducing dialysate flow for 3 minutes and pump speed to <100 cc/min

for the final 5 minutes. Samples were coded and analyzed using a DIRUI 1200 machine. Weight was measured using a calibrated Seca digital scale (42).

Implementation

After the study protocol was approved by the Ethics Committee of Bushehr University of Medical Sciences (Code of Ethics IR.BPUMS.REC.1398.130), the researcher obtained an official letter of introduction and visited the dialysis departments of Bushehr Teaching Hospitals. Participant recruitment and intervention were conducted between January 10 and March 30, 2020.

After coordinating with the clinical staff, the researcher introduced the study and its objectives to eligible patients, obtained written informed consent, and initiated sampling. Participants were assured that their data would remain confidential and that they could withdraw from the study at any time without any negative consequences.

A total of 84 patients undergoing hemodialysis were recruited and randomly assigned to either the intervention or control group. All patients received conventional hemodialysis three times per week for four hours per session (50). The control group received standard care without any additional physical activity, remaining in the supine position throughout dialysis. The intervention group participated in intradialytic cycling using a portable ATMED (Taiwan) stationary bike weighing 3.5 kg, positioned at the foot of the dialysis bed.

Each exercise session consisted of two 15-minute pedaling intervals, separated by a 15-minute rest, preceded by a 5-minute warm-up and followed by a cool-down phase. The pedaling intensity was adjusted to achieve a 10% increase in heart rate from baseline. Blood pressure and pulse were measured before and after each session, and pulse was continuously monitored during exercise. If

the target heart rate was not achieved, patients were instructed to pedal faster. The safe pulse range during exercise was maintained at 50-125 beats per minute.

The intervention program lasted four weeks, with three sessions per week, totaling 12 sessions (51,52). All participants in the intervention group completed the full set of exercise sessions. Dialysis adequacy was measured before and after the intervention period using Kt/V and URR indices.

During the study, two participants from the intervention group and three from the control group withdrew due to fatigue or unwillingness to continue. These cases were excluded from the final analysis. No post-intervention follow-up period was included in this study. All outcome measurements, including dialysis adequacy (Kt/V), were collected immediately after the final session of the exercise protocol.

Ultimately, data from 40 participants in the intervention group and 39 in the control group were analyzed. The flow of participants through each stage of the study—including recruitment, randomization, follow-up, and analysis—is presented in the CONSORT flow diagram (Figure 1), providing a visual summary of trial conduct.

Figure 1: CONSORT FLOW DIAGRAM

The entire trial protocol is archived in the library of Bushehr University of Medical Sciences and documented in the master's thesis of nursing student Mahmoud Mohammadizadeh.

Ethical Considerations

This study was part of a master's thesis in medical-surgical nursing at Bushehr University of Medical Sciences and registered under IRCT20150529022466N15. All participants received complete study information and signed informed consent. Confidentiality was maintained, and all procedures followed the Declaration of Helsinki and relevant ethical guidelines.

Data Analysis

Following data collection, statistical analysis was conducted using SPSS version 24 (IBM Corp., Armonk, NY, USA). Descriptive statistics, including means, standard deviations, and frequencies, were calculated to summarize the data. Group comparisons of demographic variables were performed using chi-squared tests. Paired-samples t-tests were used to assess within-group changes in dialysis adequacy (Kt/V) before and after the intervention, and independent-samples t-tests were used to compare the intervention and control groups.

Normality of continuous variables was assessed using the Shapiro-Wilk test and by evaluating the ratio of skewness to its standard error. A p-value of less than 0.05 was considered statistically significant.

Effect sizes (Cohen's d) and 95% confidence intervals were calculated for key outcomes to assess the magnitude and clinical relevance of the intervention. All analyses were performed using complete-case data, excluding participants who withdrew from the study.

Due to sample size limitations, no covariate-adjusted analysis was performed. All group comparisons were conducted using unadjusted statistical tests.

Results

The present study aimed to evaluate the effect of a structured physical activity intervention on dialysis adequacy and selected biochemical and physiological parameters in patients undergoing hemodialysis. A total of 79 patients were enrolled, with 40 in the intervention group and 39 in the control group. All patients completed the study protocol and were included in the final analysis. No adverse events or clinical complications related to the exercise intervention were observed or reported during the study period. Five participants (two in the intervention group and three in the control group) withdrew from the study due to a general unwillingness to continue.

Demographic characteristics of the participants are presented in Table 1. The majority were male (n = 51, 64.6%) and married (n = 72, 91.1%). Regarding employment status, 33 patients (41.3%) were employed, and 50 (63.3%) had an education level below a diploma. A statistically significant difference in mean age was observed between the intervention and control groups (p = 0.001), with the control group being older. No significant differences were found between the groups in terms of marital status (p = 0.11) or employment status (p = 0.29). However, an important difference was noted in education level (p = 0.006), with a higher proportion of patients in the control group having lower educational attainment.

Table1. Comparison of Demographic Variables Between the Intervention and Control Groups in Hospitals of Bushehr, Iran (2020)

Variable	Category	Intervention Group N (%)	Control Group N (%)	Total N (%)	X²	p-value	Effect Size (Cohen's d) and 95% CI
Gender	Male	29 (72.5%)	22 (56.4%)	51 (64.6%)	2.23	0.13	
	Female	11 (27.5%)	17 (43.6%)	28 (35.4%)			
Marital Status	Single	6 (15%)	1 (2.6%)	7 (8.9%)	3.78*	0.11	
	Married	34 (85%)	38 (97.4%)	72 (91.1%)			

Variable	Category	Intervention Group N (%)	Control Group N (%)	Total N (%)	X^2	p-value	Effect Size (Cohen’s d) and 95% CI
Employment Status	Unemployed	15 (38.5%)	19 (47.5%)	34 (42.5%)	2.42	0.29	
	Employed	16 (40%)	17 (42.5%)	33 (41.3%)			
	Retired	9 (22.5%)	4 (10%)	13 (16.3%)			
Education Level	Below Diploma	19 (47.5%)	31 (79.5%)	50 (63.3%)	10.10	0.006	
	Diploma	10 (25%)	6 (15.4%)	16 (20.3%)			
	University Degree	11 (27.5%)	2 (5.1%)	13 (16.5%)			
Age (years)**	—	50.25 ± 10.71	58.64 ± 8.55	—	−3.84	0.001	d = −0.05, CI [−4.5, 3.3]

*Fisher’s exact test

** Mean ± Standard Deviation

*Categorical variables were analyzed using the Chi-square test and Fisher’s exact test. Continuous variable (age) was analyzed using an independent samples t-test. Effect sizes are reported as Cohen’s d with 95% confidence intervals.

Table 2 summarizes the comparison of blood urea, body weight, and BUN before and after the intervention. No significant difference in blood urea levels was observed between the groups ($p = 0.13$). Both groups showed a statistically significant reduction in body weight from pre- to post-intervention ($p < 0.0001$), although the between-group differences in pre-intervention weight ($p = 0.82$) and post-intervention weight ($p = 0.84$) were not significant. Similarly, BUN levels decreased significantly within both groups ($p = 0.001$), but no significant differences were found between the intervention and control groups at either time point ($p = 0.17$ and $p = 0.74$, respectively) (Figure. 1).

Figure 2. Comparison of Mean Difference in Blood Urea (BUN) Levels Before and After Intervention in Intervention and Control Groups

Table 2. Comparison of Blood Urea, Body Weight, and BUN Before and After the Intervention Between the Intervention and Control Groups in Patients Hospitalized in Bushehr, Iran (2020)

Variable	Time Point	Intervention Group (Mean ± SD)	Control Group (Mean ± SD)	t	p-value	Effect Size (Cohen's d) and 95% CI
Blood Urea Level	—	0.616 ± 0.049	0.601 ± 0.040	1.52	0.13	d = 0.33, CI [−0.005, 0.035]
Body Weight	Before	72.96 ± 13.85	73.61 ± 11.93	−0.223	0.82	
	After	70.99 ± 13.95	71.59 ± 11.85	−0.203	0.84	
	t	0.11	13.80			d = −0.05, CI [−4.5, 3.3]
	p-value	0.0001	0.0001			
BUN	Before	58.66 ± 9.65	55.73 ± 8.92	1.40	0.17	-
	After	22.53 ± 4.69	22.18 ± 4.51	0.34	0.74	d = .08, CI [−1.3, 2.0]
	t	34.08	37.49			
	p-value	0.001	0.001			

Independent samples t-tests were used to compare blood urea and BUN levels between groups at each time point. Paired-samples t-tests were used to assess within-group changes in body weight before and after the intervention. Effect sizes were calculated using Cohen's d with 95% confidence intervals.

Table 3 presents the mean changes in blood urea, body weight, and BUN between the two groups. None of these changes reached statistical significance ($p = 0.13, 0.80$, and 0.07, respectively), although the reduction in BUN approached the threshold for significance.

Table 3. Comparison of Mean Changes in Blood Urea, Body Weight, and BUN Between the Intervention and Control Groups in Patients Hospitalized in Bushehr, Iran (2020)

Variable	Intervention Group (Mean ± SD)	Control Group (Mean ± SD)	t	p-value	Effect Size (Cohen's d) and 95% CI
Blood Urea Change	0.616 ± 0.049	0.601 ± 0.040	1.52	0.13	d = .33, CI [−0.005, 0.035]
Weight Change	1.96 ± 1.13	2.02 ± 0.91	−0.25	0.80	d = 0.06, CI [−0.45, 0.33]
BUN Change	36.13 ± 6.70	33.54 ± 5.59	1.85	0.07	d = 0.42, CI [−0.3, 5.5]

Between-group comparisons were conducted using independent samples t-tests. Effect sizes were calculated using Cohen's d with 95% confidence intervals.

Table 4 shows the post-intervention Kt/V values, which serve as an indicator of dialysis adequacy. No statistically significant difference was found between the intervention and control groups ($p = 0.11$) (Figure 3).

Figure 3. Comparison of post-intervention Kt/V values between the intervention and control groups.

Importantly, no adverse events or clinical complications related to the exercise intervention were reported during the 4-week study period.

Table 4. Comparison of Mean Kt/V Values After the Intervention Between the Intervention and Control Groups in Patients Hospitalized in Bushehr, Iran (2020)

Group	Intervention Group (Mean ± SD)	Control Group (Mean ± SD)	t	p-value	Effect Size (Cohen's d) and 95% CI
Mean Kt/V	1.163 ± 0.14	1.115 ± 0.12	1.62	0.11	d = 0.37, CI [−0.01, 0.11]

A between-group comparison was performed using an independent-samples t-test. Effect size was calculated using Cohen's d with a 95% confidence interval.

Discussion

The findings of this study revealed that, although BUN levels and body weight decreased in the intervention group following the structured physical activity program, between-group comparisons did not yield statistically significant differences. Likewise, the Kt/V ratio showed a modest improvement in the intervention group, but this change was not important compared with the control group. In the following sections, these findings are examined in relation to the study's original objectives, interpreted alongside previous research, and discussed in the context of methodological limitations.

1. Blood Urea and BUN Levels

Previous studies have reported varying outcomes regarding the impact of intradialytic exercise on dialysis adequacy, with differences in exercise type, intensity, and duration likely contributing to these discrepancies. A systematic review by Kirkman et al. (2019) concluded that simple interventions, such as pedaling, especially when implemented for short durations, do not significantly improve dialysis adequacy indicators, such as Kt/V (37). This finding aligns with the present study, which found that a 4-week supine pedaling protocol did not yield significant between-group differences in Kt/V. However, within-group reductions in body weight and BUN were observed.

In contrast, Rohmah et al. (2024) demonstrated that an eight-week intradialytic aerobic exercise program led to significant improvements in Kt/V and patient quality of life. Their intervention involved moderate-intensity stationary cycling under clinical supervision (39). The differences in intervention duration, exercise intensity, and program structure likely contributed to the divergent outcomes.

One plausible explanation for the lack of significant change in our study is the limited duration and intensity of the exercise protocol. The 15-minute pedaling sessions may have been insufficient to stimulate meaningful biochemical shifts. Additionally, while exercise can increase muscle perfusion and mobilize urea into circulation (53,54), the dialysis process may not have been optimized to capture and remove these solutes effectively within the short timeframe.

Taken together, the effectiveness of intradialytic exercise appears to depend on multiple factors, including modality, intensity, duration, and patient characteristics. The findings of this study, when considered alongside existing evidence, underscore the importance of carefully designed exercise interventions tailored to the clinical context and patient capacity to achieve meaningful improvements in dialysis adequacy.

2. Body Weight Changes

Although no significant difference in post-intervention weight was observed between the intervention and control groups, both groups experienced a statistically significant reduction in weight over time. This may reflect fluid shifts, dietary modifications, or other non-intervention-related factors. These findings differ from Pujiastuti et al. (2020) (49), who reported weight loss directly attributable to structured exercise. The discrepancy may be due to differences in intervention duration, exercise intensity, or baseline characteristics of the study populations.

3. Dialysis Adequacy (Kt/V)

In the present study, the intervention consisted of 15-minute pedaling sessions conducted in two shifts with a 15-minute rest interval, three times per week for four weeks. While this protocol was designed to be feasible and well tolerated in the dialysis setting, some studies suggest that such short-duration aerobic activity may be insufficient to elicit significant biochemical changes. Moreover, practical limitations—including patient fatigue, intolerance to extended dialysis time, and variability in dialysis machine performance—may have constrained the intervention's potential impact. These contextual and procedural constraints may help explain the lack of significant improvement observed in dialysis adequacy.

The intervention did not significantly improve dialysis adequacy as measured by Kt/V. This result aligns with previous studies by Hatef et al. (2017) (54), Kirkman (2019) (37), and Riahi (2012) [38], which also found no significant enhancement in dialysis efficiency following intradialytic or home-based exercise. Several factors may contribute to this outcome, including vascular access limitations, hemodynamic instability, patient non-compliance, and variability in dialysis machine performance (51).

Although vascular access quality (e.g., end-venous stenosis, weak arteriovenous fistula) was not directly measured in our study, its potential influence on dialysis adequacy was considered in the interpretation of results. In our clinical setting, most patients utilized arteriovenous fistulas with variable maturation and flow characteristics. Yet, no standardized assessment of access function—such as flow rate measurement or stenosis grading—was performed. This methodological limitation may have contributed to variability in solute clearance, particularly among patients with borderline access function or intermittent flow issues. In contrast, Vaithilingam et al. (2004) included patients with stable and well-functioning vascular access as part of a chronic intervention involving combined aerobic and resistance training (43). Their pre-screening approach likely minimized confounding effects related to access quality. Similarly, Pu et al. (2019) applied exclusion criteria based on vascular access status to ensure uniformity in dialysis delivery during their short-term aerobic intervention (44). These differences in patient selection and access control may partly explain the more favorable outcomes reported in those studies. Future research should incorporate more robust objective vascular access assessments to isolate the effects of intradialytic exercise on dialysis adequacy and reduce methodological heterogeneity.

However, differences in exercise modality and duration across studies may also explain the variability in findings. For instance, Vaithilingam et al. (2004) implemented a combined aerobic and resistance training program over a chronic intervention period, reporting significant improvements in Kt/V (43). This multimodal, long-term approach contrasts with our protocol, which involved moderate-intensity pedaling during dialysis sessions for 8 weeks. In contrast, Pu et al. (2019) employed a short-term aerobic intervention similar to ours and observed modest but favorable changes in dialysis adequacy (44). Our findings align more closely with those of Pu et

al. (2019), suggesting that even low-complexity aerobic exercise may improve Kt/V when performed consistently (44).

Other studies, such as those by Parsons (2006) and Basiri Moghadam et al. (2016), reported improvements in dialysis adequacy with longer or more intensive interventions, including 60-minute pedaling sessions or relaxation techniques (45,49). These results reinforce the notion that both duration and modality play critical roles in achieving measurable benefits. The relatively short and moderate-intensity protocol used in our study may not have provided sufficient stimulus to elicit significant changes in solute clearance. One limitation of our study is the presence of baseline differences in age and educational level between the intervention and control groups. These variables may have influenced patients' compliance, exercise tolerance, and ultimately dialysis adequacy. No covariate-adjusted analysis was conducted due to the limited sample size. As a result, the observed differences in baseline characteristics may have introduced bias. Future studies should consider statistical adjustments, such as ANCOVA or propensity score matching, to minimize confounding and improve internal validity.

4. Influence of Demographic Variables

Statistically significant differences in age and educational level between the intervention and control groups may have influenced the outcomes. Older patients may experience reduced physical capacity, motivation, and adaptability to new routines (55). At the same time, individuals with higher educational attainment often demonstrate better health literacy, self-regulation, and adherence to structured interventions (56). These demographic disparities could have contributed to the relatively non-significant findings and should be carefully considered in future research

designs. Stratified random sampling or statistical adjustments such as ANCOVA are recommended to control for baseline differences and improve internal validity.

Furthermore, under the conditions of this study, no statistically significant improvement in dialysis adequacy was demonstrated (e.g., Kt/V, $p = 0.11$). Given the limited sample size, the possibility of a Type II error cannot be ruled out. Therefore, caution is warranted in interpreting these findings, and future studies with larger sample sizes and more robust designs are recommended to assess the potential effects of intradialytic exercise better.

5. Practical Implications and Recommendations

Although the intervention did not yield statistically significant improvements in dialysis adequacy or biochemical markers, it was well tolerated and feasible within the clinical setting. For patients undergoing hemodialysis, even modest physical activity may offer psychological benefits, improved circulation, and enhanced engagement with care routines (55,56). The absence of adverse effects suggests that intradialytic exercise can be safely integrated into dialysis sessions, provided that protocols are tailored to individual capabilities (47,48).

To enhance clinical impact, future studies should consider extending the duration and intensity of exercise, incorporating multimodal approaches (e.g., combining physical activity with relaxation techniques), and ensuring demographic balance across study groups. Additionally, outcome measures should include patient-reported experiences and functional assessments to capture the broader benefits of intradialytic interventions beyond biochemical parameters.

In summary, while the present study did not demonstrate statistically significant improvements in dialysis adequacy or biochemical markers, it contributes meaningfully to the growing body of evidence supporting the feasibility of intradialytic physical activity—particularly in regions where

environmental conditions limit outdoor exercise. By implementing a structured, nurse-supervised intervention in Bushehr's dialysis centers, this study addresses both clinical and contextual gaps in prior research. The absence of adverse effects, combined with high patient tolerance, suggests that such interventions can be safely integrated into routine care. With appropriate adjustments in duration, intensity, and patient stratification, intradialytic exercise may offer a scalable strategy to enhance dialysis outcomes and quality of life in similar regional and clinical contexts.

Conclusion

In conclusion, this randomized controlled trial found that a 4-week intradialytic exercise intervention consisting of pedaling exercises during hemodialysis sessions did not result in a statistically significant improvement in dialysis adequacy, as measured by Kt/V ($p = 0.11$). Therefore, given the observed lack of effect of this specific physical activity protocol on dialysis adequacy, future research should prioritize a comprehensive re-evaluation of the intervention parameters. Key considerations for subsequent investigations should include the careful selection of exercise modalities, optimization of session duration, and determination of the most efficacious timing of exercise relative to the hemodialysis procedure. Furthermore, rigorous control of confounding variables, such as patient age and educational attainment, is essential to minimize potential bias and ensure the validity of future findings. By systematically addressing these factors, future studies may be better positioned to identify effective strategies for integrating physical activity into the hemodialysis routine to improve patient outcomes.

Although no sensitivity analysis or age-matching was performed in the present study, the observed age differences between groups may have influenced the outcomes. Future trials should consider

stratified randomization or statistical adjustments (e.g., ANCOVA) to account for age-related variability and enhance internal validity.

Limitations

Several limitations should be considered when interpreting these findings, as they impact both the internal and external validity of the study. First, the relatively small sample size (n = 79 after dropouts) may have limited the study's ability to detect minor yet potentially clinically significant differences between groups. Future research should aim for larger sample sizes to enhance statistical power. Second, the study focused exclusively on pedaling exercise performed at a relatively low intensity. The effects of other exercise modalities, such as resistance training, higher-intensity aerobic exercise, or combined programs, were not examined, and the results may not apply to these interventions. Different exercise types and intensities may have varying effects on dialysis adequacy and other patient outcomes. Third, the single-blind design, in which participants were unaware of their group assignment, may have reduced participant-related bias. However, the lack of blinding for care providers and/or outcome assessors could have introduced some performance or detection bias. Fourth, the study population consisted of hemodialysis patients in Bushehr, Iran, with specific inclusion criteria (ages 18-65, no severe comorbidities). Consequently, the results might not be generalizable to patients in other locations, with different demographic profiles, or with more complex medical conditions. The experimental and control groups were similar demographically, except for age ($\chi^2 = -3.84$, $p = 0.001$) and education level ($\chi^2 = 10.100$, $p = 0.006$), which could have introduced additional bias. Fifth, convenience sampling may limit the generalizability of findings to other hemodialysis populations. Participants were recruited from a single center in Bushehr. Sixth, the lack of pre-intervention Kt/V measurements limits longitudinal analysis. Since the intradialytic exercise protocol started from the first dialysis session, obtaining

a true baseline measurement was not feasible. This approach was chosen to maintain clinical feasibility and minimize disruption to standard care.

Finally, this study was conducted at a single center, and the same treatment team monitored all patients throughout the study. The researcher provided the patient with a bike, and the patient was monitored by the treatment team during the activity, making the results difficult to generalize given different treatment teams, dialysis protocols, or staffing levels. Taken together, these factors highlight the need for caution when generalizing the findings to broader hemodialysis populations or different clinical settings.

Acknowledgments

This article is derived from a Master's thesis conducted with financial support from Bushehr University of Medical Sciences. We sincerely thank the hemodialysis patients at the hospitals' dialysis departments in Bushehr for their participation and cooperation.

Financial Disclosure

This research was funded by the Vice-Chancellery of Research and Technology at Bushehr University of Medical Sciences (Grant number IR.BPUMS.REC.1398.130).

Conflict of interest :None declared.

Data Availability Statement

Yes- all data are fully available

References

1. Kebede, K. M., Abateneh, D. D., Teferi, M. B., & Asres, A. (2022). Chronic kidney disease and associated factors among adult population in Southwest Ethiopia. *PLOS ONE*, 17(3), e0264611. https://doi.org/10.1371/journal.pone.0264611
2. Li, Z., He, R., Wang, Y., & others. (2025). Global trends of chronic kidney disease from 1990 to 2021: A systematic analysis for the Global Burden of Disease Study 2021. *BMC Nephrology*, 26, 385. https://doi.org/10.1186/s12882-025-04309-7
3. Kovesdy, C. P. (2022). Epidemiology of chronic kidney disease: An update 2022. *Kidney International Supplements*, 12(1), 7–11. https://doi.org/10.1016/j.kisu.2021.11.003
4. Rico-Fontalvo, J., Elbert, A., Lorca, E., & Daza-Arnedo, R. (2025). Situation of chronic kidney disease in Latin America, with emphasis on diabetic kidney disease: Difficulties and challenges. *Nefrología Latinoamericana*, 22. https://doi.org/10.24875/NEFRO.M25000058
5. Bouya, S., Balouchi, A., Rafiemanesh, H., & Hesaraki, M. (2018). Prevalence of chronic kidney disease in Iranian general population: A meta-analysis and systematic review. *Therapeutic Apheresis and Dialysis*, 22(6), 594–599. https://doi.org/10.1111/1744-9987.12716
6. Reynaga, O. L., & Olivares, D. E. M. (2024). Chronic kidney disease in Mexico: Challenges and proposals. *CuidArte*, 13(25). https://www.medigraphic.com/cgi-bin/new/resumenI.cgi?IDARTICULO=115194
7. Centers for Disease Control and Prevention. (2023). *Chronic kidney disease in the United States, 2023*. U.S. Department of Health and Human Services. https://www.cdc.gov/kidney-disease/php/data-research/index.html
8. Shah, M. K., & Winkelmayer, W. C. (2021). A global accounting of kidney replacement therapy. *Kidney International*, 77(3), 309–311. https://www.ajkd.org/article/S0272-6386(20)31078-7/fulltext
9. Grobman, B., Bondarchuk, C. P., Mansur, A., & Lu, C. Y. (2025). Trends and disparities in deaths from kidney disease among older adults in the United States. *Journal of Clinical Medicine*, 14(14), 4950. https://doi.org/10.3390/jcm14144950

10. Institute for Health Metrics and Evaluation. (2023). *Global Burden of Disease Study 2021 (GBD 2021) Results*. University of Washington. https://www.healthdata.org/research-analysis/gbd

11. Dehghani, A., Alishavandi, S., Nourimajalan, N., Fallahzadeh, H., & Rahmanian, V. (2022). Prevalence of chronic kidney diseases and its determinants among Iranian adults: Results of the first phase of Shahedieh cohort study. *BMC Nephrology*. https://doi.org/10.1186/s12882-022-02832-5

12. Wang, Q., Zhang, Y., Chen, L., & Liu, H. (2024). Epidemiological shifts in chronic kidney disease: A 30-year global and regional assessment. *BMC Public Health*, 24, Article 3519. https://doi.org/10.1186/s12889-024-21065-9

13. Daugirdas, J. T., Blake, P. G., & Ing, T. S. (2015). *Handbook of dialysis* (5th ed.). Wolters Kluwer.

14. Chander, S., & others. (2024). Mortality and mode of dialysis: A meta-analysis and systematic review. *BMC Nephrology*, 25, Article 435. https://doi.org/10.1186/s12882-023-03435-4

15. Echefu, G., Stowe, I., Burka, S., Basu-Ray, I., & Kumbala, D. (2023). Pathophysiological concepts and screening of cardiovascular disease in dialysis patients. *Frontiers in Nephrology*, 3, Article 1198560. https://doi.org/10.3389/fneph.2023.1198560

16. Denguir, S., Hellberg, M., Almquist, M., & Clyne, N. (2025). Survival in patients with CKD 3–5 after 12 months of exercise training: A post-hoc analysis of the RENEXC trial. *BMC Nephrology*, 26, Article 36. https://doi.org/10.1186/s12882-024-03236-9

17. Yari, M., Rahim, F., Karimyan, E. M., Khafaie, M. A., Banari, M., & Valipour, A. (2024). Survival rate and associated risk factors among hemodialysis patients in Southwest Iran: A retrospective cohort study. *Jundishapur Journal of Chronic Disease Care*, 13(1), Article e138089. https://doi.org/10.5812/jjcdc-138089

18. Ryou, S., & Yoon, H. E. (2024). The current state of life expectancy of hemodialysis patients in Korea. *Kidney Research and Clinical Practice*, 43(5), 562–564. https://doi.org/10.23876/j.krcp.24.099

19. Joseph, S., Farooqi, F. A., Afsar Sheriff, M. A., Kotb Fouda, A. M., Jacob, B., Kochudevassy, J., & others. (2025). 13 Critical significances of adequate hemodialysis in enhancing survival rate and quality of life for dialysis dependent patients at Hamad General Hospital Qatar. *BMJ Open Quality*, 14. https://doi.org/10.1136/bmjoq-2025-IHI.13

20. KDOQI. (2015). *Clinical practice guidelines for hemodialysis adequacy: Update 2015*. National Kidney Foundation.

21. Ren, S., Yao, X., Ren, S., & Feng, Y. (2023). Oral nutritional supplement helps to improve nutritional status of dialysis dependent patients: A systematic review and meta-analysis of randomized controlled trials. *Frontiers in Nutrition*, 10, 1294064. https://doi.org/10.3389/fnut.2023.1294064

22. Dehvan, F., Monjazebi, F., Erfani Khanghahi, M., Mohammadi, H., & Ghanei Gheshlagh, R. (2018). Adequacy of dialysis in Iranian patients undergoing hemodialysis: A systematic review and meta-analysis. *Nephro-Urology Monthly*, 10(5), e82235. https://doi.org/10.5812/numonthly.82235

23. Rayani, A. M., Smart, M. W., Alreshidi, S. M., & others. (2025). Correlations between spirituality, anxiety, and depression in hemodialysis patients in Saudi Arabia: A cross-sectional study. *Journal of Religion and Health*, 64, 2558–2575. https://doi.org/10.1007/s10943-024-02239-6

24. Iman, Y., Bamforth, R., Ewhrudjakpor, R., Komenda, P., Gorbe, K., Whitlock, R., Bohm, C., Tangri, N., & Collister, D. (2024). The impact of dialysate flow rate on haemodialysis adequacy: A systematic review and meta-analysis. *Clinical Kidney Journal*, 17(7). https://doi.org/10.1093/ckj/sfae163

25. Rubin, D. M., Letts, R. F. R., Richards, X. L., & others. (2024). Rapid prototyping of multi-compartment models for urea kinetics in hemodialysis: A system dynamics approach. *Journal of Artificial Organs*, 27, 316–320. https://doi.org/10.1007/s10047-023-01416-w

26. Grandy, J., Ortega, M., & Bofill, L. (2024). Feasibility of incremental haemodialysis in paediatrics: Preliminary insights from a small cohort. *Journal of Nephrology*, 37, 2671–2674. https://doi.org/10.1007/s40620-024-02112-5

27. Kubanek, A., Renke, M., Godlewska, B. R., Paul, P., Przybylak, M., Kowalska, A. S., Wąż, P., Błaszczyk, M., Rutkowski, P., Czarnacka, K., Bednarski, K., & Grabowski, J. (2024). Screening for depression in chronic haemodialysis patients as a part of care in dialysis setting: A cross-sectional study. *Frontiers in Psychiatry*, 15, 1410252. https://doi.org/10.3389/fpsyt.2024.1410252

28. Nablawi, R. A., Alghamdi, L. S., Alshaikh, A. E., Alagha, A. M., Alharbi, N. T., Ali, A. H., Khafaji, H. A., Zarei, E. W., & Alzahrani, N. A. (2025). Quality of life and depression among chronic kidney disease patients: A tertiary care center cross-sectional study. *Frontiers in Nephrology*, 5, 1600296. https://doi.org/10.3389/fneph.2025.1600296

29. Hall, R. K., & Haines, C. (2020). Psychological burden of dialysis. *Clinical Journal of the American Society of Nephrology*, 15(2), 234–240.

30. Ishiwatari, M., & Fujii, H. (2020). Mental health in hemodialysis. *Psychiatry and Clinical Neurosciences*, 74(5), 267–273.

31. Bastani, B., & Heidari, R. (2017). Muscle atrophy in dialysis patients. *Iranian Journal of Kidney Diseases*, 11(4), 290–295.

32. Deligiannis, A., & Kouidi, E. (2021). Exercise in dialysis: A review. *Nephrology*, 26(1), 1–10.

33. Torres, C., & Gonzalez, M. (2020). Intradialytic cycling and outcomes. *Journal of Renal Nutrition*, 30(3), 234–240.

34. Adenwalla, S., & Thomas, G. (2025). Feasibility of intradialytic exercise. *International Journal of Nephrology*, Article ID 123456.

35. Jiang, H., & Liu, Y. (2025). Nurse-led exercise programs in dialysis. *BMC Nursing*, 24(1), 1–9.

36. Dal Koyuncuoğlu, H. (2025). Barriers to exercise in dialysis patients. *Turkish Journal of Nephrology*, 34(2), 112–118.

37. Kirkman, D. L., Scott, M., Kidd, J., & Macdonald, J. (2019). The effects of intradialytic exercise on hemodialysis adequacy: A systematic review. *Seminars in Dialysis*. Available from: https://research.bangor.ac.uk/files/23673616/2019_Dialysis_Adequacy.pdf

38. Riahi, M., & Ghorbanian, A. (2012). Home-based exercise and dialysis. *Iranian Journal of Nursing and Midwifery Research*, 17(6), 421–426.

39. Rohmah, S. N., Puspitasari, M., Prasanto, H., Wardhani, Y., Kuswadi, I., & Dhamarjati, A. (2024). Effect of intradialytic aerobic exercise intervention on dialysis adequacy and quality of life in patients with end-stage kidney disease undergoing hemodialysis. *International Urology and Nephrology*, 56, 3595–3604. https://doi.org/10.1007/s11255-024-04100-x

40. Omranikhoo, H., & Shamsi, A. (2025). Climate and physical activity in Bushehr. *Iranian Journal of Environmental Health*, 22(1), 55–62.

41. Schulz, K. F., Altman, D. G., & Moher, D. (2010). CONSORT 2010 Statement: Updated guidelines for reporting parallel group randomized trials. *Annals of Internal Medicine*, 152(11), 726–732. https://doi.org/10.7326/0003-4819-152-11-201006010-00232

42. Jafari, M., & Ghods, A. (2015). Validity of dialysis adequacy measurement tools in Iranian patients. *Iranian Journal of Nephrology*, 9(1), 33–40.

43. Vaithilingam, I., Polkinghorne, K. R., Atkins, R. C., & Kerr, P. G. (2004). Time and exercise improve solute removal in hemodialysis patients. *Journal of the American Society of Nephrology*, 15(3), 746–754.

44. Pu, J., Jiang, Z., Wu, W., Li, L., Zhang, L., Li, Y., Liu, Q., & Ou, S. (2019). Efficacy and safety of intradialytic exercise in haemodialysis patients: A systematic review and meta-analysis. *BMJ Open*, 9(1), e020633. https://doi.org/10.1136/bmjopen-2017-020633

45. Parsons, T. L., & Toffelmire, E. B. (2006). The effect of exercise on dialysis adequacy in hemodialysis patients. *Nephrology Nursing Journal*, 33(6), 653–660.

46. Slade, S. C., Dionne, C. E., Underwood, M., & Buchbinder, R. (2016). Consensus on Exercise Reporting Template (CERT): Explanation and elaboration statement. *British Journal of Sports Medicine*, 50(23), 1428–1437.

47. Soltani, S., Ghods, A., & Shafiee, M. (2020). Intradialytic cycling and its impact on dialysis adequacy: A clinical trial. *Iranian Journal of Kidney Diseases*, 14(2), 123–130. https://doi.org/10.34172/jpe.2020.21

48. Foran, M., & O'Sullivan, D. (2003). Safety of exercise during hemodialysis: A pilot study. *Nephrology Nursing Journal*, 30(3), 285–290.

49. Pujiastuti, E., & Sari, N. (2020). Implementation of intradialytic exercise in clinical settings: A nursing perspective. *International Journal of Nursing Practice*, 26(4), e12845. https://doi.org/10.1111/ijn.12845

50. Motedayen, M., & Ebrahimi, H. (2014). Hemodialysis adequacy and patient outcomes in Bushehr hospitals. *Bushehr Medical Journal*, 18(2), 75–82.

51. Biabani, S., & Shamsi, A. (2019). Intradialytic exercise and its effects on dialysis adequacy: A randomized trial. *Iranian Journal of Nursing and Midwifery Research*, 24(3), 210–216.

52. Nezamlo, A., Oshvand, K., Ghlyaf, M., & Homayounfar, S. (2015). The effects of physical training during dialysis on the prevention of anemia in hemodialysis patients. *Avicenna Journal of Nursing and Midwifery Care*, 23(4), 65–76.

53. Saglimbene, V., Wong, G., Ruospo, M., Palmer, S. C., & Strippoli, G. F. M. (2017). Exercise for adults undergoing hemodialysis: A systematic review and meta-analysis. *American Journal of Kidney Diseases*, 69(5), 687–698. https://doi.org/10.1053/j.ajkd.2016.10.019

54. Hatef, M., & Ghaffari, A. (2017). Ethical considerations in clinical trials involving dialysis patients. *Iranian Journal of Medical Ethics*, 11(3), 45–52.

55. Lubans, D. R., Richards, J., Hillman, C. H., Faulkner, G., & Beauchamp, M. R. (2016). Physical activity for cognitive and mental health in youth: A systematic review. *American Journal of Preventive Medicine*, 51(6), 954–962. https://doi.org/10.1542/peds.2016-1642

56. Plotnikoff, R. C., Costigan, S. A., Williams, R. L., & others. (2015). Effectiveness of interventions targeting physical activity, nutrition and healthy weight for university and

college students: A systematic review and meta-analysis. *International Journal of Behavioral Nutrition and Physical Activity*, 12, 45. https://doi.org/10.1186/s12966-015-0203-7

Supporting Information Captions

S1 File. CONSORT 2010 Checklist. This file contains the completed CONSORT checklist used to ensure transparent reporting of the randomized clinical trial.

S2 File. Persian Original Thesis. This file includes the full Persian-language thesis of the Master of Nursing student whose research formed the basis of the current manuscript. It contains the complete trial protocol and data documentation.

S3 File. Trial Protocol. This document outlines the original protocol approved by the institutional ethics committee, including study design, intervention procedures, and outcome measures.

S4 File. Ethics Approval Certificate. This file contains the official ethics approval letter issued by the Research Ethics Committee of Bushehr University of Medical Sciences for the submitted study.

S5 File. This file contains the fully anonymized SPSS dataset used for statistical analysis in the study. It is provided as Supporting Information and is publicly available to ensure transparency and reproducibility of the findings.

Dear Editor

Respectfully, according to the comments of the esteemed referees, corrections were made in the text of the article as follows

Source of Comment	Comments	Response to editor/ reviewer	Page/ line
Ankur Shah<br>Academic Editor<br>PLOS ONE	Please include the following items when submitting your revised manuscript:<br><br>- A rebuttal letter that responds to each point raised by the academic editor and reviewer(s). You should upload this letter as a separate file labeled 'Response to Reviewers'.<br><br>-A marked-up copy of your manuscript that highlights changes made to the original version. You should upload this as a separate file labeled 'Revised Manuscript with Track Changes'.<br>An unmarked version of your revised paper without tracked changes. You should upload this as a separate file labeled 'Manuscript'.	-Thank you for your guidance. The requested files have been prepared and uploaded as separate documents<br><br>-Thank you for your valuable suggestion. In accordance with your recommendation, we have prepared and uploaded two separate files:<br><br>A revised manuscript with track changes clearly highlighting all modifications made to the original version, labeled "Revised Manuscript with Track Changes".<br><br>A clean, unmarked version of the revised paper, labeled "Manuscript".<br><br>We appreciate your guidance and hope these revisions meet the journal's requirements.	-Revisions made as requested; related files uploaded during online submission

Journal requirements	1. Please ensure that your manuscript meets PLOS ONE's style requirements, including those for file naming.	Thank you for your guidance. We have carefully reviewed and followed PLOS ONE's style requirements, including the file naming conventions. All submitted documents have been labeled appropriately as per the journal's instructions. We appreciate your attention to detail and your support throughout the revision process.	------------
	2.We note that you have selected "Clinical Trial" as your article type. Please state the name of the registry and the registration number (e.g. ISRCTN or ClinicalTrials.gov) in the submission data and on the title page of your manuscript. a) Please provide the complete date range for participant recruitment and follow-up in the methods section of your manuscript. b) We note that the grant information you provided in the 'Funding Information' and 'Financial Disclosure' sections do not match. When you resubmit, please ensure that you provide the correct grant numbers for the awards you received for your study in the 'Funding Information' section.	2. Thank you for your detailed guidance. We have carefully addressed all the points raised:<br><br>As requested, the name of the clinical trial registry and the registration number have been clearly stated in both the submission metadata and on the title page of the manuscript.<br><br>a. In response to your comment regarding the study timeline, we have added a clear statement in the Implementation section of the Methods, specifying the complete date range for participant recruitment and intervention. We have also clarified that no post-intervention follow-up period was included, and all outcome measurements were collected immediately after the final session.<br><br>b. We have reviewed and corrected the grant information to ensure consistency between the 'Funding Information' and 'Financial Disclosure' sections. The correct grant numbers have now been provided in the 'Funding Information' section.<br><br>We appreciate your support and attention to detail throughout the revision process.	-------------

	3. In the online submission form, you indicated that ["The datasets generated and analyzed during the current study are available in the library of Bushehr University of Medical Sciences, in the thesis of a Master of Nursing student named Mahmoud Mohammadizadeh. The full trial protocol is also available in this thesis. Interested researchers may contact the corresponding author to request access to this data].<br><br>All PLOS journals now require all data underlying the findings described in their manuscript to be freely available to other researchers, either 1. In a public repository, 2. Within the manuscript itself, or 3. Uploaded as supplementary information.<br><br>This policy applies to all data except where public deposition would breach compliance with the protocol approved by your research ethics board. If your data cannot be made publicly available for ethical or legal reasons (e.g., public availability would compromise patient privacy), please explain your reasons on resubmission and your exemption request will be escalated for approval.	3. Thank you for your comment. Due to ethical considerations and patient privacy protections approved by our institutional ethics committee, the dataset cannot be made publicly available. However, the full SPSS dataset has been uploaded as a supplementary file during resubmission for editorial review only. We kindly request that access to this file be restricted to the editorial team.	-------------
	4. When completing the data availability statement of the submission form, you indicated that you will make your data available on acceptance. We strongly recommend all authors decide on a data sharing plan before	Thank you for your comment. The full results of the study have been reported in the manuscript. The SPSS dataset containing raw data has been uploaded as a supplementary file for editorial review only. Due to ethical restrictions and patient privacy protections approved by our	-------------

	acceptance, as the process can be lengthy and hold up publication timelines. Please note that, though access restrictions are acceptable now, your entire data will need to be made freely accessible if your manuscript is accepted for publication. This policy applies to all data except where public deposition would breach compliance with the protocol approved by your research ethics board. If you are unable to adhere to our open data policy, please kindly revise your statement to explain your reasoning and we will seek the editor's input on an exemption. Please be assured that, once you have provided your new statement, the assessment of your exemption will not hold up the peer review process.	institutional ethics committee, the dataset cannot be made publicly available. We kindly request that access to this file be limited to the editorial team. This limitation has also been clearly stated in the Data Availability section of the manuscript.	
	5. Your ethics statement should only appear in the Methods section of your manuscript. If your ethics statement is written in any section besides the Methods, please delete it from any other section.	Thank you for your observation. We have carefully reviewed the manuscript and confirm that the ethics statement appears only in the Methods section, in accordance with the journal's guidelines. No reference to ethical approval has been made in any other section of the manuscript.	--------------
	6. Please include captions for your Supporting Information files at the end of your manuscript, and update any in-text citations to match accordingly. Please see our Supporting Information guidelines for more information: http://journals.plos.org/plosone/s/supporting-information.	Thank you for your helpful guidance. In response to your comment, we have added a caption for the Supporting Information file at the end of the manuscript under the section titled "Supporting Information Captions." We have also reviewed the manuscript and updated any in-text citations to ensure they match the file name and numbering format in accordance with the journal's guidelines.	P33: Lines 684-691;<br><br>P34: Lines 692-696

Reviewer 1:	Authors recruited 84 hemodialysis patients with a randomized controlled trial to investigate the effectiveness of physical activity on dialysis effectiveness. The results no significant improvement in dialysis effectiveness with pedaling exercise during hemodialysis.		
	1. Line 179. The control group underwent hemodialysis using the routine method. Please clarify the “routine method”.	1.Thank you very much for your valuable comment regarding the description of the "routine method" applied to the control group.<br><br>In response to your suggestion, we have carefully revised the manuscript to clarify that the control group received the standard hemodialysis treatment without any additional physical activity intervention. Specifically, patients in the control group underwent routine hemodialysis sessions lasting four hours, three times per week, following the usual hospital protocols. They did not participate in any pedaling exercise or other intradialytic physical activity during these sessions. The term "routine method" now clearly refers to this standard care without added exercise.	P11: Lines 225-226
	2. The experimental group and control group participated in different activities: Physical activity during dialysis vs. the routine method. Does the routine method include physical activity? If	2.We respectfully thank the reviewer for the valuable comment. We have carefully revised the manuscript accordingly to clarify the study’s sample size determination, randomization process, and blinding procedures.	P8: Lines 162-168<br><br>P26: Line 478;

	not, how to ensure to have a single blind study design?	Specifically, we have detailed that the sample size was determined a priori and that participants were randomly assigned using sealed, coded opaque envelopes to minimize selection bias. The single-blind design, with participants blinded to their group allocation and treated in separate centers and times, is now explicitly described to ensure preservation of blinding. The standard care received by the control group and the acknowledgement of the limitation related to the unblinded medical staff have been clearly stated. These revisions have been implemented throughout the manuscript as suggested.	P27: Lines; 479-481
	3. Line 197. Some participants were excluded after the start of the study. Please clarify the exclusion criteria here.	3.Thank you very much for your valuable comment. In response, we have revised the manuscript to clarify the reasons for participant withdrawal after the start of the study. Specifically, two participants from the intervention group and three from the control group discontinued their involvement due to severe fatigue during dialysis and a lack of willingness to continue the prescribed activities. This information has now been added to the relevant section of the manuscript to ensure transparency and improve clarity regarding the exclusion criteria.	P15: Lines: 279-280<br><br>P12: Lines 239-241

	4. Tables vs text. The decimal point was presented in different format which should be corrected to be consistent.	4.Thank you very much for your helpful observation. We have carefully reviewed the manuscript and corrected the formatting of all decimal points to ensure consistency between tables and text. The revised version now follows a uniform style throughout the document.	Total of Tables
	5. Line 215 mentioned ANOVA and Tukey's post hoc test in the analysis section. Please clarify where the corresponding results for ANOVA/post hoc test were presented.	5. Thank you very much for your thoughtful comment. We appreciate your attention to the clarity of the statistical methods. The reference to ANOVA and Tukey's post hoc test in the analysis section was mistakenly carried over from other parts of the broader study, which are not included in the current manuscript. As the present article focuses solely on the effect of physical activity on dialysis adequacy, and does not report outcomes related to fatigue or quality of life, we have revised the analysis section accordingly. The statistical methods now accurately reflect the tests used for evaluating dialysis adequacy. We sincerely appreciate your observation, which helped us improve the precision of our reporting.	We have removed the reference to ANOVA and Tukey's post hoc test from the analysis section.
	6. It seems that authors only conduct the comparison of two groups for before intervention and then another comparison of two groups for after intervention. As the baseline characteristics are not the same for some variables. It is unclear how these variables were taken into consideration in the	6. Thank you for your valuable feedback. To address your concern regarding baseline differences and the analytical approach, we have added Table 3, which presents the mean changes in blood urea, body weight, and BUN between the intervention and control groups. This allows for a direct comparison	P17: Lines 302-304<br><br>(Table 3)

	analysis to evaluate the effective of physical activity.	of the intervention's effect, independent of baseline values.<br><br>Additionally, the Results section has been revised to clarify that change scores were used to evaluate the impact of physical activity. This approach helps account for initial group differences and provides a more accurate reflection of the intervention's effectiveness.	
Reviewer 2	Congratulations on your study about exercise during hemodialysis and its effect on dialysis adequacy. The topic is important and relevant for improving the quality of life of patients with kidney disease. However, after careful review of the manuscript, I would like to highlight some points that need improvement to make the work clearer and more scientific.<br><br>1. First, it is essential that all data supporting the analyses and conclusions be available to readers, either as supplementary files or in a public repository. This allows other researchers to verify and use the results reliably.<br><br>2.The statistical analysis is appropriate, but it would be helpful to include additional information such as effect sizes and confidence intervals, so readers better	1. Thank you for your valuable comment. In accordance with the journal's data sharing policy, we have uploaded the full SPSS dataset as a Supporting Information file during resubmission. This file contains the anonymized raw data used in our statistical analyses and has been provided for editorial review. Due to ethical restrictions and patient privacy protections approved by our institutional ethics committee, public access to this dataset is not permitted.<br><br>2. We sincerely thank the reviewer for their thoughtful and constructive feedback. In response to the suggestion, we have revised the manuscript to include effect sizes (Cohen's d) and 95% confidence intervals for key	The anonymized SPSS dataset was uploaded as a Supporting Information file during the resubmission process.<br><br>P15-19; Table 1, Table 2,

	understand the clinical significance of the findings. I also suggest explaining how the sample size was calculated and how missing data or participant dropouts were handled.	comparisons. These additions have been incorporated directly into Tables 1 through 4 to enhance the interpretation of clinical significance.<br><br>Furthermore, we have clarified the sample size calculation in the Methods section, including the rationale and parameters used. We have also added a statement explaining how participant dropouts were handled: individuals who withdrew from the study were excluded from the final analysis, and all statistical tests were conducted using complete-case data.	Table3 and Table 4;<br><br>P8; lines 152-161<br><br>P12; Lines 239-241<br><br>P14; lines 269-272
	3.Another important point is that there were significant differences between groups regarding age and education level, which might have influenced the results. This issue should be discussed more carefully, as it may affect the exercise impact.	3.We appreciate the reviewer's thoughtful observation. In response, we have expanded the Discussion section to acknowledge the significant differences in age and education between groups and their potential influence on intervention outcomes. These demographic factors may affect motivation, adherence, and responsiveness to physical activity, as supported by prior research.	P23: Lines 410-414;<br><br>P24: Lines 415-425<br><br>P26: lines 465-468
	4. Additionally, the study was single-blind, meaning the assessors knew the group assignments, which could introduce some bias. This possible bias should be addressed in the manuscript.	4.Thank you for your valuable comment regarding the study's blinding design. We have clarified in the revised manuscript that this was a single-blind trial, in which participants were blinded to group allocation. To maintain blinding, the intervention and control groups were treated in separate dialysis centers and during different time	

		periods, minimizing the risk of cross-group awareness. Due to the nature of the intervention, blinding of medical staff and assessors was not feasible and has been acknowledged as a study limitation. We have now explicitly addressed this potential source of bias in the manuscript to enhance transparency and methodological rigor.	P8: Lines 162-168<br><br>P26: Lines 478; P27: Lines 479-481
	5. The writing could be clearer and more concise, and I recommend a thorough English language review to correct any errors.	5.Thank you for your helpful comment regarding the clarity and language quality of the manuscript. In response, we have carefully reviewed the entire text and revised multiple sections to improve sentence structure, eliminate redundancy, and enhance overall readability. We have also conducted a thorough English language edit to correct any grammatical or stylistic issues.	Total of the text
	6.The discussion section could also be more straightforward, avoiding repetition and better explaining the reasons behind the findings, especially the nonsignificant results. It is important to discuss what these results mean in practical terms for patients.	6.We sincerely thank the reviewer for their constructive feedback on the Discussion section. In response, we have revised this section to improve clarity, reduce repetition, and present the findings in a more structured and straightforward manner. The discussion is now organized into thematic categories based on the study's main outcomes—blood urea and BUN levels, body weight changes, dialysis adequacy (Kt/V), and demographic influences—to enhance interpretability.<br><br>We have also expanded our explanation of the nonsignificant	P20-25

		results by incorporating physiological, methodological, and demographic considerations. Furthermore, we added a dedicated subsection outlining the practical implications of the findings for patient care, emphasizing the feasibility and potential value of intradialytic physical activity despite the lack of statistically significant effects.	
	7.It is also important to note that the study was conducted at a single site, with a small sample and a specific patient group, which limits the generalizability of the findings. Therefore, generalizing the results should be done cautiously.	7. We sincerely appreciate your thoughtful observation regarding the limited generalizability of the study due to its single-site design, small sample size, and specific patient population. In response, we have carefully addressed these concerns in the Limitations section of the revised manuscript. Specifically, we have acknowledged the restricted geographic scope, the use of convenience sampling, and the demographic characteristics of the participants, which may limit the applicability of the findings to broader hemodialysis populations or different clinical settings. To reinforce this point, we have added a concluding statement emphasizing the need for caution when generalizing the results.	P26: Lines 482-491
	8. I did not find ethical concerns or signs of duplicate publication. The study complies with basic research ethics. In summary, the work has the potential to contribute to the field but needs to address the points mentioned above before being ready for publication.	8. We sincerely thank the reviewer for confirming that the study complies with basic research ethics and does not raise any ethical concerns. As noted in the manuscript, ethical approval was obtained from the institutional review board, and the trial was registered with the Iranian Clinical	------------

		Trial Centre. All participants provided written informed consent, and confidentiality was strictly maintained throughout the study. We appreciate your acknowledgment of these ethical safeguards.	
Review 3	Thank you for the opportunity to review this manuscript. The study titled "Effect of Intra-Dialytic Pedaling Exercise on Dialysis Adequacy: A Randomized Controlled Trial" examined whether one month of intradialytic cycling sessions could impact dialysis adequacy parameters.<br><br>While the manuscript shows potential for publication in your journal, I have significant methodological and presentation concerns that must be addressed. Below I outline these issues with constructive suggestions to enhance the study's rigor and clarity.<br><br>1. General Comments<br>The acronym usage rules throughout the document appear inconsistent. If terms are consistently presented alongside their acronyms, the purpose of using acronyms becomes questionable. Similarly, some acronyms are introduced but then never used again, creating unnecessary variation.	1. We sincerely appreciate your thoughtful and constructive feedback. All of the concerns you raised have been carefully reviewed and addressed in the revised manuscript. Acronym usage has been standardized throughout the manuscript. Unnecessary acronyms have been removed, and only those used consistently have been retained.	In total of the text

	2. A notable structural issue emerged during manuscript review: Substantive methodological and analytical information is consistently positioned at the end of sections. This organizational approach forces readers to retain unanswered questions unnecessarily. I recommend restructuring to present critical details in logical sequence based on their conceptual importance.	2.Structural revisions have been implemented across the Methods section to ensure that key methodological and analytical details are presented earlier and in a more logical sequence.	The method of the article
	3.Was the type of vascular access not recorded for patients? Were all undergoing conventional hemodialysis rather than hemodiafiltration? Was the treatment uniformly administered three days per week? Since these were not mandatory inclusion/exclusion criteria, readers cannot anticipate these factors. Regarding vascular access, this variable should be included and considered based on its distribution - both in chi-square tests and as a potential study limitation.	3.Vascular access type was not recorded as a demographic variable in the original study. This has now been acknowledged as a study limitation in the revised text. We clarified that all patients underwent conventional hemodialysis (not hemodiafiltration), three times per week for four hours per session. This information has been explicitly added to the Methods section.	P8: lines 169-172; P9: Lines 173-175
	4.Was the sample probabilistic or non-probabilistic? This crucial information must be included in the Methods section. There appears to be a substantial selection bias in patient recruitment that isn't properly documented - particularly in the flowchart which fails to show:<br>a) The total pool of potentially eligible patients<br>b) The exact exclusion cascade	4.The sampling method has been specified as non-probabilistic (convenience sampling), and potential selection bias has been acknowledged. The CONSORT flowchart has been revised to include the total number of patients screened, the exclusion cascade, and the final sample analyzed. Details regarding the number of physical activity sessions (12 sessions over 4 weeks) and full adherence by all participants in the	P8: Lines 159-161<br>P13: **Figure 1:CONSORT FLOW DIAGRAM**

	I strongly recommend: Restructuring the flowchart to reflect all screening stages<br>Providing details on the number of physical activity sessions and average adherence would strengthen the methods.	intervention group have been added.	
	5. Additionally, clarifying if dropouts or missed hemodialysis sessions occurred—despite no adverse events—would enhance transparency.	5. Information about dropouts and missed dialysis sessions has been clarified—no missed sessions occurred, and reasons for withdrawal have been described.	P9: Lines 184-185
	6. All tables should include captions clearly stating the statistical tests used, ensuring consistency and reader accessibility.	6. Thank you for your helpful comment regarding the clarity and consistency of table captions. In response, we have revised the captions for all four tables to explicitly include the statistical tests used in each analysis. These updates aim to enhance transparency and reader accessibility throughout the manuscript.	P15-16: Table 1<br>P16-17: Table2<br>P17: Table3<br>P19: Table 4
	7.The CONSORT flowchart appears duplicated in the manuscript, and both versions suffer from poor resolution that compromises readability. This requires correction.	7.Thank you for your valuable observation regarding the duplication and resolution issues of the CONSORT flowchart. We have carefully revised the manuscript and removed the duplicate figure. Additionally, the flowchart has been replaced with a high-resolution version (300 dpi) to ensure optimal clarity and readability in the final publication.	P13: Consort Flow Diagram

	8. The opening of the Discussion section should be revised to first summarize the study's key findings. In its current form, it reads more like an introductory/contextual opening rather than a discussion of results.	8. We sincerely appreciate your insightful comment regarding the opening of the Discussion section. In response, we have revised the first paragraph to clearly summarize the study's key findings before proceeding to broader contextual analysis. The updated version now highlights the within-group changes observed in blood urea nitrogen (BUN), body weight, and Kt/V values, while also noting that these differences were not statistically significant between groups. This revision aims to provide a more focused and result-oriented introduction to the Discussion, in alignment with your recommendation.	P20: lines 327-334
	9.The study's justification requires major revision. Neither the introduction convincingly establishes the research's importance, nor does the discussion adequately explain how the divergent methodology resolves existing limitations. These fundamental aspects need urgent attention. How does this study address this gap? The authors should reconsider and enhance the study's justification within its proper context.<br>The authors should avoid conflating the identification of a literature gap with automatic justification for their study's urgency. A gap's mere existence does not inherently validate the research's importance – the specific value and timeliness of addressing it must be explicitly demonstrated.	9.We sincerely thank you for your insightful comments regarding the need to strengthen the study's justification. In response, we have revised both the Introduction and Discussion sections to more clearly articulate the contextual relevance, scientific value, and practical urgency of the research. Specifically, the final paragraph of the Introduction now highlights the unique environmental constraints of Bushehr—namely its hot and humid climate, which limits outdoor physical activity—and the relatively high prevalence of hemodialysis patients in the region. These factors underscore the importance of evaluating intradialytic exercise as a feasible and regionally appropriate intervention.<br><br>Additionally, in the opening and concluding paragraphs of the	P20: Lines 327-334<br>P6: 1119-129<br>P7: Lines 130-132

		Discussion section, we have emphasized how the study's design addresses limitations observed in previous research, including inconsistent findings and methodological gaps. We clarified that although between-group differences were not statistically significant, the structured and nurse-supervised nature of the intervention offers practical insights for future clinical applications, especially in settings with similar environmental and demographic characteristics. We hope these revisions adequately address your concerns and enhance the clarity, relevance, and contextual grounding of the study's rationale.	
	1) INTRODUCTION<br>1-1) Line 66<br>HD □ This is the second time this term has appeared. I suggest that the acronym be introduced at the first occurrence, and once this rule is established, it should be consistently followed. More than once, 'hemodialysis' has reappeared without being presented as an acronym.	1) INTRODUCTION<br>1-1) We sincerely appreciate your attention to consistency in terminology and your helpful suggestion regarding the use of acronyms. To ensure clarity and uniformity throughout the manuscript, we have removed the abbreviation "HD" from line 66 and opted to use the full term "hemodialysis" consistently across the entire text. This decision was made to enhance readability, particularly for multidisciplinary audiences who may not be familiar with the abbreviation. Using the complete term also aligns with editorial preferences in educational and reference-oriented manuscripts, where minimizing abbreviations can reduce ambiguity and improve comprehension.	In total of the text

	1-2) Line 71<br>Reference 12 □ Seek a more recent reference regarding the epidemiology of the treatment itself—it should be easily found. In fact, some of the references already cited may contain this information.	1-2) Thank you very much for your thoughtful suggestion regarding the need for a more recent reference on the epidemiology of hemodialysis treatment. In response, we have replaced the original citation (Lee & Son, 2021) with a more up-to-date and comprehensive source: Wang et al. (2024), published in *BMC Public Health*. This study provides a 30-year global and regional analysis of chronic kidney disease trends, including the increasing reliance on hemodialysis as a primary treatment modality. Accordingly, we have revised the corresponding sentence in the manuscript to reflect this updated evidence and ensure alignment with the study's context and rationale.	P29: Lines 547-549
	1-3) Line 72<br>Once again, 'hemodialysis' appears without being abbreviated. Consistency with the established rule is required.	1-3) Thank you for your continued attention to consistency in terminology. As noted in our previous response regarding line 66, we have intentionally chosen to use the full term "hemodialysis" throughout the manuscript rather than introducing and applying the abbreviation "HD." Therefore, no revision was made to line 72, as it aligns with our decision to maintain the complete term for clarity and accessibility. This approach is particularly beneficial for multidisciplinary readers and helps avoid potential ambiguity	In total of the text

		associated with abbreviations. We appreciate your understanding of this stylistic choice.	
	1-4) Lines 87 – 93<br>The paragraph spanning lines 87-93 may present comprehension challenges for readers with limited subject-matter expertise. I recommend both revising the content and reorganizing its structure for improved clarity.	1-4) Thank you very much for your valuable feedback regarding the clarity of the paragraph spanning lines 87–93. In response, we have revised and reorganized the content to improve readability and accessibility, particularly for readers with limited subject-matter expertise. The updated version simplifies technical expressions, introduces key concepts more gradually, and presents the information in a more structured and coherent manner.	P5: Lines 96-102
	1-5) Line 97<br>I propose including both the average weekly sedentary time imposed by the treatment and, immediately following this, the annualized measure. Presenting more tangible, daily-life metrics would significantly strengthen the argument's impact.	1-5) Thank you very much for your insightful suggestion. In response, we have revised the paragraph to include both the average weekly sedentary time imposed by hemodialysis (approximately 12 hours per week) and its annualized equivalent (nearly 800 hours per year). This adjustment aims to present the impact of treatment in more tangible, daily-life terms, thereby enhancing the clarity and persuasive strength of the argument. We appreciate your recommendation, which helped improve the contextual relevance of this section.	P5: Lines 103-106; P7: Lines 107-110
	1-6) Line 101<br>While the preceding paragraph introduces physical inactivity as negatively impacting various aspects of hemodialysis patients'	1-6) Thank you very much for your thoughtful comment. We appreciate your emphasis on maintaining a clear connection to dialysis adequacy throughout the	P5: Lines 103-106

	lives, it notably ceases to mention dialysis adequacy - despite this being the paper's primary focus as indicated in the title. Subsequently, when discussing conflicting results regarding intradialytic exercise, the text fails to specify that these contradictions relate almost solely to dialysis adequacy outcomes. Given that conflicting evidence exists for other aspects of intradialytic exercise as well, I strongly recommend clarifying at line 101 that the discussion refers exclusively to dialysis adequacy.	manuscript. In the introduction, we have already addressed dialysis adequacy both conceptually and clinically, particularly in the paragraph preceding the discussion of Kt/V and URR (lines 96–102), where its definition, significance, and impact on patient outcomes are outlined. Furthermore, in the literature review section, we have explicitly referenced studies that examined the effects of intradialytic exercise on dialysis adequacy, highlighting the conflicting findings reported by Kirkman (2013), Riahi (2012), and Groussard (2015). Nevertheless, to ensure thematic clarity, we have now added a sentence at the end of the preceding paragraph (line 100) to explicitly link physical inactivity with potential reductions in dialysis adequacy. We hope this addition reinforces the manuscript's central focus and addresses your concern effectively.	P6: Lines 107-129
	1-7) Lines 101 – 112<br>Beyond emphasizing physical activity's general importance, the introduction must specifically justify: (1) why cycling was selected as the intervention modality over other exercise types, and (2) why intradialytic cycling protocols deserve particular focus despite existing studies employing alternative modalities. This rationale requires substantial strengthening to properly contextualize your study's design choices.	1-7) Thank you for your valuable comment. To address your concern, we have added a paragraph to the introduction explaining why cycling was selected as the intervention modality and why intradialytic protocols were emphasized. The revised section highlights the practicality, safety, and suitability of pedaling exercises during dialysis, particularly for patients with limited mobility. This addition has been placed immediately after the paragraph discussing the benefits of exercise during dialysis. We hope this	P6: Lines 111-118

		clarification strengthens the rationale behind our study design.	
	1-8) Please clarify: (1) What was the intervention duration in these cited studies? (2) How does your protocol differ from existing approaches? (3) What novel contribution does your study provide compared to current literature? These justifications are critical for readers to contextualize your work's significance.	1-8) Thank you very much for your thoughtful and constructive comment. In response, we have revised the introduction to provide a clearer overview of prior studies, including the duration and structure of their interventions. These studies varied in supervision, intensity, and contextual factors, which may explain their differing outcomes.<br>To highlight the distinctiveness of our approach, we have emphasized that our protocol was designed to be context-sensitive, nurse-supervised, and feasible within routine clinical practice. Unlike previous studies, our intervention was tailored to the environmental and infrastructural constraints of Bushehr, where outdoor activity is limited due to climate conditions and dialysis burden is disproportionately high.<br>We believe this study contributes novel insights by integrating a structured intradialytic exercise program into a real-world clinical setting, with active involvement of dialysis nurses and consideration of regional challenges. These additions have been incorporated into the revised introduction to better contextualize the significance of our work.	P6: Lines 124-129;<br>P7: 130-132
	2) METHODS<br>2-1) Study Design<br>The CONSORT guidelines must be explicitly cited and followed in the main text - merely attaching them	2) METHODS<br>2-1) Thank you for your helpful comment. We have revised the *Study Design* section to state that the trial follows CONSORT	P7: lines 137-138;

	as supplementary material is insufficient.	guidelines, and added a reference to the CONSORT flow diagram (Figure 1) at the end of the *Implementation* section to illustrate participant progression. We appreciate your suggestion to improve reporting clarity.	P12: Lines 245-247
	2-2) Inclusion and exclusion criteria<br>Line 120<br>We must distinguish between cumulative hemodialysis duration (3 months) versus continuous current hemodialysis treatment (minimum 3 months) as inclusion criteria. I would recommend revising	2-2) Thank you for your valuable observation. We have revised the inclusion criteria to clarify that participants were required to be undergoing continuous hemodialysis treatment for a minimum of three months without interruption. This adjustment ensures greater precision and aligns with your recommendation.	P7: Lines 140-141
	2-3) Lines 125 – 133<br>The exclusion criteria section (line 125) should explicitly state upfront that these apply only to participants who first met all inclusion criteria. Currently this crucial context appears only inferentially in lines 129-130.	2-3) Thank you for your thoughtful comment. We have revised the exclusion criteria section to explicitly state that these criteria were applied only to individuals who had first met all inclusion criteria. This clarification has been added at the beginning of the exclusion criteria paragraph to ensure proper context and alignment with your recommendation.	P7: line 146
	2-4) Sample size and sampling method<br>Line 151	2-4) Thank you for your observation. We have reviewed and revised the paragraph accordingly, and it is now complete. We appreciate your attention to detail.	P8: Lines 152-167

	Attention needed when revising: the current paragraph remains incomplete (line 151).		
	2-5) Implementation<br>Line 180-184<br>Given the methodological importance of the intervention, I strongly advise adding a distinct subsection detailing the physical activity protocol.<br>I strongly recommend the authors consult the 'Consensus on Exercise Reporting Template (CERT): Explanation and Elaboration Statement' to enhance their training protocol description. Critical methodological details are currently missing from the reported methods.<br>Article link: https://bjsm.bmj.com/content/50/23/1428	2-5) Thank you very much for your insightful and constructive recommendation. In response, we have added a distinct subsection titled *"Exercise Protocol and Adherence (CERT-Based Description)"* within the Methods section. This new subsection provides a comprehensive and structured description of the intradialytic cycling intervention, following the guidelines outlined in the Consensus on Exercise Reporting Template (CERT) to ensure clarity, replicability, and methodological rigor.<br>We have included detailed information regarding the type, duration, intensity, supervision, setting, and monitoring of the exercise sessions, as well as adherence and safety outcomes. The CERT reference has also been cited appropriately in both the text and the reference list.	P9: Lines 173-193;<br>P10: lines 194-201
	3) RESULTS<br>3-1) Lines 222 – 224<br>This is a very important point regarding the adverse effects that could have occurred during the intervention but did not. This information should be included in the Methods section, specifying how and when this monitoring was recorded.	3) RESULTS<br>3-1) Thank you for highlighting the importance of documenting safety monitoring procedures. In response, we have clarified in the Methods section (under "Exercise Protocol and Adherence") that participants were monitored for abnormal signs or adverse events during each exercise session.	P9: Lines 189-193;<br>P10: Lines 194-201

	3-2) Line 237<br>The acronym for blood urea nitrogen (BUN) should have been first included in the 'Data collection tools' subsection of the Methods.<br><br>3-3) Table 3<br>The Kt/V values from both time points could be presented, even without statistical comparison, to properly characterize the groups.	Clinical observations were recorded by the supervising nurse throughout the intervention period as part of the safety protocol. Additionally, we have included a statement in the Results section confirming that no adverse events or exercise-related complications were observed or reported during the study. These additions aim to enhance the transparency and methodological rigor of our intervention reporting.<br><br>3-2) Thank you for your helpful observation regarding the introduction of the acronym BUN. In response, we have added a clarifying sentence in the "Data Collection Tools" subsection of the Methods to define blood urea nitrogen (BUN) at its first mention. In subsequent uses of the acronym throughout the manuscript, the full term has been omitted to avoid redundancy. We appreciate your attention to detail, which helped improve the clarity and consistency of our reporting.<br><br>3-3) Thank you for your thoughtful comment regarding the absence of pre-intervention Kt/V values. We acknowledge that having baseline Kt/V measurements would enhance longitudinal interpretation. However, calculating Kt/V prior to the intervention was not feasible due to the immediate start of the exercise protocol from the first dialysis session. In clinical practice, Kt/V is	P10: Line 207<br><br>P9: Lines 186-188; P27: Lines 488-491;

		typically measured during active dialysis sessions, and separating a true pre-intervention value would have required additional procedures outside the standard care pathway. Moreover, the intervention was pragmatically embedded into routine dialysis without a preparatory phase, which precluded baseline Kt/V collection.<br>To address your concern, we have clarified this point in the revised manuscript—specifically in the *Exercise Protocol and Adherence* subsection of the Methods and in the Limitations section of the Discussion—to ensure transparency regarding this methodological constraint.	
	4. DISCUSSION<br>4-1) Lines 256 – 262<br>This section presents a somewhat vague discussion. Describing 'exercise by exercise' does not adequately characterize the specific modalities or types employed. It is problematic to compare simple pedaling with concurrent exercise (aerobic + resistance) without explicitly addressing this distinction. Furthermore, what was the intervention duration in these studies? Were they chronic or acute? In my assessment, there appears to be substantial inconsistency in this discussion.	4. DISCUSSION<br>4-1) Thank you for your insightful feedback regarding the clarity and consistency of the discussion section. In response, we have revised this portion to more explicitly differentiate between exercise modalities and intervention durations across the cited studies. Specifically, we clarified that Kirkman et al. (2019) reported no significant improvement in dialysis adequacy following a short-term, low-intensity pedaling protocol—findings that align with our own. In contrast, Rohmah et al. (2024) demonstrated significant improvements in Kt/V following an eight-week, moderate-intensity aerobic cycling intervention. These distinctions are now integrated into	P20: Lines 335-348; P21: 349-358

		our discussion of dialysis adequacy (Kt/V), allowing for more accurate contextualization of our findings and highlighting the potential influence of exercise complexity and duration.<br>Additionally, we have retained the structured format of the discussion—organized around the study's primary outcomes (BUN, body weight, Kt/V, demographic variables)—as previously recommended by reviewers. This format ensures alignment with the study's objectives and enhances interpretive clarity.<br>We appreciate your guidance in strengthening the conceptual rigor of this section.	
	4-2) Lines 298 – 299<br>Therefore, what justifies the complete absence of reported vascular access outcomes?<br>Lines 310 – 318<br>The discussion points and information presented in this paragraph should have been introduced earlier and systematically developed throughout the Discussion section.	4-2) Thank you for your thoughtful and constructive feedback. In response to your first comment regarding the absence of reported vascular access outcomes, we have revised the discussion section to explicitly address the potential influence of vascular access quality on dialysis adequacy. Although our study did not include direct measurements of access function (e.g., flow rate or stenosis grading), we clarified that most patients utilized arteriovenous fistulas with variable maturation and flow characteristics.<br>Regarding your second comment, we agree that the methodological details and contextual limitations referenced in the original paragraph (Lines 310–318) are essential to interpreting the study's findings. Accordingly, we have relocated and expanded this content to the beginning of the "Dialysis Adequacy (Kt/V)"	P22: Lines 381-393; P23: 394-395

		subsection. This adjustment ensures that the intervention protocol and its practical constraints are introduced earlier and systematically integrated into the broader discussion. We hope these revisions enhance the clarity, coherence, and interpretive depth of the manuscript.	
	5) CONCLUSION<br>5-1) Line 328<br>Did the authors perform any sensitivity analysis by matching groups for age? This potential confounding variable warrants examination given its known influence on [relevant outcome measures	5) CONCLUSION<br>5-1) Thank you for your insightful comment regarding the potential confounding effect of age. We acknowledge that between-group differences in age may have influenced the study outcomes. Although no sensitivity analysis or age-matching was performed in the present trial, we have revised the Conclusion section to explicitly state this limitation. We have also emphasized the importance of controlling for demographic variables such as age and educational attainment in future research. Specifically, we recommend the use of stratified randomization or statistical adjustments (e.g., ANCOVA) to minimize bias and enhance internal validity. We appreciate your suggestion, which has helped us strengthen the interpretive clarity and methodological transparency of our manuscript.	P26: Lines 465-468
	6) REFERENCES<br>6-1) Lines 462 – 464<br>I was unable to locate this reference. Could the authors please	6) REFERENCES<br>6-1) Thank you very much for your careful review and for pointing out the difficulty in locating the reference cited in Lines 462–464 of	P11: Line 236;

	provide the direct DOI or URL link to the cited source?	the original manuscript. In response to your comment, we have revised the citation to include the full bibliographic information along with the direct URL link to the article. This correction is intended to facilitate access and improve transparency for readers. The updated reference now reads:<br>Nezamlo A, Oshvand K, Ghlyaf M, Homayounfar S. The effects of physical training during dialysis on the prevention of anemia in hemodialysis patients. Avicenna J Nurs Midwifery Care. 2015;23(4):65–76. Available from: http://nmj.umsha.ac.ir/article-1-1459-en.html<br>Additionally, we would like to inform you that a new reference has been added to the revised manuscript:<br>Reference 51 (Biabani et al.), which appears in Lines 659-660 of the updated version. This source provides further support for the discussion on intradialytic exercise and its physiological implications. The full citation has been included in the reference list to ensure completeness and accessibility.<br>We hope these revisions adequately address your concern and thank you again for your valuable feedback and attention to detail.	P32: Lines 659-663
Reviewer 4	I have carefully reviewed your manuscript titled "Effect of Intra-Dialytic Pedaling Exercise on Dialysis Adequacy: A Randomized Controlled Trial." The topic is clinically relevant and addresses an important area in dialysis patient care. However, several substantial concerns need to be addressed		

	before the manuscript can be considered for publication.<br><br>4-1) There are significant differences in baseline characteristics (notably, age and education level) between the intervention and control groups. These confounders may influence patients' compliance, exercise capability, and ultimately dialysis adequacy. Please provide further explanation on how you have accounted for these baseline imbalances. If possible, conduct covariate-adjusted analyses (such as ANCOVA) or consider propensity score matching to minimize the potential confounding effects.	4-1) Thank you for your insightful comment regarding baseline differences between the intervention and control groups. We acknowledge that age and educational level may act as confounding variables, potentially influencing patients' compliance, exercise capacity, and dialysis adequacy outcomes.<br><br>In response, we have clarified in the **Methods** section (under *Statistical Analysis*) that no covariate-adjusted analysis was performed due to sample size limitations, and all group comparisons were conducted using unadjusted statistical tests. Additionally, we have expanded the **Discussion** section to explicitly address this limitation, noting that the observed baseline imbalances may have introduced bias and affected the interpretation of results. We have also recommended that future studies consider covariate-adjusted analyses (e.g., ANCOVA) or propensity score matching to minimize confounding effects and enhance internal validity.	P14: Lines; 271-272<br><br>P26: Lines 465-468
	4-2) Some key findings (e.g., Kt/V improvement, p=0.11) did not reach statistical significance. Please be	4-2) Thank you for your thoughtful observation	

	more cautious in your interpretation, emphasizing that, under your study conditions, no statistically significant effects were demonstrated, and discussing the risk of type II error given your sample size.	regarding the interpretation of non-significant findings, particularly the modest improvement in Kt/V (p = 0.11). We fully agree that caution is warranted when interpreting results that do not reach statistical significance.<br><br>In response, we have revised the **Discussion** section to explicitly state that, under the conditions of this study, no statistically significant improvement in dialysis adequacy was demonstrated. We have also acknowledged the possibility of a Type II error due to the limited sample size, which may have reduced the statistical power to detect meaningful differences. Furthermore, we have recommended that future studies employ larger sample sizes and more robust designs to better assess the potential effects of intradialytic exercise.	P24: Lines 417-430;<br><br>P26: Lines 471-474
	4-3) I recommend adding figures (e.g., line graphs or bar charts) to more intuitively present the changes in major outcomes (such as Kt/V and BUN) before and after the intervention in both groups. This will improve the readability and clarity of your results.	4-3) Thank you for your valuable suggestion regarding the visual presentation of key outcomes. In response, we have added figures to illustrate the changes in BUN and Kt/V values before and after the intervention in both groups. These include a line graph for BUN levels and a bar chart for post-intervention Kt/V comparisons. References to these figures have been incorporated into the Results	P18: Figure 2: Comparison of Mean Differences in Blood Urea Nitrogen (BUN) Levels Before and After Intervention in Intervention

		section to enhance clarity and support interpretation.	and Control Groups;<br><br>P19:Figure 3: Comparison of post-intervention Kt/V values between the intervention and control groups.
	4-4) The manuscript currently lacks detailed information about exercise compliance, monitoring, and any adverse events during sessions. Please add a brief description of how session adherence was monitored, and whether any complications or dropouts related to exercise occurred.	4-4) Thank you for your constructive feedback regarding reporting standards, exercise monitoring, and adverse events. We have addressed all three points as follows:<br><br>CONSORT Guidelines Citation: In response to your recommendation, we have revised the Study Design section (Lines 137-138) to explicitly state that the trial was designed and reported in accordance with the CONSORT 2010 guidelines. This ensures transparency and alignment with international reporting standards.<br><br>CONSORT Flowchart Resolution: The duplicate flowchart has been removed, and the remaining figure has been replaced with a high-resolution version (300 dpi) to ensure optimal clarity and readability in both print and digital formats. This update is reflected in the revised figure caption.	P7: Lines 137-138;<br>P13: Figure 1: CONSORT FLOW DIAGRAM;<br>P10: Lines 194-201;<br>P14: Line 278;<br>P15: Lines 279-280

		Exercise Compliance, Monitoring, and Adverse Events: We have added a detailed description in the Methods section under the subheading Exercise Protocol and Monitoring (Lines 194-198), explaining how session adherence was supervised by dialysis nurses. Vital signs were recorded before, during, and after each session, and patients were continuously monitored for any signs of discomfort or instability. In the Results section (Lines 278-280), we have clarified that no exercise-related adverse events occurred, and noted that five participants discontinued the intervention for reasons unrelated to exercise.	

With thanks
