# Supplementary material for: Effect of intra-dialytic pedaling exercise on dialysis adequacy: A randomized controlled trial
Source: PLoS One. 2026 May 15;21(5):e0348063. doi: 10.1371/journal.pone.0348063 (PMC13178916; doi:10.1371/journal.pone.0348063)
Supplement: Attachment — Submitted filename: Response to Reviewers.docx [file pone.0348063.s008.docx]

Dear Editor

Respectfully, according to the comments of the esteemed referees, corrections were made in the text of the article as follows

| Source of Comment | Comments | Response to editor/ reviewer | Page/ line |
| --- | --- | --- | --- |
| Ankur Shah Academic Editor PLOS ONE | Please include the following items when submitting your revised manuscript:  - A rebuttal letter that responds to each point raised by the academic editor and reviewer(s). You should upload this letter as a separate file labeled 'Response to Reviewers'.  -A marked-up copy of your manuscript that highlights changes made to the original version. You should upload this as a separate file labeled 'Revised Manuscript with Track Changes'.  An unmarked version of your revised paper without tracked changes. You should upload this as a separate file labeled 'Manuscript'. | -Thank you for your guidance. The requested files have been prepared and uploaded as separate documents  -Thank you for your valuable suggestion. In accordance with your recommendation, we have prepared and uploaded two separate files:  A revised manuscript with track changes clearly highlighting all modifications made to the original version, labeled "Revised Manuscript with Track Changes".  A clean, unmarked version of the revised paper, labeled "Manuscript".  We appreciate your guidance and hope these revisions meet the journal’s requirements. | -Revisions made as requested; related files uploaded during online submission |
| Journal requirements | 1. Please ensure that your manuscript meets PLOS ONE's style requirements, including those for file naming. | Thank you for your guidance. We have carefully reviewed and followed PLOS ONE’s style requirements, including the file naming conventions. All submitted documents have been labeled appropriately as per the journal’s instructions. We appreciate your attention to detail and your support throughout the revision process. | ------------ |
|  | 2.We note that you have selected “Clinical Trial” as your article type. Please state the name of the registry and the registration number (e.g. ISRCTN or ClinicalTrials.gov) in the submission data and on the title page of your manuscript. a) Please provide the complete date range for participant recruitment and follow-up in the methods section of your manuscript. b) We note that the grant information you provided in the ‘Funding Information’ and ‘Financial Disclosure’ sections do not match. When you resubmit, please ensure that you provide the correct grant numbers for the awards you received for your study in the ‘Funding Information’ section. | 2. Thank you for your detailed guidance. We have carefully addressed all the points raised:  As requested, the name of the clinical trial registry and the registration number have been clearly stated in both the submission metadata and on the title page of the manuscript.  a. In response to your comment regarding the study timeline, we have added a clear statement in the Implementation section of the Methods, specifying the complete date range for participant recruitment and intervention. We have also clarified that no post-intervention follow-up period was included, and all outcome measurements were collected immediately after the final session.  b. We have reviewed and corrected the grant information to ensure consistency between the ‘Funding Information’ and ‘Financial Disclosure’ sections. The correct grant numbers have now been provided in the ‘Funding Information’ section.  We appreciate your support and attention to detail throughout the revision process. | ------------- |
|  |  |  |  |
|  | 3. In the online submission form, you indicated that ["The datasets generated and analyzed during the current study are available in the library of Bushehr University of Medical Sciences, in the thesis of a Master of Nursing student named Mahmoud Mohammadizadeh. The full trial protocol is also available in this thesis. Interested researchers may contact the corresponding author to request access to this data].  All PLOS journals now require all data underlying the findings described in their manuscript to be freely available to other researchers, either 1. In a public repository, 2. Within the manuscript itself, or 3. Uploaded as supplementary information.  This policy applies to all data except where public deposition would breach compliance with the protocol approved by your research ethics board. If your data cannot be made publicly available for ethical or legal reasons (e.g., public availability would compromise patient privacy), please explain your reasons on resubmission and your exemption request will be escalated for approval. | 3. Thank you for your comment. Due to ethical considerations and patient privacy protections approved by our institutional ethics committee, the dataset cannot be made publicly available. However, the full SPSS dataset has been uploaded as a supplementary file during resubmission for editorial review only. We kindly request that access to this file be restricted to the editorial team. | ------------- |
|  | 4. When completing the data availability statement of the submission form, you indicated that you will make your data available on acceptance. We strongly recommend all authors decide on a data sharing plan before acceptance, as the process can be lengthy and hold up publication timelines. Please note that, though access restrictions are acceptable now, your entire data will need to be made freely accessible if your manuscript is accepted for publication. This policy applies to all data except where public deposition would breach compliance with the protocol approved by your research ethics board. If you are unable to adhere to our open data policy, please kindly revise your statement to explain your reasoning and we will seek the editor's input on an exemption. Please be assured that, once you have provided your new statement, the assessment of your exemption will not hold up the peer review process. | Thank you for your comment. The full results of the study have been reported in the manuscript. The SPSS dataset containing raw data has been uploaded as a supplementary file for editorial review only. Due to ethical restrictions and patient privacy protections approved by our institutional ethics committee, the dataset cannot be made publicly available. We kindly request that access to this file be limited to the editorial team. This limitation has also been clearly stated in the Data Availability section of the manuscript. | ------------- |
|  | 5. Your ethics statement should only appear in the Methods section of your manuscript. If your ethics statement is written in any section besides the Methods, please delete it from any other section. | Thank you for your observation. We have carefully reviewed the manuscript and confirm that the ethics statement appears only in the Methods section, in accordance with the journal’s guidelines. No reference to ethical approval has been made in any other section of the manuscript. | -------------- |
|  | 6. Please include captions for your Supporting Information files at the end of your manuscript, and update any in-text citations to match accordingly. Please see our Supporting Information guidelines for more information: http://journals.plos.org/plosone/s/supporting-information. | Thank you for your helpful guidance. In response to your comment, we have added a caption for the Supporting Information file at the end of the manuscript under the section titled “Supporting Information Captions.” We have also reviewed the manuscript and updated any in-text citations to ensure they match the file name and numbering format in accordance with the journal’s guidelines. | P33: Lines 684-691;  P34: Lines 692-696 |
| Reviewer 1: | Authors recruited 84 hemodialysis patients with a randomized controlled trial to investigate the effectiveness of physical activity on dialysis effectiveness. The results no significant improvement in dialysis effectiveness with pedaling exercise during hemodialysis.  1. Line 179. The control group underwent hemodialysis using the routine method. Please clarify the “routine method”.  2. The experimental group and control group participated in different activities: Physical activity during dialysis vs. the routine method. Does the routine method include physical activity? If not, how to ensure to have a single blind study design?  3. Line 197. Some participants were excluded after the start of the study. Please clarify the exclusion criteria here.    4. Tables vs text. The decimal point was presented in different format which should be corrected to be consistent.  5. Line 215 mentioned ANOVA and Tukey’s post hoc test in the analysis section. Please clarify where the corresponding results for ANOVA/post hoc test were presented.  6. It seems that authors only conduct the comparison of two groups for before intervention and then another comparison of two groups for after intervention. As the baseline characteristics are not the same for some variables. It is unclear how these variables were taken into consideration in the analysis to evaluate the effective of physical activity. | 1.Thank you very much for your valuable comment regarding the description of the "routine method" applied to the control group.  In response to your suggestion, we have carefully revised the manuscript to clarify that the control group received the standard hemodialysis treatment without any additional physical activity intervention. Specifically, patients in the control group underwent routine hemodialysis sessions lasting four hours, three times per week, following the usual hospital protocols. They did not participate in any pedaling exercise or other intradialytic physical activity during these sessions. The term "routine method" now clearly refers to this standard care without added exercise.  2.We respectfully thank the reviewer for the valuable comment. We have carefully revised the manuscript accordingly to clarify the study’s sample size determination, randomization process, and blinding procedures. Specifically, we have detailed that the sample size was determined a priori and that participants were randomly assigned using sealed, coded opaque envelopes to minimize selection bias. The single-blind design, with participants blinded to their group allocation and treated in separate centers and times, is now explicitly described to ensure preservation of blinding. The standard care received by the control group and the acknowledgement of the limitation related to the unblinded medical staff have been clearly stated. These revisions have been implemented throughout the manuscript as suggested.  3.Thank you very much for your valuable comment. In response, we have revised the manuscript to clarify the reasons for participant withdrawal after the start of the study. Specifically, two participants from the intervention group and three from the control group discontinued their involvement due to severe fatigue during dialysis and a lack of willingness to continue the prescribed activities. This information has now been added to the relevant section of the manuscript to ensure transparency and improve clarity regarding the exclusion criteria.  4.Thank you very much for your helpful observation. We have carefully reviewed the manuscript and corrected the formatting of all decimal points to ensure consistency between tables and text. The revised version now follows a uniform style throughout the document.  5. Thank you very much for your thoughtful comment. We appreciate your attention to the clarity of the statistical methods. The reference to ANOVA and Tukey’s post hoc test in the analysis section was mistakenly carried over from other parts of the broader study, which are not included in the current manuscript. As the present article focuses solely on the effect of physical activity on dialysis adequacy, and does not report outcomes related to fatigue or quality of life, we have revised the analysis section accordingly. The statistical methods now accurately reflect the tests used for evaluating dialysis adequacy. We sincerely appreciate your observation, which helped us improve the precision of our reporting.  6. Thank you for your valuable feedback. To address your concern regarding baseline differences and the analytical approach, we have added Table 3, which presents the mean changes in blood urea, body weight, and BUN between the intervention and control groups. This allows for a direct comparison of the intervention’s effect, independent of baseline values.  Additionally, the Results section has been revised to clarify that change scores were used to evaluate the impact of physical activity. This approach helps account for initial group differences and provides a more accurate reflection of the intervention’s effectiveness. |  |
|  |  |  | P11: Lines 225-226  P8: Lines 162-168  P26: Line 478;  P27: Lines; 479-481  P15: Lines: 279-280  P12: Lines 239-241  Total of Tables  We have removed the reference to ANOVA and Tukey’s post hoc test from the analysis section.  P17: Lines 302-304  (Table 3) |
| Reviewer 2 | Congratulations on your study about exercise during hemodialysis and its effect on dialysis adequacy. The topic is important and relevant for improving the quality of life of patients with kidney disease. However, after careful review of the manuscript, I would like to highlight some points that need improvement to make the work clearer and more scientific.  1. First, it is essential that all data supporting the analyses and conclusions be available to readers, either as supplementary files or in a public repository. This allows other researchers to verify and use the results reliably.  2.The statistical analysis is appropriate, but it would be helpful to include additional information such as effect sizes and confidence intervals, so readers better understand the clinical significance of the findings. I also suggest explaining how the sample size was calculated and how missing data or participant dropouts were handled.  3.Another important point is that there were significant differences between groups regarding age and education level, which might have influenced the results. This issue should be discussed more carefully, as it may affect the exercise impact.  4. Additionally, the study was single-blind, meaning the assessors knew the group assignments, which could introduce some bias. This possible bias should be addressed in the manuscript.  5. The writing could be clearer and more concise, and I recommend a thorough English language review to correct any errors.    6.The discussion section could also be more straightforward, avoiding repetition and better explaining the reasons behind the findings, especially the nonsignificant results. It is important to discuss what these results mean in practical terms for patients.  7.It is also important to note that the study was conducted at a single site, with a small sample and a specific patient group, which limits the generalizability of the findings. Therefore, generalizing the results should be done cautiously.    8. I did not find ethical concerns or signs of duplicate publication. The study complies with basic research ethics. In summary, the work has the potential to contribute to the field but needs to address the points mentioned above before being ready for publication. | 1. Thank you for your valuable comment. In accordance with the journal’s data sharing policy, we have uploaded the full SPSS dataset as a Supporting Information file during resubmission. This file contains the anonymized raw data used in our statistical analyses and has been provided for editorial review. Due to ethical restrictions and patient privacy protections approved by our institutional ethics committee, public access to this dataset is not permitted.  2. We sincerely thank the reviewer for their thoughtful and constructive feedback. In response to the suggestion, we have revised the manuscript to include effect sizes (Cohen’s d) and 95% confidence intervals for key comparisons. These additions have been incorporated directly into Tables 1 through 4 to enhance the interpretation of clinical significance.  Furthermore, we have clarified the sample size calculation in the Methods section, including the rationale and parameters used. We have also added a statement explaining how participant dropouts were handled: individuals who withdrew from the study were excluded from the final analysis, and all statistical tests were conducted using complete-case data.  3.We appreciate the reviewer’s thoughtful observation. In response, we have expanded the Discussion section to acknowledge the significant differences in age and education between groups and their potential influence on intervention outcomes. These demographic factors may affect motivation, adherence, and responsiveness to physical activity, as supported by prior research.  4.Thank you for your valuable comment regarding the study’s blinding design. We have clarified in the revised manuscript that this was a single-blind trial, in which participants were blinded to group allocation. To maintain blinding, the intervention and control groups were treated in separate dialysis centers and during different time periods, minimizing the risk of cross-group awareness. Due to the nature of the intervention, blinding of medical staff and assessors was not feasible and has been acknowledged as a study limitation. We have now explicitly addressed this potential source of bias in the manuscript to enhance transparency and methodological rigor.  5.Thank you for your helpful comment regarding the clarity and language quality of the manuscript. In response, we have carefully reviewed the entire text and revised multiple sections to improve sentence structure, eliminate redundancy, and enhance overall readability. We have also conducted a thorough English language edit to correct any grammatical or stylistic issues.  6.We sincerely thank the reviewer for their constructive feedback on the Discussion section. In response, we have revised this section to improve clarity, reduce repetition, and present the findings in a more structured and straightforward manner. The discussion is now organized into thematic categories based on the study’s main outcomes—blood urea and BUN levels, body weight changes, dialysis adequacy (Kt/V), and demographic influences—to enhance interpretability.  We have also expanded our explanation of the nonsignificant results by incorporating physiological, methodological, and demographic considerations. Furthermore, we added a dedicated subsection outlining the practical implications of the findings for patient care, emphasizing the feasibility and potential value of intradialytic physical activity despite the lack of statistically significant effects.  7. We sincerely appreciate your thoughtful observation regarding the limited generalizability of the study due to its single-site design, small sample size, and specific patient population. In response, we have carefully addressed these concerns in the Limitations section of the revised manuscript. Specifically, we have acknowledged the restricted geographic scope, the use of convenience sampling, and the demographic characteristics of the participants, which may limit the applicability of the findings to broader hemodialysis populations or different clinical settings. To reinforce this point, we have added a concluding statement emphasizing the need for caution when generalizing the results.  8. We sincerely thank the reviewer for confirming that the study complies with basic research ethics and does not raise any ethical concerns. As noted in the manuscript, ethical approval was obtained from the institutional review board, and the trial was registered with the Iranian Clinical Trial Centre. All participants provided written informed consent, and confidentiality was strictly maintained throughout the study. We appreciate your acknowledgment of these ethical safeguards. | The anonymized SPSS dataset was uploaded as a Supporting Information file during the resubmission process.  P15-19; Table 1, Table 2, Table3 and Table 4;  P8; lines 152-161  P12; Lines 239-241  P14; lines 269-272  P23: Lines 410-414;  P24: Lines 415-425  P26: lines 465-468  P8: Lines 162-168  P26: Lines 478; P27: Lines 479-481  Total of the text  P20-25  P26: Lines 482-491  ------------ |
| Review 3 | Thank you for the opportunity to review this manuscript. The study titled "Effect of Intra-Dialytic Pedaling Exercise on Dialysis Adequacy: A Randomized Controlled Trial" examined whether one month of intradialytic cycling sessions could impact dialysis adequacy parameters.  While the manuscript shows potential for publication in your journal, I have significant methodological and presentation concerns that must be addressed. Below I outline these issues with constructive suggestions to enhance the study's rigor and clarity.  1. General Comments  The acronym usage rules throughout the document appear inconsistent. If terms are consistently presented alongside their acronyms, the purpose of using acronyms becomes questionable. Similarly, some acronyms are introduced but then never used again, creating unnecessary variation.  2. A notable structural issue emerged during manuscript review: Substantive methodological and analytical information is consistently positioned at the end of sections. This organizational approach forces readers to retain unanswered questions unnecessarily. I recommend restructuring to present critical details in logical sequence based on their conceptual importance.  3.Was the type of vascular access not recorded for patients? Were all undergoing conventional hemodialysis rather than hemodiafiltration? Was the treatment uniformly administered three days per week? Since these were not mandatory inclusion/exclusion criteria, readers cannot anticipate these factors. Regarding vascular access, this variable should be included and considered based on its distribution - both in chi-square tests and as a potential study limitation.  4.Was the sample probabilistic or non-probabilistic? This crucial information must be included in the Methods section. There appears to be a substantial selection bias in patient recruitment that isn't properly documented - particularly in the flowchart which fails to show:  a) The total pool of potentially eligible patients  b) The exact exclusion cascade  I strongly recommend: Restructuring the flowchart to reflect all screening stages  Providing details on the number of physical activity sessions and average adherence would strengthen the methods.  5. Additionally, clarifying if dropouts or missed hemodialysis sessions occurred—despite no adverse events—would enhance transparency.  6. All tables should include captions clearly stating the statistical tests used, ensuring consistency and reader accessibility.    7.The CONSORT flowchart appears duplicated in the manuscript, and both versions suffer from poor resolution that compromises readability. This requires correction.  8. The opening of the Discussion section should be revised to first summarize the study's key findings. In its current form, it reads more like an introductory/contextual opening rather than a discussion of results.  9.The study's justification requires major revision. Neither the introduction convincingly establishes the research's importance, nor does the discussion adequately explain how the divergent methodology resolves existing limitations. These fundamental aspects need urgent attention. How does this study address this gap? The authors should reconsider and enhance the study's justification within its proper context.  The authors should avoid conflating the identification of a literature gap with automatic justification for their study's urgency. A gap's mere existence does not inherently validate the research's importance – the specific value and timeliness of addressing it must be explicitly demonstrated.  1) INTRODUCTION  1-1) Line 66  HD  This is the second time this term has appeared. I suggest that the acronym be introduced at the first occurrence, and once this rule is established, it should be consistently followed. More than once, 'hemodialysis' has reappeared without being presented as an acronym.  1-2) Line 71  Reference 12  Seek a more recent reference regarding the epidemiology of the treatment itself—it should be easily found. In fact, some of the references already cited may contain this information.    1-3) Line 72  Once again, 'hemodialysis' appears without being abbreviated. Consistency with the established rule is required.  1-4) Lines 87 – 93  The paragraph spanning lines 87-93 may present comprehension challenges for readers with limited subject-matter expertise. I recommend both revising the content and reorganizing its structure for improved clarity.  1-5) Line 97  I propose including both the average weekly sedentary time imposed by the treatment and, immediately following this, the annualized measure. Presenting more tangible, daily-life metrics would significantly strengthen the argument's impact.  1-6) Line 101  While the preceding paragraph introduces physical inactivity as negatively impacting various aspects of hemodialysis patients' lives, it notably ceases to mention dialysis adequacy - despite this being the paper's primary focus as indicated in the title. Subsequently, when discussing conflicting results regarding intradialytic exercise, the text fails to specify that these contradictions relate almost solely to dialysis adequacy outcomes. Given that conflicting evidence exists for other aspects of intradialytic exercise as well, I strongly recommend clarifying at line 101 that the discussion refers exclusively to dialysis adequacy.  1-7) Lines 101 – 112  Beyond emphasizing physical activity's general importance, the introduction must specifically justify: (1) why cycling was selected as the intervention modality over other exercise types, and (2) why intradialytic cycling protocols deserve particular focus despite existing studies employing alternative modalities. This rationale requires substantial strengthening to properly contextualize your study's design choices.  1-8) Please clarify: (1) What was the intervention duration in these cited studies? (2) How does your protocol differ from existing approaches? (3) What novel contribution does your study provide compared to current literature? These justifications are critical for readers to contextualize your work's significance.  2) METHODS  2-1) Study Design  The CONSORT guidelines must be explicitly cited and followed in the main text - merely attaching them as supplementary material is insufficient.      2-2) Inclusion and exclusion criteria  Line 120  We must distinguish between cumulative hemodialysis duration (3 months) versus continuous current hemodialysis treatment (minimum 3 months) as inclusion criteria. I would recommend revising    2-3) Lines 125 – 133  The exclusion criteria section (line 125) should explicitly state upfront that these apply only to participants who first met all inclusion criteria. Currently this crucial context appears only inferentially in lines 129-130.  2-4) Sample size and sampling method  Line 151  Attention needed when revising: the current paragraph remains incomplete (line 151).  2-5) Implementation  Line 180-184  Given the methodological importance of the intervention, I strongly advise adding a distinct subsection detailing the physical activity protocol.  I strongly recommend the authors consult the 'Consensus on Exercise Reporting Template (CERT): Explanation and Elaboration Statement' to enhance their training protocol description. Critical methodological details are currently missing from the reported methods.  Article link: https://bjsm.bmj.com/content/50/23/1428  3) RESULTS  3-1) Lines 222 – 224  This is a very important point regarding the adverse effects that could have occurred during the intervention but did not. This information should be included in the Methods section, specifying how and when this monitoring was recorded.    3-2) Line 237  The acronym for blood urea nitrogen (BUN) should have been first included in the 'Data collection tools' subsection of the Methods.    3-3) Table 3  The Kt/V values from both time points could be presented, even without statistical comparison, to properly characterize the groups.  4. DISCUSSION  4-1) Lines 256 – 262  This section presents a somewhat vague discussion. Describing 'exercise by exercise' does not adequately characterize the specific modalities or types employed. It is problematic to compare simple pedaling with concurrent exercise (aerobic + resistance) without explicitly addressing this distinction. Furthermore, what was the intervention duration in these studies? Were they chronic or acute? In my assessment, there appears to be substantial inconsistency in this discussion.  4-2) Lines 298 – 299  Therefore, what justifies the complete absence of reported vascular access outcomes?  Lines 310 – 318  The discussion points and information presented in this paragraph should have been introduced earlier and systematically developed throughout the Discussion section.  5) CONCLUSION  5-1) Line 328  Did the authors perform any sensitivity analysis by matching groups for age? This potential confounding variable warrants examination given its known influence on [relevant outcome measures  6) REFERENCES  6-1) Lines 462 – 464  I was unable to locate this reference. Could the authors please provide the direct DOI or URL link to the cited source? | 1. We sincerely appreciate your thoughtful and constructive feedback. All of the concerns you raised have been carefully reviewed and addressed in the revised manuscript. Acronym usage has been standardized throughout the manuscript. Unnecessary acronyms have been removed, and only those used consistently have been retained.  2.Structural revisions have been implemented across the Methods section to ensure that key methodological and analytical details are presented earlier and in a more logical sequence.  3.Vascular access type was not recorded as a demographic variable in the original study. This has now been acknowledged as a study limitation in the revised text. We clarified that all patients underwent conventional hemodialysis (not hemodiafiltration), three times per week for four hours per session. This information has been explicitly added to the Methods section.  4.The sampling method has been specified as non-probabilistic (convenience sampling), and potential selection bias has been acknowledged. The CONSORT flowchart has been revised to include the total number of patients screened, the exclusion cascade, and the final sample analyzed. Details regarding the number of physical activity sessions (12 sessions over 4 weeks) and full adherence by all participants in the intervention group have been added.  5. Information about dropouts and missed dialysis sessions has been clarified—no missed sessions occurred, and reasons for withdrawal have been described.  6. Thank you for your helpful comment regarding the clarity and consistency of table captions. In response, we have revised the captions for all four tables to explicitly include the statistical tests used in each analysis. These updates aim to enhance transparency and reader accessibility throughout the manuscript.  7.Thank you for your valuable observation regarding the duplication and resolution issues of the CONSORT flowchart. We have carefully revised the manuscript and removed the duplicate figure. Additionally, the flowchart has been replaced with a high-resolution version (300 dpi) to ensure optimal clarity and readability in the final publication.  8. We sincerely appreciate your insightful comment regarding the opening of the Discussion section. In response, we have revised the first paragraph to clearly summarize the study’s key findings before proceeding to broader contextual analysis. The updated version now highlights the within-group changes observed in blood urea nitrogen (BUN), body weight, and Kt/V values, while also noting that these differences were not statistically significant between groups. This revision aims to provide a more focused and result-oriented introduction to the Discussion, in alignment with your recommendation.  9.We sincerely thank you for your insightful comments regarding the need to strengthen the study’s justification. In response, we have revised both the Introduction and Discussion sections to more clearly articulate the contextual relevance, scientific value, and practical urgency of the research. Specifically, the final paragraph of the Introduction now highlights the unique environmental constraints of Bushehr—namely its hot and humid climate, which limits outdoor physical activity—and the relatively high prevalence of hemodialysis patients in the region. These factors underscore the importance of evaluating intradialytic exercise as a feasible and regionally appropriate intervention.  Additionally, in the opening and concluding paragraphs of the Discussion section, we have emphasized how the study’s design addresses limitations observed in previous research, including inconsistent findings and methodological gaps. We clarified that although between-group differences were not statistically significant, the structured and nurse-supervised nature of the intervention offers practical insights for future clinical applications, especially in settings with similar environmental and demographic characteristics. We hope these revisions adequately address your concerns and enhance the clarity, relevance, and contextual grounding of the study’s rationale.  1) INTRODUCTION  1-1) We sincerely appreciate your attention to consistency in terminology and your helpful suggestion regarding the use of acronyms. To ensure clarity and uniformity throughout the manuscript, we have removed the abbreviation “HD” from line 66 and opted to use the full term “hemodialysis” consistently across the entire text. This decision was made to enhance readability, particularly for multidisciplinary audiences who may not be familiar with the abbreviation. Using the complete term also aligns with editorial preferences in educational and reference-oriented manuscripts, where minimizing abbreviations can reduce ambiguity and improve comprehension.    1-2) Thank you very much for your thoughtful suggestion regarding the need for a more recent reference on the epidemiology of hemodialysis treatment. In response, we have replaced the original citation (Lee & Son, 2021) with a more up-to-date and comprehensive source: Wang et al. (2024), published in BMC Public Health. This study provides a 30-year global and regional analysis of chronic kidney disease trends, including the increasing reliance on hemodialysis as a primary treatment modality. Accordingly, we have revised the corresponding sentence in the manuscript to reflect this updated evidence and ensure alignment with the study’s context and rationale.  1-3) Thank you for your continued attention to consistency in terminology. As noted in our previous response regarding line 66, we have intentionally chosen to use the full term “hemodialysis” throughout the manuscript rather than introducing and applying the abbreviation “HD.” Therefore, no revision was made to line 72, as it aligns with our decision to maintain the complete term for clarity and accessibility. This approach is particularly beneficial for multidisciplinary readers and helps avoid potential ambiguity associated with abbreviations. We appreciate your understanding of this stylistic choice.  1-4) Thank you very much for your valuable feedback regarding the clarity of the paragraph spanning lines 87–93. In response, we have revised and reorganized the content to improve readability and accessibility, particularly for readers with limited subject-matter expertise. The updated version simplifies technical expressions, introduces key concepts more gradually, and presents the information in a more structured and coherent manner.  1-5) Thank you very much for your insightful suggestion. In response, we have revised the paragraph to include both the average weekly sedentary time imposed by hemodialysis (approximately 12 hours per week) and its annualized equivalent (nearly 800 hours per year). This adjustment aims to present the impact of treatment in more tangible, daily-life terms, thereby enhancing the clarity and persuasive strength of the argument. We appreciate your recommendation, which helped improve the contextual relevance of this section.  1-6) Thank you very much for your thoughtful comment. We appreciate your emphasis on maintaining a clear connection to dialysis adequacy throughout the manuscript. In the introduction, we have already addressed dialysis adequacy both conceptually and clinically, particularly in the paragraph preceding the discussion of Kt/V and URR (lines 96–102), where its definition, significance, and impact on patient outcomes are outlined. Furthermore, in the literature review section, we have explicitly referenced studies that examined the effects of intradialytic exercise on dialysis adequacy, highlighting the conflicting findings reported by Kirkman (2013), Riahi (2012), and Groussard (2015). Nevertheless, to ensure thematic clarity, we have now added a sentence at the end of the preceding paragraph (line 100) to explicitly link physical inactivity with potential reductions in dialysis adequacy. We hope this addition reinforces the manuscript’s central focus and addresses your concern effectively.  1-7) Thank you for your valuable comment. To address your concern, we have added a paragraph to the introduction explaining why cycling was selected as the intervention modality and why intradialytic protocols were emphasized. The revised section highlights the practicality, safety, and suitability of pedaling exercises during dialysis, particularly for patients with limited mobility. This addition has been placed immediately after the paragraph discussing the benefits of exercise during dialysis. We hope this clarification strengthens the rationale behind our study design.  1-8) Thank you very much for your thoughtful and constructive comment. In response, we have revised the introduction to provide a clearer overview of prior studies, including the duration and structure of their interventions. These studies varied in supervision, intensity, and contextual factors, which may explain their differing outcomes.  To highlight the distinctiveness of our approach, we have emphasized that our protocol was designed to be context-sensitive, nurse-supervised, and feasible within routine clinical practice. Unlike previous studies, our intervention was tailored to the environmental and infrastructural constraints of Bushehr, where outdoor activity is limited due to climate conditions and dialysis burden is disproportionately high.  We believe this study contributes novel insights by integrating a structured intradialytic exercise program into a real-world clinical setting, with active involvement of dialysis nurses and consideration of regional challenges. These additions have been incorporated into the revised introduction to better contextualize the significance of our work.  2) METHODS  2-1) Thank you for your helpful comment. We have revised the *Study Design* section to state that the trial follows CONSORT guidelines, and added a reference to the CONSORT flow diagram (Figure 1) at the end of the *Implementation* section to illustrate participant progression. We appreciate your suggestion to improve reporting clarity.  2-2) Thank you for your valuable observation. We have revised the inclusion criteria to clarify that participants were required to be undergoing continuous hemodialysis treatment for a minimum of three months without interruption. This adjustment ensures greater precision and aligns with your recommendation.  2-3) Thank you for your thoughtful comment. We have revised the exclusion criteria section to explicitly state that these criteria were applied only to individuals who had first met all inclusion criteria. This clarification has been added at the beginning of the exclusion criteria paragraph to ensure proper context and alignment with your recommendation.  2-4) Thank you for your observation. We have reviewed and revised the paragraph accordingly, and it is now complete. We appreciate your attention to detail.  2-5) Thank you very much for your insightful and constructive recommendation. In response, we have added a distinct subsection titled *“Exercise Protocol and Adherence (CERT-Based Description)”* within the Methods section. This new subsection provides a comprehensive and structured description of the intradialytic cycling intervention, following the guidelines outlined in the Consensus on Exercise Reporting Template (CERT) to ensure clarity, replicability, and methodological rigor.  We have included detailed information regarding the type, duration, intensity, supervision, setting, and monitoring of the exercise sessions, as well as adherence and safety outcomes. The CERT reference has also been cited appropriately in both the text and the reference list.  3) RESULTS  3-1) Thank you for highlighting the importance of documenting safety monitoring procedures. In response, we have clarified in the Methods section (under “Exercise Protocol and Adherence”) that participants were monitored for abnormal signs or adverse events during each exercise session. Clinical observations were recorded by the supervising nurse throughout the intervention period as part of the safety protocol. Additionally, we have included a statement in the Results section confirming that no adverse events or exercise-related complications were observed or reported during the study. These additions aim to enhance the transparency and methodological rigor of our intervention reporting.  3-2) Thank you for your helpful observation regarding the introduction of the acronym BUN. In response, we have added a clarifying sentence in the “Data Collection Tools” subsection of the Methods to define blood urea nitrogen (BUN) at its first mention. In subsequent uses of the acronym throughout the manuscript, the full term has been omitted to avoid redundancy. We appreciate your attention to detail, which helped improve the clarity and consistency of our reporting.  3-3) Thank you for your thoughtful comment regarding the absence of pre-intervention Kt/V values. We acknowledge that having baseline Kt/V measurements would enhance longitudinal interpretation. However, calculating Kt/V prior to the intervention was not feasible due to the immediate start of the exercise protocol from the first dialysis session. In clinical practice, Kt/V is typically measured during active dialysis sessions, and separating a true pre-intervention value would have required additional procedures outside the standard care pathway. Moreover, the intervention was pragmatically embedded into routine dialysis without a preparatory phase, which precluded baseline Kt/V collection.  To address your concern, we have clarified this point in the revised manuscript—specifically in the *Exercise Protocol and Adherence* subsection of the Methods and in the Limitations section of the Discussion—to ensure transparency regarding this methodological constraint.  4. DISCUSSION  4-1) Thank you for your insightful feedback regarding the clarity and consistency of the discussion section. In response, we have revised this portion to more explicitly differentiate between exercise modalities and intervention durations across the cited studies. Specifically, we clarified that Kirkman et al. (2019) reported no significant improvement in dialysis adequacy following a short-term, low-intensity pedaling protocol—findings that align with our own. In contrast, Rohmah et al. (2024) demonstrated significant improvements in Kt/V following an eight-week, moderate-intensity aerobic cycling intervention. These distinctions are now integrated into our discussion of dialysis adequacy (Kt/V), allowing for more accurate contextualization of our findings and highlighting the potential influence of exercise complexity and duration.  Additionally, we have retained the structured format of the discussion—organized around the study’s primary outcomes (BUN, body weight, Kt/V, demographic variables)—as previously recommended by reviewers. This format ensures alignment with the study’s objectives and enhances interpretive clarity.  We appreciate your guidance in strengthening the conceptual rigor of this section.  4-2) Thank you for your thoughtful and constructive feedback. In response to your first comment regarding the absence of reported vascular access outcomes, we have revised the discussion section to explicitly address the potential influence of vascular access quality on dialysis adequacy. Although our study did not include direct measurements of access function (e.g., flow rate or stenosis grading), we clarified that most patients utilized arteriovenous fistulas with variable maturation and flow characteristics.  Regarding your second comment, we agree that the methodological details and contextual limitations referenced in the original paragraph (Lines 310–318) are essential to interpreting the study’s findings. Accordingly, we have relocated and expanded this content to the beginning of the “Dialysis Adequacy (Kt/V)” subsection. This adjustment ensures that the intervention protocol and its practical constraints are introduced earlier and systematically integrated into the broader discussion. We hope these revisions enhance the clarity, coherence, and interpretive depth of the manuscript.  5) CONCLUSION  5-1) Thank you for your insightful comment regarding the potential confounding effect of age. We acknowledge that between-group differences in age may have influenced the study outcomes. Although no sensitivity analysis or age-matching was performed in the present trial, we have revised the Conclusion section to explicitly state this limitation. We have also emphasized the importance of controlling for demographic variables such as age and educational attainment in future research. Specifically, we recommend the use of stratified randomization or statistical adjustments (e.g., ANCOVA) to minimize bias and enhance internal validity. We appreciate your suggestion, which has helped us strengthen the interpretive clarity and methodological transparency of our manuscript.  6) REFERENCES  6-1) Thank you very much for your careful review and for pointing out the difficulty in locating the reference cited in Lines 462–464 of the original manuscript. In response to your comment, we have revised the citation to include the full bibliographic information along with the direct URL link to the article. This correction is intended to facilitate access and improve transparency for readers. The updated reference now reads:  Nezamlo A, Oshvand K, Ghlyaf M, Homayounfar S. The effects of physical training during dialysis on the prevention of anemia in hemodialysis patients. Avicenna J Nurs Midwifery Care. 2015;23(4):65–76. Available from: http://nmj.umsha.ac.ir/article-1-1459-en.html  Additionally, we would like to inform you that a new reference has been added to the revised manuscript:  Reference 51 (Biabani et al.), which appears in Lines 659-660 of the updated version. This source provides further support for the discussion on intradialytic exercise and its physiological implications. The full citation has been included in the reference list to ensure completeness and accessibility.  We hope these revisions adequately address your concern and thank you again for your valuable feedback and attention to detail. | In total of the text  The method of the article  P8: lines 169-172;  P9: Lines 173-175  P8: Lines 159-161  P13: **Figure 1:CONSORT FLOW DIAGRAM**  P9: Lines 184-185  P15-16: Table 1  P16-17: Table2  P17: Table3  P19: Table 4  P13: Consort Flow Diagram  P20: lines 327-334  P20: Lines 327-334  P6: 1119-129  P7: Lines 130-132  In total of the text  P29: Lines 547-549  In total of the text  P5: Lines 96-102  P5: Lines 103-106; P7: Lines 107-110  P5: Lines 103-106  P6: Lines 107-129  P6: Lines 111-118  P6: Lines 124-129; P7: 130-132  P7: lines 137-138;  P12: Lines 245-247  P7: Lines 140-141  P7: line 146  P8: Lines 152-167  P9: Lines 173-193;  P10: lines 194-201  P9: Lines 189-193;  P10: Lines 194-201  P10: Line 207  P9: Lines 186-188;  P27: Lines 488-491;    P20: Lines 335-348;  P21: 349-358  P22: Lines 381-393; P23: 394-395  P26: Lines 465-468  P11: Line 236;  P32: Lines 659-663 |
| Reviewer 4 | I have carefully reviewed your manuscript titled "Effect of Intra-Dialytic Pedaling Exercise on Dialysis Adequacy: A Randomized Controlled Trial." The topic is clinically relevant and addresses an important area in dialysis patient care. However, several substantial concerns need to be addressed before the manuscript can be considered for publication.  4-1) There are significant differences in baseline characteristics (notably, age and education level) between the intervention and control groups. These confounders may influence patients' compliance, exercise capability, and ultimately dialysis adequacy. Please provide further explanation on how you have accounted for these baseline imbalances. If possible, conduct covariate-adjusted analyses (such as ANCOVA) or consider propensity score matching to minimize the potential confounding effects.  4-2) Some key findings (e.g., Kt/V improvement, p=0.11) did not reach statistical significance. Please be more cautious in your interpretation, emphasizing that, under your study conditions, no statistically significant effects were demonstrated, and discussing the risk of type II error given your sample size.  4-3) I recommend adding figures (e.g., line graphs or bar charts) to more intuitively present the changes in major outcomes (such as Kt/V and BUN) before and after the intervention in both groups. This will improve the readability and clarity of your results.  4-4) The manuscript currently lacks detailed information about exercise compliance, monitoring, and any adverse events during sessions. Please add a brief description of how session adherence was monitored, and whether any complications or dropouts related to exercise occurred. | 4-1) Thank you for your insightful comment regarding baseline differences between the intervention and control groups. We acknowledge that age and educational level may act as confounding variables, potentially influencing patients’ compliance, exercise capacity, and dialysis adequacy outcomes.  In response, we have clarified in the **Methods** section (under *Statistical Analysis*) that no covariate-adjusted analysis was performed due to sample size limitations, and all group comparisons were conducted using unadjusted statistical tests. Additionally, we have expanded the **Discussion** section to explicitly address this limitation, noting that the observed baseline imbalances may have introduced bias and affected the interpretation of results. We have also recommended that future studies consider covariate-adjusted analyses (e.g., ANCOVA) or propensity score matching to minimize confounding effects and enhance internal validity.  4-2) Thank you for your thoughtful observation regarding the interpretation of non-significant findings, particularly the modest improvement in Kt/V (p = 0.11). We fully agree that caution is warranted when interpreting results that do not reach statistical significance.  In response, we have revised the **Discussion** section to explicitly state that, under the conditions of this study, no statistically significant improvement in dialysis adequacy was demonstrated. We have also acknowledged the possibility of a Type II error due to the limited sample size, which may have reduced the statistical power to detect meaningful differences. Furthermore, we have recommended that future studies employ larger sample sizes and more robust designs to better assess the potential effects of intradialytic exercise.  4-3) Thank you for your valuable suggestion regarding the visual presentation of key outcomes. In response, we have added figures to illustrate the changes in BUN and Kt/V values before and after the intervention in both groups. These include a line graph for BUN levels and a bar chart for post-intervention Kt/V comparisons. References to these figures have been incorporated into the Results section to enhance clarity and support interpretation.  4-4) Thank you for your constructive feedback regarding reporting standards, exercise monitoring, and adverse events. We have addressed all three points as follows:  CONSORT Guidelines Citation: In response to your recommendation, we have revised the Study Design section (Lines 137-138) to explicitly state that the trial was designed and reported in accordance with the CONSORT 2010 guidelines. This ensures transparency and alignment with international reporting standards.  CONSORT Flowchart Resolution: The duplicate flowchart has been removed, and the remaining figure has been replaced with a high-resolution version (300 dpi) to ensure optimal clarity and readability in both print and digital formats. This update is reflected in the revised figure caption.  Exercise Compliance, Monitoring, and Adverse Events: We have added a detailed description in the Methods section under the subheading Exercise Protocol and Monitoring (Lines 194-198), explaining how session adherence was supervised by dialysis nurses. Vital signs were recorded before, during, and after each session, and patients were continuously monitored for any signs of discomfort or instability. In the Results section (Lines 278-280), we have clarified that no exercise-related adverse events occurred, and noted that five participants discontinued the intervention for reasons unrelated to exercise. | P14: Lines; 271-272  P26: Lines 465-468  P24: Lines 417-430;  P26: Lines 471-474  P18: Figure 2: Comparison of Mean Differences in Blood Urea Nitrogen (BUN) Levels Before and After Intervention in Intervention and Control Groups;  P19:Figure3: Comparison of post-intervention Kt/V values between the intervention and control groups.  P7: Lines 137-138;  P13: Figure 1: CONSORT FLOW DIAGRAM;  P10: Lines 194-201;  P14: Line 278;  P15: Lines 279-280 |

With thanks
