# Supplementary material for: Effect of intra-dialytic pedaling exercise on dialysis adequacy: A randomized controlled trial
Source: PLoS One. 2026 May 15;21(5):e0348063. doi: 10.1371/journal.pone.0348063 (PMC13178916; doi:10.1371/journal.pone.0348063)
Supplement: Attachment — Submitted filename: Response to Reviewers 1.docx [file pone.0348063.s011.docx]

Dear Editor

Respectfully, according to the comments of the esteemed referees, corrections were made in the text of the article as follows

| Source of Comment | Comments | Response to editor/ reviewer | Page/ line |
| --- | --- | --- | --- |
| Journal Requirements: | 1. If the reviewer comments include a recommendation to cite specific previously published works, please review and evaluate these publications to determine whether they are relevant and should be cited. There is no requirement to cite these works unless the editor has indicated otherwise. | Thank you for this comment. We have reviewed all suggested references and updated the manuscript accordingly, adding relevant new citations and removing or replacing older ones where appropriate. All references now directly support the methodology, discussion, and interpretation of our findings, ensuring accuracy, relevance, and compliance with journal guidelines. | ---------- |
|  | 2. Please review your reference list to ensure that it is complete and correct. If you have cited papers that have been retracted, please include the rationale for doing so in the manuscript text, or remove these references and replace them with relevant current references. Any changes to the reference list should be mentioned in the rebuttal letter that accompanies your revised manuscript. If you need to cite a retracted article, indicate the article’s retracted status in the References list and also include a citation and full reference for the retraction notice. | Thank you for this important comment. We have thoroughly reviewed the reference list to ensure completeness, accuracy, and relevance. Any retracted articles previously cited have been either removed or replaced with current, valid references, and any retained retracted articles are clearly indicated as such in the References list with a citation to the retraction notice. These changes have been made to ensure the scientific integrity and accuracy of the manuscript, and all modifications to the reference list are reflected in the revised submission. | ------------ |
|  | Thank you for submitting your clinical trial to PLOS One and for providing the name of the registry and the registration number. The information in the registry entry suggests that your trial was registered after patient recruitment began. PLOS One strongly encourages authors to register all trials before recruiting the first participant in a study.  As per the journal’s editorial policy, please include in the Methods section of your paper:  1) your reasons for your delay in registering this study (after enrolment of participants started);  2) confirmation that all related trials are registered by stating: “The authors confirm that all ongoing and related trials for this drug/intervention are registered | We sincerely thank the Editor for highlighting this important discrepancy. We apologize for the error in the study dates initially reported in the manuscript. There was a mistake in converting the Iranian calendar year (which started in March 2020) to the Gregorian calendar. The correct timeline is as follows:  The trial was registered on the IRCT on 05 May 2020.  Participant recruitment and the intervention were conducted prospectively, following registration, from June to August 2020.  The previously mentioned dates (January–March 2020) were incorrect and resulted from a calendar conversion oversight. We have now corrected the study timeline in the Methods section of the manuscript (see the "Study Design" and "Trial Registration" subsections) to reflect the accurate post-registration period. Furthermore, we have added the required statement confirming that all related trials are registered.  We confirm that no patient was enrolled or underwent any study procedures prior to the formal registration date. We appreciate the opportunity to correct this error and ensure the accuracy of our report. | Page: 7  Lines: 141-147 |
|  |  |  |  |
| Reviewee 3 | Major Comments  • My primary concern regarding vascular access is not its functional status per se, but  rather whether its type differed systematically between the randomized groups at  baseline. Given that different access types can directly and indirectly impact various  physical and psychosocial patient outcomes, this constitutes a potential source of bias.  I consider this a significant methodological limitation. Therefore, I strongly recommend  that the authors retrieve and report this baseline characteristic. This information should  be obtainable from the hemodialysis centers' records or the researchers' own  documentation. Presenting a comparison of vascular access type between study groups  is essential to assess the randomization integrity and the interpretability of the findings. | We thank the reviewer for this critical and insightful comment, which has significantly improved the transparency and rigor of our manuscript. We fully agree that baseline differences in vascular access type could be a source of confounding bias. We have addressed this concern through clarifications in the Methods and an enhanced discussion of the limitations, as detailed below.  Clarification in the Methods Section:  In direct response to the reviewer's suggestion to clarify data availability, we have comprehensively revised the "Treatment Uniformity and Vascular Access" subsection in the Methods. The revised text now explicitly states that detailed vascular access type was not part of the standardized records at the participating centers during the study period, precluding reliable extraction for formal analysis. It also reports our post hoc assessment, which indicated that arteriovenous fistulas were predominant and that no systematic difference between groups was apparent.  Enhanced Acknowledgement in the Limitations Section:  To ensure this methodological point is transparently communicated, we have strengthened the corresponding paragraph in the Limitations section of the Discussion. We now explicitly state that the absence of standardized baseline data on vascular access remains a limitation that could influence the interpretation of solute clearance outcomes.  Commitment in Future Directions:  Acknowledging the importance of this variable, we have added a sentence to the "Practical Implications and Future Directions" part of the Discussion, recommending that future studies incorporate prospective, standardized documentation of vascular access type.  We believe these revisions directly address the reviewer's concern by providing a clear explanation, enhancing transparency regarding the limitation, and outlining a path for improved methodology. The manuscript has been updated accordingly, with all changes highlighted. We are grateful for this valuable feedback. | page: 9  lines: 181-189  page: 23  lines: 453-457  page: 24  lines: 458-459 |
|  | • The primary objective of the study, which is prominently reflected in its title, is currently addressed only as the third topic within the Discussion section. This structural choice diminishes the prominence and analytical focus warranted by the main research aim. I strongly recommend restructuring the Discussion to address the primary objective first. The logical flow should follow the hierarchy of the study's aims: the central question should be discussed with the greatest depth and priority, followed by secondary and exploratory findings. | We thank the reviewer for this important and constructive comment regarding the structure and analytical focus of the Discussion section.  We fully agree that the primary objective of the study—the effect of intradialytic physical activity on dialysis adequacy—should be addressed first and with the greatest depth. Accordingly, the Discussion section has been thoroughly revised and reorganized to align with the study's hierarchy of aims. The revised Discussion now opens with a dedicated subsection on the primary outcome (dialysis adequacy, measured by Kt/V), followed by secondary findings and broader contextual considerations.  In addition to restructuring the Discussion, we have addressed the reviewer’s methodological concern regarding vascular access in the interpretation of the results. Specifically, potential heterogeneity related to unmeasured vascular access characteristics and function is now explicitly discussed as a factor that may have influenced solute clearance and attenuated the observed effects of intradialytic exercise. This consideration has been incorporated into the primary outcome discussion, rather than being confined solely to the Limitations section, to provide a more nuanced and transparent interpretation of the findings.  We believe that these revisions have strengthened the coherence, depth, and methodological rigor of the Discussion, while ensuring that both the primary study objective and key sources of potential bias are clearly acknowledged and analytically addressed. | Page: 19  Lines: 346-351  Page: 23  Lines: 436-439 |
|  | The discussion treats the choice of exercise intensity with insufficient depth. While it is briefly mentioned as a potential hypothesis for the absence of significant results, this aspect warrants a far more substantial and critical exploration, comparable to the detailed treatment given to exercise volume. | We thank the reviewer for this insightful comment, highlighting the need for a more in-depth and critical discussion of exercise intensity.  We agree that exercise intensity is a key determinant of the physiological response to intradialytic physical activity and should be discussed with the same level of detail as exercise volume. In response to this comment, we have substantially expanded the Discussion section to provide a more comprehensive analysis of exercise intensity and its potential role in shaping the study outcomes.  Specifically, we have added a dedicated discussion addressing the low- to moderate-intensity nature of the intervention, the clinical rationale for selecting this intensity to ensure patient safety and tolerance during dialysis, and the potential physiological limitations associated with insufficient metabolic and circulatory stimulation. The revised Discussion now explicitly considers how limited exercise intensity may have attenuated urea mobilization, muscle perfusion, and transmembrane solute transport, thereby contributing to the modest and non-significant changes observed in dialysis adequacy.  Furthermore, we have contextualized our findings within the existing literature by comparing exercise intensity across prior studies, highlighting that interventions employing higher or progressively overloaded intensities or multimodal exercise programs have reported more favorable effects on dialysis adequacy. We believe that these revisions have strengthened the analytical depth, balance, and interpretability of the Discussion section.  We appreciate the reviewer’s suggestion, which has led to a more nuanced and methodologically transparent interpretation of the study findings. | Page: 19  lines: 361-368  page: 20  lines: 373-375  page: 22  lines: 418-422 |
|  | The criteria used to define "moderate intensity" require clarification and justification. The described parameter—an increase of 10% above baseline heart rate—is not consistent with standard exercise prescription guidelines for this intensity domain. It seems to me to be more of a light/low intensity. | We thank the reviewer for this important, technically insightful comment on the definition and classification of exercise intensity.  We agree that an increase of approximately 10% above baseline heart rate does not fully align with conventional definitions of moderate-intensity exercise under standard exercise prescription guidelines. In response to this comment, we have clarified and revised the description of exercise intensity throughout the manuscript to ensure accuracy and consistency.  Specifically, the exercise protocol is now explicitly described as a conservative, light to low–moderate intensity intervention. In the Methods section, we have clarified how baseline heart rate was assessed and justified the selection of this intensity threshold based on patient safety, feasibility, and the need to minimize hemodynamic instability during hemodialysis. We have also revised the terminology used across the Abstract, Methods, Discussion, and Limitations sections to avoid overstating exercise intensity.  Furthermore, the exercise intensity's conservative nature is now explicitly acknowledged as a study limitation. The revised Discussion and Limitations sections critically address how the intentionally low intensity, while clinically appropriate in the intradialytic setting, may have limited the magnitude of physiological and biochemical responses and contributed to the modest effects observed on dialysis adequacy.  We believe these revisions have improved the manuscript's methodological transparency and strengthened the interpretation of the findings. We appreciate the reviewer’s comment, which has helped refine both the terminology and the study's scientific rigor. | Page: 10  Lines: 198-205  Page: 19  Lines: 361-364  Page: 22  Lines: 418-420 |
|  | A fundamental methodological consideration must be addressed: the study's design places it closer to an acute rather than a chronic exercise intervention paradigm. This critical aspect is currently absent from the discussion. The absence of significant changes cannot be attributed solely to the parameters of volume and intensity. The insufficient duration of the intervention to induce chronic physiological adaptations is a primary, and likely predominant, explanatory factor. | We sincerely thank the reviewer for this important, conceptually insightful comment, which highlights a fundamental methodological consideration in interpreting our findings.  We fully agree that the temporal nature of the exercise intervention represents a critical explanatory factor that extends beyond exercise volume and intensity alone. In response to this comment, we have substantially revised the Discussion section to explicitly frame the intervention within an acute rather than a chronic exercise paradigm.  Specifically, the revised Discussion now emphasizes that the four-week intradialytic exercise protocol was unlikely to provide sufficient duration to induce chronic physiological adaptations, such as sustained improvements in muscle perfusion, metabolic efficiency, or solute transport capacity. We clarify that the modest, non-significant changes observed in dialysis adequacy are more appropriately interpreted as a consequence of the limited intervention duration, rather than as being attributable solely to exercise dose parameters.  In addition, we have repositioned the study to primarily evaluate the short-term feasibility and acute physiological response to intradialytic physical activity, while acknowledging that longer-term, progressive exercise interventions are likely required to achieve chronic adaptations and clinically meaningful improvements in dialysis adequacy. Relevant comparisons with prior long-term studies have been incorporated to contextualize this interpretation.  We believe that integrating this acute-versus-chronic framework has strengthened the conceptual clarity and physiological rigor of the Discussion, and we are grateful to the reviewer for this valuable suggestion. | Page: 19  Lines: 349-351; 358-360; 366-368  Page: 20  385-388 |
|  | Minor comments | | |
| Introduction  Line 72 | The issue with acronyms has recurred, this time concerning kidney replacement  therapy (KRT). I strongly advise systematically reviewing this throughout all new sections of  the manuscript. Please ensure that all acronyms are explicitly defined upon first use (e.g.,  "kidney replacement therapy (KRT)"). Implementing this standard practice preemptively will  enhance manuscript clarity and eliminate the need for repetitive requests on this matter during  the review process. | Thank you for this valuable and constructive comment. We fully agree that a consistent and clear definition of acronyms is essential for manuscript clarity. Accordingly, we have carefully reviewed the entire manuscript, with particular attention to all newly added and revised sections, to ensure that every acronym is explicitly defined at its first occurrence. Specifically, kidney replacement therapy is now consistently introduced as “kidney replacement therapy (KRT)” upon first use, and this practice has been systematically applied to all other abbreviations throughout the manuscript. We believe these revisions have improved the manuscript's readability and overall clarity and have addressed this concern comprehensively. | Page: 4  Line: 67 |
| Lines 111 – 112 | The justification for selecting cycling as the primary intervention modality need  not be explicitly stated to the reader. There may have been some confusion stemming from my  previous comments. The intention is for the presented data and methodological rationale to  inherently justify the choice of exercise modality. I believe this is primarily a matter of  rephrasing the introductory clause of this paragraph to allow the subsequent information to  stand as its own justification.  As a suggested rephrasing for exemplification:  Cycling represents a practical and safe intervention modality that is well  suited to the physical constraints commonly experienced by patients  undergoing hemodialysis. | We appreciate the reviewer’s clarification. The paragraph has been shortened and rephrased to introduce cycling as a practical and safe intervention, with subsequent methodological details and references serving as implicit justification. | Page:6  Line: 111-116 |
| Lines 119 – 122 | Exercise volume is reported only for the intervention group that showed significant improvement, which may be perceived as somewhat selective reporting. To ensure completeness and transparency, I recommend standardizing this information. | We appreciate the reviewer’s valuable observation. In line with this comment, the introduction has been revised to include a balanced summary of previous studies, highlighting both those with short-term/low-intensity protocols that did not improve dialysis adequacy and those with longer or more structured interventions that reported favorable outcomes. Exercise volume has now been reported consistently across all referenced studies to ensure transparency and comparability. | Page: 6  Lines: 117-125 |
| Results  Table 1 | Table 1  The asterisk (*) is used within the table to denote specific statistical comparisons  (e.g., p < 0.05). It is therefore inappropriate and potentially confusing to reuse the same symbol  for the general table footnote. Please assign a distinct symbol (e.g., †, ‡, §) for the general  legend or present the footnote using a different format (e.g., a separate, non-symbol key or a  descriptive note below the table title).  Line 309  I suggest removing the entire text on line | We thank the reviewer for this observation. The footnote in Table 1 has been retained for clarity but revised to use a distinct symbol (†) instead of the asterisk, thereby avoiding any potential confusion. | Table 1  Page: 15,16 |
| Minor Comments  Lines 333 – 335 | Avoid using value-laden language when describing outcomes that showed no  statistically significant difference. If the statistical analysis confirms the absence of a significant  difference, the results should be presented neutrally. Describing non-significant data with terms  that imply a trend or a qualitative distinction (e.g., "slightly higher," "appeared better,"  "marginally lower") can be misleading and is not supported by the statistical evidence  presented. | We thank the reviewer for this valuable comment. The Result and Discussion section has been carefully revised in accordance with the reviewer’s suggestion. All expressions have been adjusted to ensure neutral, scientific wording, and the section has been rewritten to present the findings objectively and strictly in line with the statistical results. | Page: 17  Line: 327  Page: 18  Line: 333  Page: 19  Lines: 348-349; 358; 366-368  Page: 23  Lines: 436-439 |
| Lines 371 – 373 | I would like to respectfully reinforce the possibility that the inefficacy of the  protocol to generate significant changes may be attributed as much, if not more, to the exercise  intensity prescribed rather than solely to the total exercise volume. | We thank the reviewer for this insightful comment. As highlighted in the major comments, the Discussion section has been revised to acknowledge that the lack of statistically significant improvements in dialysis adequacy may be related not only to overall exercise volume but also to the prescribed intensity. This point has been incorporated into the interpretation of our findings to ensure that both exercise duration and intensity are considered as potential explanatory factors. | Page: 19  Lines: 361-366 |
| Line 379 | When citing other studies for comparative purposes, it is essential to provide the  reader with sufficient information to understand the similarities and key differences between  your protocol and those referenced. Throughout this section, a critical gap is evident: the  discussion lacks a concise comparative analysis of the very exercise parameters (volume and  intensity) that are central to your hypothesis. | We thank the reviewer for this important observation. As highlighted in the major comments, the Discussion section has been revised to include a clearer comparative analysis of our protocol in relation to previous studies, with specific attention to exercise parameters such as volume and intensity. This addition ensures that similarities and differences between our intervention and prior protocols are explicitly addressed, thereby strengthening the interpretation of our findings. | Page: 20  Lines: 369-379 |
| Lines 386 – 388 | The discussion of vascular access as a limitation should be substantially  shortened and made more factual. Rather than a speculative narrative, simply report the relevant  baseline characteristic. For example: state the number and percentage of patients using an  arteriovenous fistula versus a central venous catheter in each study group. | We thank the reviewer for this constructive comment. In line with the major comments, the Discussion section has been revised and the description of vascular access has been substantially shortened and presented factually. As the dataset lacked precise counts and percentages of patients with arteriovenous fistulas versus central venous catheters, we limited the statement to a concise acknowledgment of vascular access as a baseline characteristic and potential limitation, without speculative interpretation. | Page: 23  Lines: 453-457  Page: 24  Lines: 458-459 |
| Paragraph that starts in line 399 | How similar? Intensity? Volume? There are dozens of other studies with  prescribed aerobic exercise that report Kt/V, although they may not be concerned with focusing  on that, they need to characterize very well to what extent an intervention should be similar to  yours to be considered in the discussion. | We thank the reviewer for this insightful comment. In the revised Discussion section, we have clarified the comparison between our protocol and prior studies by explicitly addressing exercise parameters, including intensity and volume. Our intervention consisted of short-duration, low-intensity pedaling sessions, whereas studies reporting significant improvements in dialysis adequacy generally employed longer interventions with higher intensity or multimodal approaches. This addition highlights the extent to which our protocol differs from those studies and strengthens the interpretation of our findings in relation to the existing literature. | Page: 6  Line: 117,118 |
| Line 478 | This section assumes and proceeds with the classification of the exercise  modality as low intensity. This represents a direct contradiction to its description elsewhere in  the manuscript (e.g., as "moderate intensity"). This internal inconsistency must be resolved.  Furthermore, the definitive classification of exercise intensity must be explicitly and  unambiguously stated within the Methods section, specifically in the description of the exercise  protocol. | Thank you for this comment. The manuscript has been revised to fully resolve the inconsistency regarding exercise intensity. The intradialytic cycling intervention is now explicitly and definitively classified as light-intensity physical activity in the Methods section (Exercise Protocol). Exercise intensity was prescribed to elicit approximately a 10% increase in heart rate from baseline, consistent with a light workload in the hemodialysis population, and intentionally selected to ensure patient safety and hemodynamic stability during dialysis.  All references to moderate-intensity exercise have been corrected, and the Discussion has been revised accordingly to interpret the findings in the context of the intervention’s light intensity and short duration. These revisions ensure full internal consistency and methodological clarity. | Page: 10  Lines: 198-205 |
| Flowchart | I recommend modifying the participant flowchart to clearly separate individuals who  were ineligible (did not meet the inclusion/exclusion criteria) from those who were eligible but  declined to participate (refused). This distinction is methodologically important.  Providing this granularity enhances the transparency of your recruitment process and  the reproducibility of your study. Furthermore, it increases the potential utility and citation  frequency of your work. Future research—for instance, studies analyzing acceptance rates or  barriers to participation in interventional trials among the hemodialysis population—would rely  on such clearly disaggregated data. A mere detail in presentation can determine whether a  subsequent systematic review or meta-analysis can (or cannot) incorporate your findings. | Thank you for this constructive suggestion. The CONSORT flowchart has been revised to clearly distinguish between individuals who were ineligible based on inclusion/exclusion criteria and those who were eligible but declined to participate. This disaggregation enhances the transparency of the recruitment process and improves the methodological clarity and reproducibility of the study. We agree that this level of detail strengthens the utility of the flowchart for future research and systematic evidence synthesis. | Diagram file |

With thanks
